# Assessing various Infrared (IR) microscopic imaging techniques for post-mortem interval evaluation of human skeletal remains

**DOI:** 10.1371/journal.pone.0174552

**Published:** 2017-03-23

**Authors:** Claudia Woess, Seraphin Hubert Unterberger, Clemens Roider, Monika Ritsch-Marte, Nadin Pemberger, Jan Cemper-Kiesslich, Petra Hatzer-Grubwieser, Walther Parson, Johannes Dominikus Pallua

**Affiliations:** 1 Institute of Legal Medicine, Medical University of Innsbruck, Innsbruck, Austria; 2 Material-Technology, Leopold-Franzens University Innsbruck, Innsbruck, Austria; 3 Division for Biomedical Physics, Medical University of Innsbruck, Innsbruck, Austria; 4 Department of Pharmaceutical Technology, Institute of Pharmacy, Leopold Franzens University of Innsbruck, Innsbruck, Austria; 5 Interfaculty Department of Legal Medicine, University of Salzburg, Salzburg, Austria; 6 Department of Pathology, Medical University of Innsbruck, Innsbruck, Austria; Aligarh Muslim University, INDIA

## Abstract

Due to the influence of many environmental processes, a precise determination of the post-mortem interval (PMI) of skeletal remains is known to be very complicated. Although methods for the investigation of the PMI exist, there still remains much room for improvement. In this study the applicability of infrared (IR) microscopic imaging techniques such as reflection-, ATR- and Raman- microscopic imaging for the estimation of the PMI of human skeletal remains was tested. PMI specific features were identified and visualized by overlaying IR imaging data with morphological tissue structures obtained using light microscopy to differentiate between forensic and archaeological bone samples. ATR and reflection spectra revealed that a more prominent peak at 1042 cm^-1^ (an indicator for bone mineralization) was observable in archeological bone material when compared with forensic samples. Moreover, in the case of the archaeological bone material, a reduction in the levels of phospholipids, proteins, nucleic acid sugars, complex carbohydrates as well as amorphous or fully hydrated sugars was detectable at (reciprocal wavelengths/energies) between 3000 cm^-1^ to 2800 cm^-1^. Raman spectra illustrated a similar picture with less ν_2_PO_4_^3−^at 450 cm^-1^ and ν_4_PO_4_^3−^ from 590 cm^-1^ to 584 cm^-1^, amide III at 1272 cm^-1^ and protein CH_2_ deformation at 1446 cm^-1^ in archeological bone material/samples/sources. A semi-quantitative determination of various distributions of biomolecules by chemi-maps of reflection- and ATR- methods revealed that there were less carbohydrates and complex carbohydrates as well as amorphous or fully hydrated sugars in archaeological samples compared with forensic bone samples. Raman- microscopic imaging data showed a reduction in B-type carbonate and protein α-helices after a PMI of 3 years. The calculated mineral content ratio and the organic to mineral ratio displayed that the mineral content ratio increases, while the organic to mineral ratio decreases with time. Cluster-analyses of data from Raman microscopic imaging reconstructed histo-anatomical features in comparison to the light microscopic image and finally, by application of principal component analyses (PCA), it was possible to see a clear distinction between forensic and archaeological bone samples. Hence, the spectral characterization of inorganic and organic compounds by the afore mentioned techniques, followed by analyses such as multivariate imaging analysis (MIAs) and principal component analyses (PCA), appear to be suitable for the post mortem interval (PMI) estimation of human skeletal remains.

## 1. Introduction

After discovery of skeletonized human remains or individual bones, the most important apprehension for investigators and legal authorities is to distinguish forensic material from archaeological material [1],[2]. Therefore, it is crucial to determine the post-mortem interval (PMI) as precisely as possible. PMI estimation usually starts with the macroscopic examination of the bone material, together with the consideration of the gross appearance, tissue preservation and odour. Due to the fact that these features are influenced by many environmental factors (temperature, body size, accessibility for insects or animals, location of the body etc.), which can impinge on the decomposition process, the estimation of the exact PMI is very difficult [3], [4], [5]. Thus, the differentiation between forensic and anthropological material can be quite challenging and often depends on the experience of the investigator.

Techniques for the investigation of the PMI include microscopic methods [6], [7], [8], chemiluminescence tests, such as the luminol reaction [2],[9],[10], radiocarbon techniques [3, 11], chemical methods, spectroscopical analysis [12], [13], macroscopic UV fluorescence [14] and the detection of various radionuclides [15], [16], [17, 18].

The usage of different reflection infrared (IR) microscopic imaging methods for PMI research might provide a useful adjunct to conventional methodologies. IR and Raman spectroscopy has already demonstrated great promise for the characterization of bone specimens [12, 19–23]. The primary aim of our study is to test the suitability of these techniques for analyzing organic and inorganic components of bone material and to subsequently differentiate between forensic and archaeological bone material.

For this purpose IR microscopic imaging techniques, such as infrared (IR) reflection-, ATR- and Raman-microscopic imaging, followed by multivariate data analyses (MIAs), were combined in order to achieve a more sophisticated characterization of human skeletal remains.

These modern analytical techniques enable molecular imaging of complex samples and are based on the absorption of infrared radiation by vibrational transitions in covalent bonds [24]. The major advantage of these methods is the acquisition of unique images of the in situ distribution of proteins, lipids, carbohydrates, cholesterols, nucleic acids, phospholipids and small molecules with high spatial resolution whilst maintaining the topographic integrity of the tissue and avoiding time-consuming extraction, purification and separation steps [25]. These techniques also allow samples to be probed under native conditions and provides unique chemo-morphological information about the tissue status without the need for fixation, staining or application of additional markers [26]. With these methods it is possible to gain qualitative and quantitative information from heterogeneous samples, since the individual IR spectrum of any compound represents a unique `molecular fingerprint´ [27, 28]. IR microscopic imaging has already been utilised with great success for the characterization of biological specimens [29–42], malignancies in several tissues [25, 43–56], in environmental mapping [57], precision farming [58], food quality evaluation [59], product functionality [60, 61], for ascertaining the severity of plant diseases [62, 63], detecting defects [64] and contaminations [65–67], as well as for assessing the distribution of certain chemical components [32, 39, 68–70]. Hence there is good reason to believe that IR microscopic imaging measurements may also be applicable for PMI estimation of human bones through the evaluation of molecular distribution patterns.

## 2 Material and methods

### 2.1 Sample collection

Archaeological bone samples of different ages from an excavation site covering several centuries in European medieval times (n = 2) and forensic bone samples (n = 4) were investigated. Gender identifications were confirmed by DNA analyses. For this study, the part between the upper and mid third of the femur was used. Employing an oscillating bone saw a transverse section was cut to a thickness of about 7 mm from each bone. Cross sections were polished with 1200 grit carbide paper prior to the measurements. All examined samples were anonymized before the authors had access to the specimens. Anthropological properties and place of discovery of all measured human skeletal remains are summarized in Table 1 and additionally described elsewhere [41, 71, 72]. Detailed information concerning the archaeological bone samples is given in Table 2. Archaeological bone samples were provided by the Museum of Industry and Prehistory in Wattens and permission was granted by the ‘Staatssammlung für Anthropologie und Paläoanatomie MUC ‘(SAPM, Munich, Germany). Detailed information about sex identification, specimen number, complete repository information, museum name(s) and geographic location is published in [71, 72] and stated in the supplementary (S1 and S2 Figs, S1 Table and S1 File). Forensic bone samples were provided by the Institute of Legal Medicine (Medical University of Innsbruck). Bone extraction was executed according to the specifications/standards required by public prosecution for DNA extraction and identity determination. All necessary permits were obtained for the described study in agreement with Austrian legislation, which complied with all relevant regulations.

**Table 1:** Anthropological properties and place of discovery of the measured human skeletal remains.

*Samples*	*Sex*	*PMI*	*Age*	*Find Spot*
				
*Forensic*	Male	1 day^+^	Adult	Canyon
*Forensic*	Male	3 years^+^	Adult	Forest
*Forensic*	Male	25 years^+^	Adult	Glacier
*Forensic*	Male	85 years^+^	Adult	Cave / Duct
*Archaeological*	Male	650–870 years*	Adult	Grave / Soil
*Archaeological*	Male	1030–1260 years*	Adult	Grave / Soil

^+^ missing person;

* radiocarbon dated

**Table 2:** Properties of archaeological bone samples.

*Grave*	*Radiocarbon Dating*	*Morph*. *Age*	*Sex morph*.	*Molecular genetic sex typing*	*Height (cm)*	*Burial orientation*
***16***	1030–1260 years	46–50	m	X, Y	161.4	Extended position, orientation east-west with head at the west end. Skeleton in humus layer.
***144***	650–870 years	60–70	m	n.d.	169.1	Extended position, orientation north-south with head at the north end. Directly parallel to the garden wall.

### 2.2 MIR reflection and ATR microscopic imaging

Mid infrared (MIR) reflection and ATR (Attenuated Total Reflection) microscopic imaging measurements were performed at room temperature using a LUMOS Fourier transform infrared (FTIR) microscope (Bruker) with an integrated FTIR spectrometer, equipped with a photoconductive MCT detector with liquid nitrogen cooling. Visual image collection was performed via a fast and highly resolving digital CCD camera.

The bone specimens for PMI estimation were detected with a nominal lateral pixel resolution a) of 20 μm × 20 μm for MIR reflection and b) of 10 μm × 10 μm for ATR using the MCT detector. Measurements were done in a spectral range from wave numbers of 4000 cm^-1^ to 600 cm^-1^. Spectra were recorded with a spectral resolution of 2 cm^-1^ with 256 co-added scans for reflection and 128 scans for ATR.

Prior to each MIR reflection and ATR sample measurement, a background spectrum of a gold-coated substrate was recorded with a spectral resolution of 2 cm^-1^ with 256 co-added scans.

Scan number and spectral resolution were optimized in order to achieve a suitable signal to noise ratio within the recorded spectra. For detailed information about detector theory, technology and current developments see references [73–75]. After background reduction, each sample was scanned with a light microscope (LM) for histological reevaluation and comparison to the imaging results.

### 2.3 Raman imaging

Raman imaging measurements were performed in reflection mode using a WITec ALPHA300R microscope at room temperature. For the Raman excitation in the near-infrared a Toptica XTRA laser with a nominal wavelength of 785 nm and a power of 15 mW, measured at the back aperture of the objective (Zeiss EC EPIPLAN 50x/0.7), was used. Because of the large size of the sample and the structures within, it was possible to choose parameters that reduced the measurement time for a single scan, i.e. a fibre with a core diameter of 100 μm to deliver the collected light to a spectrometer and a distance between scan points of 1 μm, which does not yield the highest possible lateral resolution of the microscope. The signal was analysed in the spectral range between 0 cm^-1^ and 1776 cm^-1^ with a spectral resolution of about 6 cm^-1^. The integration time for a single scan point was optimized near the scan region prior to each measurement to yield a good signal to noise ratio without causing damage to the sample.

### 2.4 Data processing

All spectral data processing and image assembling were performed using the OPUS 6.5 software (Bruker), The Unscrambler X 10.3 (Camo, Norway, Oslo) and the CytoSpec^™^ software package (http://www.cytospec.com, Hamburg, Germany). Univariate chemical maps, depicting a single spectral feature and multivariate imaging analysis (MIAs) were generated by using the OPUS 6.5 (for MIR reflection and ATR microscopic imaging) and CytoSpec^™^ (Raman) software.

### Principal Component Analyses (PCA)

Noise and atmospheric absorptions were removed using the CytoSpec^™^ software in the run-up to principle component analyses (PCA) and image analysis. Subsequently PCA models were generated with The Unscrambler X 10.3 software. For PCA model generation tissue type-associated spectra were selected with the CytoSpec^™^ software and then ROIs (regions of interest) were assigned. Selected spectra of ROIs were imported into The Unscrambler X 10.3 software and underwent several data pretreatments (e.g., baseline correction, normalization) prior to PCA model generation.

### Image analysis

Initially, atmospheric correction and noise reduction were performed: a) by using the OPUS 6.5 software (Bruker) for MIR reflection and ATR microscopic imaging and b) by using the WITec Control software. After spectral refinement sample specific data sets were loaded in the CytoSpec software. Spectra of MIR reflection and ATR microscopic imaging were vector normalized in the wave number range 4000 cm^-1^ to 600 cm^-1^. This procedure led to more pronounced peaks, eliminated background slopes and reduced the influence of intensity changes caused by differences in tissue density and tissue roughness [76]. The processed spectral datasets were used for subsequent MIAs. Furthermore, the imaging results were assembled and compared directly with the LM images captured from the same samples.

## 3 Results and discussion

The main focus of this study was to assess the utility of different reflection IR microscopic imaging methods applied to human bone sections for the determination of the as well as evaluating of the significance of the analytical methods employed.

A total of 6 human bone samples were investigated. Basic anthropological data of the samples are summarized in Tables 1 and 2.

**Fig 1:**
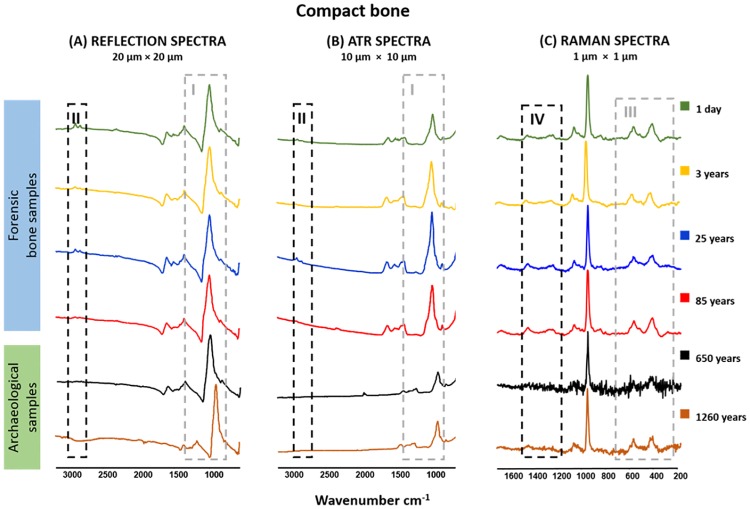
Representative reflection (A) -, ATR (B)—and Raman (C)—spectra of forensic and archaeological bone samples are shown. II = phospholipids, proteins, nucleic acid sugars, complex carbohydrates as well as amorphous or fully hydrated sugars at 3000 cm^-1^ to 2800 cm^-1^, I = indicator for bone mineralization at 1042 cm^-1^ and carbohydrates at 1185 cm^-1^, IV = protein CH_2_ deformation at 1446 cm^-1^ and amide III at 1272 cm^-1^, III = of ν_2_ PO_4_^3−^ at 450 cm^-1^ and ν_4_ PO_4_^3−^ from 590 cm^-1^ to 584 cm^-1^.

**Fig 2:**
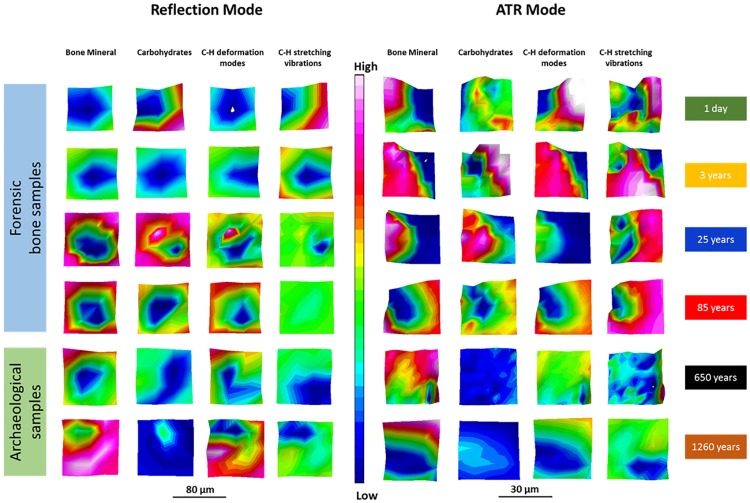
Infrared spectroscopic chemi-maps obtained for the detection of bone mineral at 1042 cm^-1^, of carbohydrates at 1185 cm^-1^, of C-H deformation modes at 1450 cm^-1^ to 1350 cm^-1^ and of phospholipids, proteins, nucleic acid sugars, complex carbohydrates as well as amorphous or fully hydrated sugars at 3000 cm^-1^ to 2800 cm^-1^.

In this study, six bone samples with various PMI from approx. 1 to 1000 years as shown in Table 1 were analysed by spectra-analysis, individual chemi-map representations, MIAs (multivariate imaging analyses) and PCAs (principal component analyses) (Figs 1 and 2). MIAs were also performed with reflection and ATR data, but there was no obvious difference in histological structures (data not shown).

Fig 1A and 1B depict reflection- and ATR- spectra of the six bone samples from the compact bones. A significant spectral feature in archaeological samples is the absorption band at 1042 cm^-1^ (indicator for bone mineralization, Fig 1I). The peak from archaeological specimens is sharp and more obvious in relation to the forensic ones. Furthermore, forensic bone samples exhibit more organic bands in comparison to archaeological ones. This could be seen by a reduction of carbohydrates at 1185 cm^-1^ (only seen after magnification in Fig 1I, data not shown), phospholipids, proteins, nucleic acid sugars, complex carbohydrates as well as amorphous or fully hydrated sugars at 3000 cm^-1^ to 2800 cm^-1^ (Fig 1II) in archaeological bone material as described previously [41] [77] [23]. Fig 1C shows Raman spectra from the aforementioned bone samples, and displays a similar pattern to that described above. In archaeological bone material a reduction of ν_2_PO_4_^3−^at 450 cm^-1^ and ν_4_ PO_4_^3−^ from 590 cm^-1^ to 584 cm^-1^ (Fig 1III), amide III at 1272 cm^-1^ (Fig 1IV) and protein CH_2_ deformation at 1446 cm^-1^ (Fig 1IV), compared to forensic samples, could be identified. These observations are in agreement with the Raman spectroscopy results of [78–80].

### 3.1 MIR reflection and ATR microscopic imaging

Results from chemi-maps are illustrated in Fig 2. The result of data analyses illustrates the potentials of reflection and ATR imaging to mirror decomposition / aging processes of bone samples with a nominal lateral resolution a) of 20 μm × 20 μm for MIR reflection and b) of 10 μm × 10 μm for ATR. Fig 2. depicts chemical maps generated by integrating the area under the band absorption at 1042 cm^-1^ (bone mineral), at 1185 cm^-1^ (carbohydrate bands), at 1450 cm^-1^ to 1350 cm^-1^ (C-H deformation modes) and at 3000 cm^-1^ to 2800 cm^-1^ (C-H stretching vibrations of phospholipids, of proteins, nucleic acid sugars, complex carbohydrates as well as that of amorphous or fully hydrated sugars). The results (ATR) demonstrate that archaeological bones exhibit a lower amount of carbohydrates (1185 cm^-1^), phospholipids, proteins, nucleic acid sugars, complex carbohydrates as well as amorphous or fully hydrated (3000 cm^-1^ to 2800 cm^-1^) sugars than the forensic ones. Chemi Maps (reflection mode) at the absorption band at 3000 cm^-1^ to 2800 cm^-1^ show less (a reduced) signal after a PMI of 3 years. The signal intensity at 1185 cm^-1^ (carbohydrates) seems to be weaker in the archaeological bones compared to the forensic samples. The 3 year sample in the reflection mode is likely to be an outlier. The chemical maps of both modes (reflection and ATR) of the absorption of bone mineral and C-H deformation modes indicate an inhomogeneous, similar distribution. As a consequence of these chemi-map investigations it can be conlcuded, that carbohydrates 1185 cm^-1^ and complex carbohydrates as well as amorphous or fully hydrated sugars 3000 cm^-1^ to 2800 cm^-1^ are more suitable for PMI estimation than peaks at bone mineral compounds 1042 cm^-1^ and at C-H deformation modes 1450 cm^-1^ to 1350 cm^-1^.

### 3.2 Raman imaging

**Fig 3:**
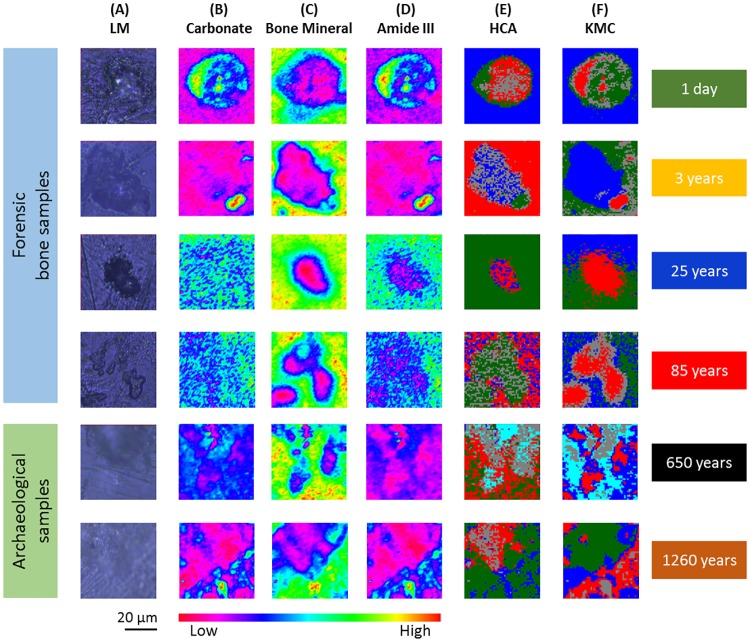
**(A)** Light microscopy images of 6 bone samples. **(B-D)** Raman imaging results shown in false colour representation, where the colours reflect the intensities of the absorption at 756 cm^-1^, which is commonly attributed to B-type carbonate **(B)**, the absorption at 957 cm^-1^, which is commonly attributed to bone mineral, containing extensive HPO_4_ **(C)**, and the absorption at 1272 cm^-1^, which is commonly attributed to protein α-helix **(D)**. **(E)** Hierarchical cluster analysis. **(F)** K-means clustering image.

Chemi maps and MIAs generated for six individual bone samples are illustrated in Fig 3. The output of the data analyses illustrates the ability of Raman imaging to reflect decomposition / aging processes of bone samples with a nominal lateral resolution of 1 μm × 1 μm per pixel for each spot.

The images in Fig 3(A) represent micrographs of bone samples taken after measurement with Raman microscopic imaging. These micrographs serve as comparative references for images constructed from chemical maps (Fig 3(B)–3(D)) and MIAs (Fig 3(E) and 3(F)). Different histo-anatomical features can be recognized in Fig 3(A): the osteon or Haversian system, containing blood and nerve supplies and mineralized connective tissue. Fig 3(B) depicts chemical maps generated by integrating the area under the band absorption at 756 cm^-1^, which is an indicator of B-type carbonate. These results demonstrate that B-type carbonate cannot be detected after a PMI of 3 years. The chemical maps of the absorption at 957 cm^-1^ refers to bone mineral, (Fig 3(C)) containing extensive HPO_4_. Even after detailed evaluation only minimal differences were seen. The chemical maps resulting from the absorption at 1272 cm^-1^ (Fig 3(D)) represent a higher signal in forensic samples, which is commonly attributed to protein α-helix. These observations indicate a large reduction in the levels of biomolecules within the osteon after a PMI of 3 years. Nevertheless, specific relationships between histological and morphological features could not be recognized with this type of data processing. Thus, various cluster analyses were performed to characterize the full range of observed spectral variations.

Fig 3(E) depicts pseudo-colour images, constructed by using HCA. The displayed images represent a four-cluster structure reproducing histo-anatomical features of the measured samples. K-means clustering images are displayed in Fig 3(F). The principal correspondence between the optical images and the K-means clustering images is obvious; most of the spectral clusters can be assigned to the histo-anatomical features. It was possible to reach a conclusion on the principal differentiation between the forensic samples, with their better-preserved bone structures, and the archaeological material, but a more detailed discrimination has not been possible so far, even when the number of clusters was increased (data not shown).

### 3.3. Mineral content ratio and the organic to mineral ratio of the IR imaging data

**Fig 4:**
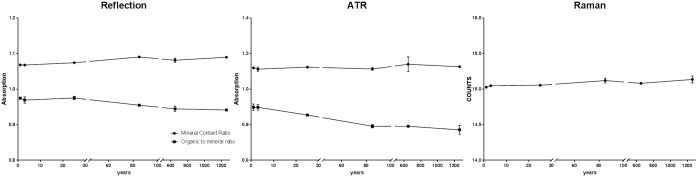
The mineral content ratio and the organic to the mineral ratio as a function of PMI is shown. For Raman microscopic imaging only the mineral content ratio was determined. Mapping results of mineral content ratio and organic to mineral ratio is given in the supplementary S3 Fig.

Since peak ratios are in this context more meaningful than absolute peak values, the mineral content ratio (MINCON = ν_4_ (PO_4_)^3-^ peak /Amide I peak) and the organic to mineral ratio (CH-aliphatic peak/phosphate peak) were calculated from the obtained reflection, ATR and Raman spectra. The mineral content ratio provides information about the change in the Amide I content. This is associated with the mineralized collagen present in the bone to the phosphate content of the mineralized bone. The amount of lipids and other organic material in the bones is displayed by the organic to the mineral ratio. Fig 4 illustrates ratio results as a function of PMI for human skeletal remains. For Raman microscopic imaging only the mineral content ratio was determined. During the age-related degradation, the mineral content ratio increases while the organic to mineral ratio decreases with time.

### 3.4. PCA analyses of the IR imaging data

**Fig 5:**
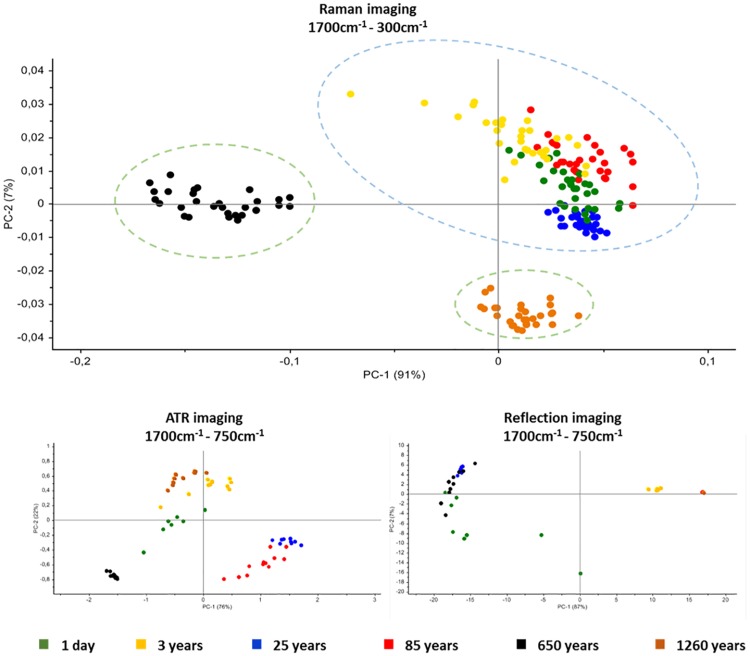
Score plot of PC1 and PC2 of Raman imaging, ATR imaging and reflection imaging spectra representation; each data point represents one spectrum of the respective (colour coded) bone sample.

For a complete characterization of the range of spectral variations principal component analyses were performed. With this type of data processing, the dimensionality of MIR microscopic imaging spectra is decreased, while as much information as feasible is retained. The scores of the first principal components are used to create significant plots without extensive comprehension of the underlying sample biochemistry. For the PCA across 6 different bone samples, 30 spectra were chosen for reflection-, ATR- and Raman- imaging. The results of spectral analyses using PCA are depicted in Fig 5. The score plot of the first and the second principal component is based on 30 spectra of the different bone samples. The score plots display a 2-D visualization of spectra clusters for the principal components 1 and 2. PCA models indicate that most of the descriptive information is contained in the region from 1700 cm^-1^ to 300 cm^-1^ (Raman imaging) and in the region from 1700 cm^-1^ to 750 cm^-1^ (reflection- and ATR imaging). The best separation between forensic and archaeological samples was achieved with the Raman imaging data. These findings can be explained by the capability of Raman to excite molecules more efficiently than other techniques and therefore yield a better signal with less interference from water.

Raman spectroscopy investigates molecular vibrations to provide a molecular fingerprint with inelastic scattering. In contrast, Fourier transform infrared spectroscopy (FTIR) is a form of vibrational spectroscopy that is based on the absorbance, transmittance or reflectance of infrared light. Thus, the light absorbance varies in a sample at various frequencies and ultimately matches with the vibrational frequencies of the bonds in the sample.

Raman spectroscopy is based on an alteration in polarizability and measures relative frequencies at which radiation is scattered by the sample and is sensitive to homo-nuclear bonds and thus can differentiate between C-C, C = C and C≡C (O = C) bonds. In contrary, IR spectroscopy reflects modifications of the dipole moment and measures the absolute frequencies at which radiation is absorbed by the sample and is sensitive to hetero-nuclear functional group vibrations and polar bonds, especially OH stretching in water. Therefore, Raman and FTIR spectroscopy differ in several basic features. Each method has its benefits and disadvantages, but combining both approaches has proven to be an efficient tool in the characterization of the PMI [73, 75, 81].

## 4. Conclusion

In order to further develop the current state of the art in PMI estimation we tested the suitability of various infrared (IR) microscopic imaging techniques regarding their ability to make conclusions about the PMI of human skeletal remains by analysing their organic and inorganic components. Various IR microscopic imaging techniques such as infrared (IR) reflection-, ATR- and Raman- microscopic imaging evaluated by multivariate data analyses (MIAs), were combined together as a means for a more sophisticated characterization of human skeletal remains.

Spectra (reflection and ATR) at 1042 cm^-1^ (indicator for bone mineralization) indicated a more prominent peak in archeological bone material compared to forensic ones. Furthermore, a reduction in phospholipids, proteins, nucleic acid sugars, complex carbohydrates as well as amorphous or fully hydrated sugars at 3000 cm^-1^ to 2800 cm^-1^ in archaeological bone material could be demonstrated. Raman spectra illustrated a similar picture with less ν_2_ PO_4_^3−^ at 450 cm^-1^ and ν_4_ PO_4_^3−^ from 590 cm^-1^ to 584 cm^-1^, amide III at 1272 cm^-1^ and protein CH_2_ deformation at 1446 cm^-1^ in archeological specimens.

Chemical maps from reflection and ATR imaging determined (highlighted) that the levels of carbohydrates 1185 cm^-1^ and complex carbohydrates together with amorphous or fully hydrated sugars 3000 cm^-1^ to 2800 cm^-1^ are higher in forensic bone samples than in archaeological bones. It can be assumed that this observation is due to diagenetic decomposition and aging processes. Therefore, the semi-quantitative analysis of these biomolecules could serve as an estimation of PMI.

Results from Raman microscopic imaging exhibit less B-type carbonate at 756 cm^-1^ and also a lower amount of protein α-helix at 1272 cm^-1^ in bones with a PMI of more than 3 years, indicating a decrease in these biomolecules within the first years post mortem. Findings concerning bone mineral compounds were inconclusive, most likely due to mineral uptake during diagenesis from the surroundings.

To gain deeper insight into the histo-anatomical features of bones, cluster analyses were performed. Results from reflection and ATR measurements did not reveal any relations to histological structures, however, spectral clusters from Raman microscopic imaging corresponded well to histo-anatomical features. Nonetheless, an in-depth differentiation could not be achieved in general.

Furthermore, the mineral content ratio and the organic to mineral ratio were calculated from the obtained reflection, ATR and Raman spectra. Results are similar to the findings published by Creagh et al.[22]: during the age-related degradation, the mineral content ratio increases while the organic to mineral ratio decreases with time.

For a complete characterization of spectral variations in correlation with the PMI, PCAs were carried out, suggesting that most of the significant information can be found in the wavelength number region of 1700 cm^-1^ to 750 cm^-1^ (reflection- and ATR imaging) and 1700 cm^-1^ to 300 cm^-1^ by Raman imaging. The most promising segregation of forensic and archaeological bone samples was achieved with Raman imaging.

Taken together, our results show that the differentiation of forensic and archaeological bone material is possible by the use of the aforementioned techniques. In the context of forensics this aspect is most important because of the various legal implications. We therefore suggest to use IR/Raman microscopic imaging techniques as another important tool in the field of PMI estimation.

We hope that this study will serve as a basis for additional developments in the field of PMI estimation. A number of questions such as the impact of environmental influences still need to be investigated in more detail. We anticipate that future studies will enable the PMI to be narrowed down further by analysing a bigger sample size taken from different decades, although obtaining an adequate and comparable sample size is still a major challenge.

## Supporting information

S1 Fig Simplified burial plan. (JPG)

S2 Fig Complete (a) and close-up (b) view of a skeleton from grave number 123. (JPG)

S3 Fig Infrared spectroscopic chemi-maps obtained of Infrared (IR) reflection-, ATR- and Raman- microscopic imaging data. Mineral content ratio and organic to mineral ratio are displayed for reflection- and ATR- microscopic imaging measurements. For Raman microscopic imaging only the mineral content ratio was determined.
(TIF)

S1 Table Summary of morphological and genetic sex-typing results. (DOCX)

S1 File Using 13C-, 15N- and 18O stable isotope analysis of human bone tissue to identify transhumance, high altitude habitation and reconstruct palaeodiet for the early medieval Alpine population at Volders, Austria. (PDF)

## Abbreviations

ATR: Attenuated Total Reflection
CCD: Charge coupled device
DNA: Deoxyribonucleic acid
FTIR: fourier transform infrared
HCA: hierarchical cluster analysis
IR: infrared
KMC: K Means Clustering
LM: light microscope
MCT: mercury cadmium telluride
MIAs: multivariate imaging analyses
MIR: mid infrared
PCA: principal component analyses
PMI: post-mortem interval
ROIs: regions of interest
UV: ultraviolet
