# Supplementary figures and images for: Assessing various Infrared (IR) microscopic imaging techniques for post-mortem interval evaluation of human skeletal remains
Source: PLoS One. 2017 Mar 23;12(3):e0174552. doi: 10.1371/journal.pone.0174552 (PMC5363948; doi:10.1371/journal.pone.0174552)

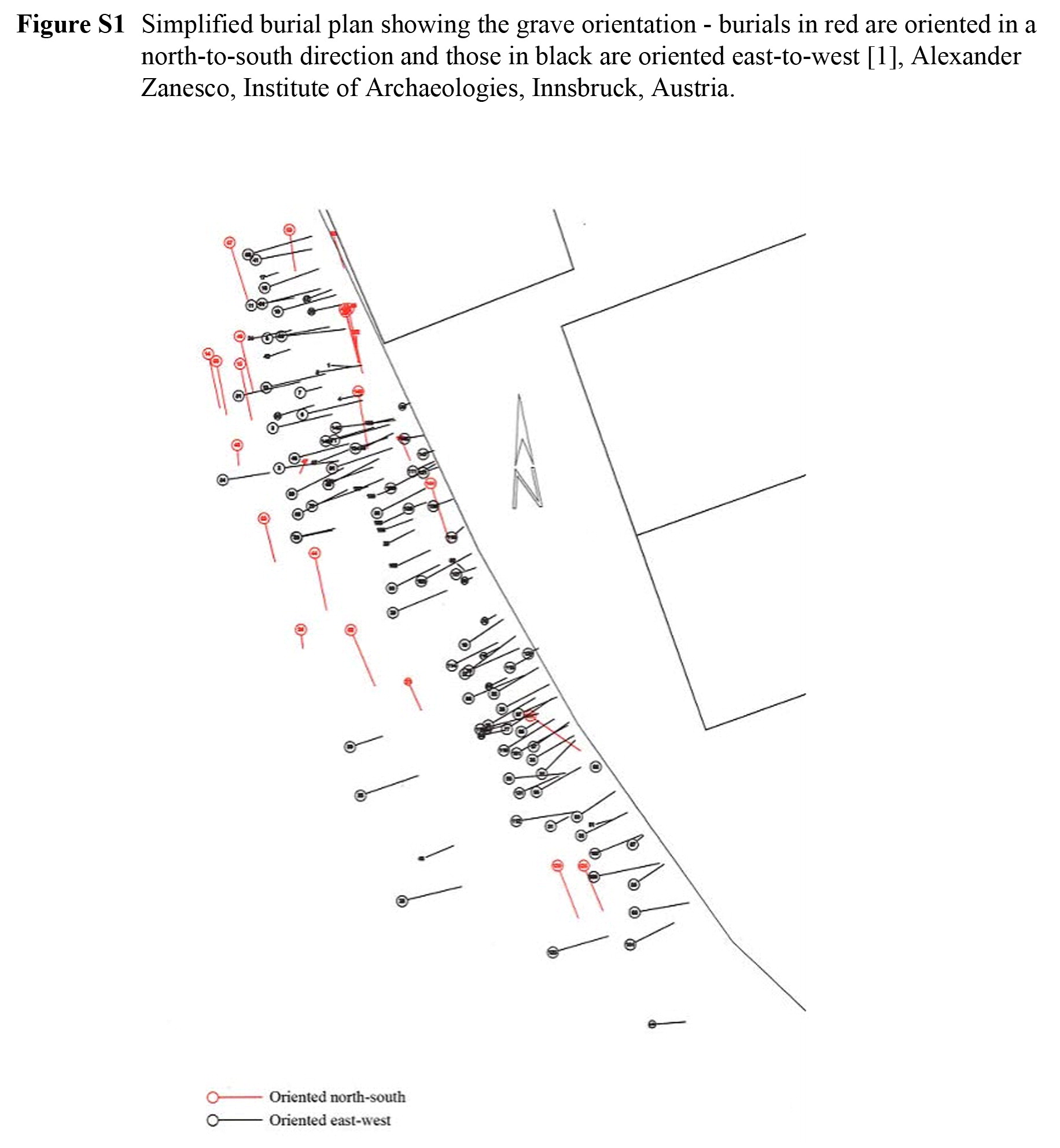

Supplement: S1 Fig — (JPG) [file pone.0174552.s001.jpg]

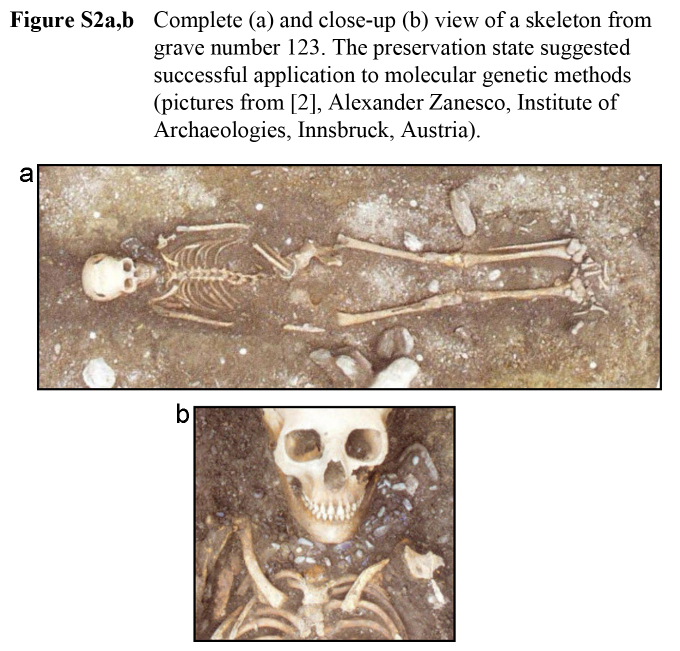

Supplement: S2 Fig — (JPG) [file pone.0174552.s002.jpg]

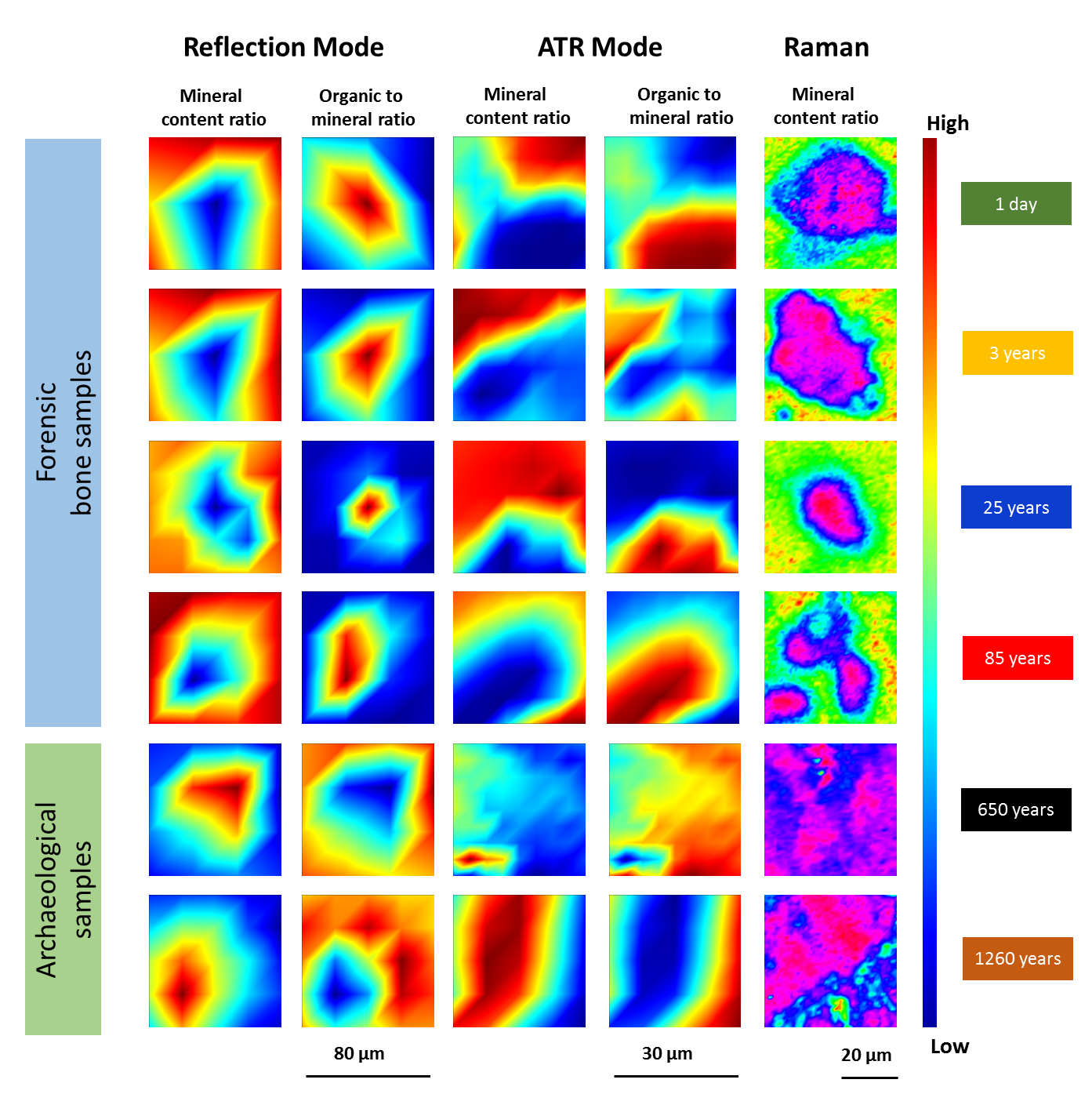

Supplement: S3 Fig — Mineral content ratio and organic to mineral ratio are displayed for reflection- and ATR- microscopic imaging measurements. For Raman microscopic imaging only the mineral content ratio was determined. (TIF) [file pone.0174552.s003.tif]
