# Supplementary material for: Assessing various Infrared (IR) microscopic imaging techniques for post-mortem interval evaluation of human skeletal remains
Source: PLoS One. 2017 Mar 23;12(3):e0174552. doi: 10.1371/journal.pone.0174552 (PMC5363948; doi:10.1371/journal.pone.0174552)
Supplement: S1 Table — (DOCX) [file pone.0174552.s004.docx]

| Grave | Sex | Molecular genetic sex typing |  |  |  |  |  |  |  |  |  |
| --- | --- | --- | --- | --- | --- | --- | --- | --- | --- | --- | --- |
|  | **morph.** | **Amelo short** | **SRY** | **PowerPlex® ESX 17** | **Amelo long** | **DXS7424** | **DXS8378** | **DXS6803** | **GATA172D05** | **PMI** | **Radiocarbon Dating** |
| 16 | m | X, Y | SRY | n.d. | X, Y | 16 | n.d. | n.d. | n.d. |  | 1030-1260 |
| 144 | m | n.d. | n.d. | n.d. | n.d. | n.d. | n.d. | n.d. | n.d. |  | 650-870 |
|  | m | n.d. | n.d. | n.d. | n.d. | n.d. | n.d. | n.d. | n.d. | 1 day | n.d. |
|  | m | n.d. | n.d. | X, Y | n.d. | n.d. | n.d. | n.d. | n.d. | 3 years | n.d. |
|  | m | n.d. | n.d. | X, Y | n.d. | n.d. | n.d. | n.d. | n.d. | 25 years | n.d. |
|  | m | n.d. | n.d. | X, Y | n.d. | n.d. | n.d. | n.d. | n.d. | 85 years | n.d. |

**Table S1** Summary of morphological and genetic sex-typing results.
