# Supplementary material for: Assessing various Infrared (IR) microscopic imaging techniques for post-mortem interval evaluation of human skeletal remains
Source: PLoS One. 2017 Mar 23;12(3):e0174552. doi: 10.1371/journal.pone.0174552 (PMC5363948; doi:10.1371/journal.pone.0174552)
Supplement: S1 File — (PDF) [file pone.0174552.s005.pdf]

Using ^{13}C-, ^{15}N- and ^{18}O stable isotope analysis of human bone tissue to identify transhumance, high altitude habitation and reconstruct palaeodiet for the early medieval Alpine population at Volders, Austria

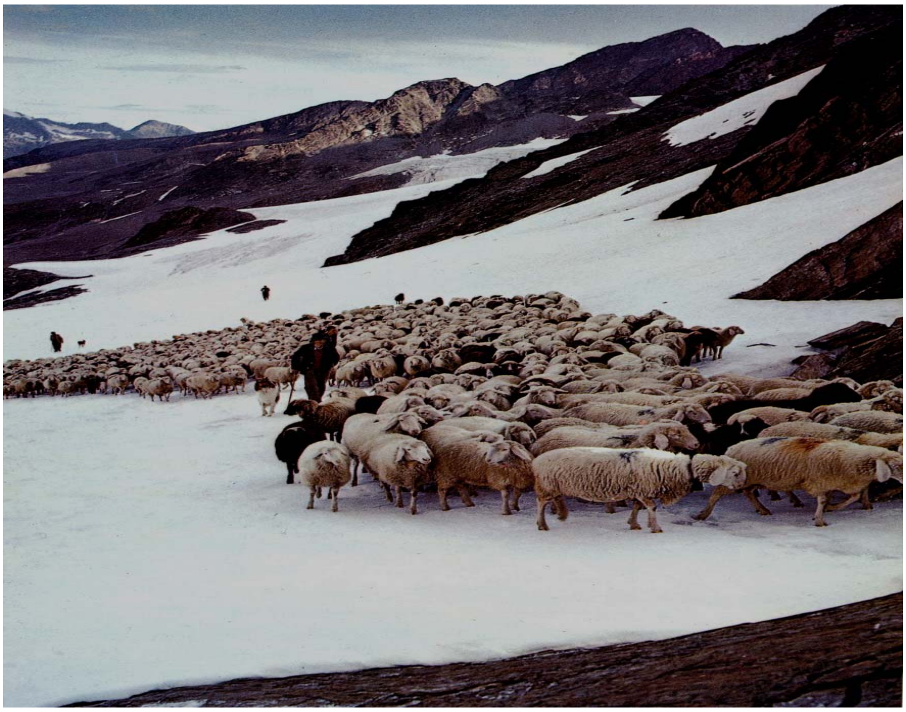

Dissertation zur Erlangung des Doktorgrades der Fakultät für Biologie der Ludwig-Maximilians-Universität München

Erstgutachter: Prof. Dr. Gisela Grupe
Zweitgutachter: Prof. Dr. Klaus Schönitzer

Eingereicht von George McGlynn München, Februar 2007
Tag der mündlichen Prüfung: 27. Juli, 2007

Time, which antiquates antiquities and hath an art to make dust of all things, hath yet spared these minor monuments

Sir Thomas Browne

Contents

1 Introduction 5
1.1 Brief history of Volders 7
1.2 Volders Topography 9
1.3 History of the Inn Valley 12
1.4 The Volders cemetery and past mortuary practices 13
1.5 High altitude habitation and habitat utilization 19
1.6 Transhumance at Volders 21

2 The Volders study 26
2.1 Oxygen isotopes, elevation, and people 26
2.2 Carbon and nitrogen isotopes and dietary reconstruction 29

3 Study objectives 31
3.1 Importance of skeletal studies for future studies 31

4 Bone structure and composition 34

5 Tooth structure and cementum analysis 37
5.1 Brief history of TCA 38
5.2 Cementum structure 39

6 Taphonomy at Volders 44

7 Literature Review-Stable isotope analysis in anthropology 58
7.1 Stable isotopes in palaeodietary reconstructions 58
7.2 Stable isotopes of carbon and nitrogen in collagen 60
7.3 Stable isotopes of carbon in structural carbonate 62
7.4 Carbon fractionation 63
7.5 Stable isotopes of nitrogen in bone collagen and nitrogen fractionation 68
7.6 Stable isotopes and diets based on aquatic resources 72
7.7 Stable isotopes of oxygen 75
7.8 Oxygen fractionation 77
7.9 Oxygen isotopes in archaeometry 85
7.10 Oxygen isotopes and migration 87

8 Methods and material 91
8.1 Morphological examination 91
8.2 Tooth cementum annulation (TCA) 92
8.3 Collagen extraction 95
8.4 Amino acid assay 97
8.5 Structural carbonate extraction 98
8.6 Mass spectrometry 98
8.7 Faunal samples 99

9 Results 100
9.1 Morphological examination 100
9.2 TCA age at death estimations (automated and manual) 108
9.3 Collagen integrity 122
9.4 Faunal collagen carbon and nitrogen stable isotopes ratios and carbonate carbon and oxygen stable isotopes ratios 124

9.5 Human collagen carbon and nitrogen stable isotopes ratios 129
9.6 Structural carbonate carbon and oxygen stable isotope ratios 137
9.7 Carbon spacing 150

10 Discussion 160
10.1 Palaeodemography 160
10.2 Tooth cementum annulation 165
10.3 Faunal analysis 169
10.4 Palaeodietary reconstruction 173
10.5 Identifying the utilization of high altitude areas 178

Summary 186
Appendix 1 (Volders Anthropological catalog) 189
References 205
Erklärung 243
Curriculum vitae 244
Acknowledgements 245

1. Introduction

The ability to adapt to diverse environments is a characteristic that ensured man and his ancestor's survivability over millions of years. The success of a species or group of organisms depends on the optimal utilization of the niche it occupies within a particular ecosystem. Adapting to a habitat requires strategies that afford the inhabitant with advantages that will increase its chances of survival. With cunning, skill, intuition and innovativeness, aided by tight knit social structure and group cooperation, humans have succeeded in colonizing virtually every imaginable terrestrial corner of the earth despite seemingly insurmountable habitat extremes, from torrid deserts to the unrelenting winter cold of the artic tundra. The climatic, topographic and ecological challenges presented by an alpine area also required substantial behavioral adaptations to overcome the natural barriers presented by this particular environment, ensuring the people success in a relatively challenging and potentially harsh environment.

To understand a past civilization, knowledge about its subsistence strategies and environmental surroundings is essential. The type of environment people are subjected to has a direct effect upon natural resource availability, agricultural pursuits, choice of animal husbandry, related work practices and subsequently food sources. To some extent, the foods we consume reflect our cultural adaptability. Today, humans subsist on diets, which span a huge range of possibilities, from those exceedingly rich in animal foods to those, which seem to be dangerously low in protein. As a result, diet patterns have wide spread consequences in regards to physical, mental and social development. Reconstructing dietary aspects of an early European culture provides theoretical and methodological means of framing the culture at a particular time in history and also the culture's change through time. Furthermore, determining subsistence practices and addressing the adaptation to an alpine environment will shed light on the current efforts towards understanding early alpine civilization, environment and biology.

The discovery of long forgotten cemeteries and burial grounds often happens inadvertently. These discoveries are frequently the result of large scale earth moving required by the increasing demand for new construction and to satisfy the material needs of an ever growing population. In the course of these various building projects, great quantities of earth are planed off or dug out, sometimes revealing ancient remains, hidden for many hundreds or thousands of years. Aged structures, utensils, textiles, ceramics and bones are often recovered in the process. It was precisely such a situation that led to the uncovering of state of Tirol's largest early Middle Age necropolis to date.

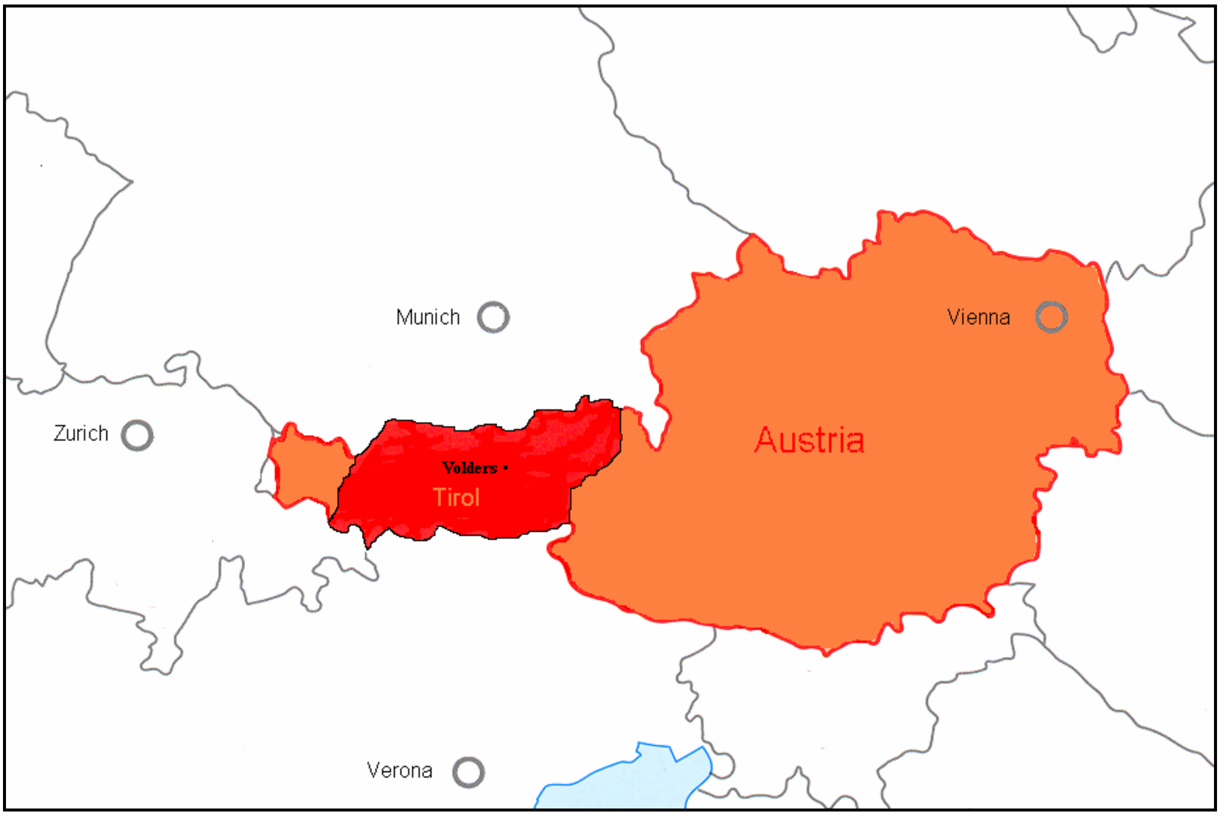

Figure 1.1. Map of Austria showing the state of Tirol and the location of Volders.

On October 29, 2001, while in the process of clearing an inconspicuous vacant lot in the Augasse 1, in the town of Volders, Austria (Fig.1.1), in preparation for the erection of an apartment complex, workers noticed that some unusual looking objects were unearthed. Once the objects were sighted, there was an immediate halt to further digging. The uncovered objects were carefully emptied upon a freshly cleared surface. Several large bones surfaced, freed of the earthen mantle in which they were wrapped. Initially however, it was not certain if the bones were of human or animal origin; however, after a recognizable human skull spilled out from the debris local officials were promptly notified, who in turn called on the expertise from local archeologists.

According to several eye-witnesses, they could recall bones turning up during the course of various past building projects in the immediate neighborhood (Zanesco & Stadler 2002). Unfortunately, the inclusion of these discoveries was never documented in the city's archives. Those doing the building are often under pressure to continue their project and also acutely aware that should they make such finds public, they risk a certain stoppage to construction.

This scenario is familiar and remains, even in spite of today's somewhat greater respect towards historical treasures, an all too frequent occurrence (Fig.1.2). Situations such as the

one encountered here, however, afford the rare opportunity to open history's door and look into the past. In the effort to accurately reconstruct history from such sites, the application of modern osteological and archaeometric methods have proven indispensable and provide the foundation for this examination.

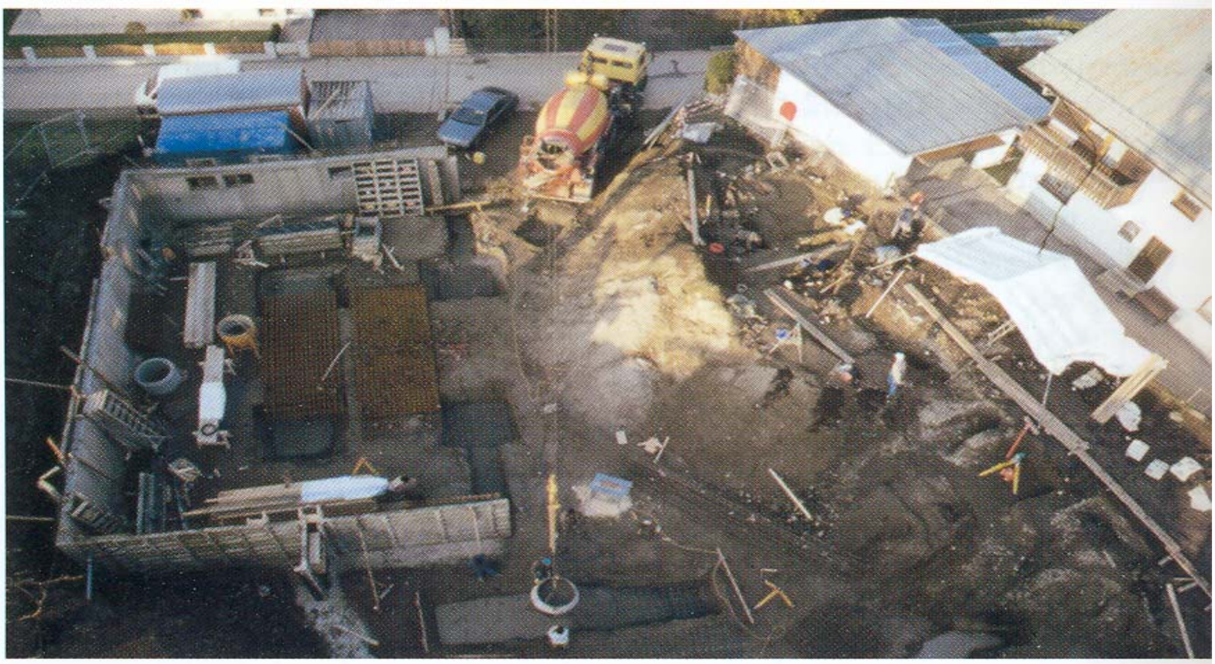

Figure 1.2. Volders cemetery in the Augasse. Aerial photograph taken from a building crane clearly showing the clash of two interests. To the right is a portion of the archaeological dig site with its characteristic tent, walking boards and theodolite, and on the left the construction of an apartment complex in full swing. The cemetery actually extends eastward under the existing garage, driveway and house (to the far right). Two years after the excavation was completed an addition on the other side of this house was built, naturally without notifying authorities, archaeologists or the office for conservation policy. Neighbors who witnessed the construction said that "bones were sticking out of the ground everywhere". This action basically typifies the usual callous stance taken by most and also the wonted lack of understanding with respect to archaeological history (Photo: A. Zanesco).

1.1 Brief history of Volders

The first mention of Volders (Fig. 1.3) in the historical records can be traced back to the year 995, where the name "Volares" appears on the books (Redlich 1886, Moser et al. 1984). However, the origins of settlement in this region dates back much further. Prehistorically, it was one of the largest settlements in the entire Inntal area (Noebl et al. 1960). This premise is based in part upon the discovery of a 2500 sq. meter cemetery containing 431 urn burials and the remains of over 800 individuals at the west end of Volders in 1955, found during the

course of digging a canal to facilitate the laying of a sewer pipe. The excavation team, led by Alfons Kasseroler, also documented four separate cremation pyres that were ^{14}C dated to 2620 BP placing them in the Urnenfelder period of the early Iron Age (Kasseroler 1959). In addition, the well known La-Tène period settlement of Himmelsreich (643m) dated to 2400 BP (approx. 400BC), located on a hill at the eastern border of Volders, as well as the early Iron Age "Johannisfeld" settlement discovered in 1976, are both a further testament to this assertion (Kasseroler 1956, Moser et al. 1984). Archeological finds recovered in the Lahnbach valley to the north of the village Fritzens, located on the other side of the river from Volders, as well as numerous other Hallstatt and La-Tène sites in various neighboring communities also point to the intensity with which this region was occupied. The people belonging to this prehistoric epoch would later be coined the Fritzens Culture. During the Roman era, Volders acted as an important station on the Roman thoroughfare connecting Italy with the north. Pottery sherds, coins, metal goods, roads, building structures and a diverse array of other artifacts indicate a multifaceted cultural mosaic of intense economic activity, religion, hand-crafts, complex social structure, transportation networks, trade, farming and land use.

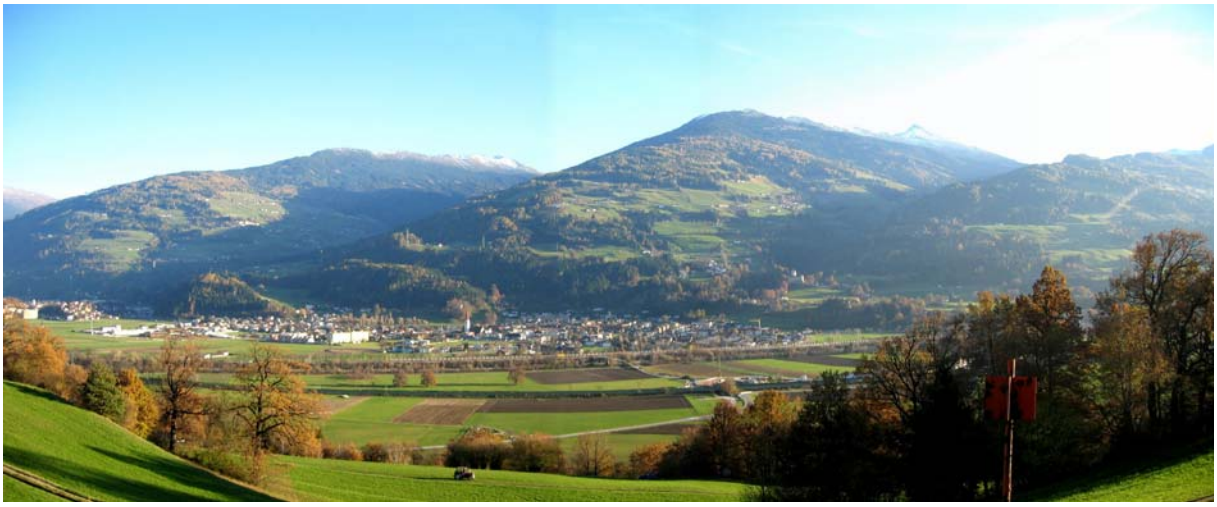

Figure 1.3. The present day town of Volders, situated at the base of Grossvolderberg, one of the mountains belonging to the Tuxer Alps, whose leading edge ends at the Inn Valley, today lush and cultivated, pictured in the foreground. (Photo: K. Wurzer. View from the village Baumkirchen, looking southward).

The Romanization of the area was so intense that its traces are still clearly detectable up until 8th century AD. Recent evidence even suggests that the Volders area itself may have been a

port stop on the Inn River during the Roman era when boats were used for transportation (Stadler, personal communication).

The origins of the inhabitants of Volders are said to be Raetish-Celtic (Gleirscher 1991), however, those living at the cross-roads to different cultures can seldom trace their inheritance to only one branch on the tall, wide tree of ascendancy. This pertains particularly to the early Middle Age, post-Roman Empire period in the Inn valley, a period in European history in which frequent migrations, active trading, and hence, heavy people traffic were commonplace.

1.2 Volders topography

The small village of Volders, Austria (557m) is an alpine community situated between the Inn River to the North and the steep, imposing Tuxer mountain range to the South (Fig. 1.4). According to Spindler (2002), the valley floors were covered by thick wetland forests and remained impenetrable until the late Middle Age when effective drainage activities, riverbank stabilization and extensive clear cutting made the land usable for agriculture and grazing. The unique topographical features surrounding Volders unquestionably provided the impetus for the adaptive response necessary to cope with the lack of arable flatland, an adaptation that resulted in making use of the mountain and foothill environment.

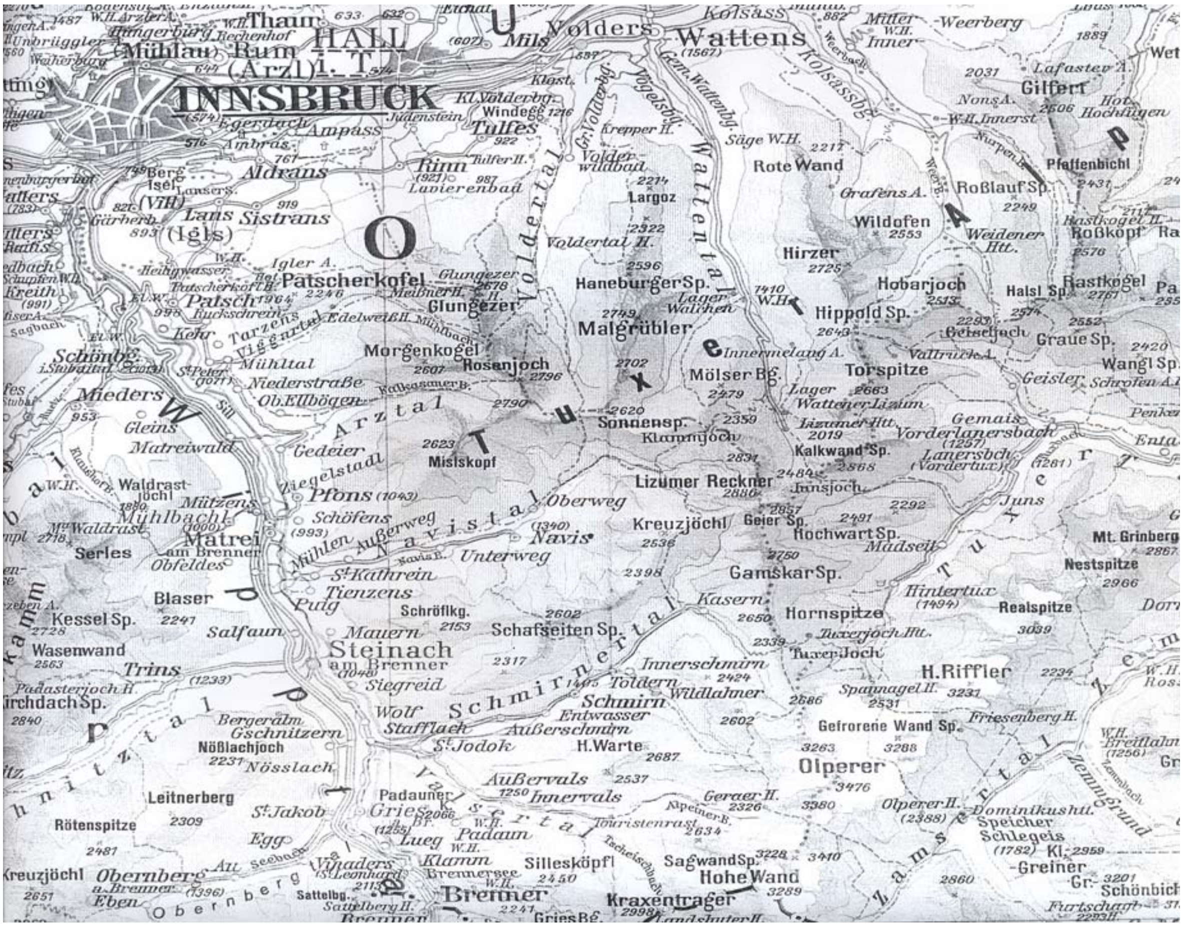

Figure 1.4. Topographical map of the area to the south of Volders, which is located at the top of the map near the center. Note the two long valleys of Voldertal and Wattental on either side of the alm areas used by the Volderer that stretch from the high Tuxer Alps and join the Inn Valley to the north. The Wipp Valley runs north to south between Innsbruck and Brenner and leads up to the important Brenner Pass connection to Italy at the bottom left of center.

In addition to the encumbering dense wetland forest, the Inn was prone to frequent and serious flooding, which made the planting of crops in the river's vicinity a risk ridden endeavor. In comparison to the lush, grassy mountain pastures, the valley floor on both sides of the Inn River consisted of swamps and moors (Fig. 1.5). In the event that a harvest was destroyed, the consequences would have been catastrophic for man and animal alike, since food crops designated for human consumption as well as feed crops for animals, vital for the winter months, would both be ruined. Heavy rains could also cause the Volderer Bach, a stream which flows down the mountain above Volders and various smaller streams such as the Veitsbachl, to swell and flood the lower lying areas. In 1908, exactly this happened, when the town suffered a floodwater catastrophe following a storm that destroyed houses, bridges, roads and submerged large parts of the town under a layer of mud and rubble. Although the

immediate vicinity surrounding the Volders town center was generally less susceptible to the hazards of rising water than others, the earlier inhabitants of the villages along the river obviously recognized this problem and chose to build their homes and plant their crops on the base of the foothills or slightly higher elevated locations at the valley floor, for example natural plateaus and mounts, to avoid a potential catastrophe.

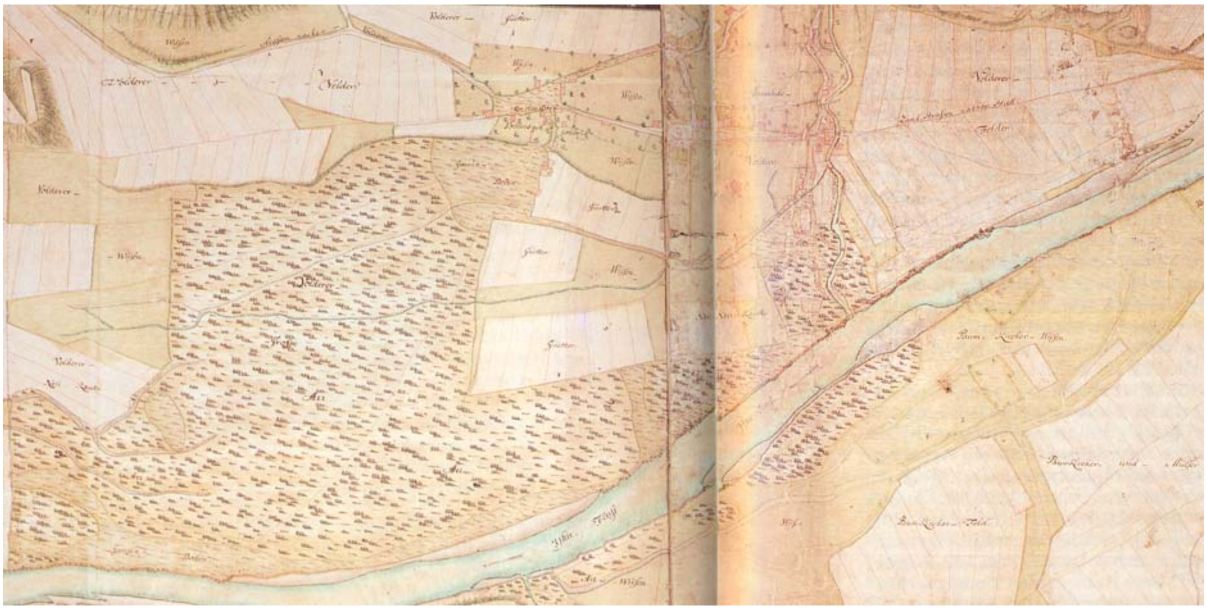

Figure 1.5. Archival map dating from 1746 that shows the extensive swampy, wet land area (Au) that still existed at this later point in time between Volders and the Inn River (In: Moser et al., 1984).

There is a host of evidence to suggest that earlier settlers of this area preferred to build their habitations at higher elevations because they were easier to defend against attackers. In times of brutal invasions this defensive tactic was vital for survival. Taken together, both are the primary motivation for the assumption made by local archaeologists that the earlier location of the village, which is still unknown, was above the present day village somewhere on the mountain incline.

The availability of land suitable for agriculture was therefore limited to the areas out of or elevated above the flood zone. More importantly, and significant for this particular study, is the fact that land required to graze herd animals was considerably reduced. Lack of sufficient space allowing for both the grazing of animals and agricultural activities led to the adaptive response mirrored in the pastoral methods employed by the early settlers of this area. That the inhabitants of this area should take advantage of the higher altitude terrain to graze their herds

and utilize the foothills to plant crops simply represents a common sense response to an, at that time, insurmountable obstacle.

The high mountain pastures or "alms", as they are called in the Alps, used by past and present day farmers of the village Volders for grazing animals and for harvesting grass, are located along the Volders valley (see "Voldertal" Fig. 1.4) belonging to the community of Grossvolderberg. These alms are located primarily on the west and north face of the Tuxer Mountains between Volders valley and Wattens valley. The Largoz Peak (2215m) along with its Largoz alm are the site of seasonal herd drives in the late spring and early fall months and of particular interest for this study.

1.3 History of the Inn Valley

The inhabitants of the Inn Valley and its smaller adjoining side valleys during the 1st century AD are described as Breunen, later as Breonen. These people were described by Strabo, as being related to the Raetians (Lippert 1989). The eastern Gothic King Theoderich noted the people's war-like character, a cultural characteristic that was apparently vital to their survival in the late Roman period. In the second half of the 3rd century AD, fighting between the Breons and the Alamannen intensified. The division of Raetia into two provinces under the control of Kaiser Diocletian and Constantine I alleviated tension sufficiently to subdue this problem. The state of Tirol and the portion of Bavaria bordering on the Alps, as they appear today, became a new province, known as Raetia Secunda. However, savage attacks in the year 357 AD into the Regensburg area from the ranks of the Juthungen, an eastern Alamannen people, resulted in the fleeing of the Roman inhabitants to fortified cities and Castells and a subsequent depopulation of the border area around the Danube river basin. Under orders from Kaiser Valentinian, a large Castell needed for military provisions was erected in the alpine area, the Veldidena (now Innsbruck-Wilten), and remained important until the 5th century, when it burned down. A second Castell, called Teriolis, was located to the west in Innsbruck's neighboring town of Zirl at Martinsbühel. Both of these military bases were absolutely necessary for infrastructure and support in patrolling the Roman thoroughfares. What impact the Alamannen incursions had upon the alpine region is not fully understood. In addition, the regional influence of the Franks on the Inn Valley during the second half of the 6th century from 545-590 AD, whose military invasions against Upper Italy led them through this particular area, is not known.

What is certain though, is that beginning with the 6th century, roughly at the start of the Middle Age, and the approximate time period to which the burials at Volders date, a new

culture was rapidly colonizing the region between the Alps and the Danube, including the Inn Valley area. These people, known as the Baiuwaren, colonized these areas as well as large parts of what is now the state of Tirol. The Baiuwaren were engaged militarily against the Slavs to the south. Due to their constant presence in this area, it followed that the alpine country in North Tirol gradually became settled. Later in the 8th century, the Baiuwaren colonization spread to the area known today as South Tirol, Italy. The upper Inn Valley, west of Innsbruck, was sparsely settled by the Roman cultures and was chosen by the Baiuwaren for their place of habitation. The lower Inn Valley was more densely populated, especially around Innsbruck and the Wipp Valley leading over the Brenner to Italy. These settlements were regularly spaced eastward down the Inn Valley and were primarily Roman. The Baiuwaren apparently respected the division and refrained from settling in this area. Archeological evidence from Pfaffenhofen in the upper Inn Valley indicates that the two cultures probably also lived side by side and mixed to some extent. A peaceful co-existence at the beginning of the Middle Age between the two groups can be assumed (Dopsch 1930) and is further supported by this author's own anthropological observations that show a surprisingly low incidence of violence induced trauma, however, the clear distinction eventually succumbed to a steady assimilation of the Roman people by the ever growing Germanic influx from the North.

Documentation and descriptions for the settlement patterns in the inner-alpine region following the collapse of the provincial Roman authority are remarkably scarce. Only with the advent of the 8th century AD, does the written documentation become more prolific. This makes the bioarchaeological examination of the so-called "Dark Ages" a more challenging and, historically speaking, necessary undertaking.

1.4 The Volders cemetery and past mortuary practices

As mentioned previously, a large segment of prehistory in the Volders area, specifically during the late Bronze Age and early Iron Age, exhibits cremation burials. Ash, bone fragments, and animal bones representing sacrificial offerings as well as lavish associations were placed in artistically embellished ceramic urns in carefully constructed stone encased graves. It is speculated that this form of mortuary practice, which was common for many European cultures at this time, continued for about 1500 years and probably represents a fundamental change in the perceptions surrounding death and religious beliefs, although it has also been suggested that eastern influences were responsible for the initiation of burning the dead (Konstam 2002).

The early Middle Age inhabitants of the Inn Valley lived in close proximity to newly Christianized Italy and were heavily influenced by Christian ideals and customs. Individuals belonging to the Christian faith were no longer cremated but interred, primarily in an east to west direction, with the head in the west, eyes facing eastwards. Grave associations tended to be sparse at this time.

How members of the two main Inntal cultures of the early medieval period were buried differed markedly. The Baiuwaren tended to inter their people with lavish grave goods. Men were buried with their warrior regalia, including weapons like swords, knives and helmets. Late Roman burials, influenced by early Christian beliefs, were characterized by the inclusion of only a few belongings such as clothing accessories, combs, hair needles and small pocket knives, or were completely lacking in any accessories what so ever. These later Roman burials were also often ringed with stones and not organized in rows (Martin-Kilcher 1993). This feature was witnessed in a number of burials at the Volders cemetery.

Most of the burials at Volders adhered to the Christian practice of laying the body stretched out on the back with hands folded over the waist or chest and occasionally at the sides (see Fig. 1.6). The majority were also found to be in the usual east to west orientation, 23 were discovered to be in a north to south direction with the head at the north end (Fig. 1.7). Only nine of the burials were documented with grave associations. A small number of skeletons were stratigraphically very close to one another without signs of disarticulation, as such, a multiple interment is presumed. There was no indication for collective trauma amongst these persons, as would be expected for battle casualties, however, it is possible that these individuals died of a rapidly lethal contagion. The vertical profile of the Volders cemetery depicts several separate layers of burials that suggest the cemetery may have been used during different chronological periods (see Fig. 1.8). The exact stratigraphic blueprint was not completed at the time of this study, nor were ^{14}C dates available to specifically distinguish the individual burial layers from one another. It is therefore possible that at least some of the skeletons derive from a later or earlier time period. Due to the mixture of different directionally oriented burials, the presence of stone encirclements around some of the graves and accessories for dress (iron belt buckles with silver inlay, knives, metal belt strap ends and combs) typical for late Roman and early medieval period, the majority of burials are believed to be from the late 6th to early 7th century. According to the archaeologist Professor Harald Stadler at the University of Innsbruck, all of the grave associations recovered at Volders are characteristic for the Langobards, a West German people that migrated southward in the 6th century and settled in the Po basin.

In the area surrounding the cemetery, fragments of simple ceramic, Terra sigillata and Lavez dating from the late Iron Age to the early Roman period were recovered. A number of bronze Roman coins dating from the 4th century AD were also discovered. These artifact finds probably indicate an earlier secular use, perhaps a settlement, trade post, or as mentioned earlier, a port on the Inn River during the Roman period.
According to a topographical survey and aerial photographs, the cemetery is located directly on the upper rim of the approximately 4 meter high ancient Inn riverbank (Zanesco & Stadler 2003). Formed by the river's natural meander, the buttress-like riverbank is actually the forward edge of a low plateau, carved away by thousands of years of hydraulic activity, and stretches from Volders several kilometers to the east through the neighboring town of Wattens. The geological characteristics of the area reflect the combined influence the Inn River's movement and proneness to flooding, as well as the erosion caused by the Volderer Bach, a large stream created by runoff from the mountains to the South that flows directly through the town. Gravel and sand, intermingled or layered, derived from these water ways, have left their footprints in the geology of the area.
Individuals living at higher altitudes on the mountains that may have died naturally or perished in an accident, appear to have been brought back down to the valley for burial. This presumption is based on the lack of historical and archaeological evidence substantiating intentional interment on the mountain. Cemetery finds in the valley are therefore probably representative of individuals living at altitude as well as the valley lowlands.

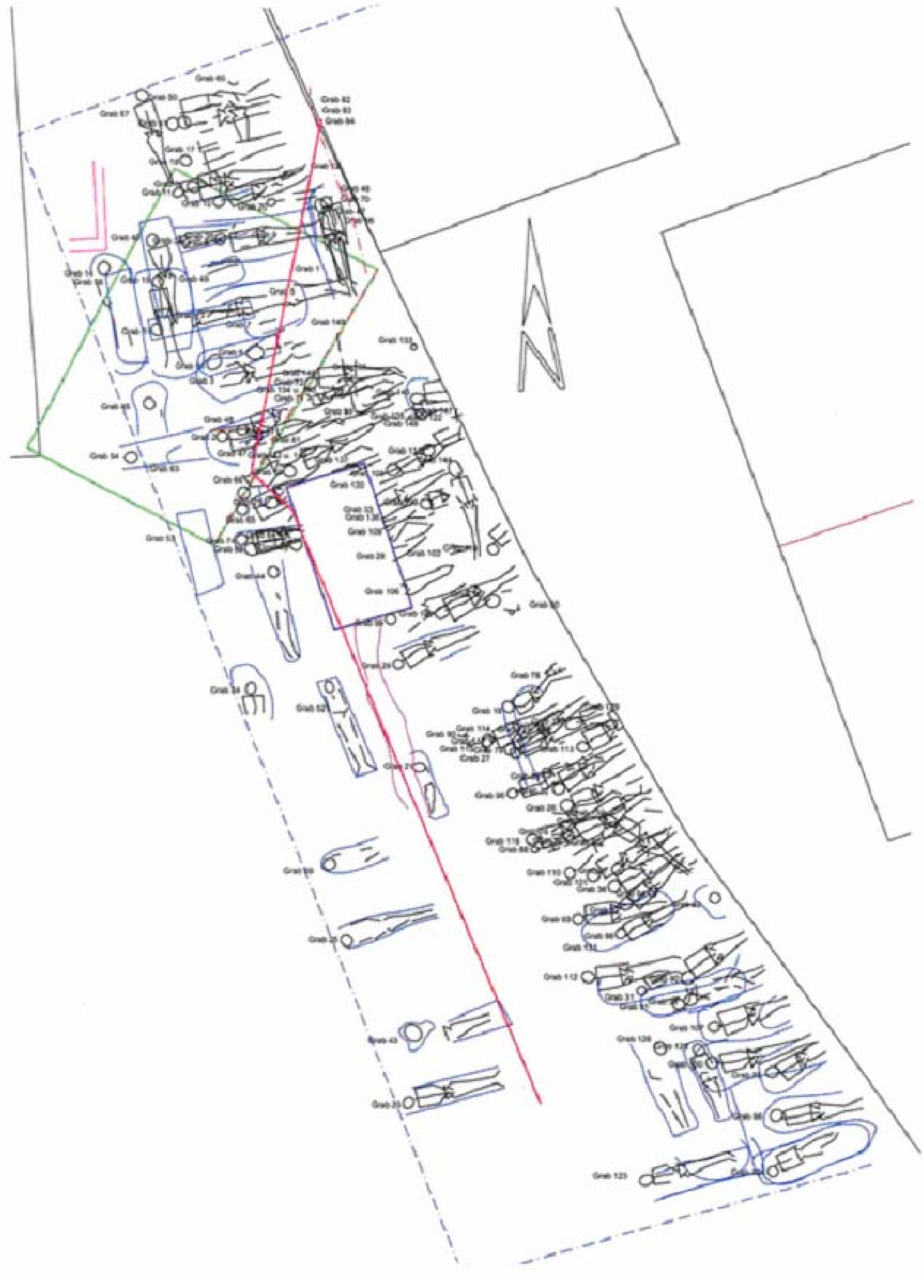

Figure 1.6. Volders cemetery plan showing layout of the skeletons, grave borders (blue solid), cemetery boundary line (blue checkered), house structure edges (right half of diagram), lime pit (violet rectangle at center), area dug out by backhoe (green rectangle), boundary of new apartment (red), and retaining wall (pink). (Excavation plans in Figures 1.6-1.8 courtesy of Alexander Zanesco, City Archaeology Hall i. T.).

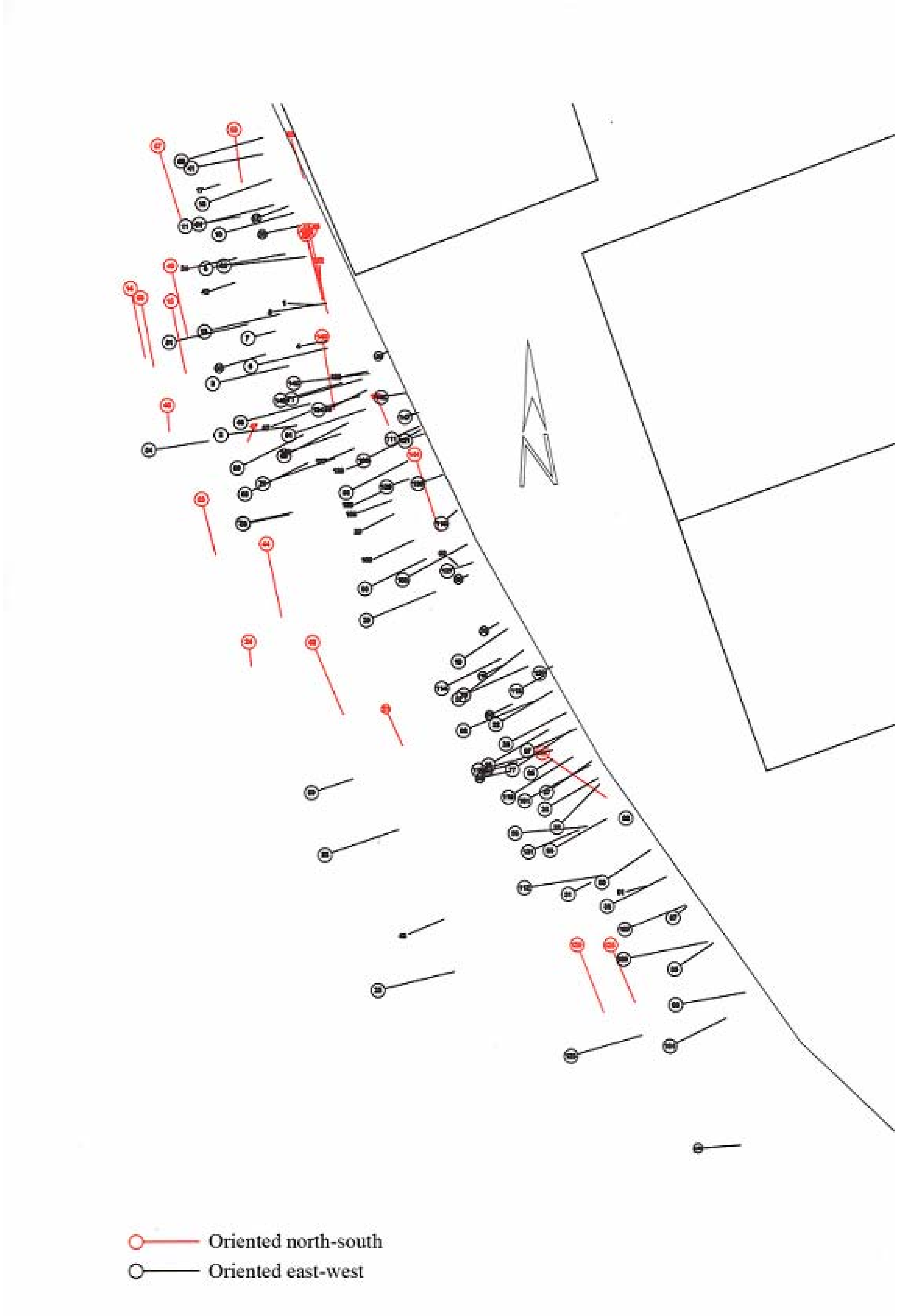

Figure 1.7. Simplified burial plan indicating the grave orientation. Burial in red are oriented in a north to south direction with the head to the north and those in black are oriented east to west with the head to the east.

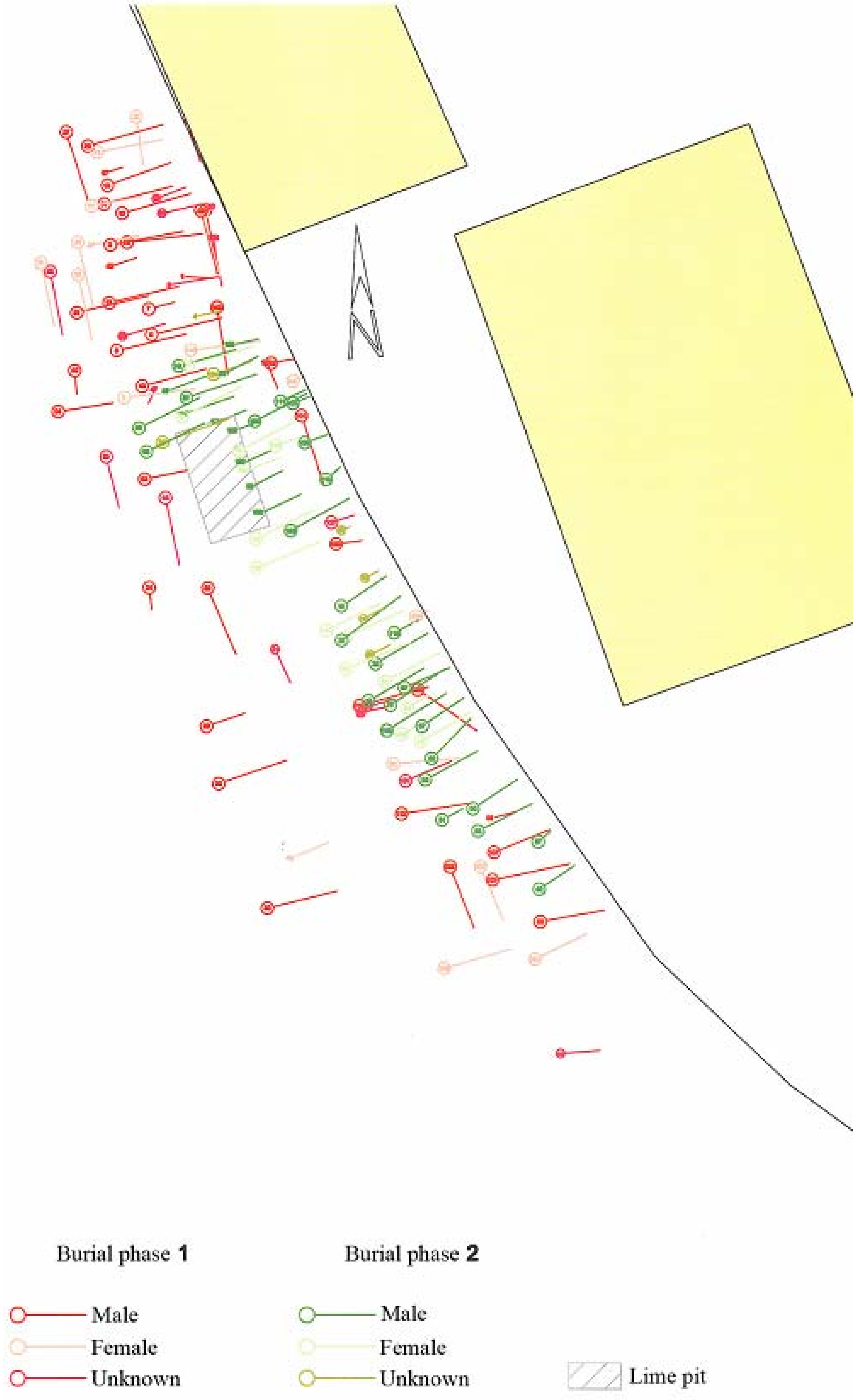

Figure 1.8. Simplified cemetery plan indicating the two main phases of burial, the first beginning in the 5th century AD and the second during the 7th-8th century AD, showing the location of male and female burials.

1.5 High altitude habitation and habitat utilization

At the heart of the Volders skeletal series study is whether the higher elevations were indeed used for living or working by these particular individuals. Neither archaeological evidence for a contemporaneous settlement or written documentation exist, which might otherwise provide indices for this, nor do the grave associations recovered at Volders lend any clues to ascertain subsistence strategies or work activities. The aforementioned background on Volders circumstantially suggests this, but definitive, empirical proof is lacking. The Volders area is no stranger to high altitude sites. The Himmelsreich site mentioned earlier, subject to numerous archeological excavations and inquiry over the last four decades, was a fortified, prehistoric settlement at the eastern border between Volders and the neighboring village of Wattens. Sites for ritual practices, abandoned mines where once copper, iron, quartz and silver were sought, and high elevation alms for animal grazing during the summer months are all known to have existed and been used prehistorically and historically in this vicinity. As early as the Mesolithic period, quartz was mined as raw material for tools at the Tuxerjoch, a mountain in the Tuxer Alps not far from Volders. The quarry is situated at 2,800m, making it the highest presently documented mine in Austria (Leitner 2001). Another prominent example is the “Zillertal Window” at 2,700m, where rock crystal was gathered during the Stone Age. Evidence for locations frequented for the purpose of ritual practices such as the La-Tène period site at Erpensteiner Hof (802m) in the district of Volders called Grossvolderberg and also the mining activities during the Bronze Age in the surrounding mountain range are sufficient evidence to infer that at least some of the area’s people inhabited the higher elevations for a greater duration well before the Middle Age.

Archeological evidence with regard to settlement placement exhibits a distinct trend, showing that settlements in the Inn Valley were located primarily at the mountain edges of the valley floor or the foothills. The Kalvarienbergl near Imst, the Martinbühel in Zirl and the Kirchhügel in Ampass were all situated in the vicinity of main trade traffic crossroads or thoroughfares, and interestingly, all elevated with respect to the Inn river. This is important with respect to the Volders study, in that the settlement, as previously suggested was probably also located at the foot of the mountain Grossvolderberg.

Various small villages, hamlets (called Weiler in Austria) and their associated pastures, which are located at higher elevations, have retained their original Raetish names, definitive, linguistic proof that the alms, absolutely necessary for the grazing of livestock, were in use much earlier in the history of this particular region than just the Middle Ages. Recent

evidence from a 2005 archeological dig focusing on a wooden structure located at a high altitude alm in Obergurgel, Tirol, dating from the late Mesolithic period approximately 8,000 BC, indicates a regular usage of the area perhaps for hunting or as a base along an early trade or travel route (Zanesco 2005).

Effects of high elevation on humans

People living and working at higher altitudes are subjected to different physical conditions compared with their low altitude or sea level counterparts. To better understand what influence high altitude exerts upon the human organism's physiology, which in part influences aspects of the archaeometric examinations conducted in this study, a short clarification is necessary. The effects of physical variables such as thermodynamics and partial pressure are associated not only with changes in isotopic compositions (discussed later) but also have a direct affect on the human physiology and physic. High altitude in occupation leads to physiological and physical changes in humans subjected to this environmental parameter. An increase in elevation results in a reduction of oxygen available for respiration. Hypoxia implies reduced oxygen in ambient air and reduced physiologically available oxygen compared with sea level.

True hypobaric hypoxia, which is usually taken to be 2500m or higher, is the most important biological stress with which populations living at high altitudes must cope, and results whenever physiological, pathological, or environmental conditions cannot deliver an adequate supply of oxygen to the tissues (Moran 2000). High altitude populations are characterized on the basis of their adaptability primarily to hypoxic stress due to exposure to low partial pressure of oxygen relative to sea-level values such as populations in the Himalayas, Tibet Plateau, Andes, high plains Ethiopia (Ulijaszek et al. 1998).

According to Beall et al. (2000), oxygen transport includes the processes of acquiring, carrying, delivering, and using oxygen. The respiratory system acquires oxygen by moving air into the alveoli of the lungs, where oxygen diffuses into the blood and combines with the hemoglobin of red blood cells. Oxygen diffuses from red blood cells in the capillaries into the surrounding tissues, where cells metabolize oxygen for energy. Oxygen carrying in the blood is a function of hemoglobin concentration and O_2 saturation. Oxygen carrying is often enhanced at higher altitudes by an increase in hemoglobin concentrations over those observed at sea level.

Homeostatic changes in oxygen transport processes that result from increasing altitude are collectively known as acclimatization and result in acute physiological adjustments. These are processes whose initiation begins immediately following exposure to higher elevations.
BMR (basal metabolic rate), which is the minimum expenditure for maintenance of respiration, circulation, body temperature and other vegetative functions, is elevated, partly due to the increase in breathing. Metabolic rate is decreased on the other hand.
There are apparently also genetic predispositions that control the body's capacity to transport oxygen, and it is not clear at this moment if the existence of different traits influences the level of oxygen isotopes seen in bone collagen.
Increase in total lung capacity, chest size, polycythemia (increase in red blood cells), amounts of hemoglobin, a decrease in blood plasma, enlargement of the capillary bed, stimulated bone marrow growth, chemical-enzymatic changes related to internal respiration that enhance oxygen utilization at the tissue level, as well as other measures of pulmonary function such as increases in the depth and rate of breathing and an increase in heart rate are seen with increasing altitude. Hypoxia is not the only stress at high elevations, also the cold and poor nutritional availability have an influence on body size and growth. Mean birth weight and fetal growth are negatively correlated with altitude. Increased altitude also results in a greater concentration of ultraviolet radiation, because the air is thinner and offers less protection. Rapid heat loss causing cold stress, and higher winds and low humidity bring about dry conditions (Relethford 1999). According to Moran (2000), all of these modifications occur in individuals that are continually exposed to hypoxic conditions. Acclimatization modifications will, however, also be seen in individuals visiting higher altitudes periodically or for single, brief periods.
Certainly all of the factors mentioned associated with higher elevations combine to exert significant pressure on the human organism, requiring both physiological and cultural adaptive responses. Cultural adaptations cannot increase the availability of oxygen but they can help to cope with other stresses such as the cold, hunger and dehydration (clothing, shelter, scheduling activities during warmer day periods, fluid hydration, food provisions) (Relethford 1999).

1.6 Transhumance at Volders

The driving of herds, especially sheep, to the alm pastures located high up in the Volders mountains during the spring and summer months was no novel idea in the early Middle Age. Evidence for this activity, also known as transhumance, in the Alps is abundant and there is

good indication that this practical method of animal husbandry existed in Europe thousands of years back into prehistory (Wernicke 1989), while others assert that definitive evidence for its existence can only be documented for the Roman period onwards, although the practice truly reached its peak in the Middle Ages. The term transhumance derives from the Latin words “trans” and “humus”, and translates roughly to mean “passage across the earth”. There are different forms of true transhumance. Movement from lowlands to high mountain areas is referred to as *t. normale*; movement from high mountain pastures to lowlands is called *t. inverse*; and movement from an area between winter and summer grazing areas which is known as *t. mixte* (Jäger 1997).

The high alpine environment is an isolated, rugged and beautiful landscape, but at the same time hazardous, unforgiving to the careless and the weather is always unpredictable. In some of the more highly situated valleys like the Lechtal in Tirol the winter and spring months together last over 6 months and at the Lech alm areas even longer, although some who live there today maintain that the winter reigns year round. Life in the high alpine region is made difficult by these conditions and underlines just how important adaptive measures were to cope with this special environment. The fact that parts of the Alps remain virtually untouched by humans is a testament to their inhospitable nature.

Commonplace for this particular alpine area, the inhabitants of Volders were engaged primarily in animal husbandry and some agriculture. The practice of bringing cows, sheep and goats to the high elevation alms had additional motivations other than simply adjusting to problems with space at the valley floor. The pristine condition of the alm environment, its isolation from the settlement, and the bountiful grasses and shrubs found there, drew the attention of shepherds dating back to the Stone Age (Wernicke 1989). At higher altitudes the snow melt is delayed as is the subsequent growth of vegetation. During the summer months when vegetation on the valley floor periphery is drying out and regenerating only at a slow pace, succulent plants and grasses on the mountain slopes as well as a continuous supply of fresh melt water rushing from the mountain are available. The alms provide up to 4 months of sustenance for the animals.

In addition to providing pastures for herds, the alms were abundant sources of tall grass, which was cut, dried and transported to the lower valley areas for use as feed and stall straw for the late fall and winter months. Animal husbandry was an extremely important feature for the alpine economy. According to Wernicke (1989) transhumance has several advantages. For example, it provides and nurtures a subsistence object, and part of the family group can remain at the settlement and engage in agricultural or non-subsistence activities while others

move with the animals. Sheep, goats, cattle, swine and fowl were the mainstay and provided the majority of products animals have to offer. Not only was meat an excellent and vital source of protein and fat, but also products such as milk, cheese and eggs were a welcome nutritional resource. Apart from the nutritional aspect that animals afforded people, arduous tasks such as plowing fields, uprooting trees to clear pastures, pulling wagons and personal transportation to name only a few, were made more efficient or even possible through the employment of powerful draught animals such as oxen and horses. Sheep were a valuable asset with regards to wool production, which was used in the manufacturing of an array of textiles. It is important to note that remains of all of these animals were recovered within the confines of the cemetery during the excavation and according to their stratigraphic placement, are contemporaneous.

Throughout history the exploitation of animals in order to survive or to ease the burden of various hardships imposed upon man by nature, has been a key motivation for their continued relationship. A utilitarian partnership spanning over thousands of years back to the Neolithic period, often evolving into a form of symbiosis, make man’s dependence on animals for his existence clear (Bökönyi 1974). Animal domestication, animal husbandry, work animals, even household pets, are all synonymous with the human occupation and have long been an integral part of human life and have no doubt played an influential role in the cultural evolution of modern man (Cyrulnik et al. 2003).

The specific activities identified under the rubric transhumance vary slightly and appears to depend on the source. According to Jäger (1997), it is described as a form of mobile pastoralism in which the seasonal movement of herds proceeds in the accompaniment of a shepherd who is employed by the animal’s owner. Relatively long distances are traveled, lasting between one day and three weeks or longer to at least two different grazing areas within the span of one year.

Distinct differences exist between transhumance and other forms of seasonally influenced animal husbandry. People living in the Alps were forced to adapt to their unique surroundings and developed a specialized form of alpine farming called “Almenwirtschaft”, translated this term means “alm economy or alm pastoralism”. Long cold winters and a short growing season hampered early efforts to settle the alpine environment. The seasonal utilization of the vertical structure of the Alps to graze animals during the spring and summer months is ancient, yet, still a preferred pastoral method today. Its beginnings reach back to about 4000 BC, perhaps even earlier, and is known to have existed in the alpine regions bordering on the Mediterranean areas (Schenk & Eichfeld 2006). The prehistoric practice of farmers in the

Near East and Mediterranean regions utilizing the different vertical clines of their ecosystem certainly did not arise as a result of their own intuition. Early farmers no doubt observed wild animals, conditioned by the seasons of the year, grazing the lowlands in the winter months, the foothills during spring and autumn and the mountain alms in the hot, dry summer months in an effort to take advantage of the best environmental conditions available to survive.

The movement and distances involved in transhumance in the Mediterranean area were much greater than those observed in the alpine valleys such as those around Volders; nonetheless, there is a significant degree of vertical movement and thorough use of the environment that ensured the optimal growth and health of the herd. The steep, abrupt assent required to reach the more highly situated alms demanded a good physical condition from both man and animal. In mountain pastoral economies, children are often assigned to the actual tasks of herding, which reduce the caloric requirements of the household (Thomas 1976).

Based upon observations and interviews with modern day shepherds who still practice transhumance, for example, in the Pyrenees, the work they have is arduous and involves a considerable amount of responsibility. Four to five months of work, with the day beginning before sunrise and continuing until late at night. Care must be taken to ensure the animals are healthy and that injuries are treated. Herds must be driven and moved to prevent destruction of the fragile ecosystem through over-grazing. Sheep require 2-3 liters of water per day, and if water is scarce higher up then the herd needs to be driven down to lower altitudes to drink.

According to Štih (1998), the actual settling of the eastern Alps, for example, only truly began around the 7th century, and was accompanied by the "new" inhabitants continued practice of animal husbandry and Almenwirtschaft as the primary mode of subsistence. Only with the advent of the 13th and 14th centuries did agrarian colonization become more established in the Alps.

The rapid increase in population in the alpine areas, particularly during the 6th and 7th centuries, eventually led to problems with the availability of land at lower elevations and later acted as an additional motivation for intensifying mountain pasture utilization (Meyer 1998). Even today in the narrower valleys that pan out into the larger Inn valley, which are often characterized by very steep faces of up to 60°, such as the Ötztal, every available patch of grass is harvested by hardy mountain men and women who engage in a sort of balancing act on the sheer inclines as they stand sure-footedly, sometimes wearing crampons, and brace themselves against the swing of their scythes or tote heavy hay bundles by hand to lower grass depots. Throughout the early Middle Age, the practice of clear cutting large portions of forest was also spawned from an increasing demand for open space to build houses, plant

crops, create fields for planting the grass needed as straw for stalls and feeding herd animals, and of course for wood as construction and heating material. The traces of this practice are especially visible in the Inn valley around Hall in Tirol a neighboring town of Volders, formally called Solbadhall, a major salt mining works in Austria, where tens of thousands of timbers were needed to shore up the extensive labyrinth of mine shafts and burned in the fires needed for the evaporation process in salt production (Hocquet 1995).

2. The Volders study

In order to properly understand a people it is vital to delve into the history of their origins and explore their roots and past aspects of their culture. It would be insufficient to depend solely upon osteological data taken from archaeological skeletal finds, which are certainly necessary and provide information and details that history cannot, but these data should be seen as part of a multi-discipline effort towards gaining a better understanding of the people. All too often bones are viewed out of context and only as inanimate objects, measurements are made and conclusions to sex, age, cranial type and height are entered into a catalogue. The information accumulated from bioarchaeological studies must be combined with those stemming from cultural anthropology, archaeology and history if the creation of a more accurate and comprehensive picture of the past socio-cultural and palaeodemographic situation is to be made possible.

Because traditional morphologically based osteological methods are somewhat limited with respect to the types of information they provide on archaeological skeletal material, a variety of archaeometric methods are employed to expand the pallet of this extractable information. These novel and sophisticated techniques are generally associated with geophysics or forensic medicine and yet have established a sturdy foothold in the field of bioarchaeology. Microscopic and chemical components of bone and teeth are analyzed to elucidate various facets of past human existence such as nutrition and subsistence patterns, weaning age, migration, elevation of occupation, and state of preservation of calcified tissues.

2.1 Oxygen isotopes, elevation, and people

The alpine topography of Austria is many facetted and characterized by a myriad of different mountain shapes, rock types, meadows, streams, lakes, valleys and glaciers. The European Alps themselves are relatively young by earth standards, and were created by tectonic plate activity in the Upper Cretaceous and sculpted by the powerful fingers of glacier ice movement. According to Relethford (1999), mountainous regions have complex distributions of biotic communities. Three major ecological features are relevant to human habitation: vertical biotic zonation, irregular biotic distribution, and geological features such as slope and rugged terrain. With increased elevation, rapid changes take place in vegetation and animal life, commonly distributed in distinctive zones or biomes. A biome represents a segment of the biosphere, and is a small and more homogenous biogeographical region. On a given mountain it is possible to have four or five major biomes and numerous ecotones, or transitional zones. Geological features such as angle to the sunlight and valleys lead to the

creation of microhabitats. Ruggedness and inadequate amounts of soil can make some slopes useless for both herding and crop cultivation. These features are also visible at the alpine ecosystem of Volders, which meant that the inhabitants engaged in high elevation activities like pastoralism could not simply make use of an already existing paradise-like mountain environment, but had to shape it and adapt it to their own needs. Pastures had to be cleared of stones (Fig. 2.1), paths leading through the forest cut, trees felled, drinking holes formed and shelters built.

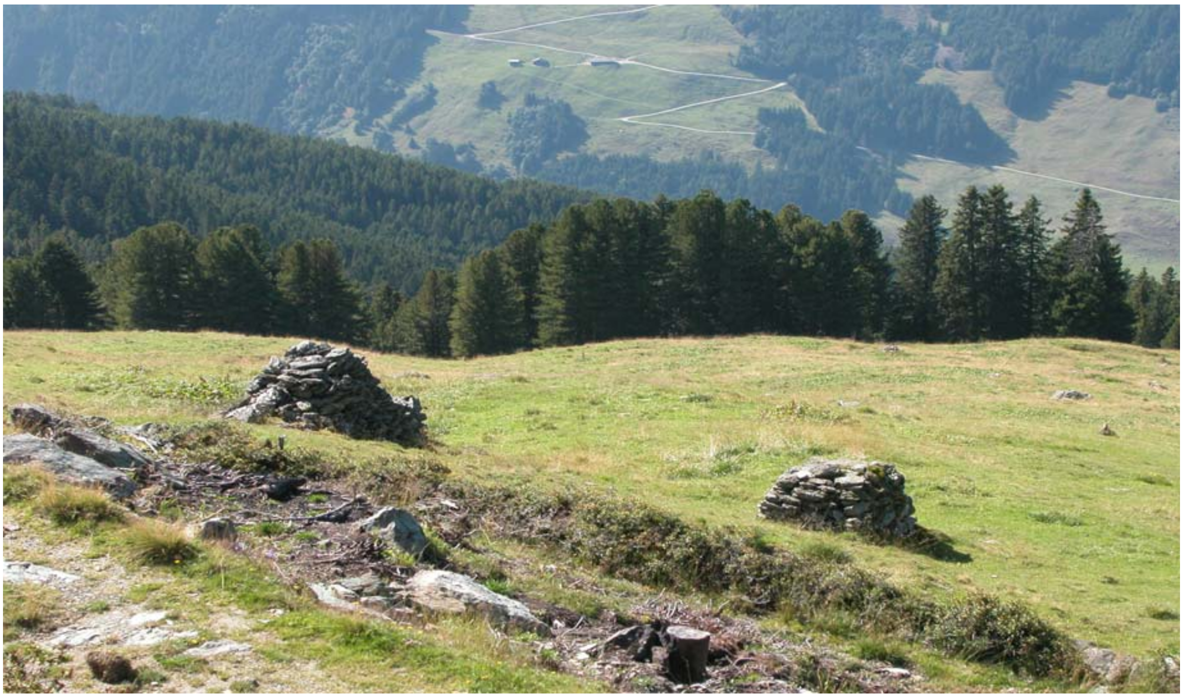

Figure 2.1. The clearing of stones from the alms above tree line at Volders function to increase grass yield and is illustrated in part by these man-made formations affectionately referred to as “Steinmandln”, translated to mean “stone men”. In the Alps these structures are used for orientation and are especially helpful to shepherds and travelers in winter when snow covers the landscape and obscures natural landmarks and paths. However, when they appear in larger groups they also serve the practical purpose of piling up unwanted stones removed from the pasture (photo: G. McGlynn).

The Volders site is unique, because the individuals living there were confronted with different environmental and cultural extremes. The settlement is thought to be at the lower elevated foot of the Grossvolderberg Mountain and yet the people were probably farmers practicing transhumance, using alms to graze their herds (Fig. 2.2), mining and cutting trees, activities that can all potentially take place at higher elevations. The majority of people are assumed to be rural peasants, but medieval Volders was located directly adjacent to a former Roman

thoroughfare station very near to the main passage through the Alps connecting Italy with the north, the Brenner Pass. The likelihood that some of the members of the community were involved in trade, transport of goods, administrative work, specialized handcrafts or clerical endeavors is therefore increased. The possibility that some of the individuals represented in the cemetery complex are migrants from the lowlands to the south must also be entertained.

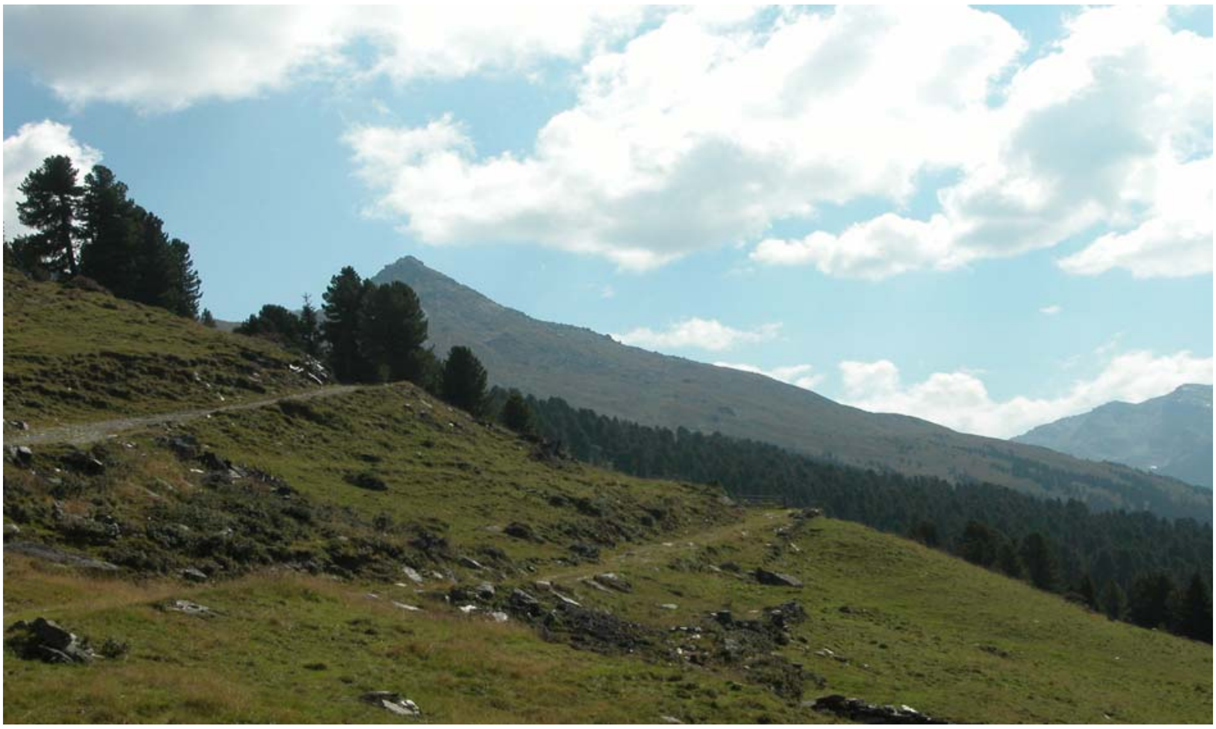

Figure 2.2. Volders alm area at tree line at about 2,000 m (photo: G. McGlynn).

The fact that the numerous alms needed for pastoral activities as well as the mining areas are all situated at elevations significantly higher than the valley floor, and that either periodic, seasonal or year round habitation existed, offers the fascinating and unique opportunity to readily identify the individuals who were engaged in activities in these areas and represents a primary focus of this study. This is made possible by examining the stable oxygen isotope signatures ($\delta^{18}O$) acquired in bone apatite samples taken from the Volders skeletal material. Oxygen isotopes have proven useful in the recent past for determining place of origin and provide researchers with the ability to reconstruct migratory patterns (Budd et al. 2004, Bentley et al. 2004, Millard et al. 2004, White et al. 1998). The pedestrian movement encountered in this situation is not horizontal as seen in migration, but vertical, and can span numerous biomes within a very short range. This study introduces an absolutely new and

innovative aspect to this particular aspect of the field of archaeometry. To this author's knowledge, it is the first time oxygen isotopes have been used to determine the utilization of high altitude sites as part of a subsistence strategy within a population based study and simultaneously identify the individuals within the complex who were engaged in these activities. Answering questions regarding mode of lifestyle, economy and place of physical occupation with this tool are made possible by the linear changes in oxygen isotope concentrations that accompany changes in altitude. This physical attribute of oxygen combined with the extreme variations in elevation within a very close proximity to where the people of Volders lived and worked, provide an optimal backdrop for conducting the study (see section 7.7 for details).

The fact that the inhabitants of Volders were basically forced to utilize the mountain environment because of topographical barriers, suggests that significant differences in the oxygen isotopes signatures can be expected. The tradition of sending juvenile boys and girls to work as shepherds on high-mountain pastures, a socio-economic strategy documented for other populations will also be investigated.

2.2 Carbon and nitrogen isotopes and dietary reconstruction

A second aspect to the Volders study concerns the reconstruction of palaeodiet using the established method of analyzing the stable isotope values of nitrogen ($\delta^{15}N$) and carbon ($\delta^{13}C$) in bone to give clues to the nutritional orientation of the population. The information provided by examining these isotopes, which are contained in bone collagen and obtained by acid extraction, yield a plethora of information pertaining to diet, dietary emphasis (consumption of C_3 or C_4 plant foods), degree of carnivory, and utilization of aquatic resources (Ambrose 1993). Potential inter-group differences resulting from sex or habitation at higher elevations will also be determined. Important to note, especially with respect to this particular survey, is the ability afforded by this method to identify individuals who may have had access to more nutritious or protein rich foods than others in the group, and in this way discern those bestowed with a higher degree of status or those who had more ready access to these foods than the remaining population such as peasant farmers. Certain individuals displayed a significantly larger body size, several had grave goods, and still others had skeletal variations thought to be heritable, perhaps indicating kindred. The isotopes of these people need to be especially scrutinized. Establishing whether or not imported marine fish or freshwater fish from the nearby Inn River were consumed by observing the $\delta^{13}C$ signatures in bone collagen is a further goal of this particular section devoted to diet. In addition, the $\delta^{15}N$ values for

skeletons belonging to infants will be examined to determine weaning practices, an analysis based upon the trophic level changes in $\delta^{15}N$ values observed between weaning and non-weaning infants (see section 7).

Relatively little is known about the early medieval Inn Valley inhabitants due to a lack of written documentation and the general scarcity of burial associations characterizing this period. The information gap primarily encompasses the provincial portion of the society, and is believed to result from widespread illiteracy. Mainly aspects related to the higher echelon of socio-economic society were preserved for future generations in written documentation, literature and art. This makes acquisition of information from skeletal remains from precisely these rural situations all the more pertinent.

An anthropological catalog documenting the osteological results, grave goods recovered and commentary to the burial situation is presented. Demographic details, pathologies and dental health among others are described and comprise a portion of the foundation necessary for this research.

The Volders study stands to make a valuable contribution to archaeometry and the field of anthropology. The use of stable isotope analysis as an archaeometric tool for palaeodietary and palaeoecological reconstructions have established their efficaciousness over the past 30 years, however, refinements and innovative usages remain in progress. This study aspires to add another useful feature to the archaeometric toolbox and provide a comprehensive survey of an early Middle Age people based upon a combination of morphological, archaeometric and historical data, at the same time shedding some light on the Dark Age.

3. Study objectives

To conduct an osteological analysis of the individuals represented in the archeological remains including age at death, height, sex and diagnosis of pathologies to facilitate a palaeodemographic reconstruction of the Volders population.

To conduct the first ever attempt at identifying individuals in an archeological burial complex who were engaged in transhumance or residing at higher altitudes by analyzing the stable isotope $\delta^{18}O$ of oxygen in the structural carbonate component of bone.

To determine the nutritional status of all 145 individuals in the archeological remains utilizing the stable isotope $\delta^{13}C$ of carbon and $\delta^{15}N$ of nitrogen in bone collagen and the stable isotope $\delta^{13}C$ of carbon in structural carbonate samples.

To observe the existence of trophic level differences amongst adult individuals that indicate significantly variable protein intake.

To determine if there is a significant difference in nutritional status between individuals found residing at higher altitudes and those found to be living at the valley floor.

To analyze $\delta^{13}C$ values in bone collagen to determine if freshwater fish were consumed.

To determine weaning age amongst the infant group using $\delta^{15}N$ values.

To determine chronological age at death by utilizing both a new automated counting procedure specially designed for the tooth cementum annulation (TCA) method and the existing manual TCA counting procedure.

3.1 Importance of skeletal collections for future studies

One of the fundamental cornerstones of human osteoarchaeology is ascertaining past health conditions in a population. The Global History of Health Project (http://global.sbs.ohio-state.edu/), for example, is a recent data collection endeavor conducted by a multinational research group including the State Collections for Anthropology and Palaeoanatomy in Munich, Germany, with the goal of accumulating comprehensive skeletal information pertaining to global human health spanning a 10,000 year period. Establishing an

anthropologically based research network designed to contribute, exchange and analyze osteological information taken from various skeletal series around the globe will ultimately lead to a clearer understanding of the evolution of palaeodemography worldwide. The project is a bold attempt at creating a massive database for future comparative studies focusing on the health of man. Questions regarding the fluctuations of health conditions through time, their regional differences and intricacies, the evolution of specific diseases and the cultural influence of varying levels of health quality, just to name a few, can all potentially be investigated. An example for this type of research was provided by Steckel and Rose (2002), which gives an overview of the demographic situation for the western hemisphere from the pre-Columbian native Americas through post-European invasion period.

Naturally, acquiring skeletal data from each of the diverse environments man was confronted with and eventually adapted to augments the spectrum of information available in the database. An alpine community such as Volders, adapted to a special set of environmental features, rural, yet at the crossroads to several cultures, represents a unique situation that certainly parallels another group somewhere else in the Alps or a different part of the globe. The health and wellbeing of people has a significant impact upon their mental, physical and social development, their ability to function efficiently as an organism, cope with environmental stresses, learn, adapt and survive. Palaeopathological diagnosis coupled with skeletal analysis and nutritional information can make a valuable contribution towards understanding these health related features.

Larsen (1997) accurately noted that studies of archaeological samples are important in addition to those based on industrialized urban populations because they provide a wider context for assessing the roles of genes and environment over time. The medieval Europe marks another era in human cultural evolution in which people made advances in technology, agricultural efficiency, land use management, craftsmanship, the manufacturing and trading of goods, civil organization and administration, education and science. In the face of unwavering human progress, the bulk of the people nonetheless remained bound to the land. Fulfilling subsistence needs still was a priority carried out by the peasantry. Interestingly, the farming community, although not part of the upper class that benefited in many ways from their social status, was through their occupation perhaps nutritionally healthier than their noble superiors. Certainly these individuals were exposed to backbreaking labor and work related dangers resulting in musculoskeletal problems and traumatic injury, however, they were physically active, may have had access to foods even during periods of famine and were probably organically not as unhealthy as sometimes presumed. Establishing palaeodemographically

related health parameters for the Volders group will promote a clearer understanding of health conditions that existed during this time period.

A future objective will be to incorporate the osteological data acquired from the Volders skeletal material into the Global History of Health Project database, thereby providing other researchers the opportunity of using this information for new comparative studies.

4. Bone structure and composition

Because both bone and teeth are essentially the only substrates used for the various examinations and analyses presented, it is necessary to provide a description of the individual structures pertinent to this study. According to White & Folkens (1991), human skeletal elements display a great variety of shapes and sizes, yet can be categorized into several basic forms that often share certain features amongst one another. Long bones, which are cylindrically shaped with flaring ends, belong to the elements comprising the upper and lower extremities. Flat, tabular shaped bones can be seen in the regions of the skull, shoulder, pelvis and rib cage. The carpal, tarsal and vertebral bones are block-like and exhibit numerous different forms. Irregardless of shape or size, bone tissue is basically the same at the macroscopic and molecular level. Bone tissue is composed of two major components: an inorganic mineral fraction (bone ash), which composes 70% of bone's dry weight in adult humans, and an organic matrix, which composes approximately 30% of bone's dry weight.

Collagen composes 90% of the organic matrix, also called osteoid, in dry, fat free bone (Price 1989). A number of other components are also contained in bone to a lesser extent, yet play important roles in its makeup and function and include water, ground substance proteoglycans, non-collagenous proteins, which are thought to be involved in regulation of bone mineralization, and also fats, vascular elements and cells (Wheater et al. 1987). Collagen is the most abundant protein in vertebrates and comprises the primary fibrous component of supporting tissues. Nineteen different types have been identified (I-XIX), and are distinguished based upon morphology, amino acid composition and physical properties (Martin et al. 1998). In bone, Type-I collagen, which belongs to a group known as fibrillar collagens, functions to provide tensile strength and resilience, two important attributes that make bone such a dynamic tissue during life and so durable after death. Type-I collagen is also found in the skin, tendons and ligaments and its structural arrangement differs in these tissues according to the mechanical support required. According to Burkitt et al. (1993), collagen is secreted into the extracellular matrix in the form of tropocollagen or propeptide, which consists of three polypeptide chains (primarily two α1 chains and one α2 chain) bound together to form a triple helical structure 300nm long and 1.5nm in diameter. The amino acid sequence of the individual polypeptide chain is Gly-X-Y repeats, a peptide arrangement that results in a coiled, left-handed helix (Currey 2002). The Gly residues are at the center of the triple helix and the X and Y residues at the surface of the helix. In one-third of the cases X is a proline and Y is hydroxyproline; the presence of hydroxyproline is essential to stabilize the triple helix and is a unique characteristic of collagen molecules. When three of the helically

coiled polypeptide chains assemble, they form a right-handed triple helix (Rossert & de Crombrugghe 1996). These molecules subsequently polymerize to form collagen fibrils. Parallel collagen fibrils further aggregate in a process of systematic "quarter staggered" overlapping to form strong bundles 2-10µm in diameter. It is precisely this tough, fibrous quality of Type-I collagen combined with the rigid, hardness of inorganic hydroxyapatite crystals interspersed within the gaps between the collagen molecules that produces the unique structural integrity characterizing both human bone tissue and the skeletal framework as a whole (Lowenstam & Weiner 1989).

In order to facilitate the analysis of this organic component, collagen must first be isolated from the other bone constituents. The process of collagen extraction from bone involves its separation from the inorganic components of bone resulting in the formation of gelatin (see methods, section 8). The complex triple helix structure of this protein is destabilized when the intermolecular bonds holding them together are broken following a heating step during the last phase of the extraction procedure, which causes the otherwise tough and insoluble polypeptide chains to disband. Gelatin is created when these unwound chains randomly tangle and fold back upon themselves during cooling. In order to isolate the mineral fraction, the same structural characteristics that make collagen and non-collagenous proteins extractable are utilized to facilitate its denaturing and removal.

According to McCarthy and Frassica (1998), the mineral component of the human skeleton including the teeth, is comprised primarily of a crystalline calcium-phosphate composite ($Ca_{10}[PO_4]_6[OH]_2$), a molecular complex that is analogous to the geological molecule hydroxyapatite (Grupe et al. 2005). In comparison to this form of hydroxyapatite, bone mineral itself possesses deficiencies with respect to calcium and hydroxyl ions, and is also characterized by numerous substitutions, mainly through carbonate. The majority of this carbonate is type-B, which substitutes for phosphate ions, and is different from type-A in that this form substitutes for OH groups (Boskey 1999). The quantity and type of substitution have a direct influence on the solubility of the bone mineral. During adulthood the fraction of carbonate increases, in contrast to the phosphate fraction which decreases, however, the total ion sum of carbonate and phosphate always remains constant. Therefore the actual mineral fraction of the human skeleton can be illustrated with the molecular combination $Ca_{8.3}(PO_4)_{4.3}(CO_3)_x(HPO_4)_y(OH)_{0.3}$, with $x + y = 1.7$ (const.) The mineral's apatite crystal structure is small relative to tooth apatite or the geological analog, and are hexagonal, symmetric, and average 5 x 5 x 40nm in size (Martin et al. 1998).

Bone continually undergoes both a modeling and remodeling process (Boskey 1999). Modeling simply involves the formation of bone in places where it has not been before and remodeling refers to the formation of bone in places where it has already been. Bone is actively remodeled for two primary reasons. The main functions are to maintain mechanical strength by replacing fatigued bone whose structural integrity is compromised and to facilitate mineral homeostasis. Within the span of one year, approximately 2-3% of cortical bone is turned over consistent with the maintenance of mechanical properties (Dempster 1999). Cancellous bone experiences a greater amount of turnover, primarily because it is more heavily involved in mineral homeostasis.

5. Tooth structure and cementum analysis

The phenomenal durability of teeth has made them a significant part of the fossil record and they have been the focus of many studies in zoology, anthropology and palaeontology, and have proven invaluable in systematics, investigations into dietary adaptation and species evolution (Hildebrand 1995). Various aspects of tooth form, types of dentition, their different functions and their development have long captured the interest of scientists. This particular section concentrates on the use of archaeological dental material originating from the human skeletons recovered at the Volders cemetery to make an age at death diagnosis in adult individuals and to acquire information about certain aspects of these people's life history that will be discussed in more detail later in this section.

One of the most consistently troublesome tasks confronting osteologists during the examination of human skeletal remains is the estimation of age at death. Mays (1998) stated, "At present, the lack of a wholly satisfactory technique for estimating age at death in adult skeletons from archaeological sites is one of the most thorny problems facing human osteoarchaeology". A diverse array of osteological methods already exist that aim to provide a solution to this important problem. The pitfall however, is that virtually all of them rely on identifying and quantifying age related skeletal changes under the long accepted assumption, that aging is naturally accompanied by specific alterations in the skeletal structure, for example, progressive cranial suture closure or surface changes in the pubic symphysis (Meindl & Lovejoy 1985, Suchey & Katz 1986). Many osteologists have voiced their concern over this and indicate there is substantial evidence to show that age estimation results derived from archaeological skeletons in fact, occasionally drift astray from the expectations based on this assumption (Houck et al. 1996). The effort to refine traditional methods and also develop new, more reliable techniques continues. One area where progress has been making headway concerns the histological analysis of tooth cementum annulation or TCA, a relatively new method for establishing age at death that has shown some promising results. This aging technique differs from others that have their foundation in the observation and categorization of morphological indicators on the skeleton itself, that in effect, are really indicators for biological age and not chronological age. The TCA method is based on the actual counting of specific microscopic structures in tooth root cementum, which will be discussed shortly, that are believed to be regulated by a set of processes resulting in the addition of one recognizable microscopic structure annually throughout an individual's lifetime.

5.1 A brief history of TCA

Tooth cementum annulation (TCA) is an analytic method designed to determine chronological age at death by counting the incremental lines of acellular extrinsic fiber cementum (AEFC) on the tooth root. It promises to be more effective than morphological estimates, which reflect biological rather than actual calendar age. AEFC, which is located around the cervical two thirds of each deciduous and permanent tooth, undergoes appositional growth resulting in the even layers of alternating dark and light bands (see Fig. 5.2). One pair of dark and light band constitutes one incremental line. The number of incremental lines counted is added to the year of eruption of the respective tooth and results in the chronological age estimation of the individual under study.

The possibility of establishing a direct relationship between age estimations and incremental lines in tooth cementum was initially explored during the 1960's on feral mammals (Klevezal & Kleinberg 1967). In the following decade a number of studies continued to examine teeth for this purpose, notably Grue and Jensen (1979), who analyzed the alternating light and dark cementum incremental lines microscopically to establish the ages of terrestrial mammals. The same method was later used by Stott et al. (1982) on human tooth samples as an age criterion in forensic dentistry. Condon et al. (1986), also concentrating on the use of incremental lines in age determination and found the method reliable. Further attempts to refine the histological technique were made by Naylor et al. (1985). Other researchers expressed their wariness with regards to the method and warned of the variables in pre-selected teeth that could affect the reliability of the TCA calculations (Lipsinic et al. 1986). Kagerer and Grupe (2001a) also voiced concerns, that pathological conditions affecting the dentition may possibly diminish or increase the number of incremental lines of the AEFC and lead to erroneous age outcomes. This cautionary note is especially important in light of the high incidence of periodontal disease observed in humans.

Traditionally, the counting of these lines has proceeded manually. It is time consuming, tedious, requires unwavering patience and prolonged visual concentration, and most importantly, is often accompanied by a high rate of interobserver variability. Recently, however, several efforts have been made to develop an automated counting procedure including (Wittwer-Backofen et al. 2003 and Czermak et al. 2006). The intention is to reduce observer subjectivity and burden, increase the rapidity of the counting procedure itself and improve the overall accuracy of the results.

Wittwer-Backofen et al. (2003), in their quest to provide a more reliable method for determining the age of human skeletons using TCA, have developed a refined preparation

technique, including improved digital graphic procedures and enhancement strategies to produce digital images with a specifically adapted software package. Their study involved using 363 known age samples and resulted in a 95 % confidence interval for tooth cementum annulation. Czermak et al. (2006) developed an automated counting procedure using Gauss lowpass filtering, Discrete Fast Fourier Transformation (DFFT) and algorithms for image analysis and pattern recognition. Advantages of this technique include the capability of discerning a greater number of gray areas compared to the human eye, the removal of distracting image artifacts, the ability to manipulate a given image by converting it to a software friendly format, which ensures optimal incremental line recognition yielding up to 400 individual counts within a specific region of interest (ROI) with substantially increased rapidity, and the subsequent statistical test of the results. The software was utilized and tested during the examination of archaeological skeletal material originating from the early medieval cemetery Etting-Sandfeld in Ingolstadt, Bavaria and also from skeletal material deriving from modern situations. The results computed by the auto-TCA software showed a very good correlation with age estimates calculated by morphological analysis of the skeleton and also those achieved by the manual TCA method. For the automated line counts in this study, the software program developed by Czermak et al. (2006) was used.

In addition to age estimations, Kagerer and Grupe (2001b) found that differential quality of incremental lines can serve as a valuable tool in identifying certain aspects of life history. Events that influence the calcium and phosphate biochemistry of the organism, such as pregnancies, skeletal traumata or renal dysfunction have been shown to affect the process of cementogenesis, leaving microscopically visible alterations in the form of the incremental lines.

5.2 Cementum structure

To understand the makeup, function and location of tooth cementum, a brief explanation is necessary. Cementum is one of four tissues that support the tooth in the jaw (periodontium), the others being alveolar bone, the periodontal ligament and gingivae (see Fig. 5.1). Its primary function is to provide an attachment point for the collagen fibers of the periodontal ligament, known as Sharpey's fibers. The periodontal ligament or membrane, which is composed of dense collagenous tissue, forms a thin fibrous attachment between the tooth root and the alveolar bone and acts as a sling for the tooth within the socket, permitting slight movements which cushion the impact of masticatory forces (Burkitt et al. 1993). Sharpey's fibers run obliquely downwards from their attachment in the alveolar bone to their anchorage

in the cementum at a more apical position on the root surface. The points of attachment of these collagen fibers in both cementum and bone are in a constant state of reorganization to accommodate changing functional stresses upon the teeth. The alveolar bone itself is stimulated by the mechanical tugging movement of the Sharpey's fibers exerted upon it during occlusal loading and results in osteoclastic resorption and deposition at different areas of the socket.

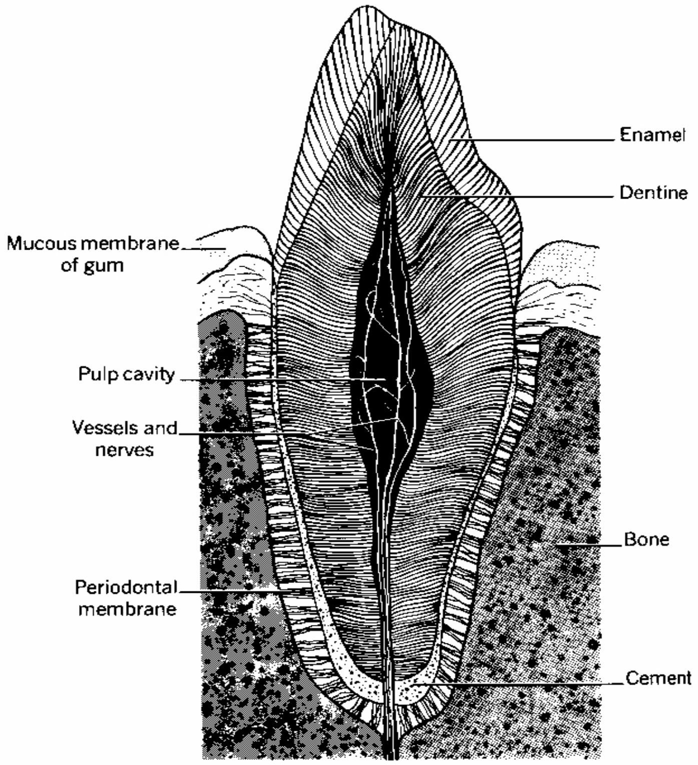

Figure 5.1. Longitudinal section of an incisor tooth and its basic anatomical structure (Hildebrand 1995).

This bone stimulation is somewhat akin to the way in which bones of the extremities, especially the points of tendon attachment, react to skeletal muscles during physical work or exercise strain.

Some aspects of tooth cementum are well documented yet, little is known about its origin, differentiation and the cell dynamics of the cementum-forming cell (cementoblast). It is very resistant to resorption, is formed continually throughout a lifetime, a process which allows for the perpetual reattachment of the periodontal ligament, and its heavy deposition at the apex can be viewed as a compensatory physiological response to occlusal wear (Berkovitz 2002). In addition, it is a highly responsive mineralized tissue responsible for maintaining the

integrity of the root, helping to maintain the tooth in its functional position in the mouth, and is involved in tooth repair and regeneration.

According to Burkitt et al. (1993), cementum consists of a dense, calcified organic material similar to the matrix of bone and has no osteons. Berkovitz (2002) indicates further, that cementum is classified according to the nature and origin of the fibrous matrix. If it originates from the periodontal ligament, then it contains extrinsic fibers and if it originates from cementoblasts then it contains intrinsic fibers. In humans, two basic cementum types exist, an acellular and a cellular. However, among these two types a number of varieties have been identified. The acellular form as the name suggests, contains no cells and covers the root adjacent to the dentine. Acellular extrinsic fiber cementum (AEFC) is the first cement formed and found on the cervical two thirds of the root (Fig. 5.2). AEFC layers form slowly, are approximately 15µm thick and well mineralized. Their collagen comes from Sharpey's fibers. Cellular intrinsic fiber cementum (CIFC) is generally cellular, forms more rapidly than AEFC, has no Sharpey's fibers, plays no role in the attachment of the root and is found on the apical third of the tooth root. When CIFC forms at a slow rate and cementocytes do not become entrapped then it is called acellular intrinsic fiber cementum (AIFC). The different cementum types AEFC and CIFC can also occur together and be observed in alternating layers. This happens primarily at the root apex and the tissue is referred to as cellular mixed stratified cementum (CMSC). When CMSC is slowly formed it produces a well mineralized acellular variety and is called acellular mixed fiber cementum (AMFC). A more rapidly formed, less mineralized cellular variety is known as cellular mixed fiber cementum (CMFC). Yet another type called afibrillar cementum, a thin acellular form with a well mineralized ground substance is found extending onto the tooth enamel near the cemento-enamel junction. The cellular cementum containing cementocytes, which is found in the apical areas, overlays the acellular cementum to some extent. Histometrically, cementum is thinnest at the cemento-enamel junction (10-15µm), becoming progressively thicker and irregular towards the tooth root apex (50-200µm, although it may exceed 600µm) where cementocytes are often entrapped in spaces called lacunae.

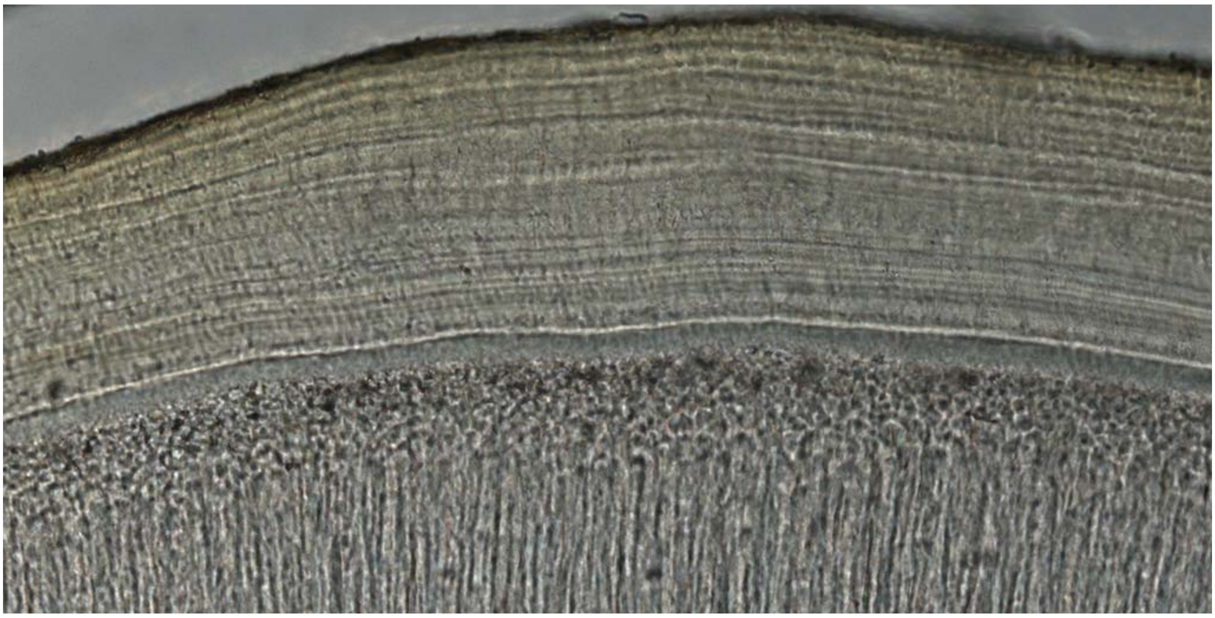

Figure 5.2. Volders burial 57. A photomicrograph showing the histological cross section of a tooth root illustrating the appearance of AEFC. The bright white line running through the center of the image is the cementum-dentin-junction (CDJ), directly below it is the granular layer of Tomes, which in turn is adjacent to the root dentine (premolar, 70µm, 20x).

Cementum contains on a wet-weight basis 65% inorganic material, 23% organic material and 12% water. Thin plate-like hydroxyapatite crystals averaging 55nm wide and 8nm thick compose the main portion of inorganic components. Collagen, virtually all of which is Type I, a type found mainly in fibrous supporting tissue with a biochemical composition that confer it with great tensile strength, makes up the organic matrix (Berkovitz 2002).

Cementum is deposited in an irregular rhythm resulting in the unevenly spaced incremental lines of Salter (Berkovitz 2002). In acellular cementum, these lines appear thin and evenly spaced. In the more rapidly forming cellular cementum, the incremental lines are irregular, thicker and farther apart. When viewed under a light microscope, a cross section of these incremental lines surrounding a tooth root appear as layers of alternating dark and light bands (see Fig. 5.3). Differences in the degree to which each of the two layers are mineralized results in this observable structural characteristic. Environmental and physiological factors apparently both influence cementogenesis and an interplay between parathyroid hormone, calcitonin and vitamin D is hypothesized to be the reason for the differences in mineralization. Parathyroid hormone, also called parathormone, is synthesized and released by the parathyroid gland and regulates the distribution of calcium and phosphate in the body (Müller 2004). Vitamin D, a sterol with hormone-like functions, maintains adequate plasma levels of calcium by enhancing the absorption of calcium and phosphorus from the intestine,

minimizing the loss of calcium by the kidney, and stimulating their resorption from bone or promoting their deposition in bone. The endogenous precursor of vitamin D, cholecalciferol (vitamin D_3), is converted from 7-dehydrocholesterol in the dermis and epidermis through the action of ultraviolet light during sunlight exposure (Champe et al. 1994). The intensity of UVB rays corresponds to a seasonal cycle, which in turn is thought to be reflected in the degree of mineralization seen in the incremental lines. To reiterate, a singular incremental line, consisting of one light and one dark band, are thought to represent one calendar year.
Other microscopic structures adjacent to the cementum are also visible in the cross section. The cementum-dentin-junction (CDJ) also called the hyaline layer of Hopewell-Smith or intermediate cementum, is observable between the cementum and the granular layer of Tomes (see Fig. 5.2), and is the point of attachment between cementum and dentin (Ho et al. 2004). The granular layer of Tomes is an imperfectly calcified tissue made of small interglobular spaces that give it a granular appearance and is found at the peripheral border of the mantle dentin (Ten Cate 1972).

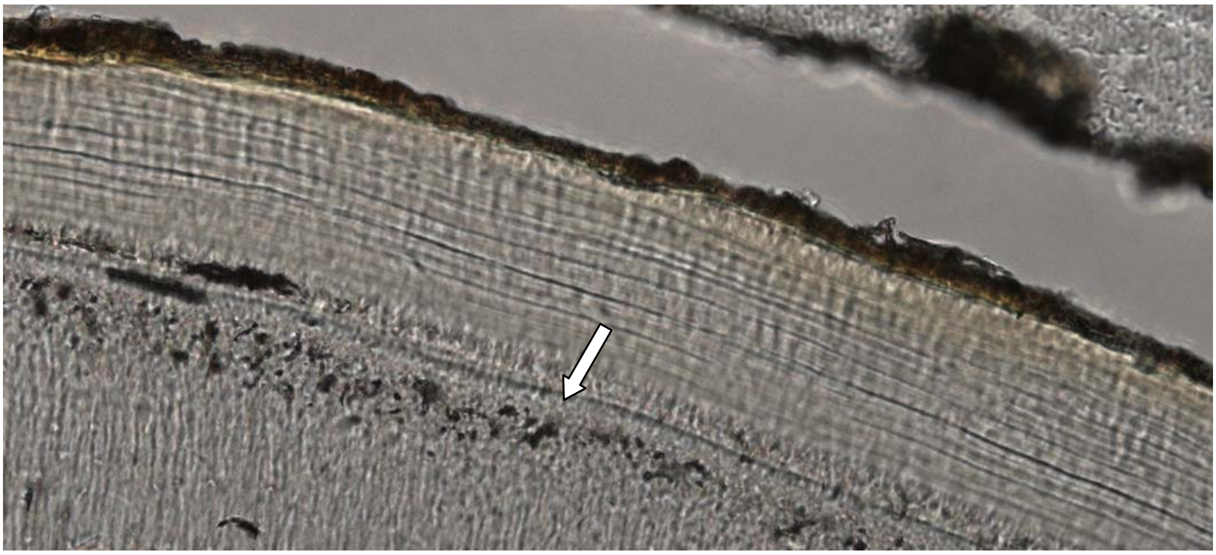

Figure 5.3. Burial 144. Photomicrograph of a tooth root cross section. Root dentin at the lower portion of the image and the incremental lines composing the cementum adhering to it. The dark black line above the granular layer of Tomes (arrow) is probably afibrillar cementum.

The incremental lines exhibited in the acellular extrinsic fiber cementum (AEFC), the layer on the cervical two thirds of the tooth root whose deposition is presumed to follow a seasonal rhythm, are the focus of interest here and the object of scrutiny in this TCA analysis.

6 Taphonomy at Volders

Over the past 30 odd years, scientists have progressively come to realize how vital insight into the postmortem history of the bioarchaeological material they are studying is with regards to the accuracy of the results upon which they are based. Anything that changes the morphological or macroscopical appearance of biological material and the situations in which they are found, or the microscopic structure or molecular composition of biological tissue, must be considered just as scrupulously as the results themselves. Acute awareness of these changes proved imperative throughout the Volders study, from the initial archaeological and osteological work in the field up to the final evaluation of the collagen based archaeometric analyses results. This section includes a broad coverage of the emergence of the field of taphonomy and its importance for interpretations of biological remains, primarily osteological, within the archaeological context. The diverse taphonomic processes encountered during the course of the Volders excavation as well as those affecting the laboratory work are discussed.

Taphonomy as a discipline finds its first application in paleontology where its goal was to explain "taphonomic histories", the environmental factors that affect organic remains between an organism's death and its final representation in the fossil record (Haglund 2003). Presently, a diverse array of sciences including physical anthropology, palaeontology, forensic medicine and archaeology all require taphonomic information to provide explanations for the things they see, and the reconstruction of palaeoenvironments, human behavior and subsistence patterns are all aided by taphonomic approaches. The importance of taking taphonomic histories into consideration as standard operating procedure is relatively recent (Nicholson 2001) and has helped researchers evaluate and interpret archaeological bone assemblages as well as reevaluate previously examined situations to properly interpret them (Pickering et al. 2004), or for example, R. A. Dart's startling, yet, erroneous conclusions regarding head hunting and cannibalistic behavior supposedly practiced by the early hominid Osteodontokeratic Culture, which was based upon the Makapansgat *Australopithecus prometheus* (today called *Australopithecus africanus*) finds (Dart 1957). Confusion in bioarchaeological findings has often been generated by the diagnosis of pathologies or description of perimortem situations that are in fact non existent, a circumstance that generally originates from the misinterpretation of postmortem changes. An essential aspect of the gross osteological examination in the field, and later on in the lab, is to discern taphonomic artifacts that result in postmortem modification from true signs of perimortem trauma.

Prior to the recognition of taphonomy as a field or the importance of understanding what influence taphonomic processes can have on bioarchaeological remains or the archaeological situations in which these remains are found, numerous bioarchaeological situations involving misconstrued observations or misinterpreted data were predetermined to happen. Although Efremov coined the term taphonomy in 1940 and referred to it as the science of the laws of embedding or burial to better describe the processes acting upon biological material spanning between an organism's death, burial and recovery, the idea behind it was by no means novel (Efremov 1940). The initial mention of various taphonomic processes, even though they were not called this, and their effects on biological material was confined to animal remains. According R.L. Lyman (2002), early signs of awareness that processes were occurring that left their marks on ancient tissues include Buckland in 1823, who observed hyenas destroying bones and concluded that ancient bones with similar damage were destroyed by ancient hyenas, and later in 1860 with Lartet who observed prehistoric butchering marks in extinct animals.

Only later was taphonomy used to clarify human burial and preservation situations, and today it plays an essential role in comprehending and explaining burial situations and macroscopic, microscopic and molecular observations. Weigelt in 1927 proposed the term biostratinomy and defined it as the study of environmental effects on organic remains that take place between the time of death and its burial (Darwent & Lyman 2002). Müller in 1963 proposed the term "Fossildiagenese", today known as "diagenesis", to denote the affect on organic remains that take place between the time of burial and their recovery by a palaeontologist or archaeologist. Taphonomy is in fact a comprehensive term and involves both biostratinomic and diagenetic variables. The chronological period over which its processes take effect can be divided into three distinct phases, namely the time of death, time of deposition in the recovery location, and the time of recovery.

One of the first comprehensive texts offering a description of taphonomic processes and how they influence osteoarchaeological material was published by Pat Shipman in "Life History of a Fossil" in 1981. Although the work draws primarily from her experiences in Africa, many of the situations she illustrates could happen anywhere. The cycle of life to death to fossilization inherently involves a loss of information regarding the history of the organism represented in the human, faunal or even floral remains. At the very core of the problems posed by taphonomic processes is the potential that the information representing the original situation is not only lost completely but transformed into a new and invariably misleading one. Although some disciplines such as forensic science rely on taphonomic processes such as

scavenger modification, exposure to water, position of body, and decomposition to provide them with valuable clues, bioarchaeologists, out of necessity, must be in the position to identify and exclude the possibly misleading effects of these same processes in order to acquire relevant or accurate data. According to Sorg & Haglund (2002), taphonomic data are evidence in forensic pathology, whereas in bioarchaeological they are bias to be stripped away to get at truth. This point is interesting because it shows how similar sciences are driven to different conclusions based upon the goal of their individual examinations.

In histological and chemical analysis, the potential of misconstruing results that are based upon erroneous data skewed by the molecular alteration of bone or tooth material is indeed omnipresent and should be of paramount concern for the bioarchaeologist. Where this same type of change might provide vital clues to the forensic examination, it can prove frustrating for the bioarchaeologist, especially when the damage is extensive. Twenty years prior to Sorg and Haglund's (2002) observations, Shipman (1981) emphasized the importance of recognizing biases that influence efforts focused on reconstructing palaeoecology based on the fossil record. The main difficulty she adds is that the fossil record provides an obscure, imperfect glimpse of a few of the original ecosystem's many elements. A contemporaneous work describing specific taphonomic variables contributing to the bias affecting interpretation such as the transport of individual bones away or to an assemblage, the differential survivability of bones, and how they can be altered by human and animal consumption was provided by Brain (1981).

Marean (1991) further defined taphonomic influences as "postdepositional processes" and described the importance of identifying chemical and mechanical action on bones and teeth after the bones and teeth have entered the sediment and are no longer sources of food for mammals.

An important and necessary distinction that effectively categorizes types of taphonomic processes was proposed by Nawrocki (1995), by dividing the environmental factors of human taphonomy into *biotic* and *abiotic* categories. Biotic factors involve living organisms such as carrion eaters, burrowing rodents, smaller organisms like fungus and bacteria, and also trees and shrubs whose root system can either destroy mechanically by root pressure or penetration and through the effects of acid that can either etch or completely dissolve cortical bone. Roots, however, can sometimes help preserve burials by drawing water away from the bone, which otherwise can result in cracks and fissures through swelling, drying and freezing. Water is one of the most devastating abiotic environmental factors that exists and is capable of incurring substantial mechanical or hydrolytic destruction to bone. Other abiotic factors are

temperature, sunlight, soil pH and depth below surface. With the exception of scavenging, the resulting damage of all the above mentioned processes was observable in the Volders skeletal material and burial area.

When biotic agents, faunal or floral, cause the physical disturbance of skeletal remains, resulting in a movement or jumbling of the bones, the process is collectively known as bioturbation. Skeletal elements are occasionally shifted from their original anatomical position or even carried away, a phenomenon that can lead to false interpretations. For example, a cranium pushed away from its true position by tree root growth or rodent activity found laterally disassociated from the postcranial skeleton, could be presumed at first glance to indicate a decapitation. A careful examination of the skull base and the cervical vertebrae to identify the telltale cut marks left by the striking blade, however, would suffice to rule this hasted conclusion out. There did not appear to be any definitive signs of bioturbation for primary burials at the Volders site other than destruction to portions of a primary burial or displacement of individual bones through an adjacent, later burial and digging related to building projects.

One of the most significant and sometimes overlooked modifiers of remains, human or nonhuman is man. What is pertinent for the forensic pathologist also bears great relevancy for the bioarchaeologist interested in historical settings since homicide, dismemberment, warfare, scavenging, cannibalism, burial ritual, and grave robbing were all actively practiced in the past as well as the present, and represent an essential dimension of taphonomy. In more recent memory, various regions of the world have been marred by war, civil unrest, military invasions, brutal dictatorial oppression and politically or racially motivated aggression that have left unspeakable destruction to human life in their wake. In response to the horrific aftermath of these tragic conflicts, the well known forensic anthropologist William Haglund stated that “Human agency is critical in the resolution of human rights abuses, including mass fatalities or mass burials” (Haglund et al. 2002). Other less macabre and understandable forms of human intervention that result in taphonomic alteration of skeletal material or assemblages involves the historical cemetery situation.

Humans as the engineers of destruction to bone complexes and contributors to the loss of information, intentional or otherwise, are frequently documented and widespread. The Volders dig proved to be no exception. The cemetery situation poses its own challenges to archaeologists and bioarchaeologists, and is often the site of substantial information loss. The active loss of information usually begins with the inadvertent discovery of a given burial grounds, as in the case of Volders, which was discovered in the process of building an

apartment complex. The initial taphonomic intrusion in this scenario was conducted by the twenty ton backhoe that destroyed approximately five graves, conservatively estimated. Once the cemetery is located, irregardless by what means, one of the challenges presented to the archaeologists is establishing where the burials are situated, which is conducted based upon recognition of the grave outline visible due to the differences in soil color or composition, a distinctive feature that is not always clearly perceivable to the untrained eye. This is of particular import during the course of an excavation involving an inexperienced digging crew and can lead to unfortunate and unavoidable difficulties. Significant and irreparable damage is incurred upon skeletal remains through unsystematic, careless or overly enthusiastic shoveling or scraping, which translates into significant and irreparable loss of information. This damage can be quite extensive especially when the skeleton is that of an infant, small child, or the bone substance is softened and in an unstable condition. An entire human neonate skeleton can be removed unnoticed with one single exuberant swing of the pickaxe. Ignorance of human bone anatomy is a further problem commonplace at the excavation and is best illustrated when human bones are mistaken for animal remains and discarded, in itself a lamentable and persistent habit. Still practiced by the unknowing, faunal remains encountered during cemetery digs are meticulously removed from the grave situation and thrown away much to the dismay of archaeozoologists and bioarchaeologists interested in the data animal bones can yield. Because all of these situations were ubiquitous at the Volders excavation, it was paramount to work together with the new members of the digging team in order to minimize the damage.

One positive aspect to the cemetery situation is that excavators get experienced quickly purely through repetition, thereby contributing to a reduction of active information loss. Another problem encountered in the cemetery situation is the packaging, transport and storage of large amounts of skeletal material. Primarily because of the inevitable lack of funds or time needed in acquiring the special boxes appropriate for this purpose, skeletons are conventionally placed or piled into fruit cartons complete with ventilation holes. These openings unfortunately guarantee that at least a portion of the smaller hand and foot bones are either strewn about or land in another fruit carton containing a different skeleton because they tend to be stacked up in towering columns. Even worse yet, they are sometimes squashed into bags. Breakage, fragmentation, molding through trapped moisture, and occasionally outright pulverization of fragile or wet bones are the result. At the Volders dig roughly 150 custom-made skeleton boxes were purchased to store the skeletons originating from primary burials. Alternately, large, tough fiber sacks normally used for packaging and transporting animal feed

were used to store bones from the grave fill when the carton supply was depleted. The positive aspect to this type of bag is that they are not hermetic and allow air to circulate, which hinders fungus growth, and they are not prone to tearing. Both occurrences common and unavoidable for bones placed in plastic bags, a storage variation still employed by many archaeology teams.

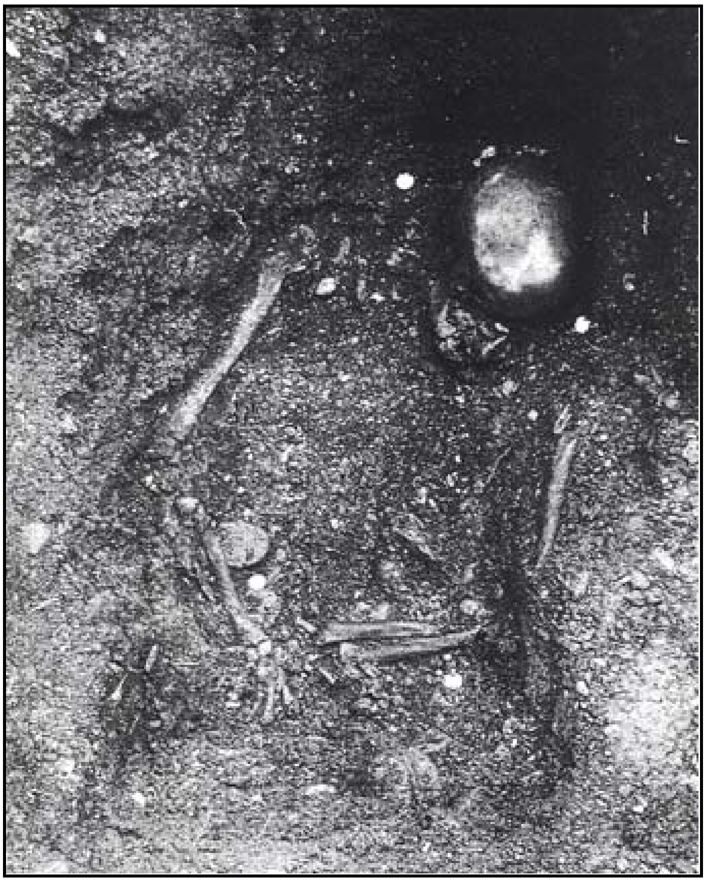

Figure 6.1. Volders burial 153 suffered significant damage from the riverbed gravel surroundings in which the body was originally interred. The lower portion of the skeleton was completely destroyed by the slow grinding action of the stones and water intrusion, and osteological data from the remaining postcranial skeleton had to be carefully documented *in situ* because the bone's preservation was so poor. Interestingly, the cranium was tilted upwards by a rock at the back of the head and survived fairly well intact. (Photo: G. McGlynn)

Yet, man is just one variable in the taphonomic equation. The burial environment itself is the stage and backdrop for many other taphonomic processes (Fig. 6.1). A gross breakdown of the Volders burial site stratigraphy depicts an approximately 30 cm deep top soil layer covering a roughly one meter layer of light brown colored, loose earth and clay, which is followed by a deep, coarse gravel bed typical for local rivers.

This does not imply, however, that skeletons buried deeply are always better preserved, indeed Lyman (1994) has indicated that the weight of sediment alone can cause bone deformation, crushing, fragmentation and post-depositional movement. Bone deformation caused by the combination of moisture and soil weight is often observable in archaeological material, particularly the crania. It should be noted that all of the aforementioned processes were recorded on various skeletons from the Volders site.

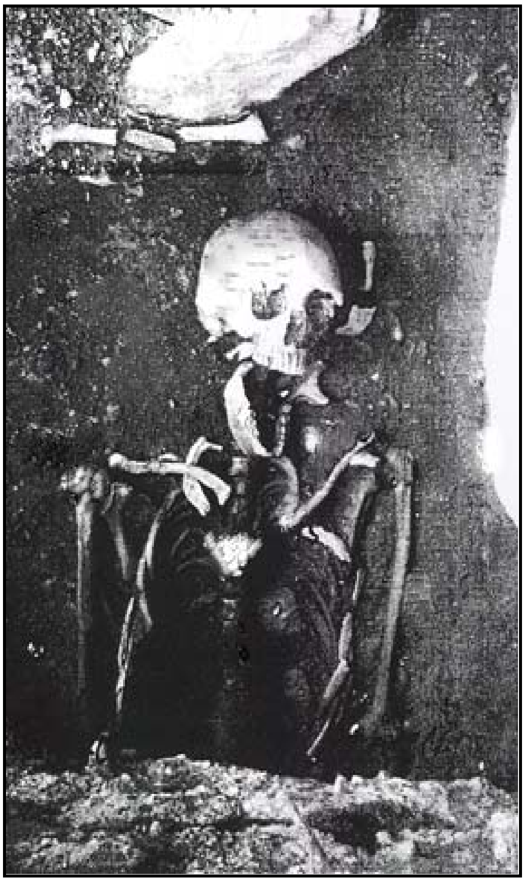

Figure 6.2. Volders burial 135 pinned tightly underneath the neighboring house's garden retaining wall (at image bottom), which was built during the 70s. The pelvis, lower extremities, and forearms were solidly cemented into the foundation. (Photo: G. McGlynn)

Each of these burial mediums is associated with different taphonomic influences. The upper humus layer is commonly the site of periodic changes in moisture by way of rain, irrigation or flooding, fluctuations in temperature that result in freezing and thawing, microorganismal activity, burrowing and gnawing by rodents, and invasion by root systems. The shallow depth of burial also makes the skeletons in this layer highly susceptible to damage or rearrangement through digging, plowing, crushing by heavy machinery or building structures (Fig. 6.2).

Skeletons recovered in the deeper clay-like earth tended to be more protected from the deleterious effects attributed to the humus layer, although in one area of the Volders site, a deep trench was dug out by workers to dispose of building refuse resulting in the partial destruction of several burials.

Diagenesis

Lyman (1994) describes diagenesis as the alteration of biological material following burial, and distinguishes between the influences of *intrinsic* factors, those deriving from the tissue specimen itself such as size, porosity, and chemical and molecular structure, and *extrinsic* factors such as soil pH, water and temperature regimes, and bacterial action. An absolutely paramount prerequisite to understanding diagenesis is not to view the individual processes independent from one another, but to recognize their interplay. The interaction of soil and bone chemical constituents and its consequences for later analysis dependant on molecular structural integrity is probably the most crucial aspect to diagenesis. Changes in original bone chemistry due to mineralization, corrosion, leaching and enrichment must be considered and tested for. Because the chemical composition of bone can be used to reconstruct the diet of an individual, bioarchaeologists are interested in bone chemistry and the interaction of soil and bone chemistry.

According to Haglund et al. (2002), the local chemical environment is one of the major factors affecting the decomposition of human remains. Tissue decomposition refers to both soft and hard, calcified types. They indicate that prior to desiccation and the loss of its organic components, bone tends to respond to modification agents as though it were fresh, and is a process that is largely dependent on microenvironment. As bone elements are exposed to their surroundings, their composition changes. There may be staining of outer layers, weathering, or mineral uptake/loss into the soil or water. Grupe (2001) also indicates that decomposition is primarily a function of the burial environment and is less reliant on the duration of interment. The specific features characterizing a given burial environment represent the decisive factor influencing the level of preservation later confronting the anthropologist, histologist, or forensic scientist. This taphonomic aspect is probably the most important factor to consider in connection with bioarchaeological analysis focusing on the chemical composition of bones and teeth.

Dupras et al. (2006) indicates that weathering is an important and potentially damaging taphonomic process whose agents include cracking, staining, flaking and sun bleaching. Behrensmeyer (1978) defines bone weathering as “the process by which the original

microscopic organic and inorganic components of bone are separated from each other and destroyed by physical and chemical agents operating on the bone *in situ*, either on the surface or within the soil zone". Buried bones weather at a much slower rate than exposed bones, an obvious but important point with respect to intentionally buried skeletal remains such as those found in cemeteries or burial grounds. The drawback is that they are then exposed to the damaging effects of soil.

Several of the most damaging agents to buried bones are moisture, mentioned previously, oxygen and low pH. Dry, static, dense loess burial environments that possess a basic soil pH because of their high calcium carbonate content, will allow for bone preservation over great periods. The lower the pH, and the looser and moister the soil, the more damage will be incurred to bones due to erosion (Herrmann et al. 1990). Anthropologists are frequently confronted with information loss due to substantial levels of bone destruction caused by aggressive burial environments, especially ones possessing increased soil acidity or alternating wet and dry conditions (Lee-Thorp 2002). Entire skeletons can be completely dissolved, sometimes resulting in a shadow-like image of the once intact skeleton, rendering them unavailable for study. A number of factors intrinsic to bone itself contribute to their susceptibility to the local burial environment. Bone density, porosity, size, age of the individual, type of bone and presence of pathologies are all key factors directly influencing bone survivability and preservation of molecular integrity (Hedges 2002). For example, subadult bone is more susceptible to the deteriorating effects of diagenesis than adult bone because it is more porous and the cortical structure is thinner and less well mineralized. Together, these factors can also increase the likelihood that other serious threats will compromise the integrity of bone such as microbial invasion or chemical exchange.

Since the advent of histomorphological and chemical analysis of bone, the level of preservation necessary to successfully conduct these examinations is largely dependant upon the state of the tissue's molecular preservation. Microbial activity is capable of destroying the organic and inorganic components of bone and soft tissues, and microbes are probably the most common agent deleterious to the integrity of calcified tissues, the effects of which can be readily seen both at microscopic and molecular levels (Collins et al. 2002). The organic constituents of bone and soft tissues are the primary focus of microbial invasion and in bone, acquire easy access through the labyrinth of tunnels offered by the Volkmann and Haversian vessel systems. A comparison of the different degrees of attack is depicted in the Figures 6.3, 6.4 and 6.5. Although this scenario is expected in soft tissues and a common topic in forensic science, bones too, often exhibit extensive levels of destruction characteristic for this type of

activity. Some burial environments are particularly conducive to bacterial proliferation and others function to suppress it. Humid environments with warm, ambient temperatures promote microbial growth more so than hot, arid surroundings. According to Urzi and Krumbein (1994), microorganisms cause decomposition through enzymatic cleavage (of organic compounds), the production of strong acids (hydrolytic and corrosive), and physical destruction by the mechanical forces they generate during their growth.

Histomorphological studies are also detrimentally affected, for example, differences between woven and plexiform bone are difficult to assess when diagenetic alteration of the underlying collagen bundles has been disturbed, and basic microscopic bone structures are rendered indiscernible (Pfeiffer 2006). Microscopic tooth structures can be fully obscured by the darkly pigmented canals and radiating tendrils characteristic of microbes and fungi.

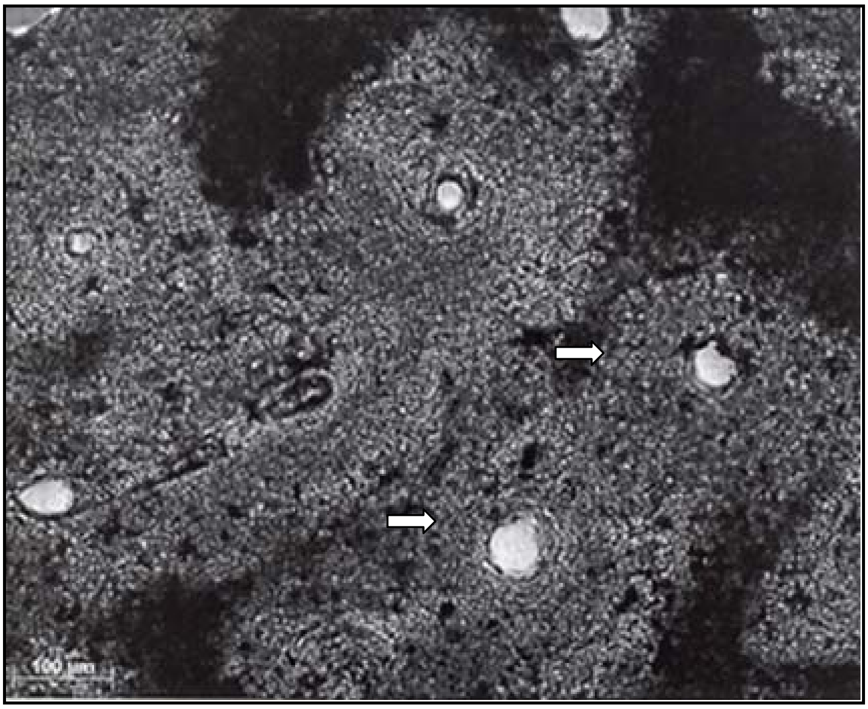

Figure 6.3. Photomicrograph of a human compact bone thin section showing extensive diagenetic alteration. Osteon borders (arrows) are barely visible and virtually no osteocytes are definable. (Femur, 70µm thick, 20x, C:N ratio 3.3, Volders burial 65).

Collagen

Collagen is relatively insoluble, owing to linkages between its triple helix polypeptide chains, and strongly bound to the inorganic matrix of the bone. Noncollagenous proteins are acidic polypeptides that adsorb strongly to the bone mineral matrix of hydroxyapatite. As bone

degrades, the adsorbed acidic proteins and peptide fragments are preferentially retained, whereas the collagen may be lost (Masters 1987). Therefore, fossil bone may contain only traces of collagen.

In an experimental study, Balzer et al. (1997) illustrated the destructive nature of soil bacteria on collagen type I in modern mammalian bone, and the bias this alteration can have for isotopic analysis of ^{13}C and ^{15}N isotopes, radiocarbon dating, and also for investigations focusing on DNA. Ratios for ^{15}N were shown to be altered by up to 5‰, a change in value which would generate entirely different conclusions if based upon these results. The biogenic decomposition incurred upon the organic matrix of bone by collagenase producing microbes can compromise the molecular structural integrity and was shown to proceed at an alarmingly rapid rate. Therefore, it must be presumed that ancient bone collagen destined for analysis is diagenetically altered, until shown otherwise (a case of guilty until proven innocent).

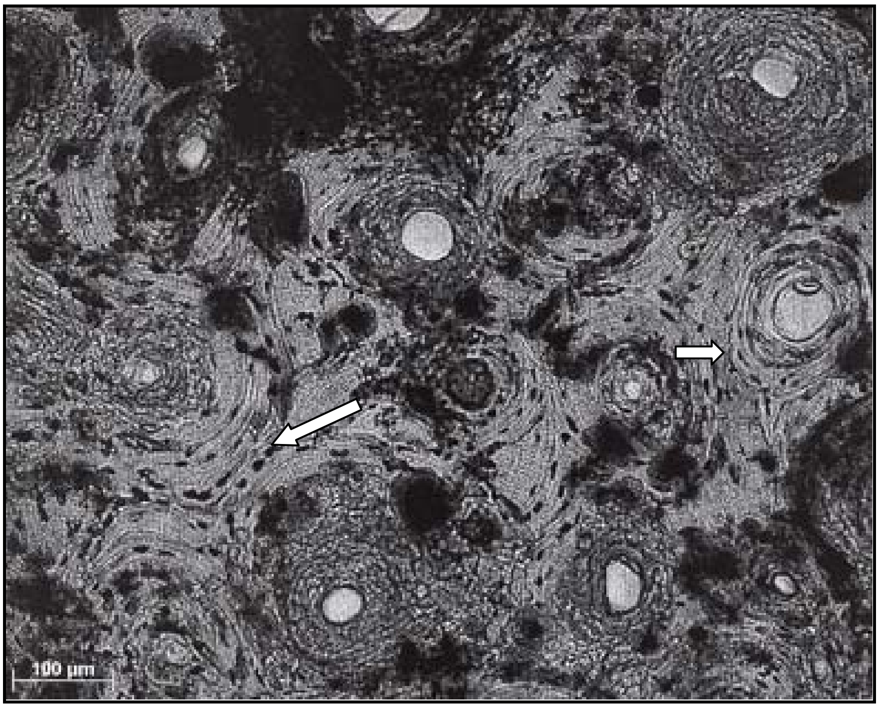

Figure 6.4. Photomicrograph of a histological thin section exhibiting moderate diagenetic alteration, with recognizable osteocytes (long arrow) and osteon borders (short arrow). (Femur, 70µm thick, 20x, C:N ratio 3.3, Volders burial 27).

Grupe & Turban-Just (1998), in an effort to identify the relationship between collagen decomposition and the state of degradation of archaeological bone, showed how these intertwining variables can bias archaeometric data. The isotopic signature of the collagen

molecule is inextricably bound to amino acid composition (Ambrose 1993), and bacterial collagenases cleave the helical biochemical structure of collagen thus altering the fundamental amino acid composition. The decomposition process apparently degrades specific amino acids like alanine and arginine preferentially because they are essential for the bacteria themselves, alanine for example, is a component of bacterial cell wall structure (Balzer et al. 1997 and Grupe & Turban-Just 1998). Biogenic decomposition is initially characterized by a rapid loss of carbon in a self-limiting process in which the reduction of carbon is dependant upon the presence of nitrogen necessary to facilitate the synthesis of bacterial protoplasm (Grupe & Turban-Just 1998).

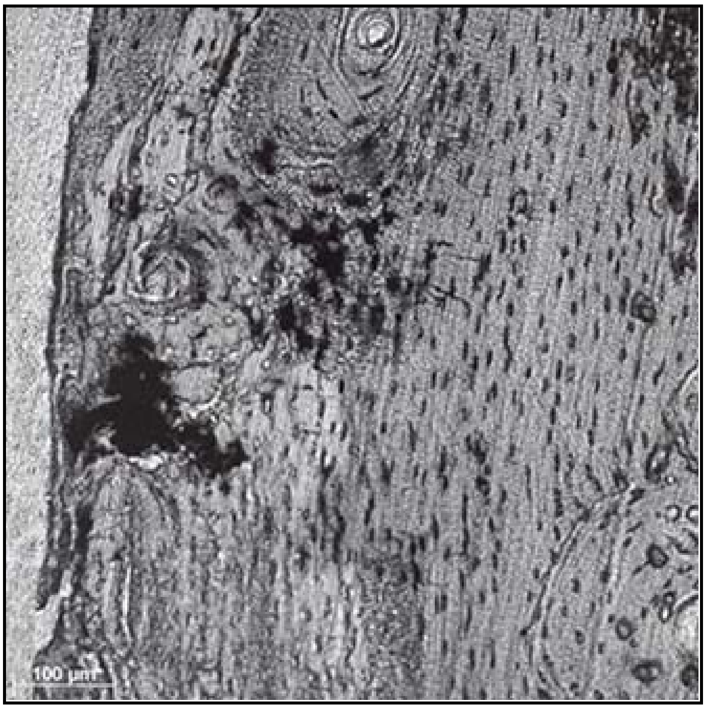

Figure 6.5. Photomicrograph of a bone thin section exhibiting minimal diagenetic damage. (Femur, 70µm thick, 20x, C:N ratio 3.2, Volders burial 50).

Grupe (1987, 1988b) and Grupe & Piepenbrink (1989) have pointed out that isotopic fractionation due to microbial activity may occur during early stages of diagenesis and asserted that if an excavated bone specimen exhibited signs of prior invasion by microorganisms that it is probably unsuitable for archaeometric tests such as trace element analysis.

It is precisely this diagenetically susceptible molar carbon to nitrogen ratio which functions as a specific marker indicating the preservation status of collagen protein and therefore its

usability for chemical analysis focusing on these molecules (Ambrose & Norr 1993). An examination of the equally fragile amino acid balance in collagen is another method of verifying the protein integrity of collagen and offers even greater accuracy than the C:N test. A limited number of these amino acid assays were conducted (for details see methods, section 8).

The proportion of carbon to nitrogen, referred to as the C:N ratio, is one of the most important indicators of collagen integrity, which in this case, can be interpreted to mean lack of contamination. C:N ratios are calculated by isotope ratio mass spectrometry analysis, which identifies the elemental composition of a sample producing the weight percent of C and N. The C:N ratio of collagen, based upon amino acid composition, is 3.21, which implies the presence of 3.21 times more carbon in the collagen than nitrogen (Ambrose 1993). The usual range of C:N ratios said to be acceptable for analysis are between 2.8 and 3.3 (Hedges 2000), although according to DeNiro (1985) and Ambrose (1990), ratios generally encountered in studies using bone range between 2.9 to 3.6, empirically speaking. Some authors suggest greater flexibility in the placement of the upper limits for analytic purposes (Bösl et al. 2006), and suggest that whole bone nitrogen content is an effective predictor of preservation (Coltrain et al. 2004). Although levels higher than 3.4 indicate probable contamination with carbon rich substances such as humic acid (Kennedy 1988), C to N ratio of up to 3.9 are thought to be acceptable for archaeological material and is the upper limit used in this study. Potential contamination is the primary motivation for sampling compact bone where it is thickest and most dense (anterior femur) and also for the thorough purification of bone samples by removing humic acids and other contaminants. Diagenetic alteration of the naturally occurring balance can result in the misconstruing of data, which for example, can lead to the "mimicking" of trophic level effects (discussed shortly) resulting in the presumption of carnivorous diets when in actuality they are herbivorous (Grupe & Schweissing 2001).

Carbonate

In an early study, Schoeninger and DeNiro (1982) found that the use of the carbonate phase of buried bone will be accompanied by the perennial issue of diagenetic effects such as isotopic exchange with groundwater carbonates, a process that can potentially lead to erroneous interpretations. The soil in the burial environment also contains inorganic sources of carbon that will decompose during the combustion step prior to isotope ratio mass spectrometry (IRMS) analysis, in the process releasing CO_2, which contaminates the organic signal. Others

have warned that bones that have been buried for long periods and/or have lost most or all of their collagen are undoubtedly more susceptible to isotopic exchange of apatite carbonate (Lee-Thorp & van der Merve 1987, 1991, Sillen 1998, Koch et al. 1990, Quade et al. 1995). In comparison to bone, tooth enamel is far less susceptible to diagenetic alteration, an observation apparently rooted in the tooth's more protective structure (Lee-Thorp & van der Merwe 1987, 1991, Koch et al. 1990). Nonetheless, a careful selection of well preserved specimens is necessary to produce data reliable for a dietary reconstruction using apatite carbonate. When diagenetically adsorbed carbonates are removed post mortem by pretreatment of tooth enamel with dilute acetic acid (Lee-Thorp et al. 1989a, Krueger 1991) it has even proven possible to obtain dietary signatures for ancient material originating from Permian, Paleocene/Eocene, Miocene and Plio-Pleistocene sites (Ericson et al. 1981, Lee-Thorp & van der Merwe 1987, 1991, Lee-Thorp et al. 1989b, Thackeray et al. 1990, Koch et al. 1992, Quade et al. 1992). In a study focusing on oxygen isotopic signatures extracted from carbonate, Zazzo et al. (2004) found evidence for selective alteration of isotope composition suggesting isotopic exchange processes related to postmortem microbial activity. The action of diagenesis in molecular structure through groundwater interaction with calcified tissues also resulted in isotope composition variations of up to 3‰. The concern is not so much that diagenetic processes, which are ubiquitous, are at work changing compositions, but that they go unnoticed, and biogenic signals become confused with diagenetic ones. Trueman et al. (2003) concluded from a study on fossil dinosaur bones that diagenetic recrystallization tends to homogenize the isotopic composition of bone apatite, rendering inferences based upon it for palaeoclimatic reconstruction unreliable. In an excellent summary of the past fears concerning diagenesis and the subsequent suggestions made to alleviate the problems involved with this process, Lee-Thorp (2002) indicates that oxygen derived from carbonate was thought inferior to that extracted from phosphate, since the P-O bond, which is formed in vivo by enzymatic catalysis, is exceptionally strong compared to the C-O bond and unlikely to experience oxygen exchange with water. It was, however, later shown that a strong correlation existed between oxygen isotope ratios derived from phosphate and those originating from structural carbonate, the latter of which is approximately 8.7‰ more positive in bone unaffected by diagenesis (Bryant et al. 1996).

7 Literature review - stable isotope analyses in anthropology

7.1 Stable isotopes in palaeodietary reconstructions

The scientific examination of isotopes was traditionally a research cornerstone in physics, biochemistry and geology and only relatively recently been associated with the fields of archeology and anthropology. Atoms of elements, such as carbon or nitrogen, can assume different physical forms called isotopes, in which the number of protons in the atoms nucleus is the same but the number of neutrons varies, resulting in slightly different atomic weights between each isotope. According to Hoefs (1997), isotopes are divided into two fundamental groups, those that are stable and those that are radioactive or unstable. Presently, about 300 stable isotopes are known to exist, unstable isotopes on the other hand number more than 1200.

Isotope values can be measured in the tissues of all plants and animals. They are passed along the food chain from plants to animals, and therefore from our food into our body. The isotope values in human body tissues reflect to a large extent a mixture of all of the isotope values of all of the food eaten and fluid drunk. Because different types of foods have distinctive isotope signatures, measurements of the isotopic values of human bone allow assumptions to be made about the foods eaten by that individual. This type of analysis has the potential to provide fundamental dietary information such as the sorts of plants consumed, degree of carnivory, and helps in establishing the trophic level position of the individual being analyzed in the overall food chain.

Stable isotope studies of palaeodiet are based on the observation that the stable carbon and nitrogen isotopes of an organism appear to be maintained in its bone following death (DeNiro & Epstein 1978a, 1981), and that the stable isotopes of carbon and nitrogen in bone collagen from osteological remains provide direct information on the lifetime diets of past populations (Vogel & van der Merwe 1977). The earliest mention of the potential use of isotopes for dietary reconstruction appears in an unpublished report by Robert Hall in 1967 titled "More About Corn, Cahokia and Carbon-14". Initial applications of stable isotope analysis to human dietary research utilized stable carbon isotopes and focused on the timing of the introduction of maize agriculture to various regions throughout North America. These examinations included the analysis of isotopic compositions of human bones from archaeological sites in Ohio, Illinois, New York, and West Virginia that revealed a gradual shift in diet from the Late Archaic period (around 2000 BC) to the Upper Mississippian period (around 1300 AD). This method has been widely applied in archaeology and physical anthropology since its introduction in 1977 in Europe, North America and South Africa. Extensive reviews of stable

isotope palaeodietary research have been presented by (Hare & Estep 1983, Haynes 1968, Koch 1998, Dorozynski & Anderson 1991, Katzenberg 1992, Keegan & DeNiro 1988, Ambrose 1987, 1993, DeNiro 1987, Norr 1995, Schoeninger & Moore 1992, Pate 1994, Schwarcz & Schoeninger 1991, Grupe 1987, van der Merwe 1982). Similar analysis also focused on the individual's diet over a lifetime (Renfrew & Bahn 1996) and others indicate that stable isotope analysis of carbon and nitrogen from collagen in human bone can also be utilized to determine what the relative proportions were of various foods present in the diet (Chisolm 1989). This method along with other indirect measures of human diet such as archeological artifacts, and faunal, botanical and skeletal analysis provides a much clearer picture of subsistence practices than previously possible.

Since the incorporation of stable isotope analysis in the arsenal of tools for clarifying questions in the archaeological context, a mass of experimental field studies far too vast to list in its entirety have documented the strong correlation between stable isotope ratios of nitrogen $^{15}N/^{14}N$, carbon $^{13}C/^{12}C$ and sulfur $^{34}S/^{32}S$ in vertebrates and that of their diets including (e.g. Bocherens et al. 2001, Richards et. al. 2000, 1998, Pingitore 2000, Richards & Hedges 1999a, 1999b, Richards 1996, Baraybar 1999, 1997, Grupe 1998, 1987, White 1998, McGovern & Quinn 1996, Sillen et al. 1998, Ambrose et. al. 1997, Cox & Sealy 1997, Mays 1997, Sillen & Lee-Thorp 1994, Ambrose & Norr 1993, Tieszen & Fagre 1993, Matson & Chisholm 1991, Ambrose 1991, 1987, 1986, Lee-Thorp et al. 1989a, 1989b, Schwarcz et al. 1985, Bender et al. 1981, Norr 1981). Other studies have shown that the ratio of ^{13}C to ^{12}C can be used to provide dietary information about extinct fauna (Lee-Thorp et al. 1994). The importance of isotopic investigations for hominid evolution was indicated by the analysis of *Australopithecus africanus* by Sponheimer and Lee-Thorp (1999a), who found that early hominids ate not only fruits and leaves but also large quantities of ^{13}C enriched foods such as grasses and sedges or animals that ate these plants, or both. The results suggest that early hominids regularly moved in open environments such as woodlands and grasslands for food. Lillie & Richards (2000) identifying subsistence strategies in Stone Age man by utilizing stable isotope analysis, and determined a fisher-hunter-gatherer diet of Mesolithic-Neolithic transition in the Ukraine. These and other studies like Ambrose and DeNiro (1989) also point out the important role environmental influences play in determining isotope compositions.

The great advantage of stable isotope analysis is that it gives a direct measure of long term human diets on the individual level, whereas more traditional methods of diet reconstruction from environmental archaeology, such as floral and faunal analyses, give information about specific foods eaten, and may only reflect single or special meals. The majority of dietary

remnants originating from archaeological sites also tend to overemphasize faunal remains, particularly those with better taphonomic survivability, for example, long bone fragments from large mammals versus fish and bird skeletal elements. Palaeobotanical remains, or indicators for their presence such as phytolith assemblages (Eichhorn 2006), are only rarely preserved, sometimes being recovered from ceramic vessels, in the form of seeds or pollen in textiles or in graves that have survived the ages, or occasionally in the digestive tract of mummified humans. Other forms of dietary analysis involved the interpretation of skeletal pathologies (Marin et al. 1985) or dental microwear characteristics (Fine & Craig 1981). These too have their drawbacks, because they may simply be indicators of diet preferences over a brief time span.

There remains, however, a definite parallel between the interests of stable isotope researchers and those exploring past human diets by different means. Together with novel advances in technology and analytical methods, stable-isotope biochemistry is now frequently employed as a routine procedure at many institutions and progressively becoming an integral part of research investigations within the anthropological field. Presently, stable isotope ratios are even accessible online, allowing for quick and easy comparisons, an option facilitating the rapid, simple and efficient exchange of information so vital to any science (see OIPC, section 7.7).

7.2 Stable isotopes of carbon and nitrogen in collagen

Though isotope analysis can be used to look at a number of elements in the diet, most researchers have focused their efforts on the presence of stable isotopes of the light elements carbon and nitrogen in bone collagen. This method of carbon and nitrogen isotope analysis is based on the observation that these signatures are transferred along food webs in predictable ways. The ratio between the two stable isotopes of carbon of interest, ^{13}C and ^{12}C, is referred to as the $\delta^{13}C$ value. For nitrogen, the two isotopes of interest are ^{15}N and ^{14}N, where the ratio is referred to as the $\delta^{15}N$ value. The $\delta^{13}C$ and $\delta^{15}N$ isotopic compositions of a sample are measured as the ratio of one isotope to another. Delta values (δ) are expressed in parts per thousand (‰) relative to an international standard.

$$\delta^{13}C = \frac{(^{13}C/^{12}C\ \text{sample} - {}^{13}C/^{12}C\ \text{standard})}{(^{13}C/^{12}C\ \text{standard})} \times 1000$$

$$\delta^{15}N = \frac{(^{15}N/^{14}N \text{ sample} - ^{15}N/^{14}N \text{ standard})}{(^{15}N/^{14}N \text{ standard})} \times 1000$$

The ratio of these isotopes ($^{13}C/^{12}C$ and $^{15}N/^{14}N$) in bone collagen in turn reflects the approximate ratios these isotopes are found in the diet and in the environment of that particular individual.

Bone carbon values are compared to the universally accepted standard Pee Dee Belemnite (PDB) and bone nitrogen values are compared to the atmospheric nitrogen standard ambient inhalable reservoir (AIR). A substance with an isotope ratio less than that of the standard will have a negative δ value, and is said to be depleted relative to the standard. A substance that is enriched relative to the standard will have a positive δ value.

The basic building blocks that have been used to make all body tissues, including bones, have been taken from foods one eats over a lifetime. More specifically, the elements carbon and nitrogen in bone are essentially the same carbon and nitrogen atoms contained in the foods we eat, and since the $\delta^{13}C$ and $\delta^{15}N$ values of various foods have been fairly well documented it is possible to establish a correlation between nutrition and organism. The physiological process by which C and N molecules are broken down and absorbed during digestion and metabolism from consumed foods is accompanied by the deposition of some of those same atoms into bone during tissue synthesis. Therefore, the ratios between the isotopes of C and N respectively mirror the dietary intake to a certain extent. If the $\delta^{13}C$ and $\delta^{15}N$ values are considered signatures specific to different types of foods, and the change that occurs in these values when these C and N atoms are deposited in our bones is known, then ascertaining the $\delta^{13}C$ and $\delta^{15}N$ values of an organism's bone will allow inferences regarding what kinds of foods the bone C and N came from.

Experimental data from Ambrose (1986, 1993, 2000) indicated that bone tissues do indeed reflect different components of the diet. Bone collagen is disproportionately produced from the protein portion of the diet. Ambrose et al. (1997) showed in analyzing the diet of peoples in the Marianas Archipelago that collagen carbon isotopes mainly reflect those of dietary protein sources and thus potentially overestimate the contribution of marine-animal foods. Stable isotope analysis of both bone collagen and apatite permit a quantitative estimate of several dietary components that reflect whole diet. Because both bone collagen and bone apatite are constantly undergoing a process of resorption and subsequent replenishment, their respective isotopic compositions reflect dietary averages over at least the last several years of

an individual's life (Bell et al. 2001). For example, in adults, the average rate of bone remodeling in adult humans is about 7-11 years, which also translates to the approximate time required for all of the collagen in a long bone such as the femur to be replaced entirely (Parfitt 1983). Therefore, adult human bone collagen provides an average record of all of the protein that has been eaten by a person over approximately a ten-year period. Short term or seasonal changes in diet are therefore difficult but not impossible to detect.

7.3 Stable isotopes of carbon in structural carbonate

As described previously in section 4, the mineral in bone is primarily crystalline calcium hydroxyapatite ($Ca_{10}[PO_4]_6[OH]_2$), a calcium-hydroxyphosphate composite called bioapatite. This bioapatite in turn contains a certain amount of carbonate, approximately 2-5% according to Chickerur (1980), that substitutes for hydroxide (Type A carbonate) and phosphate (Type B carbonate) in the crystal latticework, and can also have adsorbed or labile carbonate on its surface structure (Boskey 1999). Bioapatite, with carbon from carbonate, is a major component of the mineral phase of calcified tissues. This "structural" carbonate originates from blood bicarbonate, therefore, bioapatite $\delta^{13}C$ values also derive from foods the person or animal has eaten that were metabolized as fuel and represent a mixture of dietary protein, carbohydrates and fats.

The usefulness of isotopic analysis of carbonate in bone and also tooth bioapatite for dietary reconstruction was first demonstrated by DeNiro & Epstein (1978b). Sullivan & Krueger (1981) and Krueger & Sullivan (1984) went on to show that this phase of bone could be used for reconstructing prehistoric diet. Ericson et al. (1989) and Lee-Thorp et al. (1989a) both illustrated the utility of apatite to study the diets of animal and human populations. Collagen has a definite chronological expiration date and the majority of collagen is fully decomposed by various taphonomic or diagenetic factors after 10,000 years. Carbonate on the other hand, can preserve dietary isotopic signatures for several million years, making it especially attractive for investigations concentrating on ancient man or ancestral man. Aspects of the palaeoflora as well as palaeodiets of Pliocene and Pleistocene hominids and other mammals could therefore be studied (Ericson et al. 1981, Lee-Thorp 1989, Lee-Thorp et al. 1989b, 1997, Ambrose 1998a, 1998b, Sponheimer and Lee-Thorp 1999a, Cerling et al. 1997, Budd et al. 2000).

Researchers such as Schoeninger and DeNiro (1982) who voiced their skepticism about such analyses warned that postmortem contamination could affect the reliability of results, particularly those focusing on prehistoric remains. The use of structural carbonate in

palaeodietary studies has therefore been accompanied by the requisite development of more refined methods for collecting it from bioapatite and separating it from adsorbed exogenous carbonate (from the surrounding soil) in archaeological bones. Structural carbonate has also been used for radiocarbon dating unburnt bone on a limited scale. Recent efforts involve recovering carbonate from cremated bone for use in carbon and oxygen stable isotope studies (Lanting et al. 2001).

Carbon spacing

A number of authors have addressed the utility of determining the difference between carbon ratios originating from collagen and structural carbonate (Krueger & Sullivan 1984, Lee-Thorp et al. 1989a, Hedges 2003). The difference arises from the variable fractionation taking place between the various dietary components consisting of proteins, fats and sugars, and more specifically, because collagen ratios are representative of the protein portion of what is consumed and carbonate ratios originate from all components of the diet. The difference in carbon values, which is known as carbon spacing, basically represents the different fractionation factors that exist between macronutrients, e.g. meats (3‰) and lipids (-2‰), and results from the variable incorporation of these different macronutrients within individual diets (Bowden & Tieszen 1999). Based upon a South African ecosystem, Lee-Thorp et al. (1989a) indicated that carnivores have $\Delta_{carb\text{-}coll}$ that average about 4 ± 1.5‰, herbivore spacing lies at approximately 7 ± 1‰, and omnivores exhibit a $\Delta_{carb\text{-}coll}$ characteristic of a mixed diet. Complications can arise in animals feeding on a combination of C_3 and C_4 plants, however, this aspect is irrelevant for the Volders situation. The ratios acquired in this study originate from a temperate climate with primarily C_3 vegetation, therefore, it can be expected that the spacing differences will be slightly higher. The data must be considered within the ecosystem from which they originate. Once this is calibrated, it is the comparison within the group that is of value. The premise remains the same, individuals exhibiting $\Delta_{carb\text{-}coll}$ values that are lower than other members of the group have diets more heavily based on animal proteins and fats than those with larger spacing between ratios.

7.4 Carbon fractionation

Isotope fractionation is a partial separation of isotopes of the same element during physical (e.g., evaporation) or chemical (e.g., precipitation) processes. Physical processes are accompanied by a change in the physical state (gas, liquid and solid phases). Chemical processes involve the coupling of atoms with other atoms. Because of mass differences and

varying bonding strengths, isotopes can undergo two different types of fractionation, *kinetic isotope fractionation*, which is characterized by rate of reaction differences where molecules of lighter isotopes move more rapidly; and *equilibrium isotope fractionations* in which the thermodynamic properties of molecules with different isotopes affect reactions. Fractionation can be viewed as a metabolic bias and involves the preferential incorporation or exclusion of one isotope versus the other in the product of a chemical reaction (Price 1989). For carbon, variable fractionation takes place during photosynthesis and results in its incorporation into plant tissues. The carbon plants use in photosynthesis comes from atmospheric carbon dioxide. Carbon found in the atmosphere as CO_2 has a constant $^{13}C/^{12}C$ ratio of approximately 1:99. During the uptake and conversion of CO_2 into plant carbon, isotopes are fractionated by natural photosynthetic accumulation of carbon dioxide. It is this isotopic fractionation that influences the carbon isotope ratios characteristic for different terrestrial plants (Chisolm 1989).

Photosynthesis

Two main photosynthetic pathways discriminate more or less against the carbon isotope ^{13}C, a third form known as crassulacean acid metabolism (CAM), used by stem succulents like cacti, is not discussed since the plants utilizing this form are not all too relevant for human consumption. Grasses from hot, arid environments follow the C_4 (Hatch-Slack) photosynthetic pathway and exhibit $\delta^{13}C$ values averaging about -12.5‰, and trees, shrubs, flowering plants, and grasses from temperate regions, which follow the C_3 (Calvin-Benson) photosynthetic pathway, have $\delta^{13}C$ values that average about -26.5‰ (Bender 1968, Vogel & van der Merwe 1977, Vogel 1980, van der Merwe 1982, Matson & Chisholm 1991). With a few exceptions, most terrestrial plants are C_3 plants. It is therefore also assumed that all of the plants involved in the Volders area food chain were also C_3 plants. In some forested areas, a canopy environment generates the so-called "Baldachin effect", which occurs due to incomplete atmospheric mixing, resulting in even more negative carbon isotope ratios (van der Merwe & Medina 1991). Very negative values are therefore indicative of heavily forested areas. Although the C_3 plants $\delta^{13}C$ average is approximately -26.5‰, its range can vary from -37‰ under this closed forest canopy condition to more positive values reaching -22‰ under water stress. C_4 plants exhibit less variability because they do not exist under canopies and are better adapted to arid conditions and their range is generally from -13‰ to -10‰.

Fractionation of carbon in plants is also dependent on how specific types of plants metabolize their nutrients. For example, the aforementioned C_4 plants, which include maize, cane sugar,

some types of millet, sorghums, amaranths and chenopods, all use a 4-Carbon photosynthetic pathway and fix carbon dioxide as a C-4 carboxylic acid, and discriminate less against the heavy ^{13}C isotope than C_3 plants do, resulting in the uptake of more ^{13}C isotope during metabolism. This causes C_4 plants to have a higher (less depleted) $^{13}C/^{12}C$ ratio than observed for C_3 plants (O'Leary 1981, 1988, DeNiro et. al. 1985). Logically, organisms that consume C_4 plants have characteristically higher $^{13}C/^{12}C$ ratios in their tissues than those that rely primarily on C_3 plants for consumption. The different carbon ratios unique to certain plant types are passed along the food chain when they are eaten by animals and are incorporated into the organisms bone tissue. The process of fractionation is not restricted to this atmosphere-plant phase but continues on after animals eat the plants.

A large proportion of carbon in the biosphere is fractionated by the plant kingdom. As mentioned, atmospheric carbon dioxide has a stable ratio of ^{13}C to ^{12}C; therefore, plants have a stable ratio that is incorporated into their tissues. These plants are then digested by animals, humans or otherwise, a percent of this is then used by the animal for tissue synthesis. If a human eats herbivore meat they will then incorporate some of the ^{12}C and ^{13}C into their tissues, producing another ratio. Since C_3 and C_4 plants comprise a fundamental part of the human diet, are found in differing ecological surroundings, and possess different energy cycles that ultimately result in the assimilation of differing levels of carbon isotopes by way of fractionation, they provide the ideal medium for recognizing different human diets. The large difference in $\delta^{13}C$ values between the two plant types is retained after its incorporation into the collagen molecular composition of the consumer, and permits the identification of diets based on C_3 or C_4 plants. The original values are altered only by the loss of some ^{13}C during fractionation at each trophic level along the food chain. The process of discerning between C_3 and C_4 diets is of great interest to researchers focusing on subjects like the transition from hunter-gatherer subsistence to agriculturalism, the reliance upon maize by Native American Indians, and especially specific topics such as the food preferences of man's Australopithecine and Homo ancestors.

These isotopic fractionation differences are transferred with an enrichment factor of about +5‰ into consumer collagen and about +12‰ into consumer mineral carbonate (Schoeninger & DeNiro 1984). Animals that eat C_3 vegetation (including fruits, leaves, and the roots of trees, bushes, and forbs) have bone carbonate $\delta^{13}C$ values between about -10 and -16‰ and collagen values at approximately -21‰. Animals that eat C_4 tropical grasses (including blades, seeds and roots) have bone carbonate $\delta^{13}C$ values between 2 and –2‰ and collagen

values of around -8‰, and mixed feeders that eat both fall somewhere in between these two extremes. Carnivores have $\delta^{13}C$ values similar to those of their prey (Lee-Thorp et al.1997).
To better illustrate the diet to organism isotope value relationship and determine the increments that exist between the different diets discussed and consumer bone collagen, called the "collagen enrichment factor", Chisholm (1989) constructed a basic table of values for the trophic levels of various food chains (Tab.1). It is important to remember that the isotopic ratios in consumables and consumers may be altered due to a number of factors. Gannes et al. (1997) indicated that stable isotope ratios at the individual level can be influenced by a combination of ecological, biochemical and physiological processes. Changes in atmospheric carbon reservoirs, differences in metabolic rate or intrinsic assimilation efficiencies can all potentially lead to individuals subsisting on similar diets displaying different isotopic ratios. The following table indicates average ratios for the various diets listed.

Table 1. Carbon isotopes ratio breakdown spanning from plants to carnivores showing the +5‰ collagen enrichment factor (Chisholm 1989).

Diet	Average Carbon Isotopic Ratio	Expected Consumer Ratio
C_3 plants only	-26.5‰	-21.5‰
C_3 herbivore meat	-25.5‰	-20.5‰
C_4 plants only	-12.5‰	-7.5‰
C_4 herbivore meat	-11.5‰	-6.5‰
Marine plankton only	-19.5‰	-14.5‰
Marine herbivores meat	-18.5‰	-13.5‰
Marine carnivores meat	-17.5‰	-12.5‰

By using this standard, the approximate combination of the above categories or of these categories the diet of a given human fits. Ideally, if an organism consumes both C_4 and C_3 species in its diet, the ^{13}C value for its bone collagen will lie between the extremes of -21.5‰ and -7.5‰ (c. -14‰). This will result in an estimate for the relative amounts of C_3 and C_4 plant species of that particular consumer's diet. Similar evidence for a mixed diet can be observed if the $^{13}C/^{12}C$ ratio for a human is found to be in between C_3 diet and the diet of meat from C_3 herbivores, which suggests an omnivorous diet. However, the problem is that

this estimation does not give precise information regarding emphasis on particular consumables and attempts to discern the contributions different components of a diet make based upon isotopic ratios have proved complicated. In a controlled feeding experiment conducted to test the linear mixing model's efficaciousness compared with the Euclidian-distance-based model for determining dietary components according to stable isotopic ratios of carbon and nitrogen, Ben-David and Schell (2001) examined seven captive, adult mink (*Mustela vison*) and concluded that both models provide inaccurate estimates of proportions of food in the diet. Others have been able to pinpoint contributions of specific food types but not the amounts of these dietary components, for example, Hiraguchi et al. (1991), who studied the Mawaki site in Japan, found that the ^{13}C values for human bone collagen from these sites were correlated with two food chains, terrestrial plants with lower, more depleted values and marine phytoplankton with higher values.

Isotope ratio variations

Numerous extraneous influences can alter isotope ratios including those of carbon, for example, it is important to note whether the bones destined for analysis date from a time previous to the 1850s because the atmospheric carbon dioxide content was relatively stable until the industrial revolution beginning around this period, when it started to steadily increase, resulting in a phenomenon known as the "fossil fuel effect" that skews values by approximately 1.6‰ (Marino & McElroy 1991).

Physiologically generated aberrations are also sometimes observable, and an organism's physiology is capable of exerting a powerful impact on the isotope ratios observed in bone and teeth. Physiological effects are species specific, yet can also exhibit individual variances. Hedges (2003) observed systemic spacing in the carbon isotopic composition of bone collagen and bone apatite carbonate in a comparison between herbivores, omnivores and carnivores, which might result not only from diet but also from physiological differences such as methanogenesis, especially in ruminants. This leads to isotopically enriched apatite carbonate in ruminants because the lighter isotope is preferentially passed as gas. In addition, considerable variation has been reported not only between different animal types, but also within a single trophic level. Different physiological pathways have been identified as the origin for such differences, which react to environmental factors including changes in aridity, salinity, fire regime, and nitrogen availability (Sponheimer et al. 2003). Van Klinken et al. (2000) concluded that carbon isotope variations result primarily from climate differences and

that nitrogen variations result from variable meat consumption and the poorly understood variability in nitrogen values of plants.

Intrinsic physiological processes such as bone remodeling can also have an influence on isotopic compositions. Bone specimens from different anatomical sites can show considerable variations even within a single individual, primarily due to different metabolic turnover rates in compact and trabecular bone (Grupe 1988a). Even though experience harvested from trace element research indicates that isotope composition in a compact bone sample is well correlated with the total skeletal content of the respective element, this observation suggests the use of compact bone from corresponding anatomical sites for analytic purposes to increase the homogeneity of the sample site and reduce the risk of physiologically generated variations, a methodological approach used in the Volders study (see Methods section).

Variations in isotope values can also result from differing or ineffective collagen extraction methods. Garvie-Lok et al. (2004) examined the effects of treatment time and acid concentrations in the preparation of bone carbonate for stable isotope analysis. It was found that 4 hours of treatment were sufficient to remove highly soluble contaminants and that longer solubilization times can lead to sample recrystallization. A more dilute acid causes smaller shifts in ^{13}C and ^{18}O. It is also possible for collagen preservation itself to vary widely even within single archaeological sites (Ambrose & DeNiro 1989, Norr 1991, Tuross et al. 1988, Schoeninger 1989a). It is therefore important to provide quantitative data on collagen composition in order to permit independent assessment of the quality of the data. Comparative studies of stable isotope ratios of humans between regions, or within regions through time, must consider the systematic variations in the isotopic composition of foodwebs due to climate, habitat, nutrition and animal physiology. In addition, standardization of methods of purification and preparation of tissues for isotopic analysis, and of characterization of the elemental composition of purified residues analyzed are strongly recommended in order to facilitate comparisons (Chisholm 1989).

7.5 Stable isotopes of nitrogen in bone collagen and nitrogen fractionation

A second element important for palaeodietary studies is nitrogen. Nitrogen has two stable isotopes, ^{14}N and ^{15}N and nitrogen as an element, is found primarily in the atmosphere or in ocean water. Early studies demonstrated the relationship between stable nitrogen isotope ratios exhibited in animal tissues such as bone and those observed in the diet (DeNiro & Epstein 1981, Hare et al. 1991). In the early 1990's, technological advancements in mass spectrometry made the measurement of the $\delta^{15}N$ value much easier. This technological

advance has been accompanied by a large increase in the number of stable isotope applications, and $\delta^{15}N$ values have provided new information on the amounts of animal vs. plant protein in past diets. Even the source of protein was occasionally attributable to specific animals. Examples of these applications included demonstrating that Neanderthals in Belgium and France had diets dominated by animal protein (Bocherens et al. 1999), and that Upper Paleolithic humans at Gough's Cave in the U.K. showed a similar nutritional trend (Richards et al. 2000).

Like carbon, the nitrogen isotope value of an organism depends on the values of its nitrogen sources and on the metabolic effects within the organism itself (van Klinken et al. 2000). Various organisms frequently exhibit differences in their metabolic processes that potentially result in substantial variations in the mean $\delta^{15}N$ values. The precise magnitude of the isotopic difference between diet and a particular tissue depends on the extent to which the heavy isotope is incorporated or lost during synthesis. In contrast to carbon and sulfur, nitrogen shows a 3-4‰ enrichment in $\delta^{15}N$ in muscle tissue, bone collagen or the whole organism relative to the food source (DeNiro & Epstein 1981, Minigawa & Wada 1984, Schoeninger & DeNiro 1984). When this fractionation is taken into account nitrogen isotopes can function as a good indicator for dietary protein source. This incremental increase in $\delta^{15}N$ value, referred to as the "trophic level effect", results in distinct, chemically recognizable trophic levels created by the passage of nitrogen through the food chain from environment to plants to herbivores to carnivores, and the characteristic step-wise enrichment of the isotope in each of the respective groups. The systematic enrichment of nitrogen isotope ratios observed between trophic levels not only in mammals but also birds and fish, is thought to be associated with the discrimination against isotopically heavy urea at renal membrane boundaries during transamination or deamination of amino nitrogen during urea synthesis, which produces an endogenous nitrogen pool approximately 4‰ heavier than dietary sources (Coltrain et al. 2004, Schoeller 1999). This 3-4‰ $\delta^{15}N$ shift occurring with each trophic level provides a basis for establishing trophic structure within a group, population or ecosystem (Fig. 7.1). As such, nitrogen has been regularly used to distinguish between terrestrial and aquatic, and carnivorous and herbivorous diets. This observation underlines the importance of establishing an isotopic composition base for the ecosystem involved in the study, which is in part accomplished by analyzing the faunal remains recovered at a given archaeological site, and facilitates the placement of the test subject within a trophic framework composed of the flora and fauna of his or her environment. In this way the isotope ratios acquired from the Volders

skeletal material could be more accurately interpreted within the ecosystem particular for that series.

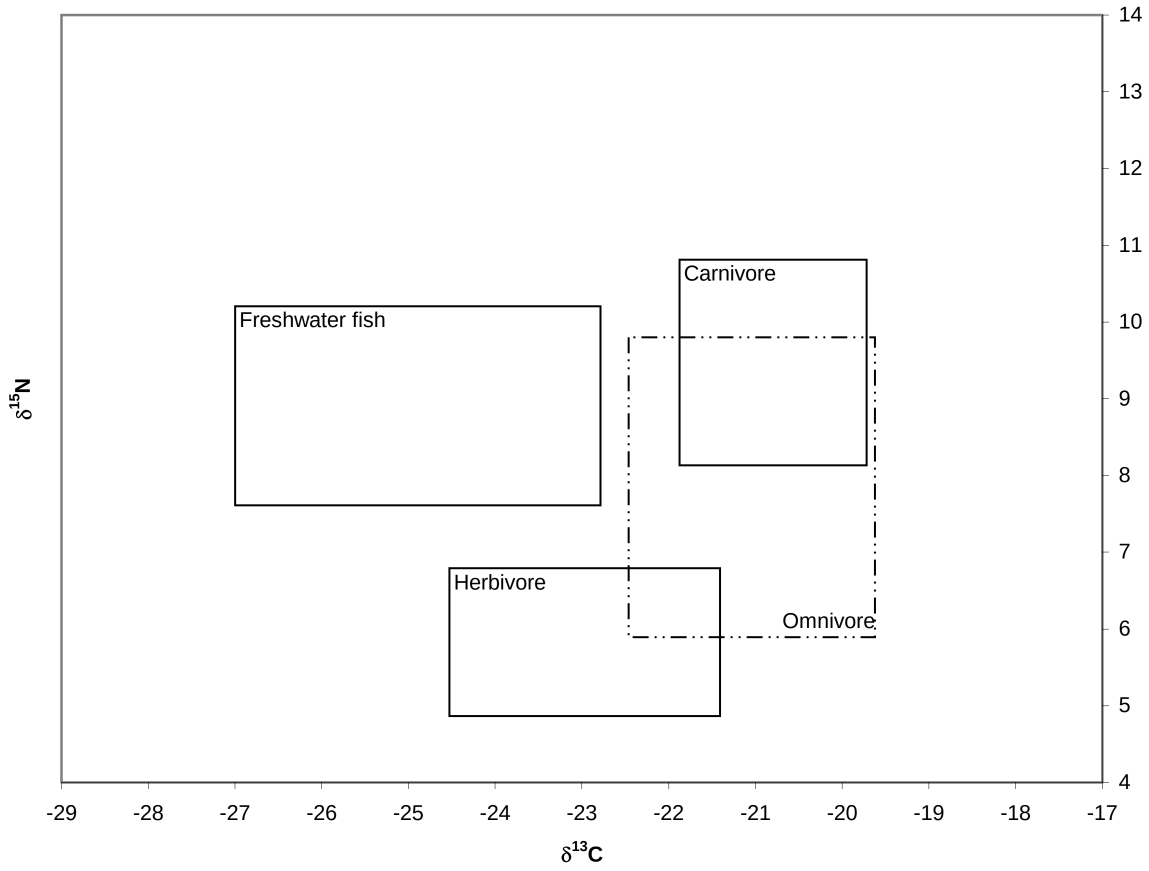

Figure 7.1. Illustration of the trophic level effect showing the step-wise enrichment in $\delta^{15}N$ from herbivore to omnivore to carnivore in a C_3 biome (after Grupe & Mekota 2005).

Using stable nitrogen isotopes to clarify questions pertaining to diet is not always straightforward. While most plants follow either the C_3 or C_4 photosynthetic pathway and have similar carbon isotope ratios in most ecological settings, nitrogen isotope ratios vary according to rainfall, altitude and other factors (Ambrose 1991). Nitrogen isotope ratios can vary considerably among aquatic organisms and are also very climate sensitive. The influences of climate are well illustrated by soil analysis, which show that cool forest soils have lower $\delta^{15}N$ values compared to hot, arid desert or savannah soils (Schoeninger & DeNiro 1984). Several researchers noted early on that complexities relating to climate, environment and physiology were mirrored in the observed nitrogen isotope variations within trophic

levels (Ambrose 1991, Ambrose & DeNiro 1989, Heaton et al. 1986, Sealy et al. 1987). Since nitrogen isotope ratios in human bones may be affected by climate and physiology they cannot be directly compared between different types of ecosystems without first determining the isotope composition of the local foodweb and the stepwise enrichment between trophic levels (Heaton 1986, Heaton et al. 1986).

Three major categories of plants and animals, in terms of isotope analysis, can be discerned using nitrogen isotopes: nitrogen-fixing plants and those animals that consume them, terrestrial food chains not involved in nitrogen fixation, and marine foods not based on nitrogen fixation. Using nitrogen isotope analysis, it is possible to distinguish which group's diets are based on leguminous, terrestrial, or marine foods (Price et al. 1985). One of the first studies involving nitrogen isotopes in the anthropological context was in the identification of legumes in terrestrial diets (DeNiro & Epstein 1981). Nitrogen was useful because legumes have a lower ^{15}N to ^{14}N ratio due to their process of nitrogen fixation. Nitrogen isotope ratios for plants depend primarily on how they obtain their nitrogen, either by symbiotic bacterial fixation or directly from soil nitrates. Leguminous plants fix atmospheric nitrogen by way of bacteria and are not reliant on the soil to provide nitrogen for fixation, and therefore incorporate a lower ratio of the isotopes during this process. Stable nitrogen isotope ratios were also used by DeNiro (1987) and Keegan (1989) to distinguish between diets composed of leguminous, all of which are C_3 based, and non-leguminous plants, which utilize both the C_3 and C_4 pathway.

A major advance in stable isotope analysis in bioarchaeology was the demonstration of a clear differentiation between carnivore and herbivore trophic levels with the aid of nitrogen isotope ratios (Minigawa & Wada 1984, Schoeninger 1985, Katzenberg 1989, Bocherens et al. 1991). It has thus been possible to evaluate the degree of human carnivory or consumption of animal protein with bone collagen nitrogen isotope ratios (Ambrose & DeNiro 1986, Schoeninger 1989b). In a notable study on the ancient Maya diet, Reed (1998) found that the Copan inhabitants based their diet on terrestrial plant sources with maize being the dominant staple. The low $\delta^{15}N$ values clearly indicated that deer and other meat sources were only occasionally utilized.

Weaning

A number of studies have indicated that nursing infants are effectively carnivores relative to their mothers whose milk is enriched in nitrogen relative to their own body levels, which allows nitrogen isotopes to also be used to document age at weaning (Fogel et al. 1989,

Schurr 1997, Katzenberg & Pfeiffer 1995). Richards et al. (2002), measured $\delta^{15}N$ ratios of bones with a high turnover rate, such as a rib, and noted that it is possible to observe when weaning generally occurred in a past population. Levels of bone ^{15}N offer a good indicator of breastfeeding since human milk is enriched with ^{15}N, and the signature is passed on to the breastfeeding child and subsequently incorporated into its bone. The trophic level difference observed showed a 2-4‰ more positive $\delta^{15}N$ value in nursing human infants than weaned children and adults (Fogel et al. 1989, Katzenberg et al. 1995, Tuross & Fogel 1994, Wright & Schwarcz 1998, Wiedemann et al. 1999). Since morbidity and mortality in children are both related to the cessation of nursing and the switch to adult food (Dittmann & Grupe 2000), a fact which ancient people were no doubt aware of, identifying average age of weaning can provide information important to understanding infant nutrition and parental care. Wright & Schwarcz (1999), for example, found in studying the isotope analysis in Kaminaljuyu children, that many children continued to nurse until a non-infant age long after they had begun to eat solid maize foods. This practice certainly indicates the necessity of breast milk to augment the one-sided diet based on maize with much needed protein.

Since milk products are also enriched in $\delta^{15}N$, high values can be used to indicate the consumption of these foodstuffs. The difference between people dependant upon agriculture compared to those reliant on pastoralism can therefore be discerned (Ambrose 1986). Since at Volders it is assumed that animal husbandry was of greater importance than agriculture, high $\delta^{15}N$ ratios can be expected not only due to meat consumption but also as a result of the availability of dairy products.

7.6 Stable isotopes and diets based on aquatic resources

The ratio of nitrogen isotopes has also proven useful to determine terrestrial or aquatic based protein intake. Aquatic food chains from both marine and freshwater ecosystems tend to be longer than terrestrial ones, and since differences at the bottom of the food chain are passed up to the plant consuming animals higher up in the chain, the $\delta^{15}N$ values of marine organisms tend to be higher than those found in terrestrial settings (Price 1989, Renfrew & Bahn 1996). The bivariate use of nitrogen isotopes in conjunction with carbon provides an effective tool for distinguishing between terrestrial and aquatic food sources when there is overlap of values from different sources. It was demonstrated early that the combination of stable carbon and nitrogen isotopes can be used to make this differentiation in food sources (DeNiro & Epstein 1978a, 1978b, 1981). Thus, human populations that rely on the

consumption of marine and freshwater food sources will exhibit higher $\delta^{15}N$ to $\delta^{14}N$ ratios than they would if their diet was based upon agriculture or terrestrial hunting and gathering.

In an early study, Tauber (1981) quantified marine resource consumption by measuring the $\delta^{13}C$ value of bone collagen extracted from Mesolithic and Neolithic humans in coastal Denmark. He found that Mesolithic individuals had $\delta^{13}C$ values of -12‰ indicative of marine diets. However, following the advent of Neolithic material culture in Denmark, all of the human $\delta^{13}C$ values exhibited differences indicating diets incorporating no marine foods ($\delta^{13}C$ = -20‰). It became clear after Tauber's early work that from the Neolithic onward in Western Europe marine foods no longer comprised a significant part of human diets in prehistory. Following on from Tauber's early work others have attempted to look for changes in diets across the Mesolithic/Neolithic transition in other coastal areas such as Portugal (Lubell et al. 1994). Richards & Hedges (1999b), found an abandonment of marine foods associated with the introduction of Neolithic material culture into Britain.

Because the use of $\delta^{15}N$ and $\delta^{13}C$ ratio differences yielded so much important insight with regards to the assumptions about the diets of people represented in archaeological contexts, especially those in coastal regions, the subject deserves some attention. Marine plants use dissolved bicarbonate rather than atmospheric CO_2 during photosynthesis, and bicarbonate is about 8.5‰ more enriched in ^{13}C than atmospheric CO_2. Marine plants also utilize dissolved nitrate and ammonium and are about 7‰ to 10‰ more enriched in ^{15}N than terrestrial plants. This has consequences for ratio values in food chains incorporating aquatic resources. For example, the $\delta^{15}N$ value for marine animals feeding on fish is about 16.5‰, and for marine animals feeding on invertebrates is 13.3‰ (Schoeninger & DeNiro 1984), which results in the logical observation that marine animals have higher $\delta^{15}N$ and $\delta^{13}C$ than that of marine plants. This difference is passed on to human consumers of marine foods (Norr 1981, Tauber 1981, Chisholm et al. 1982, 1983, Schoeninger et al. 1983, Hobson & Collier 1984). In a recent study, Prowse et al. (2003) utilized isotopes in collagen and bone apatite from individuals buried in the first and third centuries near Rome, Italy and inferred that their diet consisted of marine foods.

Terrestrial herbivores and carnivores have average $\delta^{15}N$ values of 5.3‰ and 8.0‰ respectively and human consumers of terrestrial plants and animals typically have $\delta^{15}N$ values in bone collagen of about 6-10‰ whereas consumers of freshwater or marine fish, seals and sea lions may have $\delta^{15}N$ values of 15-20‰ (Schoeninger et al. 1983). Based on fish specific $\delta^{15}N$ signatures it has also been possible to identify the habitat of fish consumed, and therefore the preferred area for fishing (Keegan & DeNiro 1988). Other researchers have been

able to pinpoint the aquatic foods being exploited in Mesolithic Scotland (Richards & Mellars 1998), as well as the European Atlantic coastal region (Richards & Hedges 1999b). Stable isotope analysis conducted on 27 Greenland Norse people excavated from churchyard burials from the late 10th to middle 15th century revealed that the diet of the Greenland Norse changed dramatically from predominantly terrestrial food around 1000 AD to predominantly marine food toward the end of the settlement period around 1450 AD (Arneborg et al. 1999).

Further efforts have focused on differentiating between the resources exploited by coastal and interior populations. For example, populations subsisting on a marine economy like the Alaskan and Northwest Coast Indians had $\delta^{15}N$ values ranging from 17‰ to 20‰, whereas populations with an agricultural economy (manioc farmers from Columbia, South America; Mesoamerican maize agriculturists, and grain growers from the Neolithic period in Europe had $\delta^{15}N$ values ranging from 6‰ to 12‰. Groups utilizing a combination of aquatic and terrestrial foods had intermediate $\delta^{15}N$ values (Schoeninger et al. 1983). Newsome et al. (2004) looked at carbon and nitrogen ratios of Holocene period human burials in California and determined main food sources were derived from multiple sources. The reconstruction of diets in tropical settings where both aquatic and terrestrial resources were exploited is also the object of extensive study (Schoeninger et al. 1983, Walker & DeNiro 1986, Sealy & van der Merwe 1988, Keegan & DeNiro 1988, Norr 1995).

The likelihood that marine resources were consumed at Volders is minimal due to the great distance from the nearest sea, however, the probability that freshwater fish from the Inn River were used as a food source, a river which is in close proximity to the archaeological site, is comparatively high. There still remains the possibility that dried saltwater fish products were imported or that individuals from coastal areas migrated to the Volders area along the Roman road through the Brenner Pass. A number of studies have used the differences observed in isotopic signatures that result from fractionation characteristics unique to marine, freshwater and terrestrial habitats, in order to identify diets based on resources originating from these individual systems or a mixed diet of aquatic and land based foods. Bonsall et al. (1997) provided direct evidence for the importance of freshwater fish to the Mesolithic inhabitants of the Iron Gates region of Southeast Europe, and Green (1998), in a study of a palaeoindian woman from Southern Idaho, examined carbon and nitrogen isotopes and discovered a $\delta^{13}C$ value of -19.5‰ and a $\delta^{15}N$ value of 15.5‰. The $\delta^{13}C$ value of -19.5‰ indicates a dependence on terrestrial or aquatic resources with supplementation from anadromous fish. The highly positive $\delta^{15}N$ value of 15.5‰ indicated a diet of meat and fish, comprising animals most likely at the end of a long food chain. Schoeninger and DeNiro (1984) showed

that carnivorous freshwater fish have average $\delta^{15}N$ ratios of about 8‰, which is very similar to that exhibited by terrestrial carnivores, and Peterson and Fry (1987) showed that freshwater fish $\delta^{13}C$ values are very similar to terrestrial carbon ratios. An examination of the nitrogen and carbon ratios in European freshwater fish originating from Neolithic Bavaria, Germany indicates that the ratios of species including catfish, pike, barbel, carp, chub and other cyprinoids range from 8-10.5‰ for $\delta^{15}N$ and -23 to-27‰ for $\delta^{13}C$ (Asam et al. 2004). Should any of the Volders inhabitants have relied on freshwater resources, the resulting isotopic ratios incorporated into their bone will differentiate them from those individuals that sustained themselves on agricultural products including herbivore meat based upon their $\delta^{13}C$ ratios.

7.7 Stable isotopes of oxygen

Oxygen is the most abundant element on earth, and occurs in gaseous, liquid and solid compounds, most of which are thermally stable over large temperature ranges, a fact that makes oxygen one of the most interesting elements in isotope geochemistry (Hoefs 1997) Oxygen occurs in nature as three common stable isotopes, each with 8 protons. ^{16}O, which is the most abundant comprises 99.765% of all oxygen atoms on earth, ^{18}O (0.1995%), and a very rare ^{17}O. Oxygen (and hydrogen) isotope ratios from liquids such as water are expressed using the (δ) notation relative to an international standard like Standard Mean Oceanic Water (SMOW) or Vienna standard mean oceanic water (VSMOW). When oxygen isotopes are collected from carbonates such as bone, the composition can also be compared to the standard PDB. The conversion between SMOW and PDB is achieved using the following equation: $\delta^{18}O_{SMOW} = 1.03091(\delta^{18}O_{PDB}) + 30.91$. A substance with a $^{18}O/^{16}O$ ratio equal to that of PDB ($\delta^{18}O_{PDB}$ = 0‰) would have a $\delta^{18}O$ value on the SMOW scale of 30.91‰, but the same $^{18}O/^{16}O$ ratio. (Ratios are calculated with the following equation and expressed in parts per thousand:

$$\delta = \frac{(^{18}O / ^{16}O_{\ SAMPLE} - ^{18}O / ^{16}O_{\ PDB})}{(^{18}O / ^{16}O_{\ PDB})} \times 1000$$

Water molecules can assume different molecular configurations (nine exist) that incorporate different types of isotopes of hydrogen or oxygen, the most common of which is $^{1}H_2{}^{16}O$. In the hydrologic cycle, oxygen isotope compositions ($^{18}O/^{16}O$ ratios) vary in meteoric water depending on variables such as latitude (distance from the equator), altitude, and distance

from the sea. The primary factor influencing the composition, however, is temperature. In an early study dedicated to the behavior of isotopes, Urey (1947) indicated the importance of temperature in the fractionation process that results in isotope ratio variations. The relationship between water $\delta^{18}O$, temperature, and the equilibrium $\delta^{18}O$ of calcite was determined empirically by Epstein et al. (1953), later modified by Craig & Gordon (1965): $T°C= 16.9-4.2(\delta_c-\delta_w) +0.13(\delta_c-\delta_w)^2$. The oxygen isotopic composition of precipitation changes systematically with altitude and latitude, and for example, becomes isotopically lighter with increasing altitude (Fricke et al. 1998, Rozanski et al. 1993, Faure 1986).
Temperature affects fractionation at a rate approximately 0.5‰ for every °C for oxygen. Similar effects are found with increasing elevation and increased distance from the equator (both of which correspond to precipitation and lower temperature). The majority of net water evaporation and subsequent precipitation occurs in the tropics at or near the equator where the earth's surface area and temperature are greatest. Evaporation and boundary layer diffusion between atmosphere and ocean are two main factors in this low-latitude region that contribute to the bulk of atmospheric moisture (Boyle 1997). Vapor leaving the surface of the ocean cools as it rises and rain forms when the dew point is reached (Hoefs 1997). During the removal of rain from a moist air mass, the residual vapor is continuously depleted in the heavy isotopes because the rain leaving the system is enriched in ^{18}O and deuterium (Craig & Gordon 1965). Dalai et al. (2002) collected water samples of Himalayan origin from three different seasons (summer, monsoon and post-monsoon) and found seasonal and altitudinal variations in water originating from higher elevations and those collected during monsoon seasons. They were both characterized by well preserved isotopic signatures and displayed relatively depleted isotopic compositions. Non-monsoon rainfall and river water from which the samples were taken, exhibited signatures typical of evaporation (excess in the hydrogen isotope deuterium).

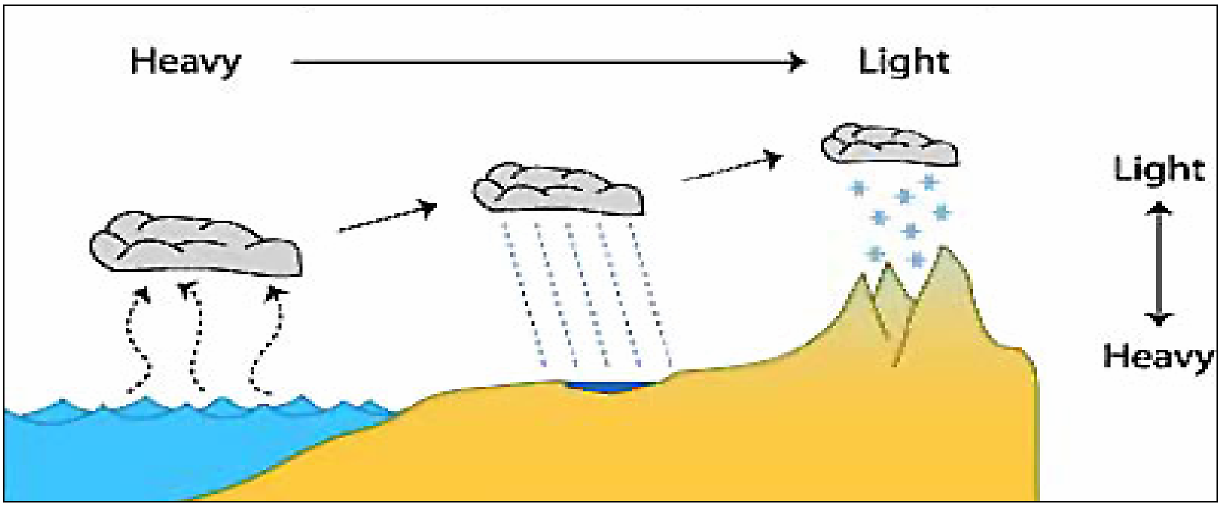

Figure 7.2. The gross partitioning of isotopes in vapor and precipitation

Researchers interested in tracking oxygen isotope distributions worldwide or reconstructing palaeoclimate have been collecting data for decades in an effort to create a topographical overview based on temperature, altitude, longitude and latitude, and distance from the sea. For example, approximately 180,000 sets of isotopes and meteorological data were accumulated by the International Atomic Energy Agency (IAEA data 1969-1994). From this extensive data base it can be decoded how geographic factors influence the isotopic composition of precipitation. An example of this is provided by the Online Isotopes in Precipitation Calculator (OIPC), in which data pertaining to geographical and topographical parameters for the area in question are entered and isotopic ratios for deuterium and oxygen are estimated.

7.8 Oxygen fractionation

Like other elements, oxygen too undergoes a fractionation process, which results in variable isotope ratios occurring in the different physical states of water and under the influence of various atmospheric conditions. For example, in the fractionation of water, H_2O with ^{18}O ($H_2{}^{18}O$) is heavier than H_2O with ^{16}O ($H_2{}^{16}O$) and does not evaporate as easily because ^{18}O requires more energy for evaporation than ^{16}O. Molecular bonds holding lighter isotopes break more easily than those holding heavy isotopes. Because temperature directly affects this process, the greatest differences in isotopic composition are observed at lower temperatures, which then gradually disappear as temperature increases. When water evaporates (kinetic fractionation), the water vapor is enriched in molecules carrying lighter isotopes compared to the water remaining in the fluid state. By contrast, when this vapor condenses into water

(equilibrium fractionation), it becomes enriched in molecules carrying the heavier isotope compared to the vapor left behind (Fig. 7.3).

During precipitation a raindrop's readiness to fall and its velocity are controlled by the size of the droplet. The ratio of ^{18}O to ^{16}O in the water droplets is always less than that of the seawater where it originates. This is mostly because during evaporation, isotopic fractionation occurs under non-equilibrium conditions in which relative humidity is less than 100%; the condensation of water in the clouds occurs under equilibrium conditions, with a relative humidity of 100%. Isotopic fractionation under non-equilibrium conditions, which prevail during evaporation, is significantly larger than the equilibrium fractionation that occurs during condensation. Over oceanic islands and coastal locations, average ^{18}O to ^{16}O ratios in precipitation are consistently lower than Standard Mean Oceanic Water (SMOW), which approximates the mean isotopic composition of the world's oceans (Physics Today 2004).

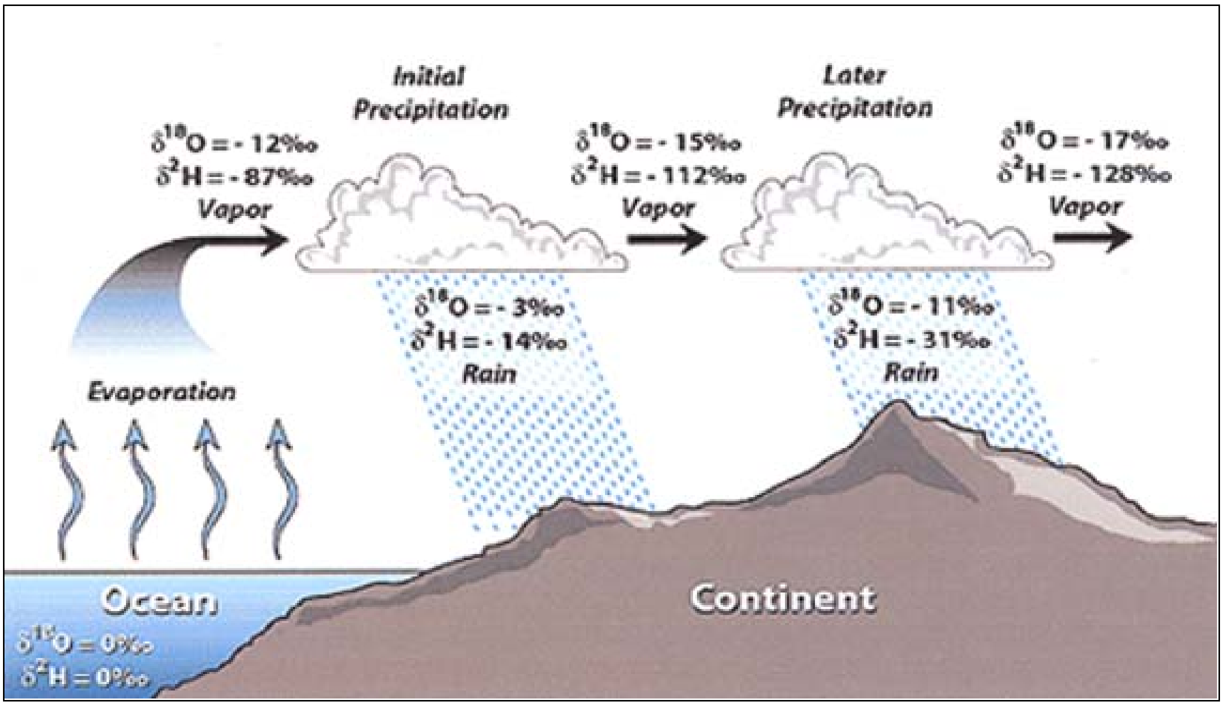

Figure 7.3. This illustration shows how depletion in ^{18}O and ^{2}H progresses as water vapor travels inland away from the oceanic source. Notice how values become more negative (depleted) over mountains as water vapor goes through repeated cycles of condensation and precipitation (after SAHRA, based upon Hoefs 1997 and Coplen et al. 2000).

The continuous process of evaporation and precipitation that happens daily around the world can be viewed as multiple stage distillation, also called Raleigh-distillation. The ocean is the reservoir and the rain at different latitudes is the condensate at different stages. As water that has vaporized at the lower latitudes moves to higher latitudes, perpetual steps of raining out that occur as the vapor progresses towards the poles leaves it more and more depleted in the heavier ^{18}O isotope, which is preferentially lost in the clouds as precipitation (Fig. 7.4).

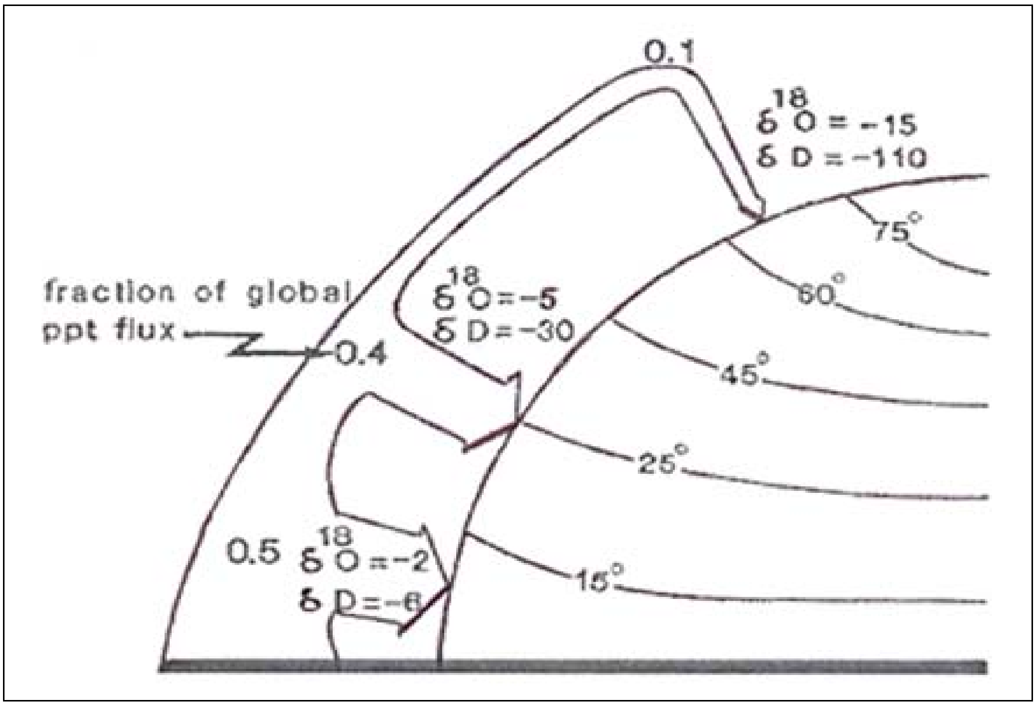

Figure 7.4. Illustration of global Raleigh fractionation in which water vapor (ppt) becomes isotopically lighter through depletion of the heavy oxygen isotope ^{18}O in the more northerly latitudes (after Craig and Gordon 1965).

Based on the Raleigh distillation model, the positive correlation between $\delta^{18}O$ in precipitation and local temperature is due to the ongoing isotopic depletion of cloud water during loss of water, which is assumed to be caused by cooling condensation (Saurer et al. 2002). It should be noted, however, that Bowen and Ravenaugh (2003) have indicated the existence of significant zonal heterogeneity in vapor transport patterns at high latitudes, which can lead to dramatic variability in isotopic compositions of $\delta^{18}O$ and δD and complicate isotope ratio predictions for these areas (Fig. 7.5). For example, they show that the interpolated δD of precipitation at 60° north latitude ranges from a low of -170‰ over southeastern Alaska, to a high of -50‰ in the central north Atlantic, and the interpolated δD at sea level over the Atlantic Ocean at this level is 45‰ heavier than that over the Pacific. Atmospheric circulation

and other climatic parameters are apparently the cause for these observations (Fig. 7.7) (Bowen & Wilkinson 2002).

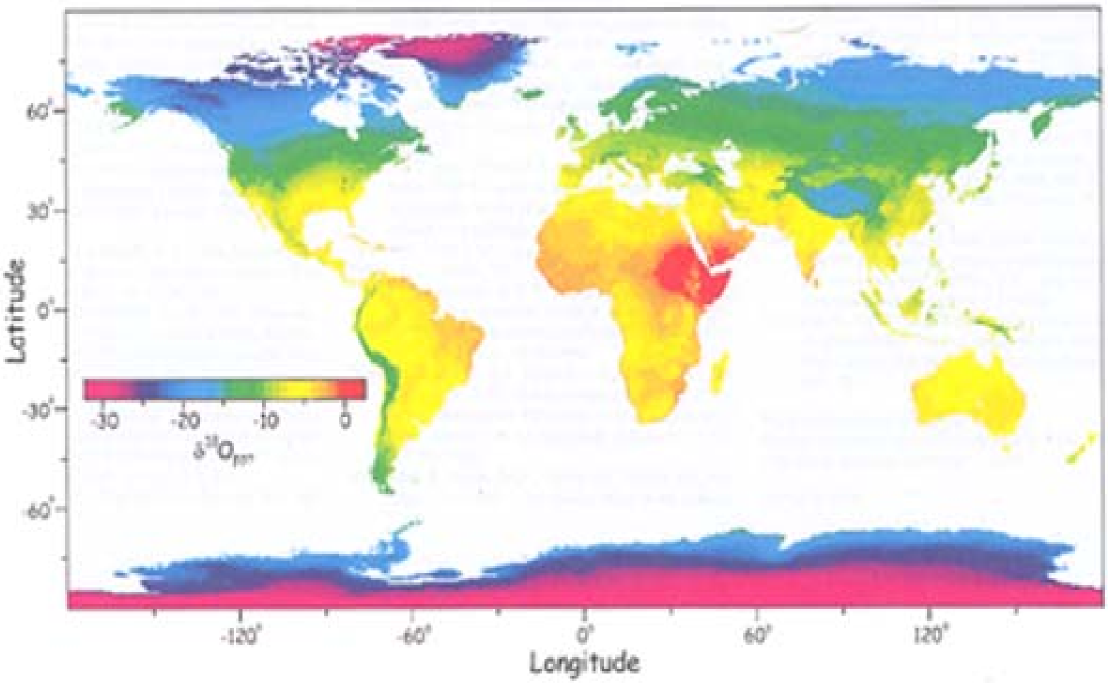

Figure 7.5. High resolution map illustrating the steady depletion of $\delta^{18}O$ with distance to the equator and in high elevation areas. Values are most negative in the polar reaches (after Bowen & Wilkinson 2002).

Indeed, water analysis from the alpine area in the vicinity of where a 5300 year old mummified human was discovered entombed in glacier ice, affectionately dubbed the "Iceman" or sometimes "Ötzi", showed spatial differences in isotopic composition within a relatively small geographical region between north to south and east to west (Müller 2005).

Rozanski et al. (1993) found that with increasing temperature, precipitation becomes enriched and the heavier isotopes ^{18}O and ^{2}H in a linear relationship (see Fig. 7.6). Cold air on the other hand, holds substantially less vapor and has previously lost most of its water. The existing pockets of vapor extant under cold temperature conditions, however, have already lost most of their heavy isotopes as precipitation and are isotopically extremely light. This phenomenon is often referred to as the "rainout effect" or "amount effect". Water vapor traveling over high altitude areas also becomes isotopically lighter because temperature drops with increasing elevation, and both rain and snow falling from the clouds depletes vapor further of heavier oxygen isotopes. This "altitude effect" is the result of continuous isotopic

depletion of the atmospheric water vapor as it rises over the slopes of the mountains and heavy isotopes are preferentially removed by precipitation.

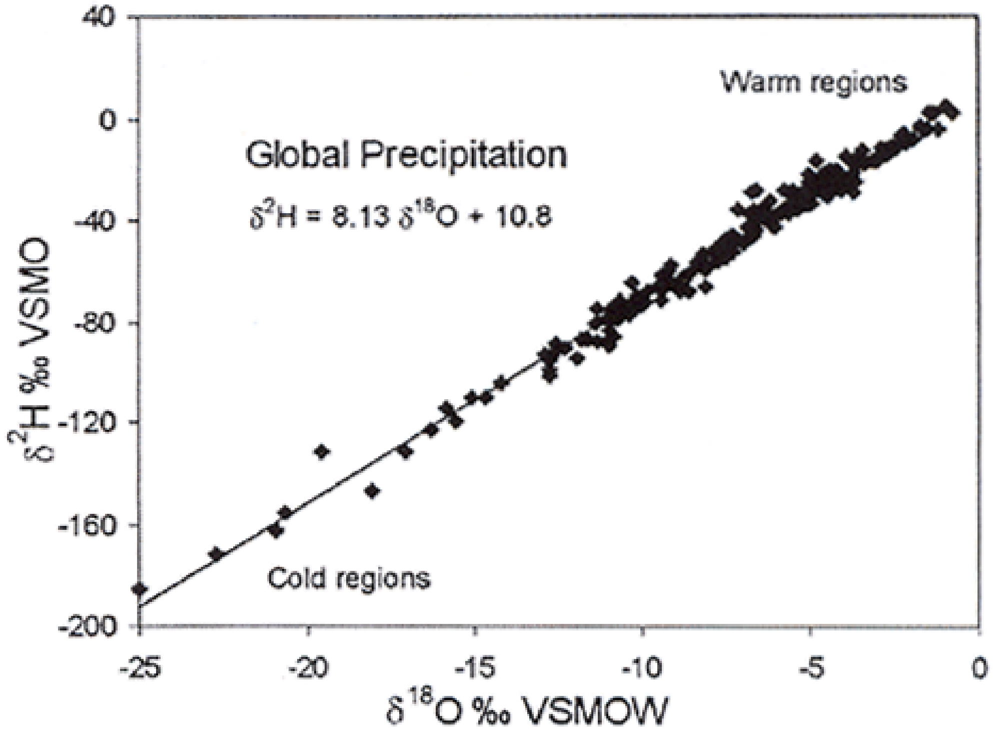

Figure 7.6. Linear relationship between δD and δ^{18}O data compiled by Rozanski et al. 1993 and plotted against the global meteoric water line as defined by Craig (1961), (after SAHRA, 2006).

An altitude change of 250 m produces a clear difference between the two ratios δ^{18}O and δD which are preserved when the precipitation infiltrates the aquifer, and detectable differences in isotopic composition with altitude changes of 100 m are indicated by the OIPC. Dalai et al. (2002) determined a change of 0.11‰ per 100 m in their examination of Yamuna river water, which differed by a factor of about two less than a previous study of the Ganges river water that originates from a similar altitude, indicating spatial-regional differences in fractionation.

Fairchild et al. (1999) used isotopic parameters to investigate water sources from Glacier de Tsanfleuron in Switzerland and showed that melt water and glacier ice samples lay close to the meteoric water line defined by waters from small tributary streams. Heavy isotopic compositions of bulk melt water were caused by rainfall events. These, however, recovered within days to a δ^{18}O baseline of around −12‰. The study revealed an absence of diurnal variations in δ^{18}O.

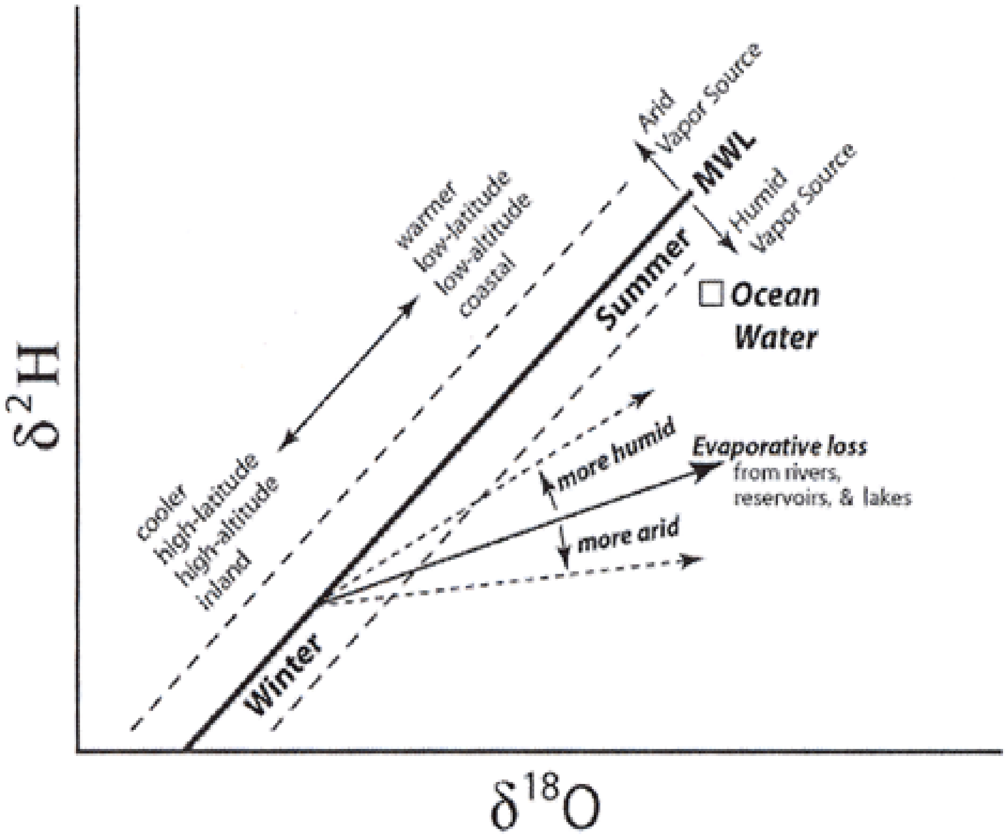

Figure 7.7. Effects of hydrological processes on oxygen and hydrogen isotopic composition in water, with more negative values near the axis origin (after SAHRA, 2006).

The observed influence of altitude on isotopic ratios lays at the very core of the Volders oxygen isotope study since the cooler temperatures characteristic for higher elevations in the area result in significant isotopic composition differences along the mountain incline.
Sauer et al. (2002) also noted that certain additional variables can influence the isotopic composition of precipitation such as atmospheric circulation, fluctuations in rainfall amounts, and seasonality, which for example, results in a strong depletion of heavy isotopes in winter. A further important observation is that isotope composition of precipitation decreases with distance to the coast, a phenomenon called the "continentality effect" (see Figs. 7.3 and 7.7).
An important point of contention, particularly considering the steep, mountainous topography in the immediate vicinity of Volders, is ascertaining the amount of mixing that occurs once rainwater reaches the earth and seeps into the soil and rock of a given area. Norton and Panichi (1978) studied water samples from natural springs and artesian wells in the Abano region of northern Italy and indicate that the isotopic composition of these potential drinking

water sources are characterized by their local geological environment and subject to the influences of groundwater circulation and reactions between rocks and the groundwater itself. They found that local variation in isotope compositions of water is consistent with the mixing of local meteoric waters and formation fluids that were ultimately derived from northerly situated Alpine sources.

Water that falls as rain on mountain slopes either infiltrates the geology or runs off, forming fast flowing streams that transport the water to catchments, lakes, rivers or other destinations. As the water travels downward it undergoes a variable mixing process with waters possessing different isotopic compositions unique for the area or elevation where they precipitated out (Fig. 7.8).

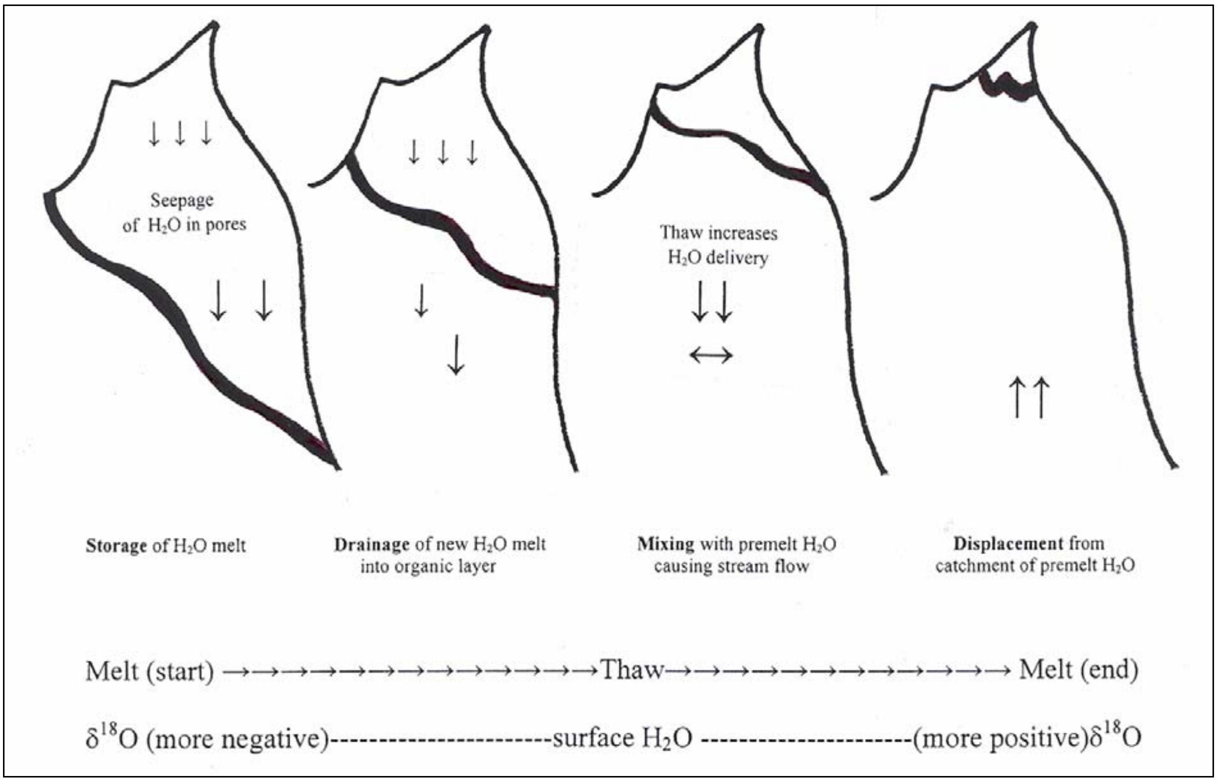

Figure 7.8. Changes in H_2O delivery to ground and surface water during annual snowmelt on the mountain incline.

It is difficult to reconstruct exactly how the original precipitated water on the rugged slopes above Volders interacts with different waters from the surrounding mountain environment, and further what the effects of evaporation and transpiration of surface waters combined with natural mixing processes have on the isotopic composition. What is certain, however, is that

there is a gradual enrichment in the heavier oxygen isotope ^{18}O as water moves down the slope. Research on snowmelt runoff generation in a small alpine catchment area in Canada by Carey & Quinton (2004) revealed the complicated mixing process of new meltwater with meltwater stored from the previous year and its potential impact on isotopic composition. In a study of the origin and flow paths of water in mountainous regions using $\delta^{18}O$, Maréchal and Etcheverry (2003) report active groundwater circulation and the usefulness of oxygen isotope composition in tracking "recharge" altitudes as water moves down a subvertical path and becomes isotopically more enriched. Groundwater recharge was also observed to result from inflowing lake, stream and river water.

Fractionation of oxygen is not limited to environmental circumstances and it is apparent that the physiology of an organism can also fractionate oxygen isotopes and lead to signatures that are actually untypical for their respective environment. Bernasconi (2002) showed that ground water will affect tree stress and that a variety of chemical, physical, and biological factors can affect the oxygen isotopic composition of the tree rings. Different parts of plants also exhibit variations in isotopic ratios. Water in leaves is enriched in ^{18}O because of preferential evaporation of ^{16}O resulting from leaf respiration, and is especially marked in hot, arid environments (Yakir 1992). C_4 plants in arid environments transpire late in the day after C_3 plants have stopped, leading to $\delta^{18}O$ value differences of up to 10‰ (Sternberg et al. 1984). In turn, dietary preferences of animals feeding on these plant types plays a role in that organism's own $\delta^{18}O$ values.

Mammals have a constant body temperature and their isotopes tend to reflect source water oxygen isotopic composition (Millard et al. 2004). They are also slow growing, which results in a "smoothing out" of signatures distinct for seasonal variations in meteoric H_2O. However, metabolic rates can have a significant impact on the isotopic compositions of oxygen isotopes in body water compared to meteoric water. In a study focusing on isotope signatures in bone phosphate, Luz & Kolodny (1989) showed that when metabolic rate is high compared to the rate of drinking, bone phosphate is not sensitive to changes in the given ratio of ^{18}O in environmental water. In addition, the $\delta^{18}O$ ratios in skeletal tissues depend not only on the $\delta^{18}O$ of the environmental water, but also the ambient temperature and humidity. Fluid metabolism in mammals can vary significantly between species, especially those adapted to environmental extremes like heat and the accompanying water stress. According to Lee-Thorp et al. (2003), water outputs include liquid forms such as urine and sweat that have an ^{18}O isotope composition similar to body water, and as a vapor lost through the skin, nose, and mouth of panting animals. Like the plant leaf adapted to arid climates, the vapor exhaled

during respiration by these animals is enriched in the lighter $H_2{}^{16}O$, a thermophysiological circumstance that can have significant affect on the isotope composition of body water. Herbivorous browsers that acquire their water primarily through leaf eating exhibit higher ^{18}O values than animals that are obligatory drinkers (Sponheimer & Lee-Thorp 1999b). Their examination of enamel carbonate $^{18}O/^{16}O$ from the Swartkrans and Equus Cave fossil assemblages showed that patterns could be interpreted in terms of drinking behavior, diet and physiology. This relation varies for different species and must be calibrated for the species under consideration. In some species of animal, isotopic ratios of oxygen are influenced by both diet and physiology. Some vertebrates, for example, roe deer, acquire water primarily through feeding and not directly by drinking. Different mechanisms of water conservation have evolved in some species as a response to fluid stress experienced in extraordinarily arid habitats. Adaptive pressures also result in specialized metabolism of nutrients that affect the uptake of oxygen in tissues. Therefore, in some species, the oxygen isotope ratios do not necessarily represent the isotope composition of this element in drinking or meteoric water. Based upon this knowledge, it is a requirement that the specific mechanism of uptake for the isotope be assayed for the particular organism used for sampling. Isotopes analyzed for species like man that obtain water by drinking and do not possess specialized metabolic pathways accurately parallel the isotopic composition of meteoric water (Kohn 1996).

Luz et al. (1984) indicate that the fractionation of oxygen isotopes between body and environmental drinking water is dependant on the rates of both drinking and respiration. Schoeninger et al. (2000) also indicate that body size in mammals influences H_2O balance. Since oxygen is ubiquitous in every solid, liquid and gas consumed and excreted, body water oxygen isotope composition in mammals is a function of the oxygen mass balance of the specific animal (Kohn 1996, 1999).

7.9 Oxygen isotopes in archaeometry

The use of oxygen isotopes in gaining access to information regarding climate, origin and habitat use have only recently been added to the list of empirical tools available to anthropologists. The unique fractionation characteristics exhibited by oxygen isotopes, which lead to varying compositions in water, water vapor, snow and ice, have made it an invaluable substrate for climate studies, especially those focusing on the changes in climate over time. Accessing oxygen isotope signatures, however, is not limited to ice cores or deep lake sampling commonly associated with palaeoecological studies.

Efforts in this direction have been made possible since the pioneering works of Tudge in 1960 who developed the method of producing a stable phosphate preparation necessary for analyzing oxygen isotopes, Dansgaard (1964) who developed an equation for calculating the relationship between annual mean air temperature and the amounts of oxygen isotopes in precipitation, and Longinelli (1973), the first to perform oxygen isotope analysis on bones and teeth deriving from terrestrial mammals. In many instances isotopes have proven an effective means of reevaluating past preconceptions regarding nutrition, hunting, fishing, agriculture and climate. More recently, the interpretive potential of this method has been expanded to elucidate questions regarding migration and even more specifically, local population movement. To accomplish this task a thorough examination of the isotopes deriving from bone apatite is necessary. Data on oxygen isotopes are obtained parallel to $^{13}C/^{12}C$ measurements of CO_2 released from carbonate in biological apatite, yet was often ignored because of the presumed risk of diagenetic alteration through exchange with water molecules (Lee-Thorpe et al. 2003). Oxygen can also be extracted from the phosphate ions in the mineral phase of bone and tooth material.

It is possible to detect diagenetically unaltered isotopes in calcified tissues originating from humans, invertebrates and mammals for use in tracking climate fluctuations. An early study by Shackelton and Renfrew (1970) identified Neolithic trade routes by analyzing the ratio of ^{18}O to ^{16}O in Spondylus shells once used for jewelry or as an ornamental accessoire. They found that the calcium carbonate deposited by mollusks during shell formation is in isotopic equilibrium with the water it inhabits, and that the proportion of ^{18}O atoms to ^{16}O is not governed by biological factors but rather the principals of thermodynamics. The ratio in shells was found to be only slightly different from that of the parent water, and the differences were attributable to temperature. The important point here is that the shells possess a signature that is unique to the environment in which it was incorporated during uptake, and that this signature can be used later as a mode of identification for water source, temperature fluctuations and the organism's origin. The oxygen isotope ratios of these well preserved marine calcareous fossils found in deep sea forams are also indicative of the temperature of oceans and can be used to provide palaeoecological data over millennia. Importantly, mammalian bone exhibits a similar pattern of signature incorporation.

Luz et al. (1990) examined variations in $\delta^{18}O$ of white tailed deer deriving from different areas in North America and found that the deer living in the same localities displayed little isotopic variance in ratios yet were distinct from those originating from other regions. This indicated that the deer were acquiring their water from a relatively small area and also

illustrated these areas retained distinct isotopic signatures resulting from local humidity and precipitation characteristics. This information is important with respect to studies focusing on skeletal series originating from a specific locality.

Fricke et al. (1995) identified rapid atmospheric cooling at around 1400 to 1700 AD in their analysis of Norse and Inuit teeth from different North Atlantic sites. More importantly it was able to imply that environmental changes exerted a direct influence upon societal activities.

Oxygen isotope analysis of palaeosol (buried soil) can be used to ascertain the temperature and moisture content of a palaeoenvironments (Cerling 1992). This provides critical evidence supporting theories that environmental changes that took place during hominid evolution offered new dietary options resulting from fluctuations in temperature and rainfall (Vrba 1985).

Wright and Schwarcz (1998) used a combination of ^{18}O and ^{13}C values in tooth enamel to identify breastfeeding and weaning in human archaeological remains from the Kaminaljuyu site in Guatemala. Breast milk is more enriched in ^{18}O compared to drinking water, and enamel taken from teeth which differ in their completion of developmental make it an effective tool for discriminating between nursing and already weaned children.

Plant material can also be used to identify water origin, which then has implications for human use and adaptability to arid environments, such as a study conducted by Coltrain et al. (2005) who found the potential for using cellulose ^{18}O levels to investigate prehistoric irrigation practices and source water, used for maize production in the American South West.

7.10 Oxygen isotopes and migration

Migration patterns of people and aspects to colonization have always been of interest to anthropologists and archaeologists. Questions regarding residence change and territorial control represent major factors in determining population development. Conventional methods of tracing the movement of people or an individual from one geographic location to another rely primarily on the identification of similar attributes arising in different places. These can be man made or the man himself, and include things such as pottery styles, patterns of ornamentation on textiles, baskets, jewelry, ceramics, military gear, tools and other aspects of material culture. However, such items can be carried far from their origins or traded over long distances rendering them less reliable. Distinct morphological features related to cranial form, dentition and bone have augmented the artifactual method (Ramsl 2004). This archaeologically and osteologically based information is invaluable for clarifying what influences neighboring or far away populations had on one another, however, its limitations

become apparent, for example, in situations where skeletons are found without burial goods or without an associated settlement, or when preservation of morphological traits is inadequate. The use of stable isotopes for tracking migration has traditionally involved measuring strontium (Sr) and lead (Pb), elements ingested in diets from geologically distinct areas that subsequently become incorporated into bone tissue and teeth (Grupe et al. 1997, Price et al. 1998, 2000, 2001, Whittle 1996, Burton et al. 2003, Montgomery et al. 2000, Latkoczy et al. 1998, Iacumin et al. 1998, Lillie & Richards 2000, Ericson 1989, Price et al. 1994b, Bentley 2001, Burger & van der Merwe 1990, Sealy et al. 1995, Sillen et al. 1998, Hodell et al. 2004, Bentley et al. 2004, Knudson et al. 2004, Schweissing & Grupe 2003). A number of others have indicated the importance of isotope analysis in determining migration patterns (Bamforth et al. 2000, White et al. 1998, Wang et al. 1997, Gulson et al. 1997, Ezzo et al. 1997, Price et al. 1994a, 1994b).

Since oxygen isotope compositions characteristic for specific geographic regions are identifiable and to some extent predictable, it is logical to assume that recently emigrated individuals originating from isotopically distinct areas or altitudes within one geographical area are distinguishable from one another based upon their individual isotopic signatures (Fig. 7.9). The use of oxygen isotopes for clarifying archaeometric problem statements is based on the principle that the $\delta^{18}O$ isotopic composition of terrestrial water is controlled primarily by the $\delta^{18}O$ composition of the local meteoric precipitation, and that the oxygen isotope composition of organic material is regulated by the $\delta^{18}O$ isotopic composition of its drinking source or soil water. Therefore, it is possible to measure oxygen isotope ratios in various organic compounds and use the information to trace the origin of the specific molecules. Longinelli (1984) established a correlation between oxygen isotopic composition of mineralized mammalian tissues and oxygen isotopes of ingested meteoric water. More specifically, the oxygen isotope composition in drinking water is incorporated into the skeletal tissues of mammals including humans in vivo. Furthermore, the composition of phosphate and structural carbonate in mammalian enamel and bone apatite are linked to that of body water at a constant temperature of 37°C and can be recovered and recorded (Bryant et al. 1996). This correlation proves essential in the identification of population movement or individual origin, and provides a chemical record of residency at the stage of life at which the tissue was formed. The first attempt to use ^{18}O for purposes of identifying place of origin was conducted by Schwarcz et al. (1991). It allows reconstructing the oxygen isotopic composition of water drunk by “ancient” people and provides an additional isotopic tracer in particular for areas with considerable altitude contrast or across a watershed. It should be

noted that "modern" people consume significant amounts of water originating from sources other than those in their immediate living area, and are therefore not useful for estimating the isotopic composition of environmental water (Luz & Kolodny 1989). Others indicate that bone phosphate from modern humans can also be tested and is correlated to environmental water, but that a correction factor must be employed Levinson et al. (1987).

As a result, organisms like humans, all carry the $\delta^{18}O$ isotopic signature of the water they drank in the area of their residency. This makes them good ecological markers of the surrounding in which they live and also their locational stability (sedentarism) or movement (migration). This approach offers a powerful method of elucidating some aspects of life history from skeletal remains and for identifying those people who traveled considerable distances or resided in areas geologically unique to one another.

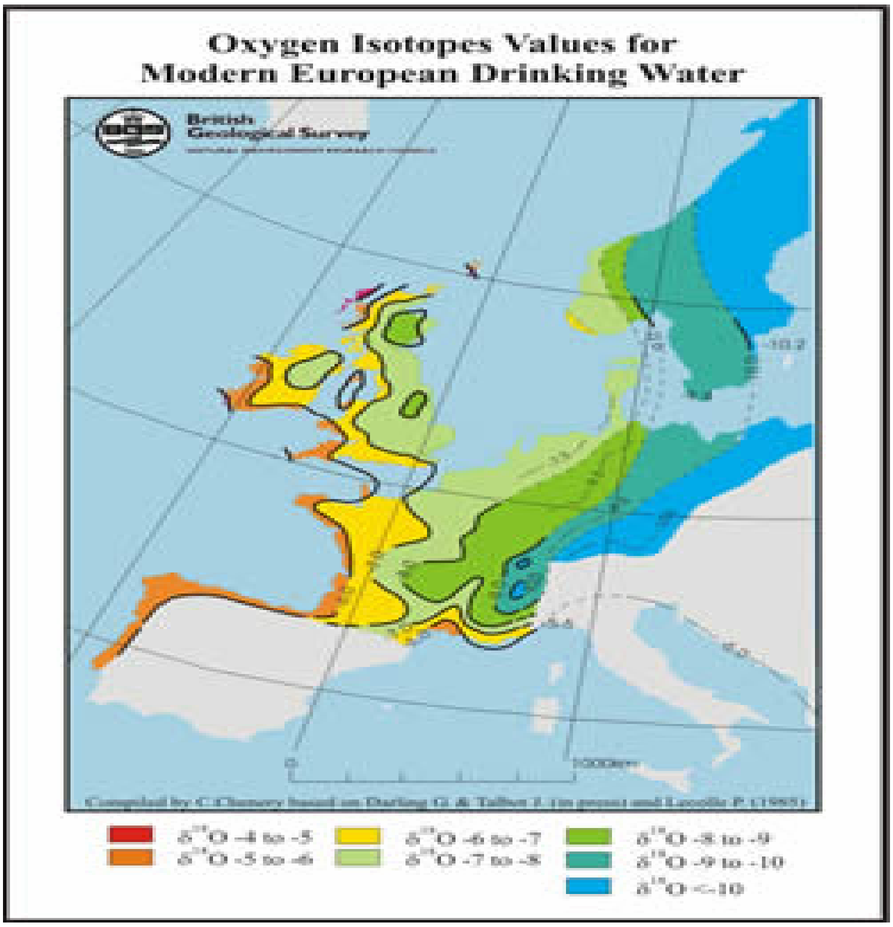

Figure 7.9. Present European $\delta^{18}O$ isotope ratios (British Geological Survey).

Budd et al. (2004) indicated that oxygen isotope analysis is a highly effective tool by which to trace population movements at a regional and international level and to determine if

individuals in a burial series are local or non-local. The efficaciousness of using $\delta^{18}O$ isotope ratios in human bone for anthropological studies focusing on migratory behavior was shown by White et al. (1998) who compared the bone oxygen isotope signatures in archaeological remains to those characteristic for a specific geological region to trace geographical origins in the Valley of Oaxaca in Mexico. Evans et al. (2006) examined two burials from Amesbury England, one of which is known as the "Amesbury Archer", and found that this individual's ^{18}O composition is not characteristic for Britain but rather central Europe, implying that the "Archer" was a non-local. The $\delta^{18}O$ values from the second burial indicated that the individual was probably from southern England or Ireland. Scientists analyzing the remains of the 5300 year old Neolithic "Iceman" mentioned earlier, which was discovered in Northern Italy, found the bones to be significantly lighter in ^{18}O compared to the tooth enamel, indicating that the Iceman spent his childhood at lower altitudes (Müller et al. 2005, Hoogewerff et al. 2001). Dupras and Schwarcz (2001) used oxygen isotopes signatures in human remains discovered at the Dakhleh Oasis, Egypt to identify non-local inhabitants.

Various meteorological, biological and geochemical factors cause foods in different environments, such as high altitude versus low altitude, to have characteristically different stable-isotope compositions (Kohn et al. 1996, Koch et al. 1994). Because the isotope composition of the bone reflects what has been consumed, this data can tell us about the individual's occupancy of different environments. This in turn allows investigation of a wide range of issues including resource use and migration patterns. For example, Cerling et al. (1998) has found that foods at low altitudes generally have higher D/H, $^{13}C/^{12}C$, and $^{18}O/^{16}O$ than foods at higher altitudes because of differences in rainwater composition and in the proportions of plants that use C_4 versus C_3 photosynthetic pathways (C_4 plants have a higher $^{13}C/^{12}C$ than C_3 plants), the latter is of less importance here.

8. Methods and Material

8.1 Morphological examination

Basic information regarding burial method, grave geology, disturbances to the grave, macroscopic condition of bone preservation and the presence of grave associations was recorded for all 144 individual skeletons “in situ”. An osteological field examination was conducted on the remains, which proceeded simultaneous to the removal of the individual skeletal elements. Height estimations and morphological age and sex determinations were made using criteria standard for European osteological examinations.

The osteological examination included the measurement of long bones to facilitate the estimation of physical height. Equations to calculate height estimation are provided by Bach (1965) and Breitinger (1938) for adult European males and females respectively. The estimations for height in subadults were made according to Telkkä et al. (1962).

The identification of sex related morphological characteristics necessary for discriminating adult male and female individuals in skeletal remains, was made using guidelines presented by Acsádi and Nemeskéri (1970). Subadult gender was determined using information provided by Schutkowski (1987) and White & Folkens (1991).

A morphological age at death estimation for adults was made in the field and later repeated in the lab, which relied on the examination of a combination of features including alterations in rib end morphology, metamorphosis of the pubic symphyseal surface, fusion of cranial sutures and general age-related bone alterations, such as tooth abrasion and non-rheumatic arthritic pathologies associated with wear and tear (Brooks & Suchey 1990, Herrmann et al. 1990, White & Folkens 1991). Age estimations for subadults were based on epiphyseal development, long bone length and tooth eruption (Herrmann et al. 1990, Stloukal & Hanáková 1978, Ubelaker 1989).

Gross pathological changes and bone defects associated with traumatic events were also recorded during this examination. Dental status for all individuals was assayed to determine tooth health, wear, and if present, extent of tooth loss and alveolar resorption.

Following the removal of the skeleton, the bones were placed in labeled boxes for transportation to the City Archaeology in Hall in Tirol. The bones were subsequently washed with a soft kitchen scrub-brush under tap water and laid out on tables to dry. Bones in a clean dry state, were further examined for anomalies, variations in structure, small defects resulting from injury, pathologies such as porosities, vascular indentations, arthritic abrasions or other traces of disease, including tooth decay, abscesses and dental calculus that may have been obscured previously by dirt. Information collected for each burial during both the field and

lab examinations were recorded in the anthropological catalogue, which is designed for quick referencing.

8.2 Tooth cementum annulation (TCA)

To facilitate the counting of incremental lines in tooth cementum in an effort to provide accurate age at death estimates (see section 5 for details), it was necessary to remove teeth from the alveolar socket.

Preferably, one maxillary or mandibular premolar was carefully extracted from the alveolar bone. The tooth was embedded in Biodur E12 resin (Gunter von Hagens, Heidelberg) and left one week to harden in a desiccating box.

Twelve thin sections (70µm) of the cervical third portion of each sampled tooth root were made using a Leica 1600 Leitz Co. rotating microtome. The thin sections were fixated to glass slides with Eukitt and capped with cover slips. The slides were viewed using a Zeiss Axioskop 2 plus phase contrast light microscope under objective power first at 20x for scanning purposes and subsequently 40x for analysis. The microscopic image was digitalized using the AxioVs 3.0 and Photoshop 7.0 software. Incremental lines were manually counted directly on the monitor, starting with the first dark band immediately adjacent to wide light band marking the point of eruption (see Fig. 5.2) and tallying the number of successive dark bands outwards in the direction of the tooth root surface. Incremental lines were also counted digitally with the aid of a computer software program (Auto-TCA) specially developed by Czermak et al. (2006) at the Ludwig-Maximilians-University in Munich. The total number of counted lines was then added to the person's estimated biological age in years at the point of eruption of that particular tooth.

An in depth specification to this method is provided in the section labeled TCA.

Sampling criterion

Tooth samples taken for this age estimation study had to meet two basic criteria in order to allow for a tooth cementum annulation (TCA) analysis. First, healthy intact teeth needed to be present. A number of individuals at Volders had either experienced total tooth loss during their lifetime or the cranial skeleton was not recovered. Others had incurred such extreme abrasive damage upon their teeth during their lifetime, that not only the crown, but also the upper portion of the tooth root dentine, from which the sections are normally taken, was missing.

Pathological conditions, such as periodontitis, caries or abscesses had deleterious effects rendering the diseased tooth inadequate for TCA analysis. Several important factors need to be taken into consideration with regards to dental pathology when sampling teeth for a cementum examination. Some researchers have shown that an unhealthy oral cavity environment can severely compromise the structural integrity and biochemical composition of the extracellular matrix, which plays a pivotal role in maintaining the homeostasis of cementum (Grzesik & Narayanan 2002). Others have shown that systemic diseases such as Type 2 diabetes or non-insulin-dependent-diabetes (NIDDM) can have a substantial influence upon tooth loss through their negative effects on cementum production and stimulation of alveolar bone resorption (Gokhan et al. 2004). In an effort to study the mechanisms of tooth loss, Gokhan et al. examined the teeth of 46 patients suffering from Type 2 diabetes, a disease which afflicts between 2-6% of the modern western population, and found significant differences in the thickness of cementum in 4 vertical areas of the tooth root spanning from the apex to the cervical portion. Paget's disease, with a prevalence of approximately 3% in people over 40, has been shown to cause hypercementosis, leading to substantial formation of cementum, and conditions such as hypophosphotasia result in minimal cementum formation and accompanying tooth exfoliation (Kaplan et al. 1994).

Since histometric changes in tooth cementum are proven to be influenced by not only local factors and systemic disease but can also result from idiopathic conditions, the question then arises if teeth should be sampled at all from a skeleton if evidence exists for periodontal disease or extensive tooth loss resulting from a suspected or documented systemic disease that might in turn skew conclusions based on cementum structure. Foster (1994) indicates that more than 40% of Pima Indians in the United States have Type 2 diabetes, evidence that the prevalence of this disease can be culture specific. Certainly awareness of these facts would be vital before sampling such a population for this particular examination. In 1996, Houck and his colleagues gave the cautionary reminder that "biology does not always correlate with expected outcomes, particularly in such multifaceted traits as age".

Another frequent problem encountered, which potentially altered tooth cementum structure and influenced the selection of tooth samples, was the destruction to teeth caused by diagenetic factors such as the acidic nature of the earth that some of the skeletons were buried in or the mechanically erosive hydrokinetic effects of the ground water some skeletons were subjected to. These sufficed to virtually dissolve some skeletons completely, leaving only the remnants of tooth crown enamel (Fig. 8.1).

In addition, a past construction project next to the cemetery resulted in the dumping of large quantities of mortar and lime in a shallow ditch made directly above a cluster of burials that was apparently utilized as a waste pit for discarded building materials. The skeletal remains, including the teeth, under this ditch were badly damaged by the chemical effects of the lime and unusable for this analysis. As with diseased teeth, it was important to exercise selectivity in choosing the samples so as not to include teeth that may have been exposed to corrosives and undergone postmortem structural changes.

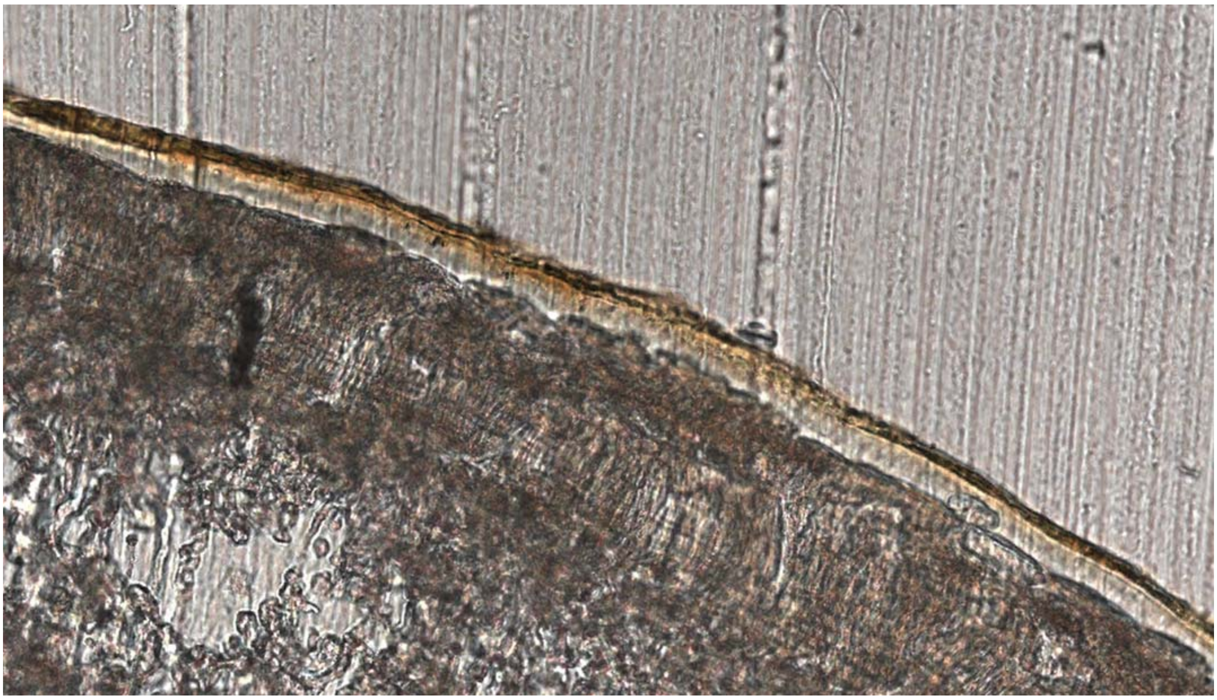

Fig. 8.1. Photomicrograph showing diagenetically altered tooth root dentine and cementum layers. (Premolar, 75µm thick, 20x, Volders burial 27).

Second, in addition to selecting healthy tooth specimens, primarily teeth from adults were sampled. It is generally not necessary to apply the method to individuals younger than approximately 20 years of age, because various characteristics intrinsic to skeletal and dental development provide indicators more than sufficiently accurate to make an age at death assessment, however, several exceptions were made during examination of the Volders material.

Tooth sections

A point of concern related to the TCA method following sampling and sectioning, was where on the thin section to perform the microscopic scan. Anomalous tooth root structure can inadvertently lead to exaggerated or reduced line totals. It was therefore imperative to locate an area of tooth root that visually appeared to be normal prior to both the automated and manual scans. Areas displaying cementum fissures, cracks, indentations, wave-like projections, tears produced by the mechanical removal of the tooth and diagenetically altered or destroyed portions were all strictly avoided (Fig. 8.2).

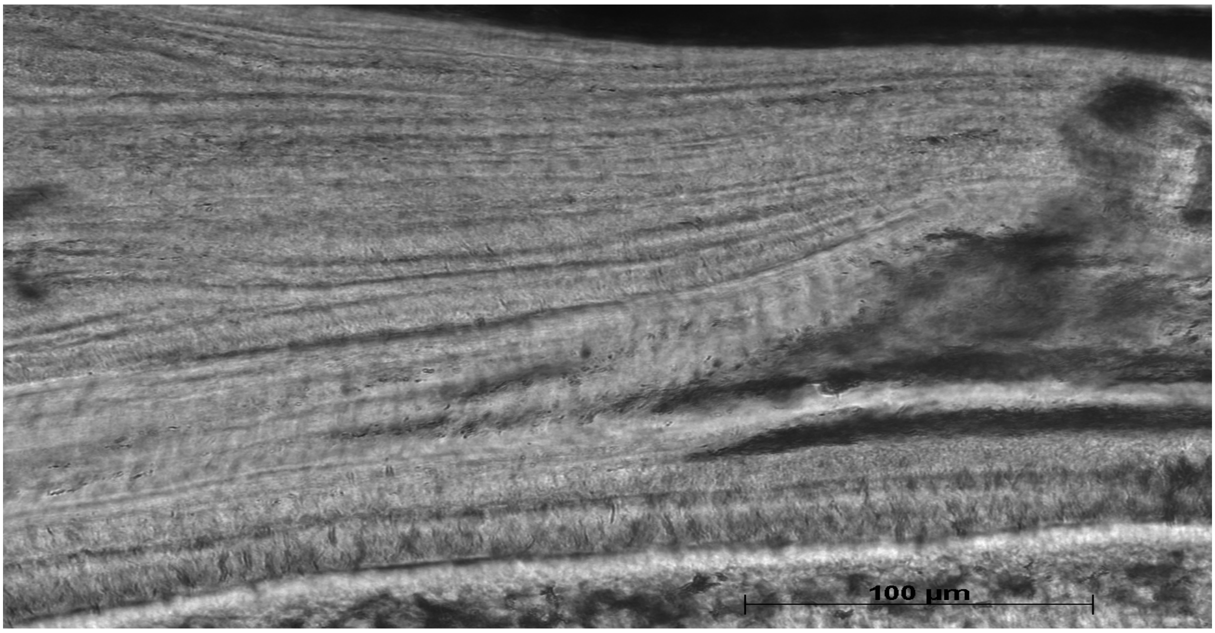

Figure 8.2. Photomicrograph showing irregularly organized cementum layers. (3rd molar, 75µm thick, 40x, Volders burial 37).

8.3 Collagen extraction

Organic bone collagen was extracted and the isotopic composition was found utilizing isotopic ratio mass spectrometry. Collagen is an organic component of the bone, and like other archaeologically recovered organic material, is subjected to the elements of nature that can potentially contaminate and alter its structure. The quality of its preservation must therefore be carefully monitored before analysis. An attempt was made to actively reduce the influence of diagenetic effects by selecting only certain portions of the bone. The intention was to avoid bones that visually appeared to be decomposed, overly porous or unsuited for analysis due to the lack of intact organic substrate. This visual selection proved to be the least

effective method for determining destruction through diagenesis. Inevitability, skeletal material that gave the optic impression that no protein could possibly have survived turned out to be exceptionally well preserved and virtually free of diagenetic assault. By contrast, bone samples derived from anatomically well preserved skeletons, looking almost like fresh bones, were sometimes unusable for isotopic analysis due to the advanced state of protein decomposition.

Cortical sections of a long bone diaphysis, preferentially from the anterior femoral surface, were chosen as a sampling region. The femur diaphysis was used because it is less porous and has a thicker shell of compact bone than other elements, which make it less susceptible to diagenetic effects and produces a larger amount of bone material relative to sample size. A crescent shaped sliver of cortical bone, weighing approximately 3-4 g, produced by two separate band saw cuts angled inwards towards one another, was taken from the anterior diaphysis and mechanically scraped of gross impurities with a scalpel. The sample was pre-washed under running tap water and subsequently cleansed ultrasonically in distilled water. This cleaning instrument was especially effective in removing dirt and other impurities locked in pores, holes, seams and the fine labyrinth-like network of the bone's endosteal matrix. In the event, that the femur was not viable for sampling, the tibia, humerus, radius or ribs, in this order of preference, were used as substitutes.

The sample was then dried at room temperature for several days. The dry bone sample was then converted to powder form in a pulverizing machine. 500 mg of this bone powder were weighed out on an A&D Instruments HR-120-EC electronic scale and placed in a labeled Teflon test tube fitted with a screw on cap. 10 ml of 1 M HCl was carefully (to avoid foaming) added to the Teflon test tube containing the 500 mg sample. The Teflon test tube containing the mixture was then placed on a shaker for 20 min to remove the mineral component of the bone sample. The test tube was capped off to avoid sample loss; however, it was important that the cap was not screwed down tight, which would otherwise produce an airtight container. Pressure resulting from the chemical processes involved built up rapidly; therefore, the cap had to be loosely applied to facilitate a pressure release.

By means of a vacuum driven suction apparatus equipped with a hose and tipped with a pipette, the HCl and dissolved mineral fraction of the sample were removed from the remaining solid precipitate. Caution was taken not to suction off any portion of the sample, as this tended to happen abruptly. To neutralize the sample and raise the pH to 6 or approximately that of distilled water, the sample was left in the Teflon test tube, filled with distilled water and capped off. In contrast to the previous step, this time, tightly. The distilled

water and solid were then briefly placed on a vortex to ensure thorough dispersion of the acidic solid in the watery solution.

The mixture was then centrifuged at 3000 rpm for 5 min with a Sigma Laboratory 4K15 centrifuge. The centrifugal action resulted in the formation of a distinct pellet-like sample. Utilizing the same suctioning equipment, the distilled water solution containing the washed out HCl was removed. This step was repeated at least five times before the first litmus test was conducted. After the litmus test indicated that a pH of 6 had been reached, the solution was suctioned off.

In a step necessary to remove traces of the contaminant humic acid, the Teflon test tubes containing the pellet were filled with approximately 10 ml of a 0.125 M NaOH solution. The test tubes were capped, and once again, in such a manner as to allow built up pressure to escape, and placed on the shaker for a period of 20 hours. The test tubes containing the pellets mixed with the NaOH solution were centrifuged for 5 min at 3000rpm. The solution was suctioned off and, as before, the pellet was rinsed with distilled water, mixed briefly on the vortex and subsequently centrifuged to achieve a pH of approximately 6. This step was also repeated at least 5 times before conducting the first litmus test.

The pellet, still in the Teflon tube, was treated with 10 ml of 0.001 M HCl (pH 3). The test tubes were then placed in a warm water bath (90°C) for at least 10 hours, but no longer than 17 hours. The warm acid functioned to gelatinize the pellet by breaking the helical structure of the protein.

The collagen solute was filtered off using a glass funnel equipped with a screen filter (pore size 5 µm) and a vacuum. The solubilized gelatin was then poured into small, labeled glass vessels, leaving the remaining pellet exposed on filter. The glass vessels containing the gelatin solution were covered with a piece of perforated aluminum foil and lyophilized in a Christ Alpha 1-4 freeze drying apparatus under a vacuum bell for 72 hours at -52°C.

Taking care to avoid contamination, a 1 mg sample was weighed out on the electronic scale and wrapped in square of tin foil and placed in an Eppendorf-cup. The samples were subsequently tested at the GeoBio-Center at the LMU, Munich.

8.4 Amino acid assay

The amino acid assay was conducted to determine the protein integrity of bone collagen. Protein preservation was determined using this method proceeded on a random sample basis, principally because a complete analysis would have been too time consuming and cost ineffective. In addition, because the molar C/N ratios for collagen on the whole, fell within

the acceptable range of 2.9-3.6 (Ambrose 1993) it was decided to perform only 10 amino assay tests. Approximately 1 mg of lyophilized gelatin was hydrolyzed with 1 ml of 6N HCL for a period of 24 hours. The acid was allowed to evaporate under the ventilation hood. A solution containing 1 ml distilled water and 1 ml lithium-citrate buffer (pH 2.2) was added to the sample. 20µl of this solution were then tested in the amino acid analyzer (Pharmacia Alpha Plus, Lithium-System). This analyzer is calibrated to a precision of 0.5nmol and is also capable of detecting both physiological and microbial amino acids, and is thus an excellent indicator for biotic diagenesis.

8.5 Structural carbonate extraction

5 ml of 4% NaOCL were added to 100 mg of bone powder in a test tube and mixed on the vortex machine. The test tubes were then placed on the shaker for 72 hours. Following the initial 24 hours, the test tubes were centrifuged and the NaOCL was removed and replaced with fresh NaOCL. The test tubes were centrifuged for 5 min at 3000 rpm, the NaOCL solution removed by suction and the pellet rinsed with distilled water. The procedure of rinsing the pellet with distilled water was repeated five to six times until a pH of approx. 6 was achieved. The pellets were then treated with 5 ml of a 1 M calcium-acetic-acid-buffer (pH 4.5). The solution was then vortexed and placed on the shaker for a period of 10 hours. This step functions to remove exogenous carbonate (Koch et al. 1994). The samples were then rinsed with distilled water, using the same stepwise method of centrifugation, suctioning, and testing with litmus paper, 4-5 times, until a near-neutral pH was achieved. Glass vessels containing the carbonate fraction were then lyophilized in a vacuum for 72 hours at -52°C.

8.6 Mass spectrometry

The mass spectroscopy analysis for this research project was conducted at the GeoBio-Center at the LMU in Munich.

a) Collagen

The $\delta^{15}N$ and $\delta^{13}C$ from organic collagen samples were detected online by a mass spectrometer type Thermo Finnigan Delta plus, coupled with a CHN-analyzer Thermo Finnigan NA2500. Approximately 0.3 mg of the lyophilized samples were enclosed in Sn-capsules and incinerated with injected oxygen at roughly 1500°C in the CHN-analyzer. The resulting gas was transferred into the mass spectrometer as CO_2 or N_2 via CONFLO II-Interface and He gas as the carrier. Calibration was performed against a laboratory standard, i.e. against the standards NBS 19 and NBS 20 (for CO_2) and against N1 and N2 (for N_2).

Isotopic ratios were expressed in the conventional δ-notation relative to the PDB- and AIR-standards. In addition to the δ-values, percentage of C and N, and the C/N molar ratio were determined to control for the molecular integrity of the extracted collagen. Measurement error never exceeded 0.15‰.

b) Carbonate

The $\delta^{18}O$ and $\delta^{13}C$ from carbonate were determined utilizing a coupled Gas bench II/Delta plus (Thermo Finnigan) system, in which the samples reacted with ortho-phosphoric acid at 72°C to produce CO_2. The resulting CO_2 was then transported with helium as the carrier gas into the mass spectrometer. The isotope values were determined based on a gaseous laboratory standard calibrated to the NBS 19 and NBS 20 (for CO_2) standard set by the IAEA. The isotopic ratios were expressed in the conventional δ notation relative to the PDB standard used for solids. Measurement error did not exceed 0.1‰.

In this manner isotopes were separated and the ratio, expressed as (δ), for the samples was then calculated using the following equation, where *X and X represent the heavier and lighter isotope. The results are expressed in parts per thousand (‰).

$$\delta{*X}\ \text{‰} = (*X/X)_{standard} / (*X/X)_{sample}) \times 1000$$

8.7 Faunal samples

22 bone samples from 22 individual animals (8 domestic cattle- *Bos taurus*, 3 domestic sheep- *Ovis aries*, 2 domestic chickens *Gallus domesticus*, 6 domestic pigs- *Sus scrofa domesticus*, 1 domestic horse- *Equus caballus*, 1 domestic goat- *Capra hircus*, 1 red deer- *Cervus elaphus*) originating directly from the Volders archeological site, were analyzed for their C, N and O stable isotopic compositions using the same preparatory and analytic methods utilized for the human bones. The archaeozoological examination, including species identification and bone anatomy, were carried out by the author at the Bavarian State Collections for Anthropology and Palaeoanatomy in Munich.

9. Results

9.1 Morphological examination

The 153 individual burials discovered yielded 144 human skeletons. An additional grave discovered during road work at exactly the same site in the 1960s was given to the Ferdinandeum State Museum in Innsbruck and included in this series since it is presumed to belong to the same burial grounds. This puts the total number of skeletons examined at 145. Due to various taphonomic effects, the remaining graves contained little or no skeletal material for osteological examination or chemical analysis. The archeological findings revealed that of the 153 burial situations, 23 were oriented in a north-south direction (see Fig. 1.7), the remaining were in an east-west direction and only eight contained grave associations. The specific osteological data for each skeleton is presented in the anthropological catalog (appendix 1).

Skeletal condition

Burial surroundings varied and skeletal preservation was divided into three gross categories. In all, 37 skeletons were considered to be in good condition, 46 were listed as average, and 62 as being poor. The first half of the excavation was conducted during the late fall and early winter when snow, freezing temperatures and sudden thawing took their toll on exposed bone material. The second half of the dig was carried out in the springtime, a season characterized by brief snowfall, heavy rains, cold temperature not conducive to drying, and muddy soil. In all, the weather conditions can be described as less than optimal for good bone preservation. At least 10 burials were severely damaged by building machinery during the initial earthmoving operations and several others experienced slight damage during the course of recovery or transport. The geology of the individual burial surroundings also played a role in skeletal preservation and it was observed that skeletons interred in the gravel layers were often less well preserved because they were subject to greater mechanical destruction than those buried in soil. A minimum of 10 burials were partially destroyed by the lime pit used by construction workers some time in the past, probably during the erection of the garden wall, which itself claimed no less than 25 skeletons victim, trapped in its cement clutches.

Burial associations

Only eight burials were discovered containing grave associations, and these consisted primarily of gender related items such as combs, glass bead necklaces, rings, arm bands, small

knives and belt buckles. A detailed report to the archaeological findings is currently in preparation (Zanesco & Stadler, in prep.).

Age at death diagnosis

Human skeletons composing the archeological remains represented by the Volders cemetery consisted of 119 adult and 26 non-adult individuals, plus one for which the age was not identifiable with certainty, but assumed to be adult (burial 63). Each main age group was further subdivided into specific age categories delineated by 5 year increments. Subdivisions for the adult group begin with 20-25 years and end with >60 years. Occasionally the increment was extended to 10 years, for example, 50-60 in cases where a more specific estimation was unrealistic due to insufficient presence of age related characteristics. Adult individuals that could not be placed into a specific age category were simply listed as adult. Morphological and TCA age estimation were used in combination when necessary, for example, when the morphological age spanned 10 years and the TCA age fell in between, thus allowing for a more accurate placement into a 5 year increment. Subadult age groups include neonate, perinatal, 1-7 years, 8-12 years, and 13-19 years. A breakdown of all adult age groups is listed in Table 2, and the division for subadults is listed in Table 3.

Bone preservation presented a problem for the morphological identification of age. In 48 of the 119 adults, the age determination was limited to the description "adult".

Table 2. Breakdown of the adult age groups for the 119 adult individuals at Volders and the total number of individuals determined for each age division.

**Age**	**Total (n)**	**Age**	**Total (n)**
20-25	1	46-50	11
26-30	7	51-55	6
31-35	6	56-60	3
36-40	7	>60	25
41-45	5	adult	48

Table 3. Breakdown of the subadult age groups for the 26 subadult individuals at Volders and the total number of individuals determined for each group.

Age	Total (n)	Age	Total (n)
neonatal	3	8-12	2
perinatal	1	13-19	7
1-7 years	13	subadult	0

Palaeodemography

Age related data acquired from an archaeological skeletal series are rarely sufficient to allow the construction of a complete life table, therefore, an abridged life table containing age groups and not single ages was constructed in order to better understand basic aspects of the cycle of living and dying amongst the Volders population. The life table is a time-honored and efficient means for analyzing age-specific death rates (ASDRs), and is also referred to as survival analysis (Newell 1988). Most of the columns in the life table are calculated from the others. The Volders life table (Table 4) indicates a very high life expectancy ($e°x$) for this population, which implies not only the existence of good living conditions, adequate nutrition and excellent physical health, all factors that increase survival rates, but also reflects the optimal adaptation of these people to the rigors of the alpine environment. Relative to adults, there is a significant deficit in infants. One reason may be attributed to taphonomic processes that more readily lead to the destruction of fragile infant skeletons, however, it is more probable to assume that the lower proportion of child-bearing women directly influenced this observation. Quite rationally, the numerical relationship between the number of recovered infant skeletons in an archaeological complex is related to the number of physically mature women that lived in the population represented by that skeletal series (Grupe 1985). The age group related life expectancy distribution at Volders, especially that of the infant group, is normal when compared to the standard figures established for other early medieval European populations (Langenscheidt 1985). According to formulas provided by Herrmann et al. (1990), which are based upon Bocquet and Masset (1977), life expectancy for populations represented by archaeological skeletal series can be calculated by factoring in age groups with larger age ranges (e.g. adults over 20yrs). This is accomplished by using the equations: $D_{5\text{-}9}$ / $D_{10\text{-}14}$ and $D_{5\text{-}14}$ / $D_{20\text{-}\omega}$ where D is the total number of deaths and the numbers represent ages in years (i.e. $D_{5\text{-}9}$ is the total number of deaths in 5 to 9 year olds). In order to be representative of a normal distribution, $D_{5\text{-}9}$ / $D_{10\text{-}14}$ should be ≥ 2 and $D_{5\text{-}14}$ / $D_{20\text{-}\omega} \geq 0.1$. The Volders sample produced the Figures 8.0 and 0.08 respectively, indicating lower rate of death

in youths (10-14yrs), which is characteristic for agrarian populations, yet, too few young infants, substantiating the inference that the child deficit is not the result of taphonomic processes, but due to the low number of women capable of bearing them.

Table 4. Volders life table data including age categories (x), range in years of the individual age category (a), number of individuals for that category (D*x*), relative number of individuals who died in a specific category (d*x*), relative number of survivors (l*x*), likelihood of mortality (q*x*), total number of years between one category and the next (L*x*), total number of years remaining to be lived (T*x*) and average life expectancy (e°*x*).

x	a	D*x*	d*x*	l*x*	q*x*	L*x*	T*x*	e°*x*
0-7	8	17	177.1	1000.0	177.1	7291.6	40671.7	40.7
8-12	5	2	20.8	822.9	25.3	4062.5	33380.1	40.6
13-19	7	7	72.9	802.1	90.9	5359.5	29317.6	36.5
20-25	6	1	10.4	729.2	14.3	4344.0	23958.1	32.8
26-30	5	7	72.9	718.8	101.4	3411.7	19614.1	27.3
31-35	5	6	62.5	645.9	96.8	3073.2	16202.4	25.1
36-40	5	7	72.9	583.4	124.9	2734.7	13129.2	22.5
41-45	5	5	52.1	510.5	102.1	2422.2	10394.5	20.4
46-50	5	11	114.6	458.4	250.0	2005.5	7972.3	17.4
51-55	5	6	62.5	343.8	181.8	1562.7	5966.8	17.3
56-60	5	3	31.2	281.3	110.9	1328.5	4404.1	15.6
61-65	5	5	52.1	250.1	208.3	1120.2	3075.6	12.3
66-70	5	7	72.9	198.0	368.2	807.7	1955.4	9.9
71-75	5	5	52.1	125.1	416.5	495.2	1147.7	9.2
76-80	5	2	20.8	73.0	284.9	313.0	652.5	8.9
81-85	5	1	10.4	52.2	199.2	235.0	339.5	6.5
86-90	5	4	41.6	41.8	995.2	104.5	104.5	2.5

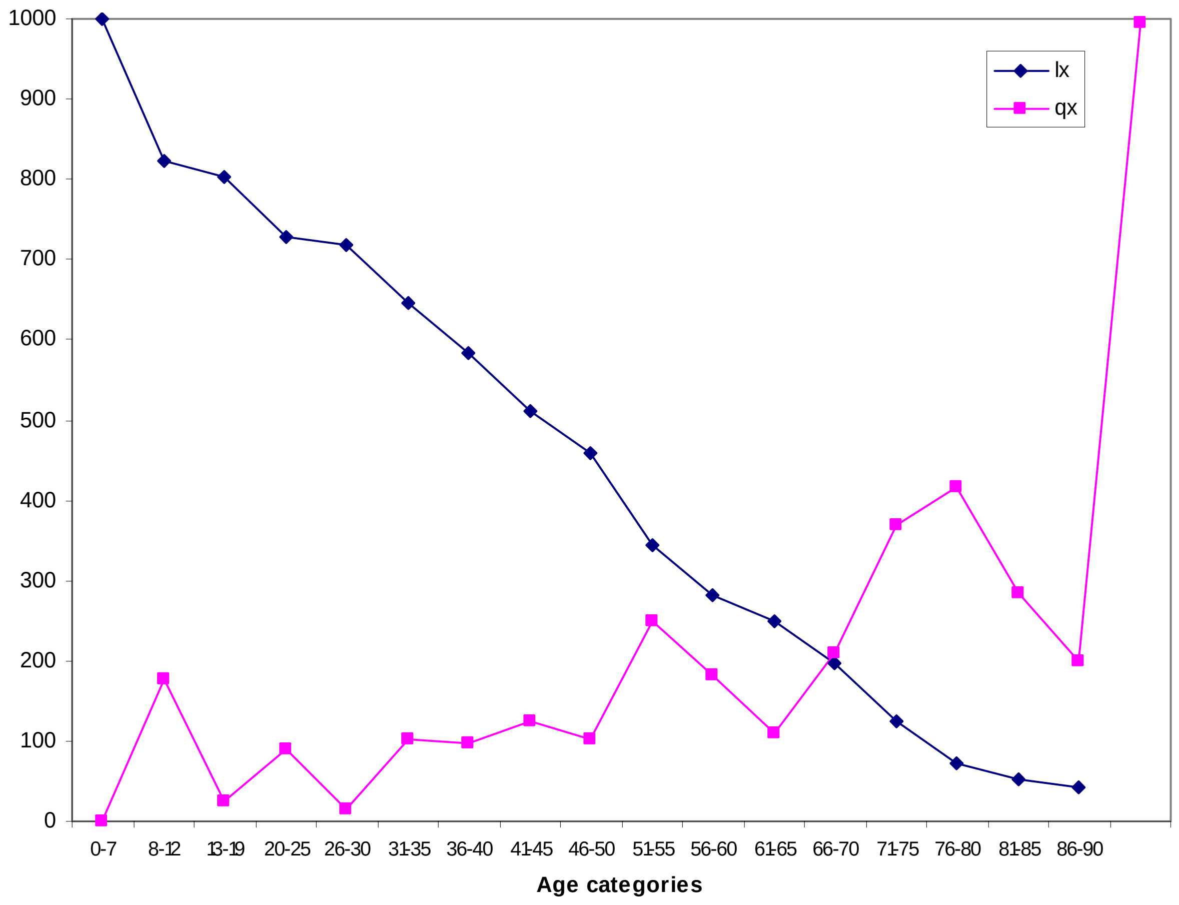

Figure 9.1. Graphic illustration of the mortality risk (qx) and the relative number of surviving individuals per category. Age groups are based both on morphological and manual TCA estimation results.

According to Figure 9.1, the qx line clearly shows the deficit of individuals in the 0-7 year age bracket. The fact that only two individuals fell within the 61-65 year age group, resulting in a drop in the qx line, probably represents an outlier. The graphic also illustrates the lack of any peaks related to increased risk of mortality for any one group, rather showing a uniform rate of death in all the groups, which gradually increases with lifespan. A striking observation was made with respect to longevity, in which 21 of the 66 adults (31.8%) reached an age exceeding 60 years.

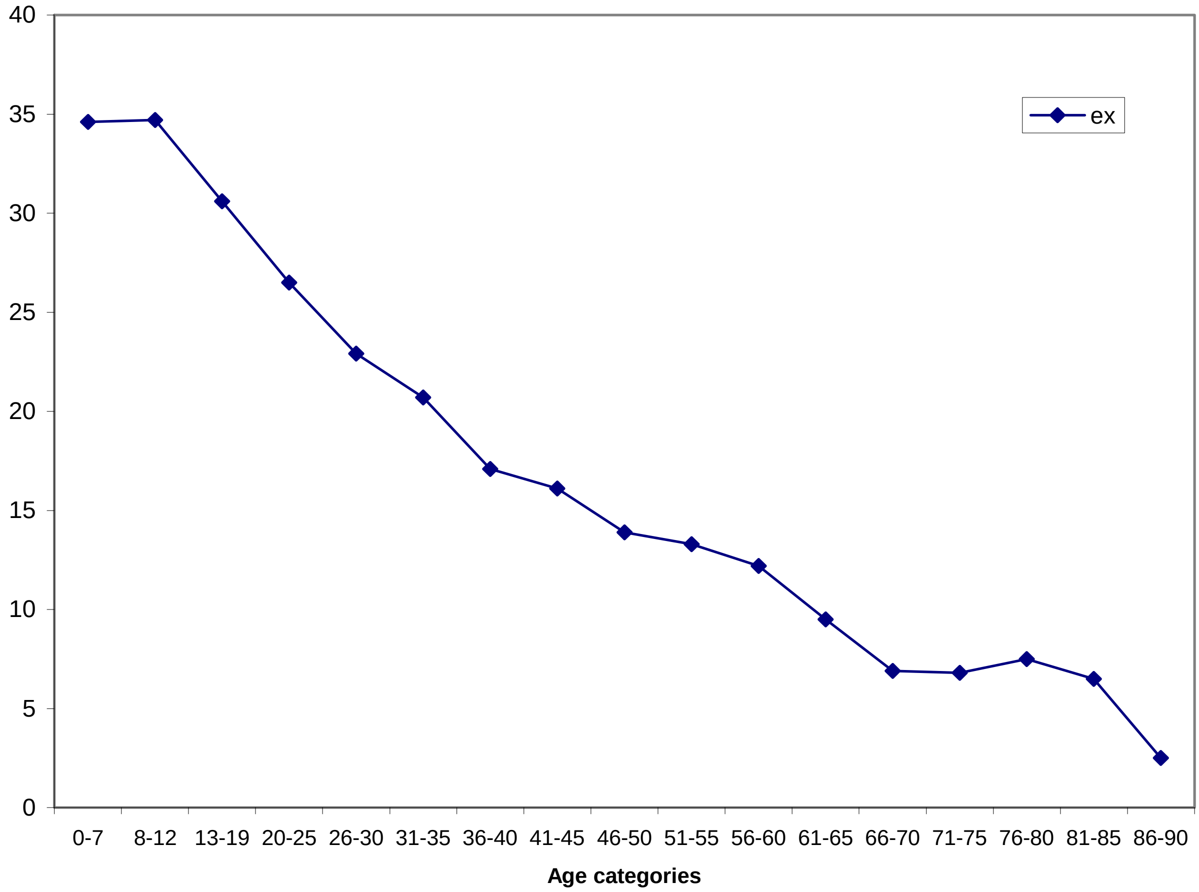

Figure 9.2. Early Middle Age life expectancy at Volders. With the exception of the deficit in infants, which can possibly be attributed in part to taphonomic processes, but is more likely due to the relatively small number of females, the curve is typical for a preindustrial living population.

Sex

A determination of sex for the adult individuals was made using standard osteological discriminatory methods. Of the 119 adults, 69 were determined to be male, 31 female, 11 were indicated as tendency male, 3 as tendency female, and 5 were not identifiable as either. The ratio of males to females is marked. If the 5 unidentified individuals are excluded and the tendency factor is calculated in, males outnumber females by a factor of over 2 to 1. The masculinity index (MI), which is calculated using the equation [males (n) x 100 / female (n)], is regularly employed to indicate the predominance of men or women within a group (Herrmann et al. 1990). Calculations resulting in a figure greater than 100 indicate a male predominance and less than 100 a predominance of females. The Volders MI for juveniles and adults was 218.4 and reflects the overwhelming prevalence of males in the population. There is no indication that the extremely high rate of morbidity amongst males suggests a preferential burial area or that greater male attrition is due to work related activity or disease.

Neither could be substantiated by the palaeopathological examination. On the contrary, the palaeodemographic figures show otherwise and it is probable that Volders represents a population makeup reflecting male dominated work practices.

Of the 26 subadult individuals, 3 were found to be male, 5 female, one with a tendency towards male and 17 for which the sex was not discernable. The large figure in this last category reflects the poor level of skeletal preservation in young individuals, especially infants. Of the nine subadults between the age of 8 and 19, a sex determination was achievable in eight cases. Of the 17 subadults under the age of 7 years, 16 were not determinable and one was recorded as tendency male.

Height

The mean height and standard deviation for adult males was 168.6 cm with a standard deviation of 5.58. The mean height for females was 160.4 cm with a standard deviation of 4.65. Individual heights for adults and subadults are listed in the anthropological catalog. Large individuals that were at least one standard deviation above the mean are listed in Table 5 together with variables that may indicate an elevated status level. Additional aspects related to height are listed in the discussion section.

Table 5. Tall males and females listed with characteristics potentially associated with higher status and better nutrition. (ew: east/west, ns: north/south).

Burial	Sex	Height	Age	Robust stature	Burial orientation	Artifact	$\delta^{15}N$
25	m	175	50-60	yes	ew	yes	10.34
35	m	180.1	50-60	yes	ew	no	10.19
49	m	175	26-30	yes	ew	no	9.47
50	m	181.5	31-35	yes	ew	yes	11.5
70	m	176.1	46-50	no	ns	no	10.70
84	m	174	adult	yes	ns	no	9.87
86	m	174	26-30	yes	ew	yes	--
100	m	175.7	36-40	yes	ew	no	11.70
108	m	177.3	40-50	yes	ew	no	9.86
110	m	174.2	26-30	yes	ew	no	9.64
116	m	177.2	>60	yes	ew	no	9.34
121	m	174.2	adult	yes	ew	no	--
153	m	175.5	50-60	yes	ew	no	10.84
26	f	167.7	26-30	no	ew	no	9.05
40	f	171.3	15±36m	no	ns	yes	--
60	f	164.7	adult	no	ns	no	9.61
95	f	169.7	adult	no	ew	no	--
96	f	164.4	adult	no	ew	no	--
101	f	165.1	46-50	yes	ew	no	11.23
129	f	164.5	26-30	no	ew	no	10.55
146	f	164.5	adult	no	ew	no	9.6

Palaeopathology

An examination of the remains for pathologies revealed numerous degenerative effects related to activity patterns and aging. Several bone fractures and other evidence for traumatic injury could be detected on the skeletal remains. There was, however, little evidence for bone specific infections or other systemic diseases. A mere two cases of osteomyelitis were

diagnosed, neither of which was severe. Eight cases of bone lesions, all of which were small and related to non-systemic infections could also be observed.

The complete absence of any signs of trauma related to warfare, combat or other forms of aggression is striking for the early medieval period and adds support to the assumption of peaceful cohabitation between the different Inn Valley groups. Although it should be noted that a large number of skeletons were damaged or partially destroyed, a situation which could lead to the coincidental loss of those skeletal elements that carried evidence for violence, such as the cranium or ribs.

A total of 25 skeletons showed signs of differing degrees of osteophytosis. Combined with the additional fact that 33 individuals were noted to have a particularly robust stature with pronounced points of muscle attachment, this would seem to indicate that at least a portion of the Volders population was a physically active one, perhaps engaged in agricultural work. Only six individuals were found to show evidence of bone fracture, a relatively low rate of bone injury for an active people.

Approximately 15% of the preserved dentitions showed evidence for periodontal diseases such as caries, periodontitis or peri-apical abscesses. Tooth wear leading to various degrees of tooth crown loss were frequently observed and is perhaps the main reason for the relatively low rate of periodontal diseases such as caries. Individual cases of periodontitis and levels of tooth wear are listed in the catalog (see Appendix 1).

9.2 TCA age at death estimations (automated and manual)

The teeth from 65 adult males and females out of total of 119 met the standards required and were sampled. Discounting non-adults, the method was not applicable in 54 cases. To satisfy scientific curiosity and test the reliability of the TCA method, 4 juveniles were also included in the analysis. Histological tooth root sections initially underwent manual TCA analysis. It was during this procedure, that the still frames used for the automated TCA were made. The results of both TCA methods and the morphological age at death estimation are presented in Table 6. The age estimations produced by each were compared to one another and subsequently compared to the age at death estimations according to the morphological examination of the skeleton.

Table 6. Age at death of sampled skeletons according to morphological, auto-TCA and man-TCA examinations.

Burial	Sex	Morph. Age	Tooth	Eruption age	Auto-TCA	Max. count	Auto-TCA age est.	Man-TCA	Man-TCA age est.
2	f	**>60**	pm (13)	12	87	106	**99**	78	**90**
6	tm	**50-60**	pm (21)	12	48	62	**60**	42	**54**
10	m	**60-70**	pm (12)	12	60	73	**72**	60	**72**
11	f	**50-60**	pm (29)	12	15	20	**27**	35	**47**
14	f	**60-65**	pm (21)	12	65	72	**77**	73	**85**
15	f	**31-35**	pm (21)	12	25	39	**37**	25	**37**
16	m	**46-50**	pm (21)	12	36	50	**48**	28	**40**
17	m	**>60**	c (6)	12	79	82	**81**	67	**79**
18	m	**50-60**	pm (29)	12	46	59	**58**	37	**49**
23	m	**50-60**	m3 (32)	15	51	67	**66**	52	**67**
24	m	**50-60**	pm (29)	12	78	98	**90**	74	**86**
25	m	**50-60**	pm (20)	12	47	65	**59**	46	**58**
26	f	**26-30**	pm (20)	12	25	33	**37**	30	**42**
27	m	**51-55**	pm (28)	12	26	32	**38**	30	**42**
35	m	**50-60**	pm (12)	12	55	70	**67**	61	**73**
36	m	**46-50**	pm (28)	12	36	48	**48**	31	**43**
37	m	**60-70**	m3 (17)	15	not countable		**no result**	55	**70**
40	f	**15±36m**	pm (21)	12	not countable		**no result**	5	**17**
42	m	**60-70**	pm (29)	12	28	34	**40**	33	**45**
43	f	**30-40**	pm (21)	12	19	27	**31**	20	**32**
45	m	**60-70**	pm (29)	12	77	90	**89**	60	**72**
48	m	**50-60**	pm (21)	12	41	53	**53**	36	**48**
49	m	**26-30**	i (10)	10	27	40	**37**	25	**35**
50	m	**31-35**	pm (28)	12	27	34	**39**	22	**34**
52	m	**30-40**	pm (21)	12	45	56	**57**	40	**52**
53b	m	**30-40**	pm (28)	12	31	48	**43**	30	**42**
54	m	**40-60?**	m2 (15)	8	76	87	**84**	60	**68**
56	f	**50-60**	pm (20)	12	46	53	**58**	42	**54**
57	m	**>70**	pm (21)	12	51	65	**63**	60	**72**
59	m	**30-40**	pm (21)	12	31	41	**43**	28	**40**
61	m	**30-40**	pm (21)	12	26	44	**38**	23	**35**
65	m	**36-40**	pm (20)	12	24	30	**36**	32	**44**
66	m	**36-40**	pm (28)	12	35	45	**47**	38	**50**
67	m	**60-70**	pm (28)	12	61	84	**73**	66	**78**
68	m	**40-50**	pm (21)	12	not countable		**no result**	26*	**38**
70	m	**46-50**	pm (21)	12	41	55	**53**	37	**49**
71	f	**26-30**	pm (20)	12	19	27	**31**	14	**26**
73	f	**30-40**	pm (21)	12	28	38	**40**	20	**32**
74	f	**60-65**	pm (21)	12	46	59	**58**	47	**59**
79	f	**50-60**	pm (5)	12	30	37	**42**	27	**39**
81	m	**31-35**	pm (20)	12	56	87	**68**	38	**50**
86	m	**40-50**	pm (12)	12	15	22	**27**	14	**26**
90	m	**40-50**	pm (4)	12	49	59	**61**	46	**58**
91	m	**60-70**	pm (28)	12	62	77	**74**	50	**62**
93	f	**46-50**	pm (12)	12	27	43	**39**	34	**46**

98	m	**15±36m**	pm (29)	12	15	21	**27**	2**	**14**
99	f	**30-40**	pm (21)	12	22	31	**34**	26	**38**
100	m	**36-40**	pm (20)	12	34	47	**46**	38	**50**
101	f	**40-50**	pm (12)	12	28	35	**40**	30	**42**
103	m	**31-35**	pm (5)	12	15	26	**27**	15	**27**
104	f	**16±24m**	pm (28)	12	11	18	**23**	4**	**16**
105	f	**50-60**	pm (28)	12	53	65	**65**	50	**62**
108	m	**40-50**	pm (29)	12	34	50	**46**	38	**50**
110	m	**26-30**	pm (4)	12	24	36	**36**	15	**27**
112	m	**55-65**	pm (21)	12	100	111	**112**	76	**88**
113	m	**65-70**	pm (28)	12	46	60	**58**	56	**68**
114	f	**65-70**	pm (20)	12	63	78	**75**	54	**66**
116	m	**60-70**	pm (21)	12	59	74	**71**	42	**54**
123	f	**18-19**	pm (20)	12	17	19	**29**	6**	**18**
129	f	**26-30**	pm (20)	12	25	33	**37**	18	**30**
132	m	**36-40**	pm (28)	12	28	37	**40**	32	**44**
135	f	**50-60**	pm (13)	12	18	29	**30**	14	**26**
139	f	**40-50**	pm (29)	12	59	70	**71**	60	**72**
144	m	**60-70**	pm (21)	12	59	71	**71**	57	**69**
145	m	**60-70**	pm (29)	12	66	81	**78**	44	**56**
148	m	**50-60**	m1 (15)	8	97	109	**105**	80	**88**
151	m	**30-40**	pm (21)	12	46	56	**58**	43	**55**
153	m	**50-60**	pm (28)	12	36	51	**48**	45	**57**
19152	m	**>60**	pm (28)	12	66	77	**78**	55	**67**

(Key to Table 6)

All ages are in years.

Teeth are identified as pm (premolar), m (molar), c (canine) or i (incisor) with the numeration specification 1-32, beginning at the 3rd molar of the upper right quadrant moving clockwise and ending at the 3rd molar of the lower right quadrant.

The category “max. line count” lists the greatest number of lines counted by the automated TCA method.

Auto-TCA refers to the automated TCA mode and includes the number of lines counted most often by this procedure. These totals were used to calculate the age at death estimations.

Man-TCA refers to the manual TCA method and indicates the maximum number of lines counted by hand under the microscope.

* Represents the minimum number of lines, since the image was incomplete due to diagenetic destruction. The estimated number of lines obscured by the destroyed segment of the image totals approximately 10. The estimation is based on a comparison with the number of lines in an area of equal width where the lines were clearly visible and countable. The individual was more likely in his late forties.

** An example for the automated procedure’s tendency to overshoot the line count calculation in tooth samples originating from juvenile individuals.

Comparison of the manual and automated TCA methods

The actual count in both procedures began starting from the line directly following the one representing the point of tooth eruption (right after the CDJ) in an area on the displayed image where the individual lines were easily discernable.

One immediately obvious dilemma with the automated counting method was that it produced numerous, widely varying line count totals for just one vector of one tooth thin section. Following the automated counting procedure, the total number of lines appeared with the actual number of times that particular total was read by the software on a printed readout. Troubling disparities in the count totals often accompanied a scan. This was explainable primarily because of the variable readability of lines along any given vector.

This particular circumstance also posed a continuous problem for the manual line count. During the manual examination, the clarity of the lines invariably diminished at some point making it necessary to browse laterally along the image to find a section with adequate image clarity, rediscover the last line read at this adjacent area, and proceed with the counting. Burial 35, by no means an exception, exemplified the variability encountered by the automated method, producing count totals ranging between a maximum of 70 and a minimum of 41, albeit each was counted only 4 and 5 times respectively. The line count totals 54 and 55 on the other hand, were counted by the computer 30 and 34 times respectively. The problem arose, if the maximum number of lines counted should be used or the count total found by the software scan to exist most often. The fact is, either the lines are there or they are not. Since this software is designed to eliminate or ignore so called “doubling” effects, a cementum developmental process generating twice the number of lines actually representative of the age (Cipriano-Bechtle et al. 1995), and other figments which might lead to erroneous results, then it would seem clear that the maximum line total is verifiable and authentic, and is indeed the numerical total that should be added to the eruption age in calculating the ultimate age at death. The problem here is that some exceedingly high ages were computed. For example, a maximum of 111 lines was counted for burial 112, which would theoretically yield an age at death of approximately 123 years and with that, probably set a new medieval longevity record. This is no doubt a certain indication of the software’s apparent inability to avoid counting image abnormalities such as the doubling effect mentioned previously. A manual scan of this same slide produced a count of 76 dark incremental lines, an impressive total, yet curiously nearly 50 lines less than the maximum automated computation. This supports the assumptions made by Czermak et al. (2006), who advise that the maximum line count is not

to be used and the so-called "mode" or line count computed most often be used for the age estimation calculation.

Burials 98, 104 and 123 (Fig. 9.3) served to accentuate this concern. All three were juvenile individuals morphologically aged at 15, 16 and 18 years respectively. Although examinations of the stages of skeletal and dental development in these three cases were more than sufficient to establish an age at death diagnosis, tooth samples were nonetheless taken to test the accuracy of the TCA method. The manual count yielded only 2 lines for burial 98, 4 for burial 104 and 6 for burial 123, or estimated ages of 14, 16 and 18 years, thus coinciding almost perfectly with the age at death calculations determined by the skeletal analysis. The automated counts on the other hand, were grossly inaccurate, producing maximum line counts of 21, 18 and 23 or ages at death of around 33, 30 and 35 years respectively.

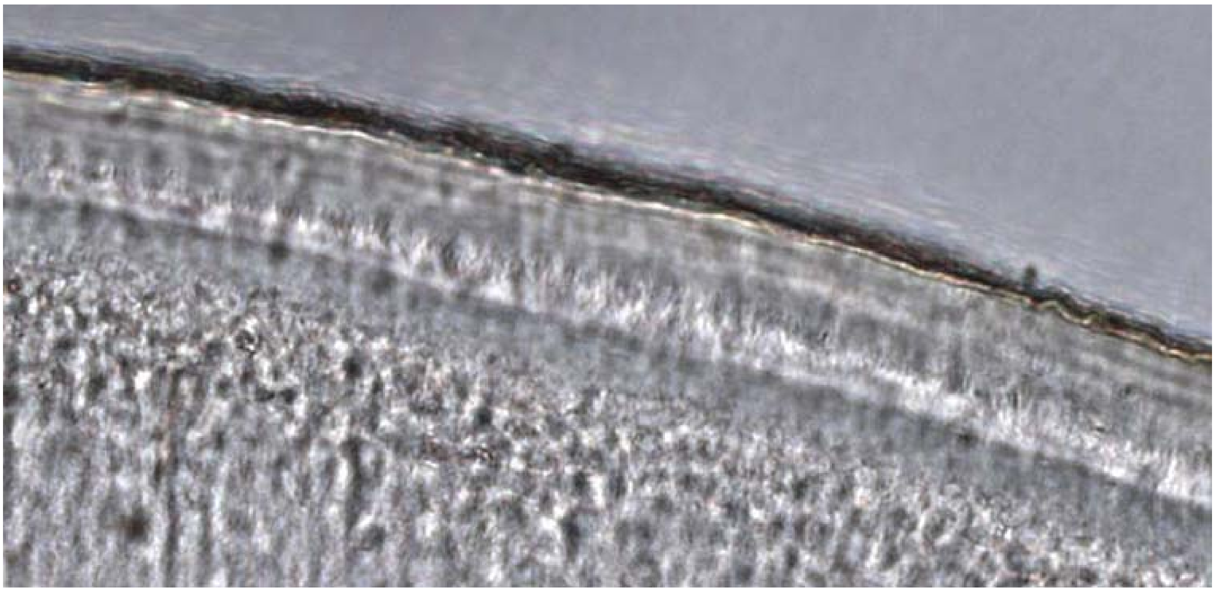

Figure 9.3. Burial 123, a juvenile female approximately 18 years of age.

The age estimation in years according to the mode for these three individuals was 27, 23 and 29 and is an example for the automated procedure's tendency to overshoot the count when an image displayed only a small number of incremental lines. A fourth juvenile (burial 40), morphologically diagnosed at 15-16 years of age, was not analyzable by auto-TCA, yet produced a manual count of 5 lines or an age of approximately 17 years, further evidence for the considerable effectiveness of the man-TCA method in determining chronological age.

One unavoidable drawback to the automated procedure is that it relies on still images (here a tif format). Occasionally it proved unavoidable to produce an image of less than mediocre

quality because thin section surface irregularities rendered it impossible to capture the entire image in focus. During a manual count, the observer has the advantage of being able to adjust the image focus on the microscope continually and so minimize the risk of including doubled lines, blotches or shadows in the count at the same time exercising a bit of precautionary wisdom. It was seldom the case that all of the lines on the entire image were in focus at the same time and focus adjustment always proved useful in teasing out lines from portions of the image that were unclear. Burial 11 presented one of the rare occasions that the manual count exceeded the automated count by some margin and was a good example of the role image clarity plays in the latter. The still image was only of moderate quality leaving the automated count hampered, yet under the microscope the section was easily analyzable through steady focus adjustment as counting proceeded from one side of the cementum to the other. A suggestion for an alternate counting method in the event still images of poor quality are available, would be to count 5 or 10 clearly visible dark lines and measure the distance these 5 or 10 lines span using a compass. By then walking the compass along the image linearly, the same way as it is done on a geographical map, and multiplying the number of compass steps by the line increment chosen, one could achieve an approximation no less inaccurate than some present attempts have proven. The problem here is that cementum appears not always to form in regularly thick layers, as they are influenced by physiological and perhaps environmental factors. Therefore, observer discretion would be advised when employing this particular alternative.

It appeared that very large numbers of cementum layers (older individuals) and very small ones (juveniles), were accompanied by the tendency of the computer to overshoot the count. This discrepancy was unequivocally verifiable only in the instances where teeth from juveniles were sampled, an age category established previously through skeletal analysis. Figure 9.4 shows two images of thin sections taken from the premolar teeth burials 98 and 104. The very small number of visible incremental lines clearly shows that both individuals were still in their teenage years.

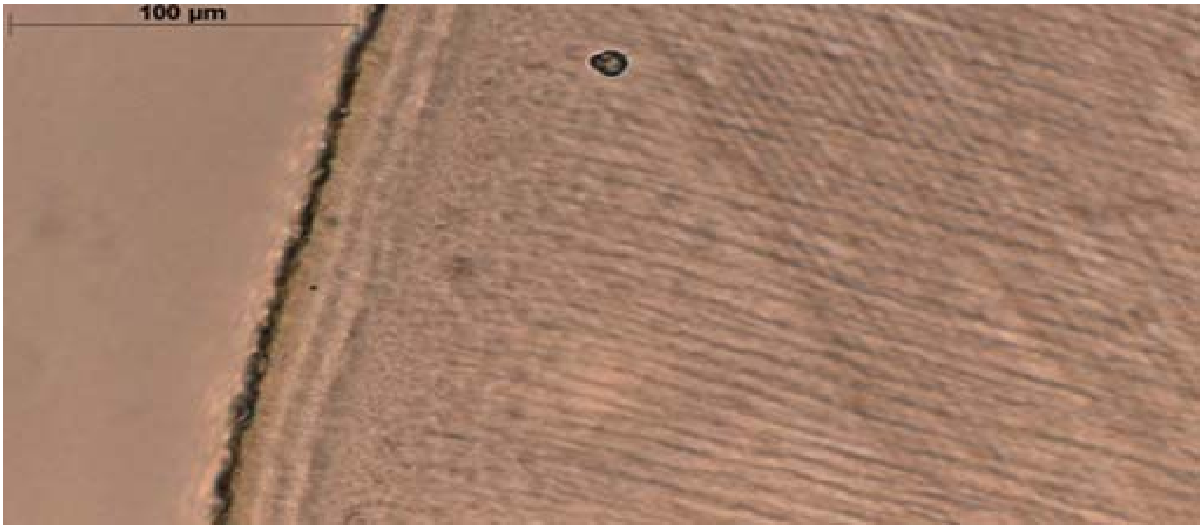

(a)

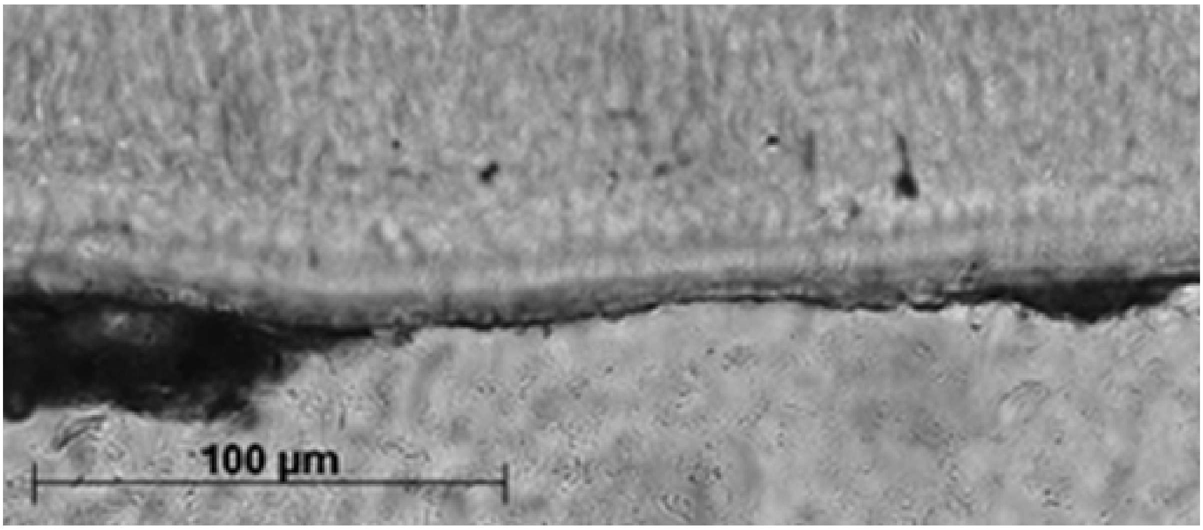

(b)

Figure 9.4. Thin sections from burials 104 (a) and 98 (b) both exhibiting clearly definable incremental lines, which substantiated the morphological age at death approximation of 16 and 15 years of age.

Furthermore, it appeared that different cross sections performed on a specific portion of the cervical third of the tooth root, could have varying numbers of cementum layers. Burial 108 is a good example to highlight the importance of making numerous thin sections to account for this situation. Of the 12 thin sections made, 11 revealed no more than 15 to 18 lines in both counting modes. A twelfth section, however, showing no structural abnormalities, yielded 38 lines in the manual count and 34 in the automated, with a maximum of 50. This translates into a difference of approximately 20 years lifespan in this one section compared with the other 11. Because cementum is soft and thinnest cervically, it can be removed by abrasion when gingival recession exposes the root surface to the oral environment during a lifetime. This

might explain the observation of differing layer counts at roughly the same area of the root. One area of the image that consistently proved problematic not only for the automated but also the manual procedure was the frequently darkened outer layer, usually presenting as a thickened, ragged band. It was often observed in more translucent portions along the length of this dark band, that up to 5 individual layers were masked by the dark pigment (humic acid residue).

Automated TCA vs. manual TCA age estimation results

The manual TCA results were compared with those produced by the automated TCA approach utilizing a Pearson-product-moment statistical test to determine if differences existed between the ability of the two methods in establishing age at death estimates.

In three instances the automated procedure was not able to calculate a line count due to poor image clarity. All three were eliminated from the test. The correlation coefficient was ($r = .37$) indicating a very low positive relationship, and demonstrating that substantial disparities between the outcomes of both methods are present.

Fig. 9.5 illustrates the outcomes of both TCA methods together. With the exception of the first age category representing juveniles (13-20 yrs), ages were subdivided into categories spanning 5 years. Chronological age estimations produced by both TCA examinations were inserted into these respective categories. Ages that fell on the cut between two categories were arbitrarily always included in the higher age classification, for example, an individual estimated to be 25 years of age is put into category 25-30 yrs. and not 20-25 yrs.

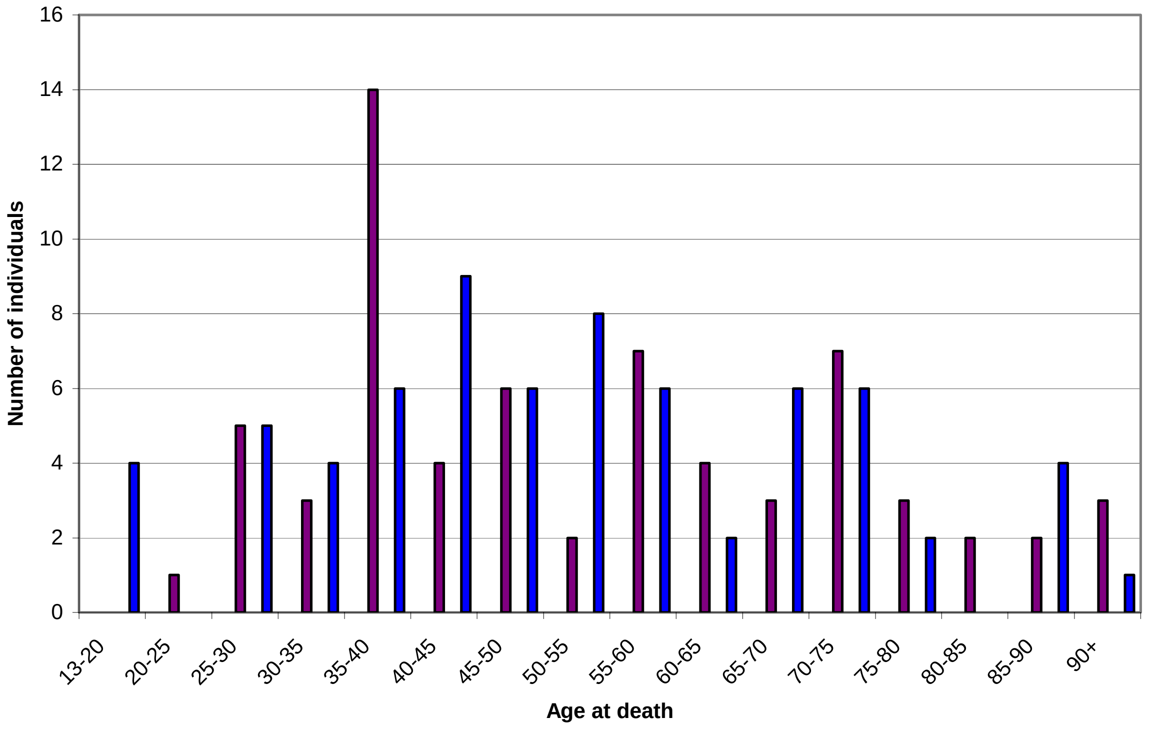

Figure 9.5. Bar graph illustrating the estimations of age at death for all 69 individuals sampled as calculated by the automated (n = 66) and manual TCA (n=69) procedures. Auto-TCA “mode values” (line counted tallied most often) appear in violet and the manual TCA age at death estimations are in blue.

Morphological vs. TCA age estimates

A comparison between the age at death estimations acquired by morphological examination with the estimations resulting from the manual TCA analysis was made and yielded some promising results. The general correlation between the two methods was, on the whole, quite good. More specifically, in the majority of cases adults were similarly identified by both methods as early, middle or late adults. Figures 9.6 and 9.7 illustrate the comparison between the two methods using age categories of 5 and 10 year increments respectively. For comparative purposes, it was necessary to devise general age categories of up to 10 years because of the difficulty in ascertaining a more precise age at death in many of the morphologically based examinations. In order to facilitate a statistical analysis, a median age was determined for the morphological age estimates attained for each individual. Juxtaposed to the individual manual TCA results, a Pearson-product-moment test yielded the very high positive correlation coefficient of ($r = .93$), indicating a close relationship between the outcomes of the two methods.

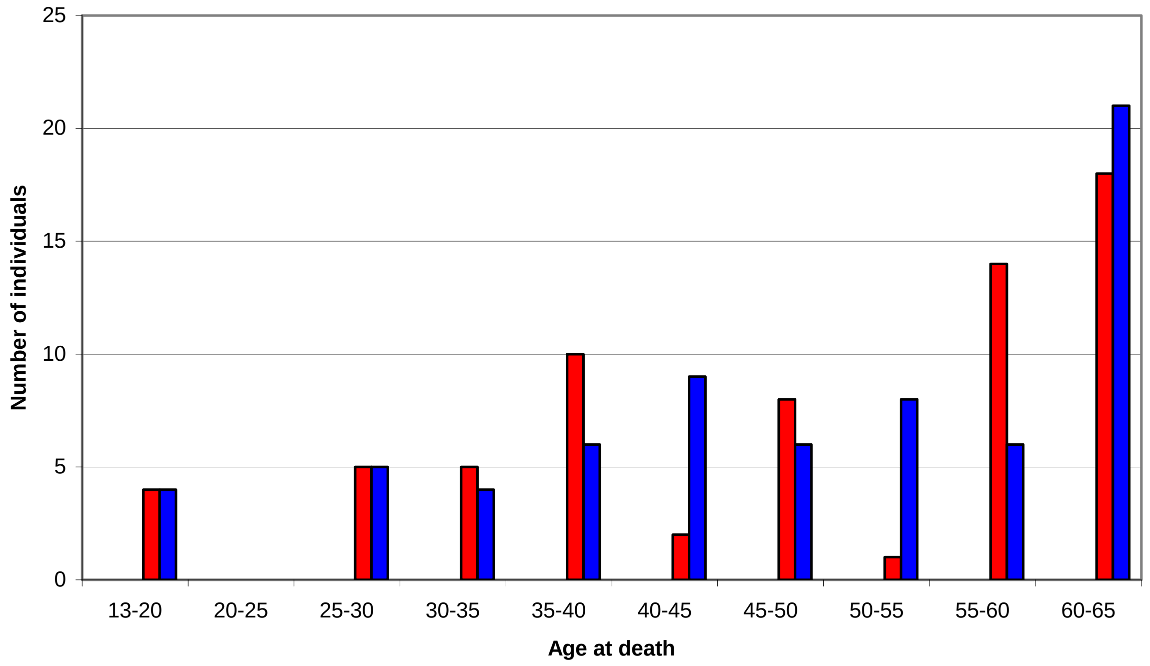

Figure 9.6. Comparison of manual TCA (blue) and morphological (red) age at death estimations using 5 year increment age categories.

A noticeable pattern is exhibited in Fig. 9.6, in which the morphological ages (red columns) are systematically higher than the TCA ages in the second half of each decade after 30 years of age (35-40, 45-50, 55-60). The TCA ages show a similar pattern in the first half of the same decades with the exception of 30-35 (where they are nearly the same and 60-65 where the pattern continues). When the age increment is increased to 10 years, these differences become less apparent and the age estimations from both procedures correlate almost identically (see Fig. 9.7).

It can be surmised, that the morphological method is prone to underestimation of the age at death or that the TCA method overestimates the ages. Because of the biological changes incurred upon the skeleton during a lifetime, changes that in part form the basis for morphological examinations for determining age, it is conceivable that the morphological age estimations are subject to inaccuracies associated with variable degrees of expression of these specific bone alterations or errors in interpretation of these changes. Ideally, the incremental lines in AEFC are not influenced by lifestyle, for example, back breaking fieldwork which can leave their traces on the vertebrae and be falsely assumed to indicate older age in the

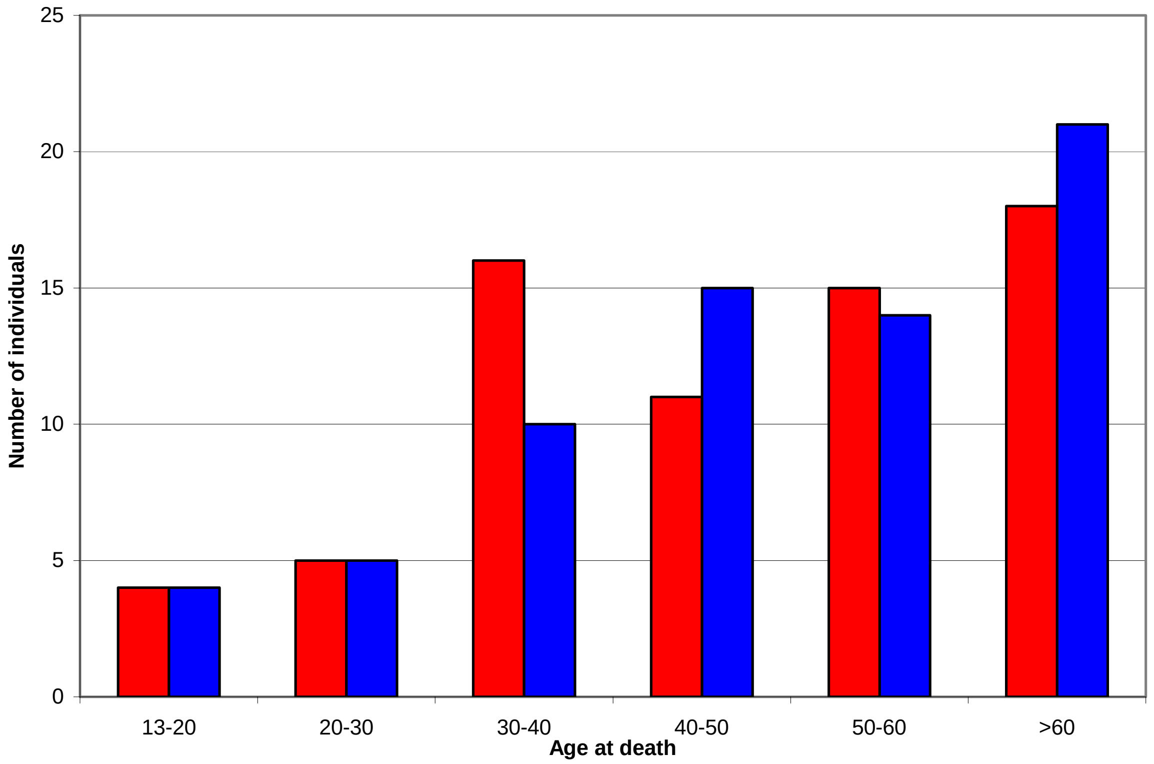

Figure 9.7. Comparison of the manual TCA (blue) and morphological (red) age at death estimations using 10 year increments. The diagram illustrates the comparatively similar results acquired between the morphologically won age at death estimates with those acquired by the man-TCA method.

morphological diagnosis, and therefore represent a criterion for the age estimations that remain unaltered by such external biological activities.

In the middle age range (30-50 yrs.), the auto-TCA age estimates based on the "mode" coincide very well with the morphological results, although according to Fig. 9.8, the method does appear to propel the age estimates in the youngest and oldest age categories in the positive direction. This is most obvious in the first and last age categories.

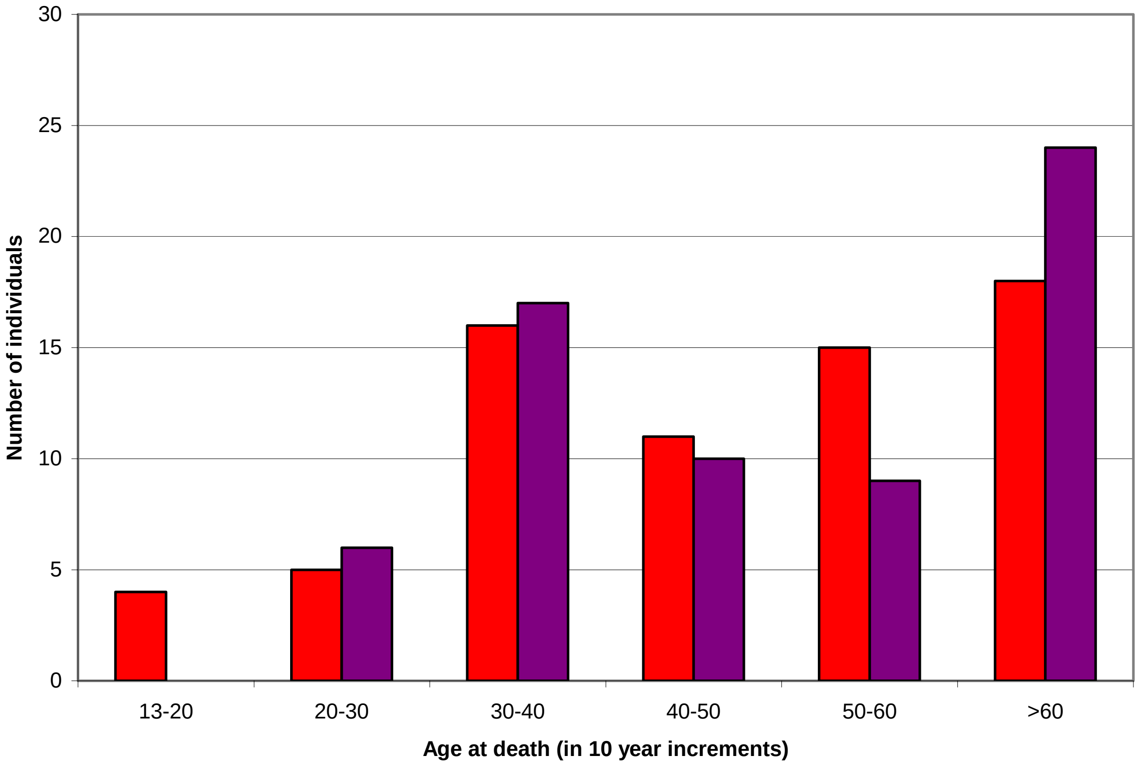

Figure 9.8. Comparison of the automated TCA (violet) age estimations using the “mode“ and the morphologically (red) won age at death estimations. Note the absence of auto-TCA individuals in the 13-20 year category.

For the purposes of providing an illustrative overview, Figure 9.9 shows all three methods together using 10 year increments. The diagram depicts a relatively good complementation between the three methods. It would appear that in cases in which a very young or very old age is suspected, that the TCA method, if conducted, should be done so manually to avoid age overestimation.

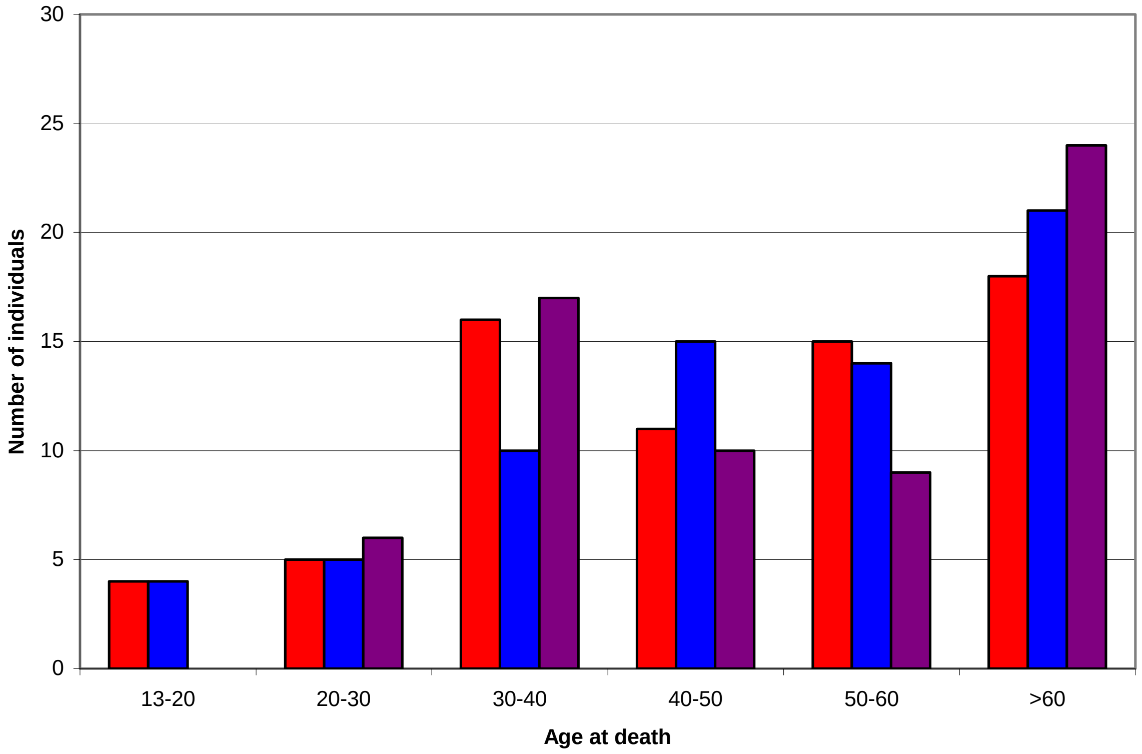

Figure 9.9. A comparison of the age at death results established by all three methods using 10 year increments. Manual TCA is indicated in blue, automated TCA in violet, and morphological age estimates are in red.

As illustrated in Figure 9.10, when the "maximum" number of lines counted by the automated procedure was calculated into the age at death estimation for this particular method, no less than 37 individuals, more than half of those tested, are found to be over 60 years of age. A breakdown of the age groups over 60 into 10 year increments would seem to indicate a tendency for this method, when using the maximum line count, to overestimate the ages in the older groups. 9 individuals fell between the range of 60-70 years, 8 between 70-80, 9 between 80-90, 6 between 90-100, and 5 were estimated at over 100 years of age (102, 110, 117, 118, 123 years respectively). Likewise, the conspicuous lack of any individuals under the age of 30 years also indicates that younger ages are drastically overestimated.

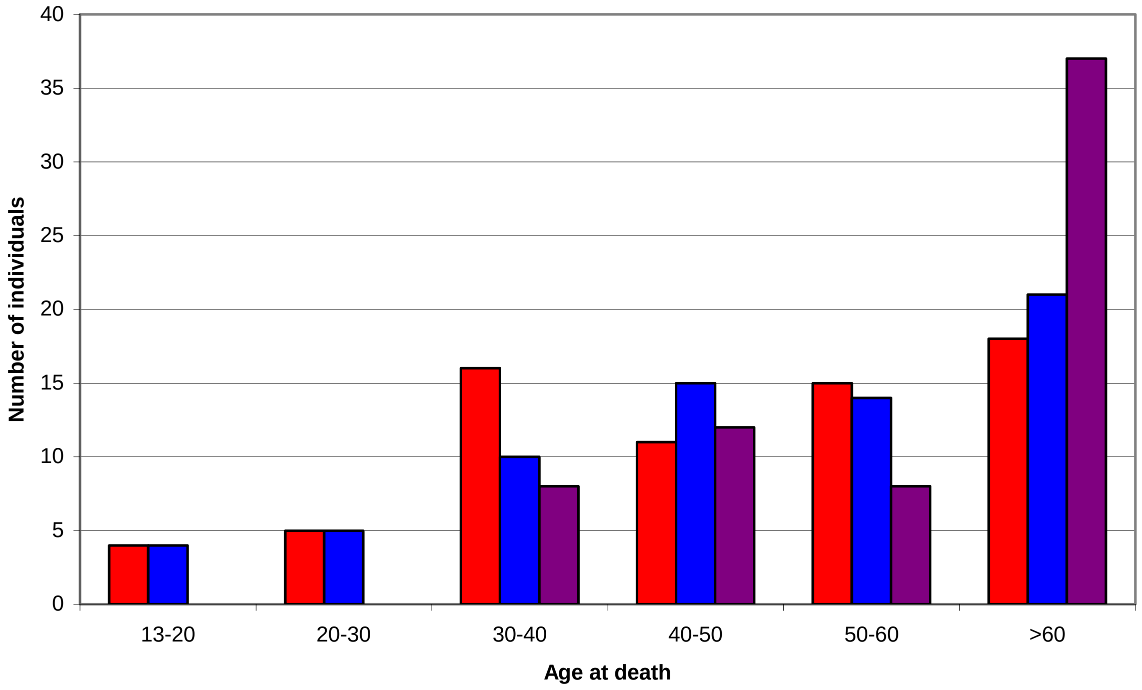

Figure 9.10. Diagram depicting the automated TCA (violet) age at death results when the "maximum" number of incremental lines counted is used in the auto-TCA calculation compared to the manual TCA (blue) and morphological (red) methods. This figure clearly illustrates why the maximum line count computed by the auto-TCA procedure should be avoided when making the age at death estimations. Young individuals are completely lost and are transformed to middle aged people and the very old become far too numerous.

Interestingly, a review of the skeletal material following the conclusion of this study revealed the importance of a combined effort of the morphological and histological methods. Several burials serve as notable examples, like 86, 90, 139, and 103, of which the first three were morphologically aged at 40-50 years and the later at 30-35 years. The manual TCA results for the first two were 26 and 58 respectively. Osteological features overlooked during the initial morphological examination of burial 86 (incomplete apophyseal fusion of the innominate bone) led to a reevaluated age estimation of 25-30 years. Burial 90 showed evidence for an age older than originally estimated due to features characteristic for older aged individuals such as fusion of the manubrium and xyphoid process to the corpus sterni that were not taken into consideration. The same situation applies to burial 139, in which the pubic symphysis surface was found bagged separately with other small fragments of the skeleton after the initial morphological examination. Together with an examination of tooth wear, the morphological age estimation was found to be considerably older than originally diagnosed. Burial 103 had a TCA age estimation of 27, which was fairly close to the original

morphological age of 30-35. A review of the catalogue showed a discrepancy between the recorded age and the osteological documentation, which indicated a younger age between 25-30 years because of non-fusion of the clavicular medial epiphysis and incomplete fusion of the iliac crest apophysis. The original data was left as is, the newly calibrated results, however, speak even more favorable for the TCA method, not only as a backup, but also as a primary method for age estimation.

9.3 Collagen integrity

The reliability of collagen based results depends in large on the integrity and molecular balance of collagen carbon ($\delta^{13}C$) and nitrogen ($\delta^{15}N$), which is established during isotopic analysis and known as the molar C:N ratio. The C:N ratio for each corresponding bone sample is listed in Table 10. In addition, random samples of collagen were tested for their amino acid compositions and compared with standards indicated by Ambrose (1993). Individual values are listed in Table 7 below and a graphic display of the peaks associated with these values is illustrated for burial 25 in Figure 9.11.

Amino acid composition

Ten random samples were conducted out of the 145 available skeletons. Results of these ten assays are listed in Table 7 below. C:N ratios from these 10 samples were all within the 2.6 to 3.9 range.

Table 7. Listing of the results of the Volders amino acid assay. Standard according to Ambrose (1993).

**Conc. % AA**	**Burial 27**	**Burial 42**	**Burial 50**	**Burial 56**	**Burial 65**	**Standard**
OH-pro	10.6	8.1	9.8	9.2	9.6	8.9
Asp	3.9	1.6	3.1	3.4	3.6	4.4
Thr	1.9	0.2	1.7	2.1	2.1	1.7
Ser	1.7	0.3	1.6	1.7	1.8	3.6
Glu	5.1	4.6	4.2	4.5	4.8	7.4
Pro	17.2	23.4	17.8	17.3	17.5	13.0
Gly	23.4	27.3	25.0	23.6	24.2	33.4
Ala	14.4	14.3	15.5	15.0	15.2	11.2
Cys	0.2	-	0.1	0.1	0.2	-
Val	3.2	3.3	2.6	2.9	3.1	2.5
Met	0.6	-	0.3	0.1	0.5	0.5
Ile	1.2	0.8	0.9	1.0	1.3	0.9
Leu	4.0	2.6	3.6	3.7	3.9	2.3
Tyr	-	0.4	0.1	0.4	0.5	0.3
Phe	2.1	0.1	1.8	1.9	1.9	1.2
His	0.6	0.3	0.2	2.6	0.2	0.5
OH-lys	0.4	0.2	0.4	0.3	0.4	0.5
Lys	3.4	3.0	3.4	3.5	3.3	2.7
Arg	5.9	9.4	7.7	6.6	5.9	5.0
nmolAS/40µl	378.5	201.7	321.2	347.8	334.4	

**Conc. % AA**	**Burial 18**	**Burial 3**	**Burial 149**	**Burial 25**	**Burial 44**	**Standard**
OH-pro	16.4	7.8	8.2	4.9	6.3	8.9
Asp	2.7	2.9	3.0	2.4	2.5	4.4
Thr	1.6	1.4	1.3	1.2	1.1	1.7
Ser	1.5	1.2	1.3	1.2	1.0	3.6
Glu	3.1	3.8	4.3	4.2	2.6	7.4
Pro	15.5	12.9	12.6	13.4	16.4	13.0
Gly	25.0	30.3	30.1	45.7	35.9	33.4
Ala	16.1	18.9	18.9	12.1	15.5	11.2
Cys	0.3	0.2	0.2	0.1	0.0	-
Val	2.2	2.4	2.3	2.1	3.0	2.5
Met	0.8	0.4	0.4	0.0	0.0	0.5
Ile	1.1	1.0	0.9	0.6	0.4	0.9
Leu	3.0	3.2	3.1	2.3	2.8	2.3
Tyr	0.3	0.1	0.5	0.2	0.0	0.3
Phe	1.4	1.5	1.5	1.2	1.4	1.2
His	0.4	0.5	0.4	1.3	1.4	0.5
OH-lys	0.3	0.5	0.4	0.2	0.0	0.5
Lys	2.8	3.7	3.7	2.0	3.4	2.7
Arg	5.2	6.9	6.7	4.3	6.0	5.0
nmolAS/40µl	664.8	520.7	510.9	163.1	159.0	

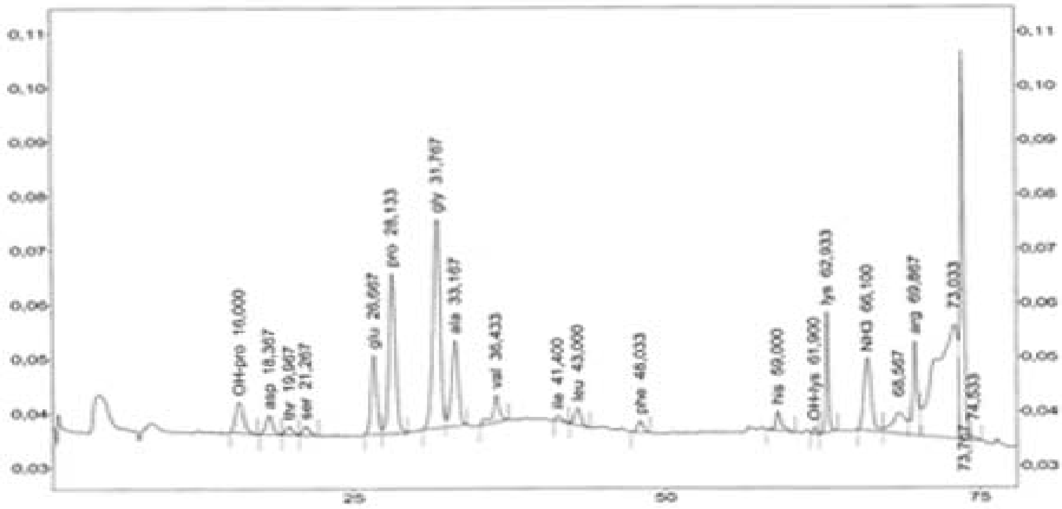

Figure 9.11. Volders burial 25. One of ten randomly sampled for amino acid assay (C:N 3.8). Proportions of individual amino acids are within the acceptable range compared with the standard presented by Ambrose (1993).

9.4 Faunal collagen carbon and nitrogen stable isotope ratios and carbonate carbon and oxygen stable isotope ratios

Faunal $\delta^{13}C$ and $\delta^{15}N$ isotopes from bone collagen

The data for 22 bone samples originating from 22 different individual animals, representing seven species, are presented in Table 8 and Figure 9.12. All of the faunal remains were recovered at the Volders archeological site. This analysis functioned as a basis of comparison for the human bone analysis. Acquiring a pallet of values originating from domestic and wild animals, including mammals and non mammalian species is necessary for an accurate isotopic positioning of humans in their environment.

Table 8. Faunal collagen data. *The C:N ratios exhibited were all within an acceptable range with the exception of the red deer (Cervus elaphus) sample, which indicated a large degree of contamination and was excluded from further analysis.

Species	Coll. (mg)	C/N ratio	δ^{13}C(PDB)	δ^{15}N(PDB)
Bos t. dom.	0.28	3.62	-22.18	5.54
Bos t. dom.	0.38	3.70	-22.22	5.03
Bos t. dom.	0.21	3.61	-22.14	5.70
Bos t. dom.	0.49	3.50	-21.98	5.70
Bos t. dom.	0.35	3.59	-21.91	4.58
Bos t. dom.	0.54	3.36	-21.09	5.96
Bos t. dom.	0.48	-	-21.24	-
Bos t. dom.	0.58	3.58	-20.70	3.56
Capra h.	0.25	3.64	-22.07	4.40
*Cervus e.	0.26	8.88	-21.70	3.74
Equus cab.	0.44	3.64	-23.07	3.79
Gallus dom.	0.38	3.66	-20.74	9.17
Gallus dom.	0.30	3.67	-21.31	9.43
Ovis a.	0.44	3.60	-21.64	5.00
Ovis a.	0.36	3.68	-21.71	5.77
Ovis a.	0.25	3.69	-21.78	4.92
Sus s. dom.	0.39	3.73	-21.80	7.10
Sus s. dom.	0.36	3.63	-21.93	7.77
Sus s. dom.	0.48	3.62	-21.81	7.07
Sus s. dom.	0.38	3.68	-22.40	3.87
Sus s. dom.	0.25	3.68	-22.26	5.87
Sus s. dom.	0.38	3.67	-21.58	8.12

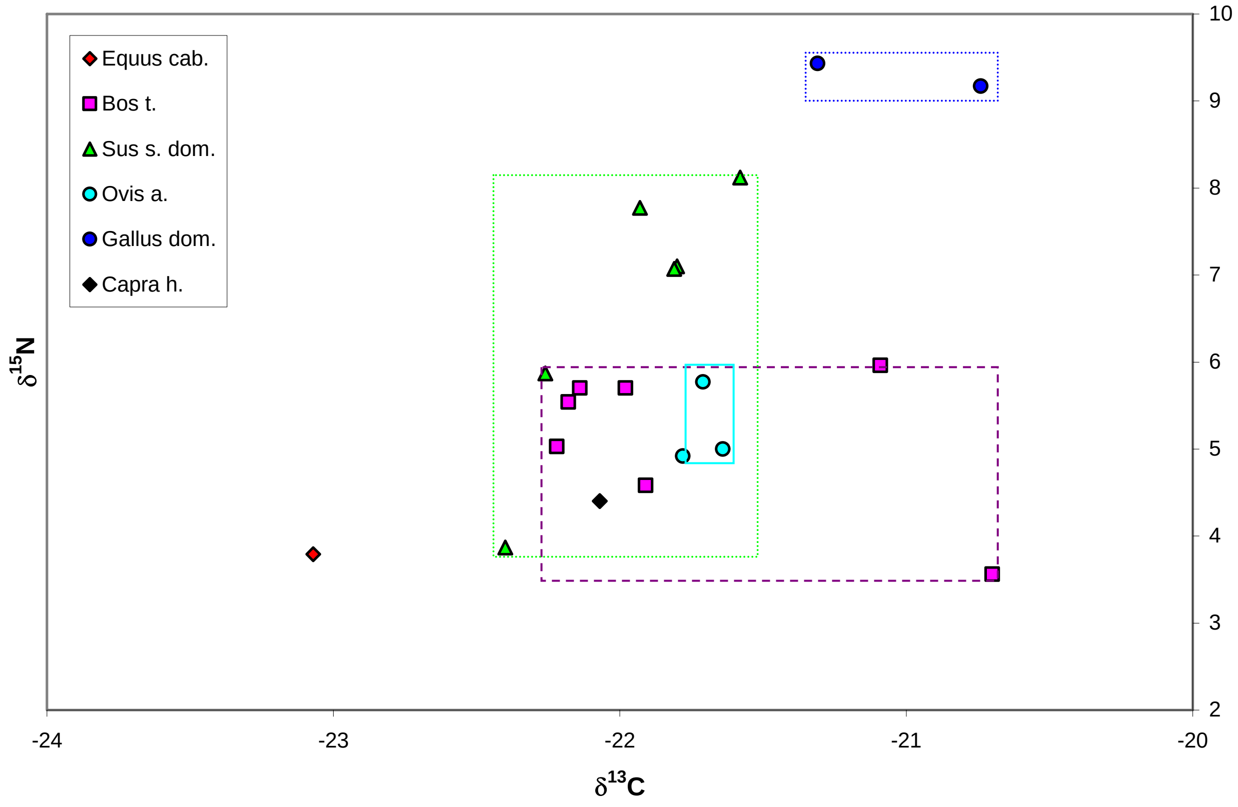

Figure 9.12. Volders faunal $\delta^{13}C$ and $\delta^{15}N$ stable isotope ratios from bone collagen. The ranges for cattle (violet), pigs (green), sheep (light blue) and chicken (dark blue) are indicated with the rectangles.

The faunal collagen ratios exhibit a pattern generally characteristic for the species within a central European ecosystem. Herbivores like the horse, cattle and sheep have typically low $\delta^{15}N$ ratios and subsisted on indigenous C_3 plants. The $\delta^{13}C$ ratios ranging between -20.7‰ and -23.07‰ are indicative of C_3 plants not subjected to the “Baldachin effect”, which would otherwise display highly depleted $\delta^{13}C$ ratios and indicate that these animals were acquiring their food in a thickly forested area. The omnivorous pig, with the exception of one animal that was apparently eating only vegetation, show levels higher than their herbivorous compatriots. The chicken, an animal which generally lives in close proximity to (or even together with) their human keepers, are often fed table scraps, kitchen garbage in addition to grain feed. They also browse and scratch up insects, worms and grubs, further augmenting their diet with protein rich foods and therefore tend to display relatively high nitrogen ratios. Although the ratios for red deer are approximately where they should be for a grazing herbivore, the C:N proportion indicated significant diagenetic alteration, thus making this result unreliable. It can be inferred from these data, that those individuals who were subsisting

upon products derived from cattle and sheep would have lower overall $\delta^{15}N$ ratios than those who were receiving their animal protein from pig meat.

Faunal $\delta^{13}C$ and $\delta^{18}O$ isotopes from bone carbonate

Parallel to the collagen analysis, the stable isotopes of carbon and oxygen from the same 22 samples from the same seven species were analyzed to augment the establishment of an isotope baseline for Volders. The results are presented in Table 9 below.

Table 9. Faunal structural carbonate data.

**Species**	**Sample (g)**	**Extract (mg)**	**δ^{13}C(PDB)**	**1 sigma**	**δ^{18}O(PDB)**	**1 sigma**
Bos t. dom.	101.83	56.04	-12.33	0.11	-9.47	0.11
Bos t. dom.	103.46	57.48	-12.88	0.10	-9.57	0.08
Bos t. dom.	101.44	57.34	-13.16	0.14	-8.83	0.11
Bos t. dom.	103.66	57.47	-12.79	0.11	-8.36	0.15
Bos t. dom.	102.02	43.11	-13.49	0.10	-7.22	0.10
Bos t. dom.	102.3	55.69	-13.26	0.10	-10.37	0.06
Bos t. dom.	102.53	50.42	-12.80	0.09	-8.21	0.10
Bos t. dom.	103.26	47.36	-12.57	0.05	-10.73	0.07
Capra h.	101.64	52.90	-13.43	0.09	-9.77	0.17
Cervus e.	102.69	47.77	-13.47	0.14	-8.87	0.06
Equus cab.	99.77	58.40	-13.84	0.08	-9.31	0.04
Gallus dom.	102.21	57.15	-13.17	0.12	-7.91	0.09
Gallus dom.	101.02	48.52	-13.25	0.08	-13.19	0.13
Ovis a.	99.95	45.04	-13.94	0.10	-9.96	0.14
Ovis a.	102.55	58.99	-14.15	0.05	-8.16	0.08
Ovis a.	100.02	49.38	-15.32	0.12	-12.33	0.14
Sus s. dom.	102.68	45.33	-15.96	0.12	-11.54	0.19
Sus s. dom.	103.19	6.18	-13.62	0.13	-9.34	0.15
Sus s. dom.	103.59	55.42	-14.99	0.09	-9.28	0.11
Sus s. dom.	102.23	47.56	-14.32	0.06	-10.11	0.10
Sus s. dom.	100.91	42.71	-13.96	0.10	-11.98	0.07
Sus s. dom.	104.22	33.25	-14.33	0.12	-12.51	0.12

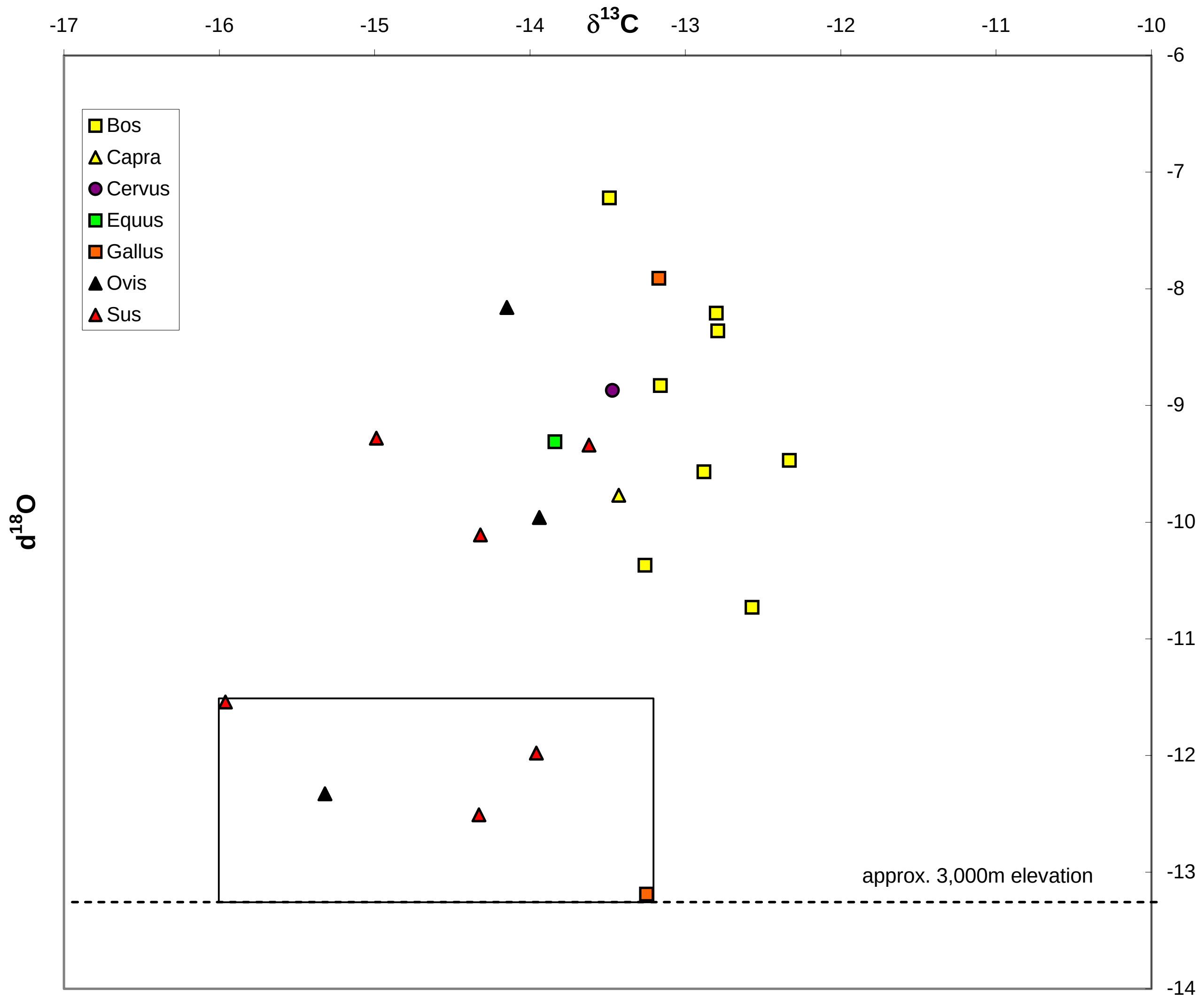

Figure 9.13. Faunal structural carbonate carbon and oxygen isotopes. The checkered line does not imply that the animals were living at 3,000 meters but that the water they were drinking contained the signature of meteoric water deriving from this elevation.

The dispersion of ratios among the different fauna as well as between members of the same species of fauna from Volders show a pattern similar to that observed for the human inhabitants (see Fig. 9.24). It can be assessed from these data that animals with more depleted oxygen levels (Fig. 9.13, enclosed in box) were getting their drinking water from sources located at higher elevations. The data from one of the sheep exhibited a relatively enriched oxygen ratio and did not fulfill expectations that all of these animals were part of the seasonal herd drives to the alm areas. It is possible that this animal was transported in accompaniment of a migrant to the area. According to Amschler (in Pittioni 1973) archaeological evidence from the Inn Valley shows that pigs were often taken to higher elevations in the spring before their slaughter in late autumn, which might explain the depleted levels of oxygen seen in three

of the six samples. These animals were fed on the by-products of the alm dairy and so underwent an intense period of fattening. According to OIPC estimates, one cow and one chicken exhibit $\delta^{18}O$ ratios that might be characteristic of warmer, slightly lower lying areas, perhaps to the south in the northern part of Italy or to the northwest in Germany (see Fig. 7.9).

9.5 Human collagen carbon and nitrogen isotope ratios

The data gathered for the stable isotopic compositions for nitrogen and carbon derive from 91 adult and 12 subadult bone collagen samples and are presented together in Table 10. Collagen sample viability was based primarily upon the C:N molar ratios and few additional amino acid analyses. Samples that exhibited C:N proportions greater than 3.99 were considered too contaminated and excluded from the statistical calculations (i.e. burials 112, 113, and 114). In all, 30 samples did not produce any result whatsoever due to poor collagen survivability.

Table 10. Carbon and nitrogen stable isotope ratios from bone collagen for all individuals represented in the Volders skeletal series.

Burial	Bone sampled	Sex	Sample weight (g)	Sample weight for lypholization (mg)	Extract (mg)	Extract total %	%C	%N	C/N (molar)	$\delta^{13}C$	$\delta^{15}N$
1	tibia	tm	4.93	501.78	2.85	0.57	40.36	13.16	3.58	-19.72	9.74
2	humerus	tf	1.2	506.81	12.43	2.45	50.14	16.38	3.57	-20.20	10.51
3	femur	m	2.36	500.6	7.63	1.52	46.54	15.26	3.56	-20.18	10.25
4	tibia	tm	1.08	499.55	0.97	0.19	41.40	14.40	3.31	-20.10	9.30
6	femur	tm	2.81	501.08	5.31	1.06	36.52	12.12	3.51	-19.65	11.10
7	humerus	m	1.2	500.13	6.54	1.31	36.02	13.35	3.15	-19.74	11.18
8	humerus	tm	1.25	502.09	-	-	-	-	-	-	-
9	scapula	f	2.12	500.55	6.22	1.24	41.71	13.90	3.50	-20.04	10.51
9b	radius	nd	2.05	501.21	16.43	3.28	45.59	14.94	3.56	-20.18	9.36
10	femur	m	0.98	497.78	4.77	0.96	39.14	12.47	3.66	-19.59	10.61
11	femur	f	0.98	500.43	0.98	0.2	27.83	11.20	2.90	-19.08	11.91
12	femur	nd	1.15	501.6	1.02	0.2	27.29	9.73	3.27	-19.19	12.85
13	femur	tm	1.12	502.4	6.57	1.31	41.58	13.05	3.72	-19.89	10.43
14	femur	f	0.78	504.14	2.23	0.44	41.89	13.19	3.70	-20.29	10.50
15	femur	f	1.01	500.36	9.61	1.92	48.26	14.62	3.85	-20.20	9.13
16	femur	m	1.06	498.39	11.29	2.27	45.31	14.26	3.71	-20.10	8.30
17	clavicle	m	1.57	500.23	1.43	0.29	48.52	15.91	3.56	-20.17	10.43
18	femur	m	3.39	501.34	13.29	2.65	34.27	10.37	3.86	-20.18	9.75
19	femur	m	0.92	500.14	3.3	0.66	38.16	11.80	3.77	-19.97	10.09
20	femur	nd	3.76	499.18	24.86	4.98	42.25	13.52	3.65	-19.52	11.34
21	cranium	nd	4.48	501.07	3.4	0.68	42.43	14.42	3.43	-19.91	11.05
22	humerus	m	9.84	499.6	8.19	1.64	-	-	-	-	-
23	femur	m	1.56	458.14	5.13	1.12	14.76	46.60	3.68	-20.11	9.97
24	femur	m	1.4	501.1	5.12	1.02	31.11	9.91	3.66	-19.92	10.10

25	femur	m	1.66	500.9	21.88	4.37	42.94	13.10	3.82	-19.93	10.34
26	femur	f	1.07	499.21	17.68	3.54	37.47	11.97	3.65	-20.14	9.05
27	femur	m	4.03	501.2	2.81	0.56	-	-	3.38	-20.45	9.94
28	tibia	tm	0.81	502.57	4.55	0.91	27.98	9.22	3.54	-20.52	9.60
29	femur	f	0.64	499.3	-	-	-	-	-	-	-
30	femur	tm	1.08	501.5	23.51	4.69	41.81	13.05	3.74	-20.59	9.10
31	femur	m	2.5	501.3	18.1	3.6	-	-	-	-	-
32	Rib	m	3.16	502.54	3.25	0.65	-	-	-	-	-
33	Fibula	f	5.88	499.24	3.6	0.72	-	-	-	-	-
34	femur	f	0.85	502.05	1.33	0.26	-	-	-	-	-
35	femur	m	2.21	498.06	2.18	0.44	37.98	12.84	3.45	-19.67	10.19
36	humerus	f	8.42	498.5	0.98	0.19	27.10	8.84	3.58	-19.81	10.68
37	cranium	m	5.45	499.63	1.2	0.24	-	-	-	-	-
38	femur	m	1.14	503.7	4.6	0.9	30.85	10.76	3.34	-19.77	9.86
40	femur	f	1.08	506.34	39.98	7.9	-	-	-	-	-
41	femur	f	0.86	500.49	4.01	0.8	39.05	13.56	3.36	-19.55	10.06
42	femur	m	3.12	500.14	2.54	0.5	-	-	2.60	-20.39	9.85
43	femur	f	0.85	501.12	10.61	2.12	6.00	14.23	3.86	-20.19	10.04
44	femur	nd	1.04	500.6	12.5	2.5	45.99	15.04	3.56	-19.72	10.12
45	cranium	m	5	502.24	3.85	0.77	35.36	11.41	3.61	-19.46	9.97
46	cranium	nd	1.61	498.29	22.33	4.48	41.70	13.13	3.71	-18.74	11.74
47	tibia	nd	0.85	498.31	11.88	2.38	-	-	-	-	-
48	femur	m	1.21	497.35	3.7	0.74	50.42	16.02	3.67	-19.42	9.92
49	tibia	m	1.06	501.23	7.46	1.49	46.00	15.27	3.51	-19.40	9.47
50	femur	m	3.65	500.2	2.64	0.52	-	-	3.28	-20.06	11.50
51	cranium	m	2.61	499.98	16.7	3.34	-	-	-	-	-
52	femur	m	0.92	506.23	5.34	1.05	49.25	15.84	3.63	-19.95	9.78
53b	femur	m	0.62	500.33	3.66	0.73	-	-	-	-	-
54	cranium	m	5.65	500.96	8.27	1.65	40.96	13.32	3.59	-20.06	10.50
56	femur	f	4.44	501	2.7	0.53	-	-	3.29	-21.24	10.29
57	femur	m	1.13	514.63	5.31	1.03	42.37	14.56	3.39	-20.15	9.56
59	femur	m	1.46	500.31	4.23	0.85	44.84	15.37	3.40	-20.35	9.66
60	femur	f	2.37	500.48	12.28	2.45	54.89	17.98	3.56	-19.84	9.61
61	femur	m	2.95	500.74	21.71	4.34	46.88	15.06	3.63	-19.85	10.25
63	calcaneus	nd	4.72	502.22	12.21	2.43	43.09	13.83	3.63	-20.23	10.07
64	humerus	nd	4.37	495.59	3.14	0.63	47.56	16.58	3.35	-20.25	9.74
65	femur	m	3.1	501.3	2.54	0.5	-	-	3.39	-21.47	7.78
66	femur	m	2.67	498.21	10.95	2.19	40.64	12.93	3.67	-19.74	9.12
67	femur	m	1.46	501.91	4.54	0.91	35.08	11.33	3.61	-19.99	9.33
68	humerus	m	5.46	498.67	5.52	1.11	40.73	13.06	3.64	-20.12	9.61
69	femur	f	1.97	503.09	1.32	0.26	34.71	11.64	3.48	-19.88	9.46
70	femur	m	1.4	500.25	11.83	2.36	41.65	14.13	3.44	-19.49	10.70
71	femur	f	1.27	501.34	0.66	0.13	29.55	12.70	2.72	-20.89	10.36
73	cranium	f	5.82	533.69	5.99	1.12	42.10	13.27	3.70	-20.42	9.87
74	femur	f	1.29	499.28	11.59	2.32	42.64	13.60	3.66	-19.26	9.85
76	femur	tm	1.45	500.36	6.23	1.25	43.49	15.73	3.22	-20.49	9.49
77	femur	m	0.51	484.06	1.49	0.31	35.43	11.16	3.70	-20.25	9.57
79	femur	f	1.28	501.4	1.24	0.25	26.29	10.86	2.82	-19.72	11.69
80	femur	f	3.05	502.32	11.4	2.27	-	-	-	-	-
81	femur	m	1.17	500.94	1.63	0.33	38.19	11.19	3.98	-19.84	8.06
82	femur	m	1.07	500.68	12.46	2.49	37.99	12.20	3.63	-19.78	10.40
83	rib	f	2.33	499.46	15.69	3.14	34.52	12.11	3.32	-19.95	9.94
84	tibia	m	1.02	455.81	17.63	3.87	43.23	13.61	3.71	-19.75	9.87

85	femur	m	1.33	503.3	0.55	0.11	23.30	6.81	3.99	-19.99	8.88
86	femur	m	1.03	498.94	-	-	-	-	-	-	-
87	cranium	m	7.24	497.92	2.69	0.54	-	-	-	-	-
88	femur	nd	2.23	490.7	14.2	2.89	-	-	-	-	-
89	femur	nd	3.56	497.1	9.5	1.91	-	-	-	-	-
90	femur	m	0.92	500.66	-	-	-	-	-	-	-
91	femur	m	0.78	504.61	5.18	1.03	35.41	11.58	3.57	-20.20	10.43
92	cranium	m	1.78	502	2.8	0.55	-	-	-	-	-
93	femur	f	1.06	502.58	2.02	0.4	31.80	9.81	3.78	-20.00	9.00
94	femur	f	2.03	483.1	11.9	2.46	-	-	-	-	-
95	femur	tf	0.34	281.89	4.11	1.46	-	-	-	-	-
96	femur	f	0.54	443.08	9.2	2.07	-	-	-	-	-
97	femur	m	1.75	495.8	11.3	2.27	-	-	-	-	-
98	cranium	m	7.07	495.38	2.38	0.48	-	-	-	-	-
99	femur	f	1.67	502.59	2.08	0.41	29.04	10.10	3.35	-20.17	9.06
100	femur	m	0.84	502.3	1.41	0.28	30.65	9.73	3.67	-20.51	11.70
101	femur	f	1.61	501.78	2.9	0.58	30.61	11.79	3.03	-19.99	11.23
102	femur	nd	1.12	479.9	11.7	2.43	-	-	-	-	-
103	femur	m	1.25	506.87	6.97	1.38	40.75	12.90	3.68	-20.06	9.41
104	femur	f	0.64	490.09	7.3	1.49	-	-	-	-	-
105	femur	f	1.09	497.84	-	-	-	-	-	-	-
106	femur	m	1.43	500.43	3.9	0.78	38.16	12.65	3.52	-20.47	8.92
107	femur	m	1.4	501.35	1.37	0.27	40.37	14.19	3.32	-19.91	8.98
108	femur	m	1.57	500.12	4.59	0.92	42.15	13.97	3.52	-20.28	9.86
109	femur	tf	0.79	498.7	-	-	-	-	-	-	-
110	femur	m	0.9	501.03	4.71	0.94	48.32	14.54	3.88	-19.50	9.64
111	humerus	m	1.66	500.2	8.4	1.68	-	-	-	-	
112	femur	m	1.12	500.36	1.01	0.2	47.49	10.84	5.11	-23.60	9.57
113	femur	m	0.92	502.32	0.55	0.11	27.55	7.15	4.49	-19.90	9.34
114	femur	f	0.69	499.44	0.75	0.15	25.60	6.68	4.47	-19.85	10.84
115	humerus	nd	2.23	469.7	10.1	2.13	-	-	-	-	-
116	femur	m	1.28	500.93	1.73	0.35	36.94	12.09	3.56	-20.21	9.34
117	femur	nd	1.96	498.4	15.3	3.08	-	-	-	-	-
118	humerus	m	6.8	485.87	8.9	1.83	-	-	-	-	-
119	tibia	nd	2.69	497.86	5.16	1.04	30.85	10.91	3.30	-19.54	12.24
120	tibia	tm	1.27	501.93	6.1	1.22	39.83	12.66	3.67	-20.14	9.82
121	humerus	m	1.88	502.81	7.5	1.49	31.83	10.65	3.49	-20.41	10.15
123	femur	f	1.21	496.71	2.06	0.41	42.34	13.39	3.69	-19.77	9.98
124	femur	nd	2.78	484.3	23.9	4.93	-	-	-	-	-
125	femur	f	2.88	487.3	4.9	1.0	-	-	-	-	-
126	femur	m	4.54	489.7	19.7	4.02	-	-	-	-	-
127	femur	nd	1.21	484.1	12.7	2.62	-	-	-	-	-
129	humerus	f	1.44	500.25	15.15	3.03	49.99	16.71	3.49	-20.18	10.55
132	femur	m	1.47	500.85	5.75	1.15	49.21	14.67	3.91	-19.48	10.11
133	humerus	nd	1.56	498.92	7.12	1.43	32.57	10.74	3.54	-19.82	10.31
134	femur	nd	2.94	500.17	15.14	3.03	44.47	13.36	3.88	-19.66	9.77
135	humerus	m	0.34	229.33	0.39	0.17	-	-	-	-	-
136	tibia	m	0.8	502.91	1.29	0.26	34.07	11.74	3.39	-19.52	10.56
137	tibia	m	1.57	499.9	1.91	0.38	35.20	11.45	3.59	-20.02	8.71
138	tibia	tm	1.17	499.66	1.42	0.28	34.92	12.81	3.18	-20.36	11.37
139	humerus	f	7.27	503.43	10.09	2	-	-	-	-	-
140	femur	m	1.03	501.65	1.59	0.32	26.07	8.90	3.42	-20.18	10.73
141	cranium	nd	7.06	499.27	1.38	0.28	26.73	9.51	3.28	-21.06	5.71

142	metatarsal	tm	4.02	198.37	10.31	2.07	49.95	14.94	3.90	-19.95	9.06
143	tibia	m	1.24	506.44	5.59	1.1	39.47	12.38	3.72	-20.30	9.61
144	tibia	m	0.8	499.4	17.66	3.54	34.49	11.02	3.65	-20.09	10.27
145	humerus	m	0.57	426.98	10.78	2.25	42.15	14.96	3.29	-20.23	10.49
146	femur	f	1.65	501.46	2.89	0.58	39.23	12.45	3.67	-19.49	9.60
147	femur	f	1.45	500.1	24.4	4.87	-	-	-	-	-
148	femur	m	0.73	500.44	19.52	3.9	41.47	13.12	3.69	-18.75	9.90
149	femur	m	1.25	500.35	9.39	1.88	38.86	12.54	3.61	-19.52	10.37
150	cranium	f	1.11	499.52	17.06	3.42	43.48	14.19	3.57	-19.87	10.44
151	humerus	m	1.97	499.67	9.5	1.9	45.74	14.73	3.62	-19.54	9.72
152	femur	nd	2.01	500.74	16.54	3.3	48.25	15.55	3.62	-20.00	11.13
153	humerus	m	1.03	502.09	13.94	2.78	37.41	14.19	3.08	-20.09	10.84
19152	vertebra	m	0.89	503.4	6.18	1.23	-	-	-	-	-

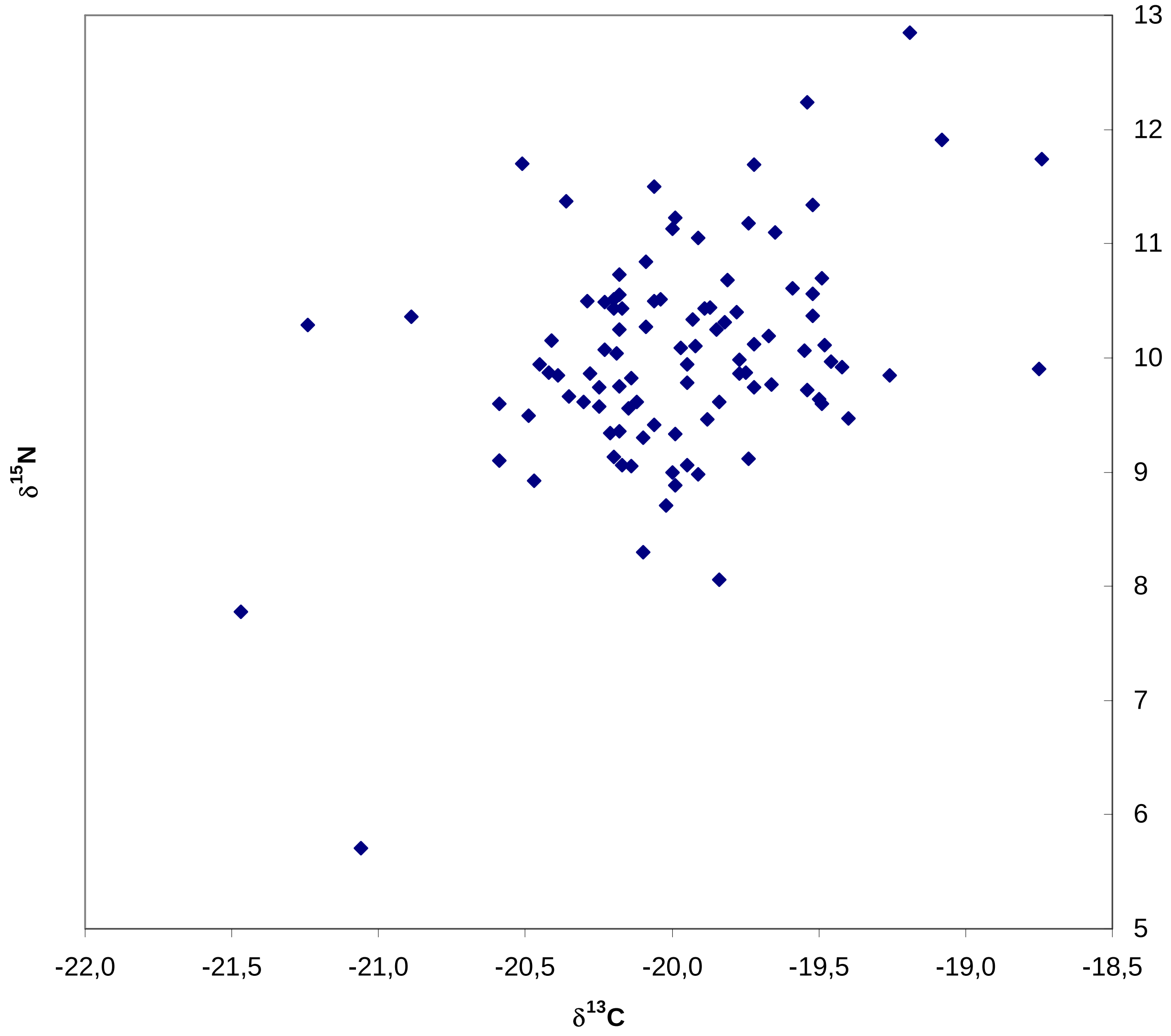

Figure 9.14. Overview of the bone collagen carbon and nitrogen stable isotope ratios for the entire Volders skeletal series.

A wide range of isotope variability is observable for the Volders skeletal series particularly with respect to nitrogen levels and is illustrated in Figure 9.14. Differences in the range in carbon ratios are distinctly less variable, which is even more apparent in an individual comparison of the adult and subadult ratios.

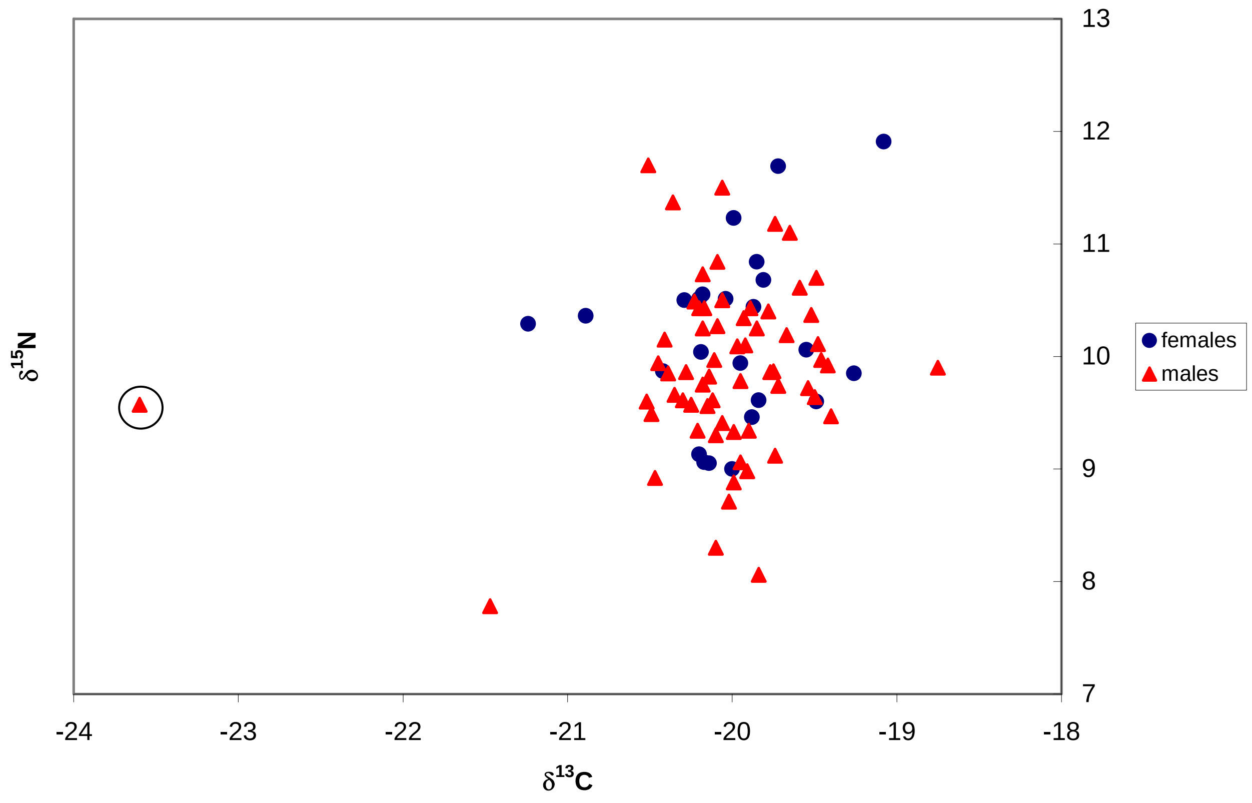

Figure 9.15. Comparison between bone collagen carbon and nitrogen stable isotope ratios in adult males and females.

A comparison of adult nitrogen and carbon stable isotope ratios in Figure 9.15 illustrates the large range of variability exhibited within the population. The $\delta^{15}N$ values for adult females ranged between 9.00-11.91‰ and those for males fell between a low of 7.78‰ and a high of 11.70‰. The variability exhibited within each group, $\Delta\ \delta^{15}N$ = 2.91‰ for females and $\Delta\ \delta^{15}N$ = 3.92‰ for males both constitute a full trophic level. Overall, there was no significant difference between males and females (see Table 11), yet, it is interesting to note, that 15 of the lowest 20 nitrogen ratios belonged to males and 13 of the highest 20 also belonged to males. Although it should be mentioned that males did outnumber females 2 to 1. The male

individual highlighted with a circle in Fig. 9.15 shows a $\delta^{13}C$ value approaching that of the inclusion of freshwater fish in the diet, however, the C:N ratio 5.1 indicated significant contamination that renders this specific isotopic composition unreliable.

The results of a statistical analysis of the isotope ratios acquired from bone collagen in adults are elucidated below. The means, standard deviations and two sample t-test results are listed in Table 11.

Nitrogen ratios $\delta^{15}N$

A comparison of adult male individuals with adult females indicated no significant difference in isotopic compositions of nitrogen and carbon. There were 5 females with mean nitrogen score 11.27‰, 2 standard deviations above the mean. Two of these females were 3 standard deviations above the mean for nitrogen. Fifteen males had a mean nitrogen score of 10.89‰, 2 standard deviations above the mean for nitrogen. Two of these males were 3 standard deviations above the mean for nitrogen.

In a comparison of nitrogen ratios between three juveniles with six adults for the mean for juveniles was 10.35‰ and the standard deviation was .249, for adults the mean was 10.03‰ and the standard deviation was .924. The t= .702, which is not significant at the .05 percent level.

Combined adult $\delta^{15}N$ ratios exhibit a very high degree of variability, which is remarkable for a presumably agrarian based population. A number of individuals are separated by a full trophic level.

Carbon ratios $\delta^{13}C$

The mean $\delta^{13}C$ value for males was -20.03‰ with a standard deviation of .597 and the mean for females was -19.9‰ with a standard deviation of .370. This level of carbon isotope is indicative for humans consuming C_3 herbivores and C_3 plants, which reflect a carbon signature typical for the C_3 plant biome indigenous to the Inn Valley and were more or less awaited for this skeletal series.

Table 11. Two sample t test for carbon and nitrogen stable isotopes in bone collagen including means, standard deviation and two sample t-test. Adult males vs. adult females.

	Males		Females		t at .05
	Mean	SD	Mean	SD	
Carbon	-19.99‰	.387	-20.02‰	.438	1.03*
Nitrogen	9.89‰	.757	10.17‰	.767	1.84*

* Not significant at the .05 level

Table 12. Subadult bone collagen carbon and nitrogen stable isotope data.

Burial	Bone sampled	Sex	Age (yrs)	Sample weight for lypholization (mg)	Extract (mg)	Extract total %	%C	%N	C/N (molar)	$\delta^{13}C$ (‰)	$\delta^{15}N$ (‰)
12	femur	nd	1.5-2	501.6	1.02	0.2	27.29	9.73	3.27	-19.19	12.85
20	femur	nd	5-7	499.18	24.86	4.98	42.25	13.52	3.65	-19.52	11.34
21	cranium	nd	4-5	501.07	3.4	0.68	42.43	14.42	3.43	-19.91	11.05
30	femur	tm	neonate	501.5	23.51	4.69	41.81	13.06	3.74	-20.59	9.10
46	cranium	nd	<1	498.29	22.33	4.48	41.70	13.13	3.71	-18.74	11.74
119	tibia	nd	2-3	497.86	5.16	1.04	30.85	10.91	3.30	-19.54	12.24
123	femur	f	18-19	496.71	2.06	0.41	42.34	13.39	3.69	-19.77	9.98
133	humerus	nd	neonate	498.92	7.12	1.43	32.57	10.74	3.54	-19.82	10.31
134	femur	nd	3-5	500.17	15.14	3.03	44.47	13.36	3.88	-19.66	9.77
136	tibia	m	17-19	502.91	1.29	0.26	34.07	11.74	3.39	-19.52	10.56
141	cranium	nd	5-7	499.27	1.38	0.28	26.73	9.52	3.28	-21.06	5.71
152	femur	nd	<1	500.74	16.54	3.3	48.25	15.55	3.62	-20.00	11.13

The range of $\delta^{15}N$ values from the subadult group displayed an even more striking variability than those of the adults. The spread of ratios for subadults at Volders is illustrated in Figure 9.15. The $\Delta\ \delta^{15}N$ = 7.14‰ represents a two trophic level difference between the lowest 5.71‰ and highest 12.85‰ nitrogen values. These differences are certainly in part the result of nitrogen enrichment through nursing, however, if the highest (11.34‰) and lowest (5.71‰) ratios recorded for presumably non-nursing subadults are used (burials 20 and 141 respectively), the $\Delta\ \delta^{15}N$ of 5.63‰ still indicates a significant trophic level difference.

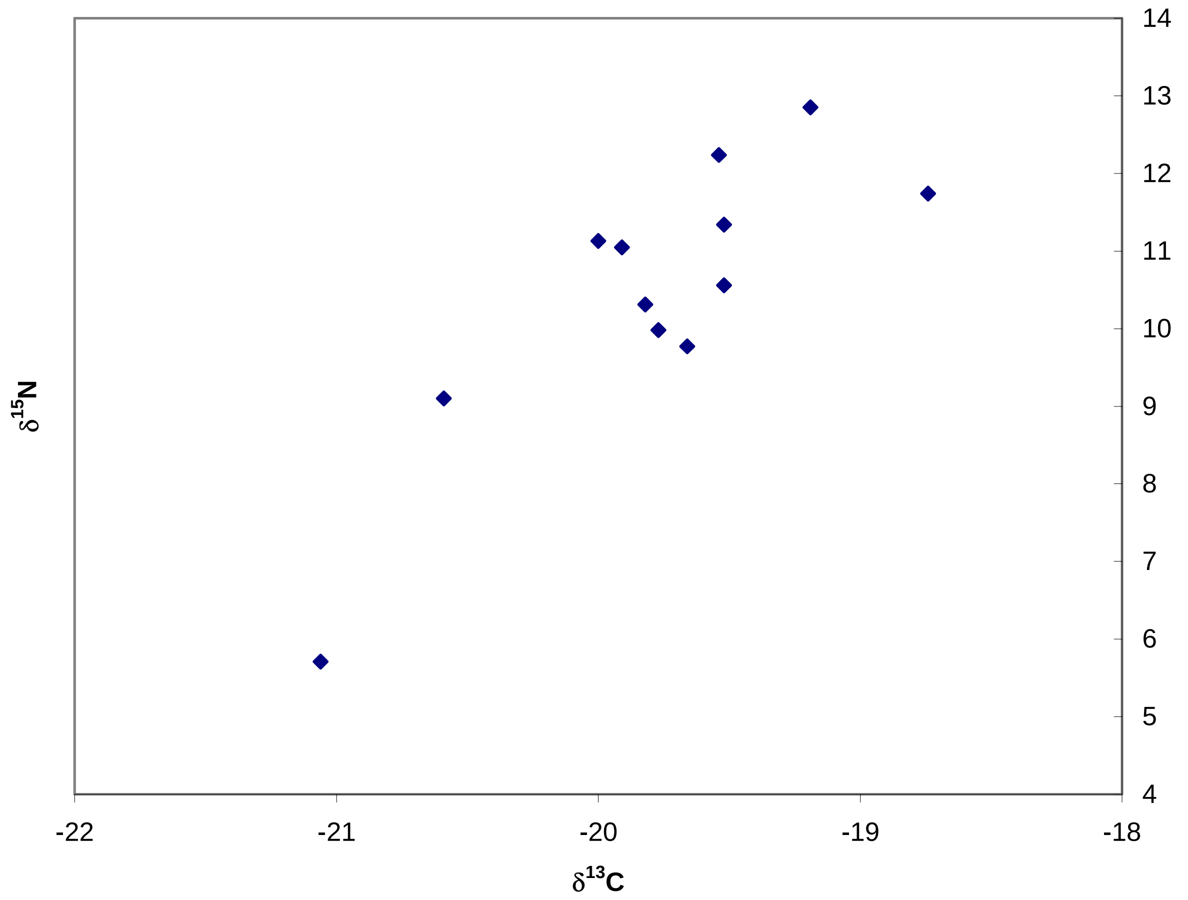

Figure 9.16. Subadult collagen carbon and nitrogen ratios.

With the exception of burial 141 (see Table 12), which exhibits an extremely low nitrogen level perhaps indicative of low protein-high C_3 plant intake, the data deriving from subadult individuals indicate that children were either weaned late, continued to nurse to augment their new diet or received high protein diets in early childhood. Ratios from burials 12 and 119 both containing children approximately 2 years of age would indicate that nursing was practiced at least through the second year. The curve produced by the subadult values and shown in Figure 9.17 also supports this assumption. The preservation of the neonate in burial 133 was very poor, making a morphological age determination complicated. The $\delta^{15}N$ ratio suggests that the baby had begun nursing and was not a newborn.

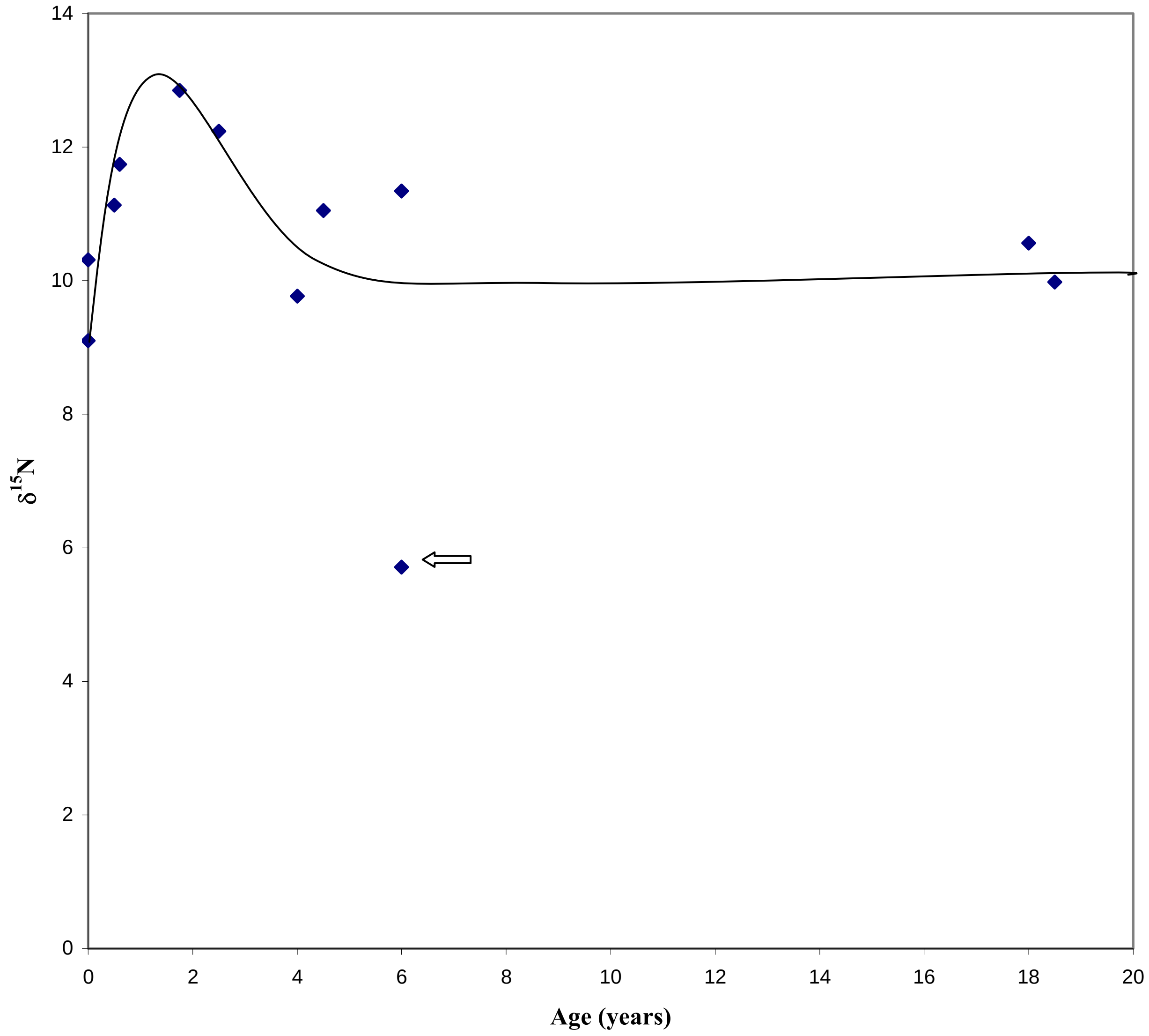

Figure 9.17. Depiction of the enrichment and depletion trend in $\delta^{15}N$ amongst subadult individuals. The rapid increase in nitrogen levels within the first year after birth is clearly shown, peaking at approximately 2 years of age and then dropping off to the levels characteristic for the adult population. The child in burial 141 (arrow), which as already mentioned exhibited an extraordinarily low nitrogen level (5.71‰), obviously represents a case of low protein consumption or perhaps was suffering from an unspecified physiological disorder resulting in this exceptionally depleted nitrogen ratio.

9.6 Structural carbonate carbon and oxygen stable isotope ratios

Structural carbonate was extractable from bone samples originating from 105 adult and 24 subadult individuals from the Volders skeletal series and tested for the isotopic compositions of carbon and oxygen. The data is presented in Tables 13 and 14 and illustrated on scatter graphs in Figures 9.18 and 9.19 (adults), and 9.20 (subadults).

Table 13. Adult carbon and oxygen stable isotope data acquired from bone structural carbonate for the Volders skeletal series. Extract data for samples 1-16 and 19 were lost in the shuffle and not available. C:N ratios were included here simply to serve as an indicator for potential contamination. Burial 49 exhibited an aberrant carbon value (-0.84‰) and was eliminated from further analysis.

Burial	Bone sampled	Sex	Sample weight for lypholization (mg)	Extract (g)	Extract total %	C/N (molar)	$\delta^{13}C$ (PDB)	$\delta^{18}O$ (PDB)
1	tibia	tm	102.7	-	-	3.58	-12.00	-7.60
2	humerus	tf	102.2	-	-	3.57	-14.30	-7.57
3	femur	m	102.1	-	-	3.56	-13.21	-7.90
4	tibia	tm	101.5	-	-	3.31	-14.83	-7.39
6	femur	tm	100.6	-	-	3.52	-13.00	-7.65
7	humerus	m	101.5	-	-	3.15	-12.79	-8.18
8	humerus	tm	102.9	-	-	-	-12.28	-8.05
9	scapula	f	101.6	-	-	3.50	-12.62	-7.75
9b	radius	nd	101.1	-	-	3.56	-13.95	-7.83
10	femur	m	100.7	-	-	3.66	-12.51	-7.93
11	femur	f	101.0	-	-	2.90	-12.08	-8.21
13	femur	tm	102.0	-	-	3.72	-12.82	-8.35
14	femur	f	100.7	-	-	3.71	-14.01	-8.84
15	femur	f	100.5	-	-	3.85	-13.24	-7.33
16	femur	m	100.4	-	-	3.71	-13.84	-8.17
17	clavicle	m	101.8	71.7	70.4	3.56	-12.12	-8.77
18	femur	m	101.5	28.0	27.6	3.86	-14.02	-9.84
19	femur	m	100.3	-	-	3.77	-12.80	-8.54
22	humerus	m	100.8	61.5	61.0	-	-13.66	-9.49
23	femur	m	100.1	46.1	46.1	3.68	-12.82	-9.62
24	femur	m	100.1	48.7	48.7	3.66	-12.15	-10.32
25	femur	m	101.3	41.4	40.8	3.82	-14.55	-14.34
26	femur	f	100.7	50.4	50.0	3.65	-13.68	-15.26
27	femur	m	100.6	70.0	69.6	3.38	-13.28	-9.34
28	tibia	tm	102.4	49.0	47.9	3.54	-12.41	-9.02
29	femur	f	61.7	22.8	37.0	-	-12.80	-8.25
31	femur	m	100.5	52.2	51.9	-	-14.06	-11.94
33	fibula	f	101.1	50.6	50.0	-	-12.84	-9.63
34	femur	f	100.6	53.9	53.6	-	-11.39	-9.09
35	femur	m	99.7	61.0	61.2	3.45	-12.63	-10.53
36	humerus	f	101.7	62.0	61.0	3.58	-12.63	-9.59
37	cranium	m	101.7	74.9	73.6	-	-13.47	-8.31
38	femur	m	100.3	57.4	57.2	3.34	-13.44	-6.42
41	femur	f	97.9	44.9	45.9	3.36	-12.68	-8.21

42	femur	m	101.7	64.2	63.1	2.60	-12.72	-10.60
43	femur	f	100.8	54.9	54.5	3.86	-14.20	-6.71
44	femur	nd	101.5	48.0	47.3	3.57	-13.78	-8.58
45	cranium	m	100.9	71.7	71.1	3.61	-12.63	-8.45
48	femur	m	102.2	45.4	44.4	3.67	-12.70	-13.08
49	tibia	m	100.7	45.6	45.3	3.51	-0.84	-4.99
51	femur	m	100.9	34.2	33.9	-	-13.62	-10.14
56	femur	f	100.8	63.8	63.3	3.29	-12.82	-8.21
57	femur	m	100.5	60.2	60.0	3.39	-12.46	-9.05
59	femur	m	101.3	65.7	64.9	3.40	-13.61	-13.59
60	femur	f	102.6	47.8	46.6	3.56	-13.80	-9.26
61	femur	m	100.2	51.6	51.5	3.63	-13.24	-12.19
64	humerus	nd	100.3	58.7	58.5	3.35	-13.56	-8.50
67	femur	m	99.4	58.4	58.8	3.61	-12.99	-10.11
70	femur	m	103.9	64.9	62.5	3.44	-13.19	-9.82
74	femur	f	100.8	66.0	65.5	3.66	-12.69	-13.75
77	femur	m	101.2	57.0	56.3	3.70	-13.99	-8.65
80	femur	f	100.3	46.0	45.9	-	-13.93	-6.19
81	femur	m	99.6	79.2	79.5	3.98	-12.78	-9.60
82	femur	m	101.2	22.8	22.5	3.63	-14.05	-11.32
83	tibia	f	100.0	41.1	41.1	3.32	-11.30	-9.22
84	tibia	m	101.4	46.1	45.5	3.71	-14.30	-9.88
86	femur	m	100.8	69.4	68.8	-	-12.91	-8.49
87	cranium	m	101.0	68.5	67.8	-	-13.36	-9.96
90	femur	m	102.0	48.9	47.9	-	-13.63	-11.81
91	femur	m	76.7	57.2	74.6	3.57	-13.74	-10.35
92	cranium	m	100.7	64.9	64.4	-	-13.80	-14.20
93	femur	f	99.7	63.6	63.8	3.78	-13.62	-8.89
95	femur	tf	100.3	56.4	56.2	-	-14.06	-9.49
96	femur	f	100.5	59.3	59.0	-	-13.60	-13.53
97	femur	m	100.6	36.5	36.3	-	-14.00	-8.32
99	femur	f	99.8	46.1	46.2	3.35	-12.74	-9.68
100	femur	m	101.2	48.4	47.8	3.67	-13.88	-9.20
101	femur	f	100.4	61.1	60.9	3.03	-13.75	-10.00
103	femur	m	101.7	65.4	64.3	3.68	-12.87	-9.24
105	femur	f	99.3	70.8	71.3	-	-12.41	-9.02
106	femur	m	101.2	60.2	59.5	3.52	-12.41	-8.33
107	femur	m	100.4	68.9	68.6	3.32	-12.88	-9.48
108	femur	m	100.3	66.4	66.2	3.52	-13.32	-11.19
109	femur	tf	101.2	43.8	43.3	-	-12.41	-8.33
110	femur	m	101.0	70.6	70.0	3.88	-13.58	-9.74
111	humerus	m	100.2	60.1	60.0	-	-14.06	-10.10

112	femur	m	101.8	27.8	27.3	5.11	-13.11	-8.73
113	femur	m	100.1	69.8	69.7	4.49	-13.19	-10.19
114	femur	f	100.4	36.5	36.4	4.47	-12.93	-8.54
116	femur	m	99.7	58.3	58.5	3.56	-13.99	-11.20
118	humerus	m	100.5	43.6	43.4	-	-12.90	-8.87
120	tibia	tm	99.0	30.2	30.5	3.67	-12.94	-16.03
121	humerus	m	100.7	49.5	49.2	3.49	-13.54	-8.33
125	femur	f	100.9	48.4	48.0	-	-13.46	-10.21
126	femur	m	100.0	27.9	27.9	-	-13.64	-9.85
127	humerus	nd	100.0	40.7	40.7	-	-13.54	-14.77
129	humerus	f	101.2	34.9	34.5	3.49	-14.49	-9.91
132	femur	m	101.3	66.5	65.6	3.91	-13.29	-9.14
135	humerus	m	100.8	57.0	56.5	-	-13.30	-8.96
137	tibia	m	100.0	60.3	60.3	3.59	-12.75	-10.28
138	tibia	tm	100.1	69.3	69.2	3.18	-10.93	-10.19
139	humerus	f	100.6	58.8	58.5	-	-14.52	-10.70
140	femur	m	100.4	67.4	67.1	3.42	-12.26	-9.73
142	metatarsal	tm	101.3	44.5	43.9	3.90	-14.52	-10.70
143	tibia	m	101.2	61.8	61.1	3.72	-12.84	-9.37
144	tibia	m	102.0	41.6	40.8	3.65	-13.92	-12.43
145	humerus	m	100.3	38.0	37.9	3.29	-13.14	-8.52
146	femur	f	101.9	66.6	65.4	3.67	-12.84	-9.27
147	clavicle	f	100.4	62.0	61.8	-	-13.41	-8.58
148	femur	m	102.9	55.7	54.1	3.69	-13.21	-12.07
149	femur	m	100.8	51.5	51.1	3.61	-14.30	-10.71
150	cranium	f	100.5	51.9	51.6	3.57	-13.32	-12.91
151	humerus	m	100.5	55.2	54.9	3.62	-13.42	-11.69
153	humerus	m	101.2	53.8	53.2	3.08	-12.69	-9.17
19152	vertebra	m	101.7	59.3	58.3	-	-14.17	-10.01

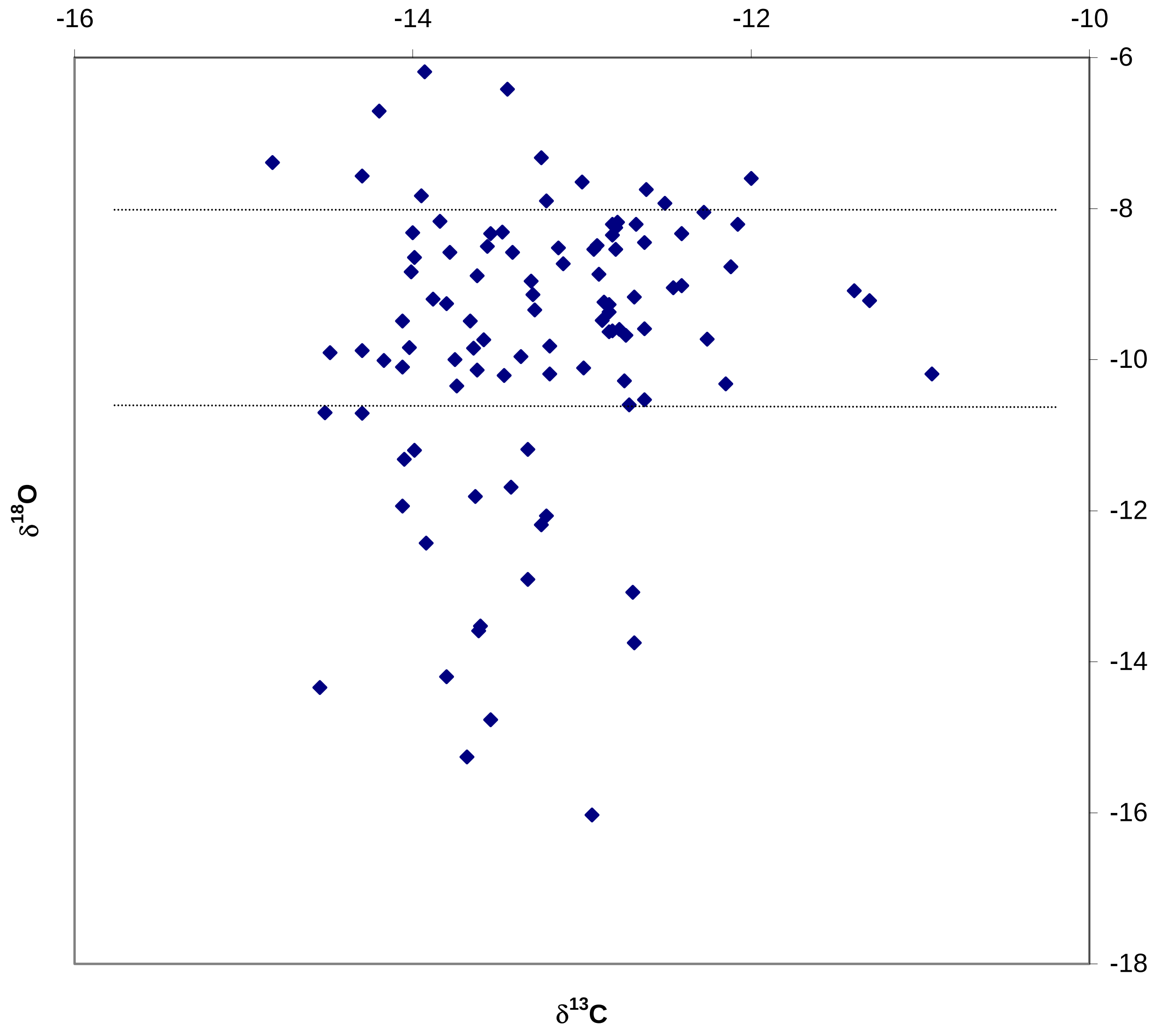

Figure 9.18. Adult structural carbonate carbon and oxygen isotope ratios. A very large variability is illustrated for $\delta^{18}O$ and although an obvious cluster exists between approximately -8‰ and -10.5‰ (between lines), there are numerous individuals whose ratios differ significantly from this group. The range displayed by the majority of $\delta^{13}C$ ratios falls within approximately 2‰ of one another.

Unlike various species of animals which derive significant amounts of water from their food through grazing and browsing, which can be enriched in $\delta^{18}O$ relative to the local meteoric water, only a small portion of the water isotopes incorporated into the bone mineral of humans is derived through food intake (White et al. 2004). Since humans are obligate drinkers and obtain their water primarily by drinking it, the $\delta^{18}O$ detected in their tissues originates predominantly from this liquid source (Schoeninger et al. 2000). Their bone carbonate $\delta^{18}O$ composition therefore parallels the isotopic composition of the meteoric water that composes their drinking water. The high level of variability in both $\delta^{18}O$ and $\delta^{13}C$ seen in Figure 9.18 is

marked and shows a substantial number of individuals with depleted $\delta^{18}O$ values associated with higher elevation levels. This directly indicates residence at higher elevations for periods long enough to cause significant alterations in bone oxygen isotope signatures different from those typical for the valley floor (see discussion).

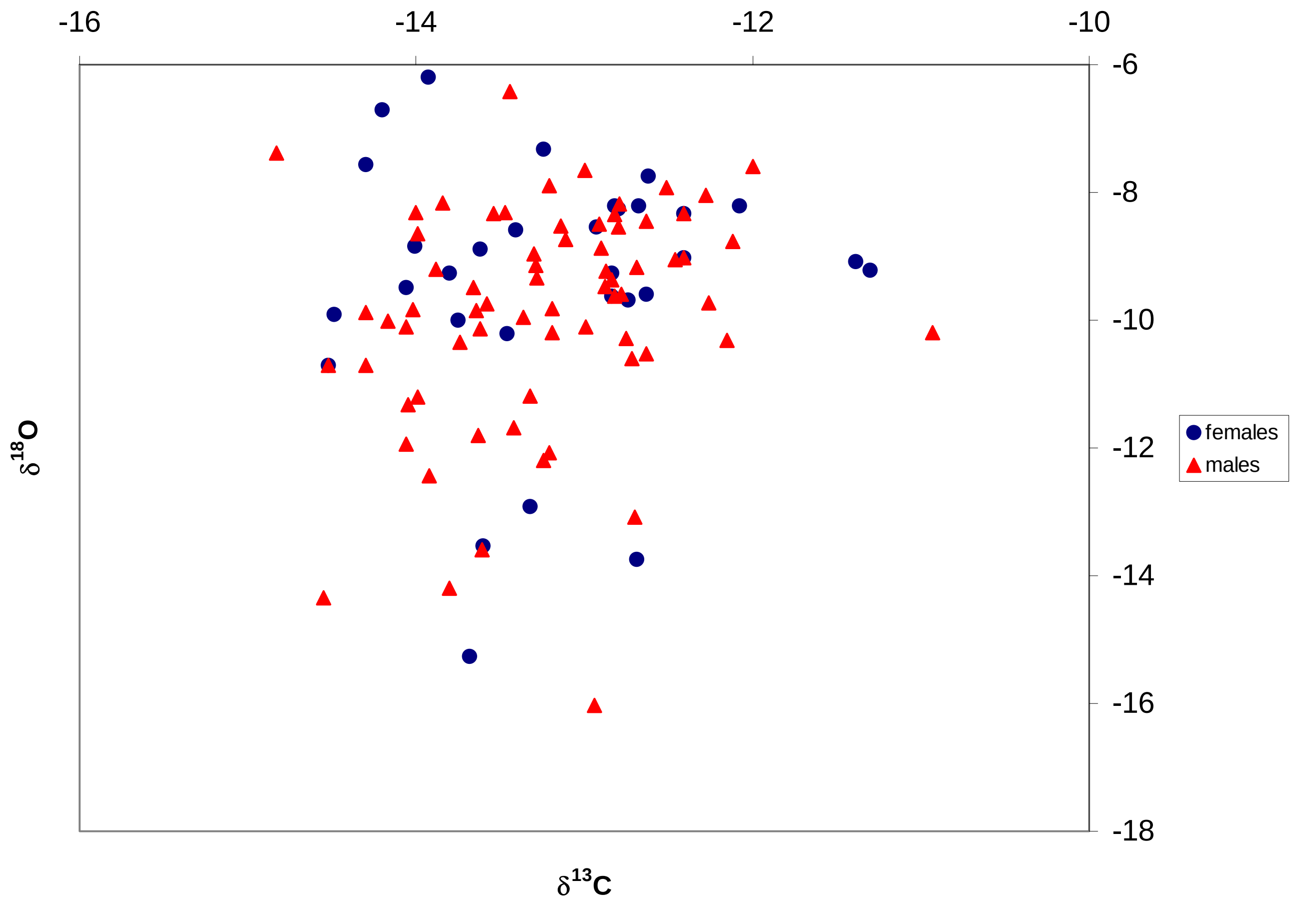

Figure 9.19. Comparison of the stable isotope ratios of oxygen and carbon from structural carbonate between adult males and females. There is an obvious contrast between the number of males exhibiting depleted ratios compared to females. A total of 35 ratios were found to be –10‰ or less, and of these, only seven belonged to females.

An illustrative comparison of the $\delta^{18}O$ and $\delta^{13}C$ in Figure 9.19 shows that the majority of those individuals believed to be residing at higher elevations due to very negative oxygen ratios were males. There were 19 individuals with a $\delta^{18}O$ of -11.0‰, which according to the OIPC estimates for the area, corresponds to roughly 1800 meters elevation. Fifteen of these people were males. This group must have regularly imbibed meteoric water that was heavily depleted in $\delta^{18}O$. The reservoir from which the drinking water was taken obviously was not subject to much of the natural mixing process along the mountain incline that would cause it to become more enriched. At these elevations only two main occupations come into consideration. Pastoral activities require the shepherds to remain for extended periods at higher elevations, especially with sheep herds which graze and search for vegetation at the highest altitudes. Mining in this area is not restricted to the high altitude areas, however, caches of rock crystal, a type of transparent quartz much coveted for jewelry, are often found at the higher elevations and therefore frequently sought after. The mining of metal ores such as copper and iron were also known to have been practiced at these higher elevations. However, because subsistence is a primary necessity, these individuals were more than likely mountain farmers, engaged in a mix of pastoral and agricultural activities. The highly situated alms around the Volders area also needed constant attention and care much the same way as a field is tended. This was accomplished with much toil and involved stone removal to clear pastures, provide irrigation to dryer spots and weeding to keep the alms free of unwanted shrubs and rapidly reproducing, less desirable vegetation (Hubatschek 1990). The most grueling and important work was the harvesting and transport of hay to storage shacks for the winter months. This work was not completed overnight and mountain farmers and shepherds stayed at high altitude for long periods or perhaps even year round.

Table 14. Subadult stable isotope carbon and oxygen ratios from bone structural carbonate. As with Table 13, C:N proportions were included as a measure for contamination.

Burial	Bone sampled	Sex	Age	Sample weight for lypholization (mg)	Extract (g)	Extract total %	C/N (molar)	$\delta^{13}C$ (PDB)	$\delta^{18}O$ (PDB)
12	femur	nd	1.5-2	101.3	60.2	59.4	3.27	-11.57	-8.77
20	femur	nd	5-7	102.1	61.0	59.7	3.65	-13.59	-8.3
21	cranium	nd	4-5	99.9	60.2	60.3	3.43	-13.43	-15.31
30	femur	tm	neonate	101.5	40.8	40.2	3.74	-15.21	-8.68
32	Rib	m	15±36m	101.9	57.9	56.8	-	-13.85	-8.03
40	femur	f	15±36m	100.0	39.9	39.9	-	-13.72	-12.33
46	cranium	nd	<1	101.1	48.6	48.1	3.71	-13.14	-9.4
47	tibia	nd	ca. 2	101.2	42.1	41.6	-	-14.06	-12.83
88	femur	nd	1-1.5	100.4	32.0	31.9	-	-15	-8.12
89	femur	nd	4-5	100.6	56.4	56.1	-	-13.75	-15.45
94	femur	f	7-9	100.3	57.3	57.1	-	-13.81	-9.48
98	cranium	m	15±36m	101.3	73.4	72.4	-	-13.41	-9.19
102	femur	nd	7-8	100.7	44.3	44.0	-	-13.71	-9.01
104	femur	f	16±24	101.0	35.6	35.2	-	-13.87	-9.56
115	humerus	nd	6-7	101.0	43.3	42.9	-	-13.26	-12.4
117	femur	nd	perinatal	100.0	50.7	50.7	-	-13.35	-9.21
119	tibia	nd	2-3	100.5	45.7	45.5	3.30	-14.63	-8.35
123	femur	f	18-19	100.6	19.5	19.4	3.69	-12.98	-8.64
124	femur	nd	6-7	100.7	49.5	49.2	-	-14.33	-15.16
133	humerus	nd	neonate	100.5	62.8	62.5	3.54	-13.8	-9.18
134	femur	nd	3-5	98.9	66.2	67.0	3.88	-13.92	-11.89
136	tibia	m	17-19	102.7	65.5	63.8	3.39	-12.68	-10.38
141	cranium	nd	5-7	100.2	57.3	57.2	3.29	-12.73	-8.59
152	femur	nd	<1	100.5	44.3	44.1	3.62	-14.56	-10.64

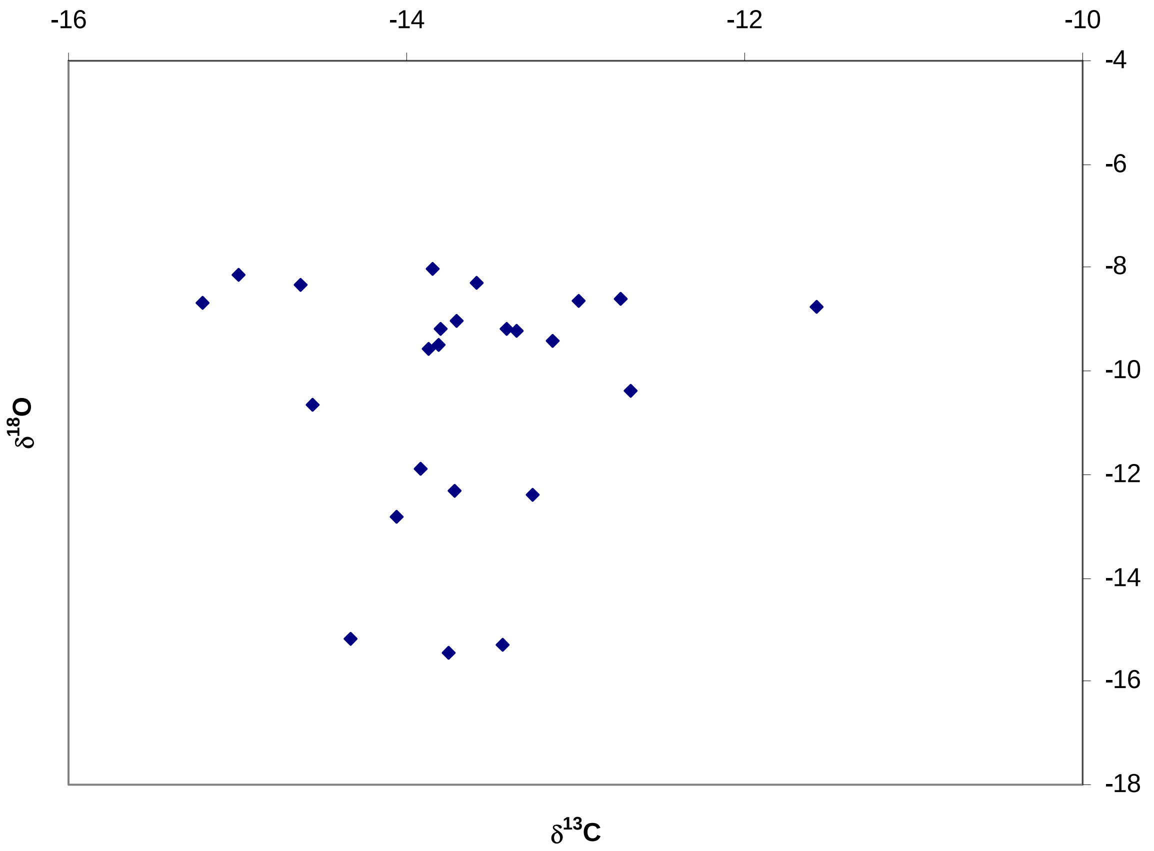

Figure 9.20. Subadult carbonate carbon and oxygen isotope ratios.

Figure 9.20 illustrates the dispersion of ratios among subadults. As with the adults, the ratios of the subadult group exhibit large variability, which appears to be related to age differences. There is a distinct tendency for the $\delta^{13}C$ ratios to become more enriched as the children get older, although it must be noted that here too, the variability of values is apparent (see Fig. 9.21). Infants who are breastfeeding acquire their water primarily through their mother's milk. Nursing infants have been shown to exhibit more elevated $\delta^{18}O$, a trophic level effect probably due to water fractionation during milk production, which draws from the mother's own body fluid (White et al. 2000). The level exhibited by a nursing infant, however, is to some extent dependant upon the mother's own isotopic signature. Water turnover in breastfeeding mothers is high and therefore, it might be expected that the isotopic signature of her milk should also reflect the meteoric water she drinks. Women living at different altitudes and drinking water containing different isotopic signatures, some of which is eventually converted into breast milk would explain the enormous degree of variability amongst the nursing age children observed at Volders (Fig. 9.22).

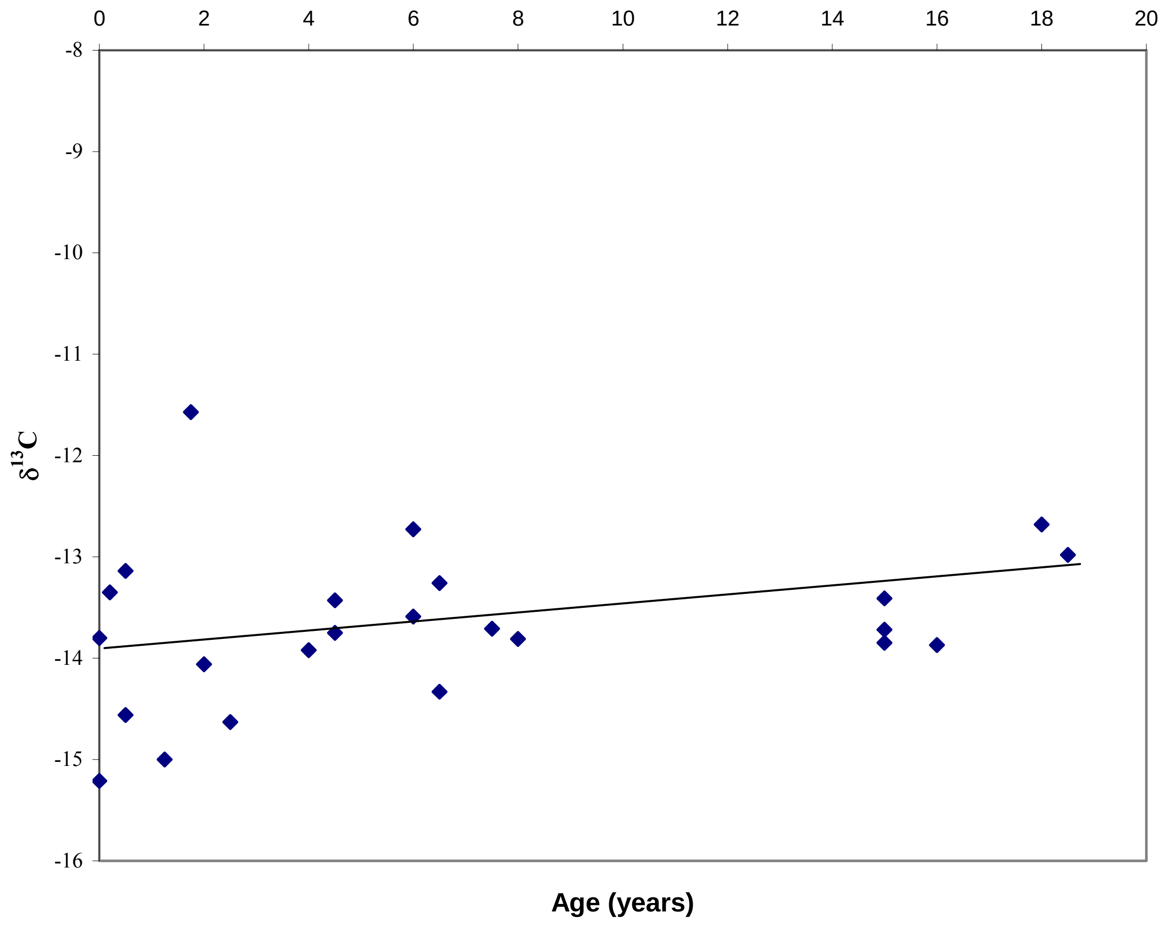

Figure 9.21. Scatter diagram showing the tendency of $\delta^{13}C$ values to become more enriched with increasing age.

As illustrated by Figure 9.22, only two of the children under four years of age (within lined rectangle) showed ratios characteristic for water derived from higher elevations (i.e. occupation at higher altitudes). The relatively enriched values indicated by the circle amongst the children under four years of age possibly result from the trophic effect caused by nursing. All of the ratios, however, fall well within the range of adults. Other subadults with enriched values might also have accompanied their parents who migrated to the area and therefore display values more positive than expected for the lowest elevation at Volders. In addition, the data does not clearly support the suggestion that youngsters were sent into the mountains as shepherds to tend herds. The implication of this finding is that there was no real necessity to reduce energy expenditure for the family group, which is critical for survival, as is witnessed for other cultures utilizing higher elevations to fulfill subsistence needs (Thomas 1976). The most depleted levels were observed for three children (burials 21, 89 and 124) all approximately of the same age, however, these children were certainly too young to have been employed for tending the herds.

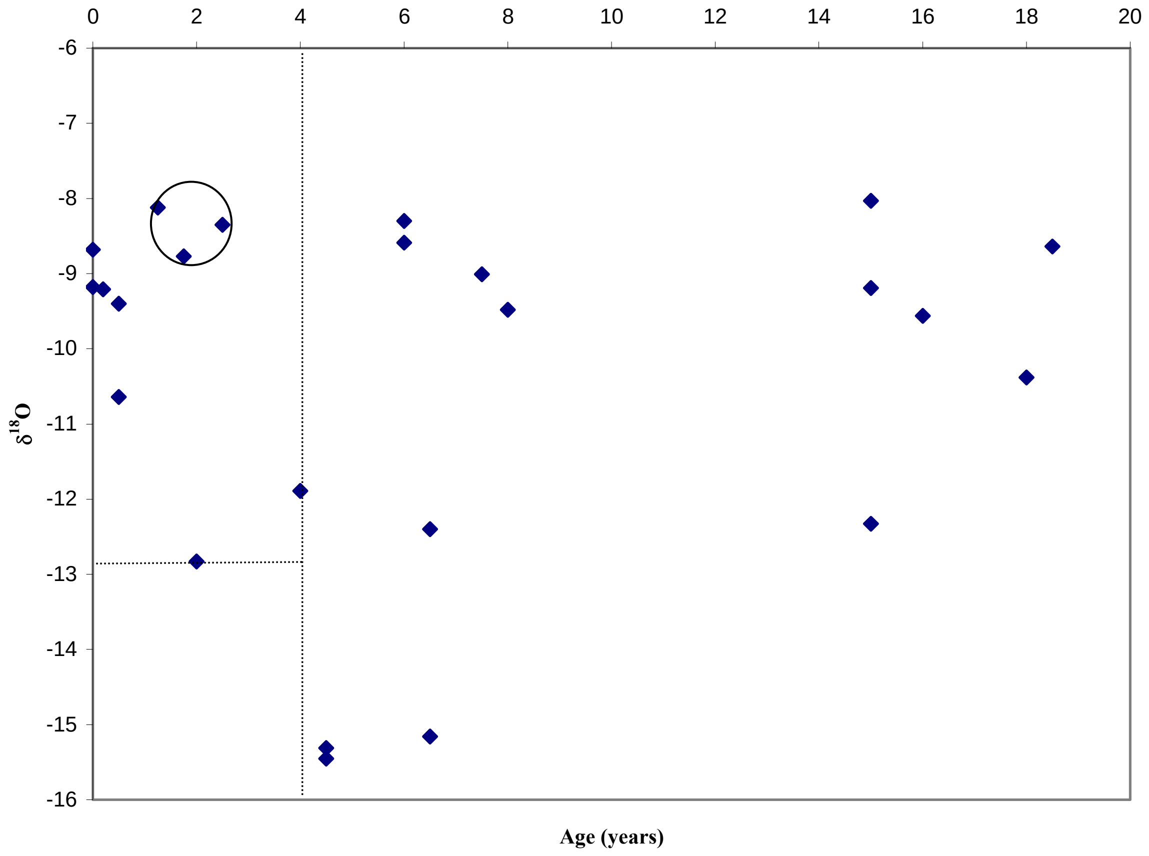

Figure 9.22. Diagram depicting subadult $\delta^{18}O$ values according to age. Borders are included to indicate children that may still have been nursing or were still being weaned.

The possibility of the existence of nutritional differences between individuals believed to have occupied higher altitudes with those who resided at lower elevations was also investigated. The criterion for the selection of the individuals belonging to the high elevation group (n=12) was a $\delta^{18}O$ isotope value of -11.56‰ or lower, which was one standard deviation above the mean for the entire group. A two sample t-test showed a significant 0.05 level difference in $\delta^{13}C$ between the high altitude and the low altitude groups. There was no significant relationship between $\delta^{13}C_{sc}$ and $\delta^{15}N$ from bone collagen and $\delta^{18}O$ ratios. It was, however, remarkable that all of the extraordinarily high nitrogen levels that were above 10.5 (n=15), were found in individuals inhabiting low altitudes.

A statistical comparison of the carbonate and related collagen isotopes for the high altitude group and the remaining Volders population is presented below in Tables 15 and 16.

Table 15. Two sample t test indicating means, standard deviation and t for carbonate carbon and collagen carbon and nitrogen between high altitude individuals (those with ^{18}O ratios one standard deviation above the mean) vs. the remaining population.

	High altitude (n=12)		Low altitude (n=63)		t 0.5
	Mean	SD	Mean	SD	
$^{13}C_{carbonate}$	-13.44	-0.53	-12.86	-1.69	2.18*
$^{13}C_{collagen}$	-19.7	-0.43	-19.98	-0.63	1.89**
$^{15}N_{collagen}$	-9.88	-0.36	-10.02	-0.82	0.97**

*Significant at the .05 level

**Not significant at the .05 level

Table 16. Two sample t test indicating means, standard deviation and t for $\delta^{18}O$ ratios in adult males vs. females.

	Males (n=47)		Females (n=21)		t 0.5
	Mean	SD	Mean	SD	
$\delta^{18}O$	-10.5	-1.26	-9.4	-2.07	1.30*

*Not significant at the .05 level

OIPC $\delta^{18}O$ estimates for differing altitudes

To understand the placement of the human oxygen isotope ratios within the ecosystem they were once formed in, an estimation of levels in nature in Volders is necessary. Table 17 is a simple list of $\delta^{18}O$ ratios to varying altitudes as calculated using the OIPC. The figures listed are present day oxygen isotope value estimations for consecutive increments in altitude of 100m. Estimates were made using the VSMOW as the standard for latitude 47.3° and longitude 11.8°. The linear relationship between altitude and $\delta^{18}O$ ratios under ideal conditions is illustrated in Figure 9.23. This type of relation is naturally affected by the fractionating influences exerted by both exogenous and endogenous factors such as atmospheric variability, ground geology and an organism's physiology respectively.

Table 17. Present day estimations for oxygen isotope values in meteoric water corresponding to different altitudes for the geographical coordinates at Volders, Austria according to the OIPC. * Approximate elevation of Volders above sea level.

**Altitude (m)**	**δD‰**	**δ^{18}O‰**		**Altitude (m)**	**δD‰**	**δ^{18}O‰**
2500	89.59	-12.69		1600	76.64	-10.93
2400	88.15	-12.49		1500	75.20	-10.74
2300	86.71	-12.30		1400	73.76	-10.54
2200	85.27	-12.10		1300	72.32	-10.35
2100	83.83	-11.91		1200	70.88	-10.15
2000	82.39	-11.71		1100	69.44	-9.96
1900	80.95	-11.52		1000	68.00	-9.76
1800	79.52	-11.32		560*	61.67	-8.90

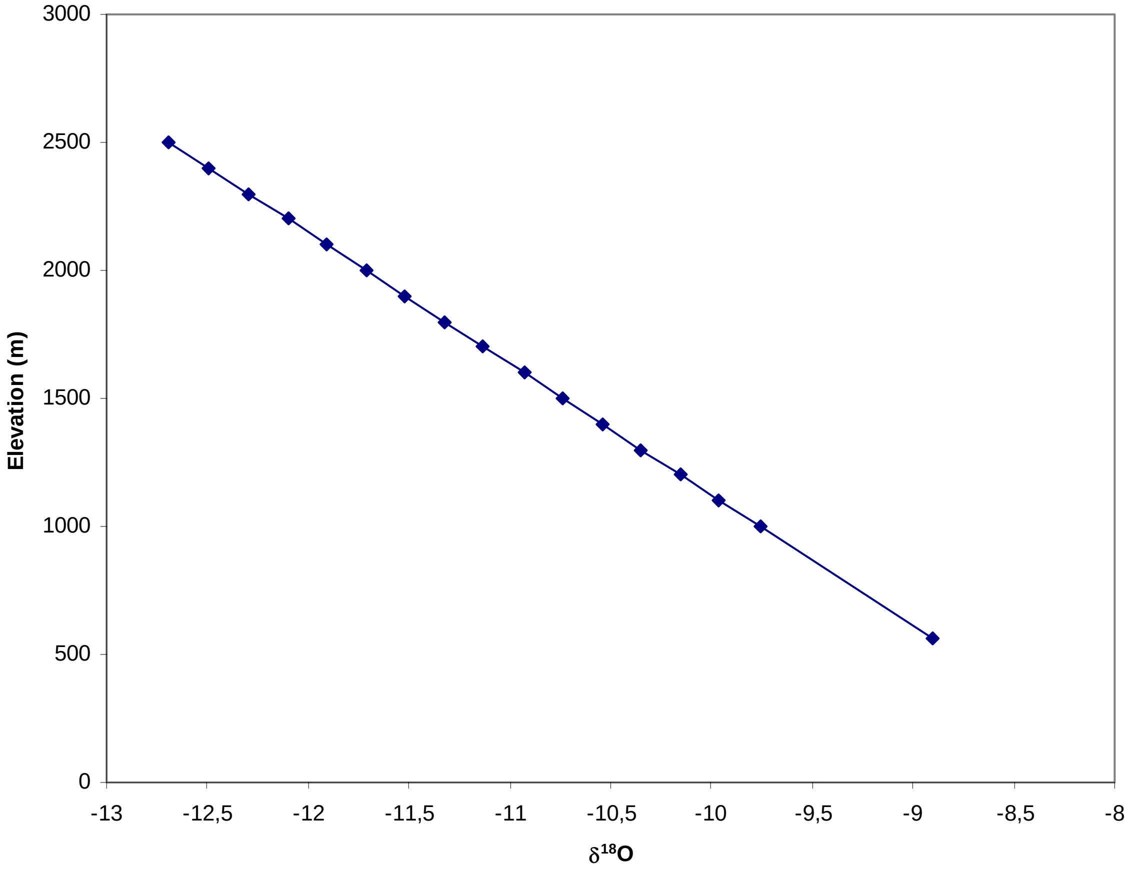

Figure 9.23. Illustration of the simple linear relationship between elevation and $\delta^{18}O$ values listed previously in Table 16. An increase in altitude coincides with more depleted (more negative) levels of $\delta^{18}O$ (‰) in meteoric water. Theoretically these varying signatures are passed on to the organism dependant on this meteoric water for drinking.

9.7 Carbon spacing

The carbon spacing values are useful to investigate degree of herbivory and carnivory, and also to help differentiate between food choices, for example, animal proteins, fats and plant carbohydrates. The data presented in Table 18 for adult individuals at Volders shows a mean of 6.79‰, which is within the typical range for an omnivorous diet based on C_3 plant foods and C_3 herbivore meat (Krueger & Sullivan 1984). Burial 49 exhibits a distorted value possibly indicating diagenetic alteration and was eliminated from the calculation.

Table 18. Δ = $\delta^{13}C$(carb) - $\delta^{13}C$(coll) for all individuals at Volders. Shaded cells indicate subadults.

Burial	$\delta^{13}C$(carb)	$\delta^{13}C$(coll)	Spacing‰	Burial	$\delta^{13}C$(carb)	$\delta^{13}C$(coll)	Spacing‰
1	-12.00	-19.72	**-7.72**	67	-12.99	-19.99	**-7.00**
2	-14.34	-20.20	**-5.86**	70	-13.19	-19.49	**-6.30**
3	-13.21	-20.18	**-6.97**	74	-12.69	-19.26	**-6.57**
4	-14.83	-20.10	**-5.27**	77	-13.99	-20.25	**-6.26**
6	-13.00	-19.65	**-6.65**	81	-12.78	-19.84	**-7.06**
7	-12.79	-19.74	**-6.95**	82	-14.05	-19.78	**-5.73**
9	-12.62	-20.04	**-7.42**	83	-11.30	-19.95	**-8.65**
9b	-13.95	-20.18	**-6.23**	84	-14.03	-19.75	**-5.72**
10	-12.51	-19.59	**-7.08**	91	-13.74	-20.20	**-6.46**
11	-12.08	-19.08	**-7.00**	93	-13.62	-20.00	**-6.38**
12	-11.57	-19.19	**-7.62**	99	-12.74	-20.17	**-7.43**
13	-12.82	-19.89	**-7.07**	100	-13.88	-20.51	**-6.63**
14	-14.01	-20.29	**-6.28**	101	-13.75	-19.99	**-6.24**
15	-13.24	-20.20	**-6.96**	103	-12.87	-20.06	**-7.19**
16	-13.84	-20.10	**-6.26**	107	-12.88	-19.91	**-7.03**
17	-12.12	-20.17	**-8.05**	108	-13.32	-20.28	**-6.96**
18	-14.02	-20.18	**-6.16**	110	-13.58	-19.5	**-5.92**
19	-12.80	-19.97	**-7.17**	113	-13.19	-19.9	**-6.17**
20	-13.59	-19.52	**-5.93**	114	-12.93	-19.85	**-6.92**
21	-13.43	-19.91	**-6.48**	116	-13.99	-20.21	**-6.12**
23	-12.82	-20.11	**-7.29**	119	-14.63	-19.54	**-4.91**
24	-12.15	-19.92	**-7.77**	120	-12.94	-20.14	**-7.20**
25	-14.55	-19.93	**-5.38**	121	-13.54	-20.41	**-6.87**
26	-13.68	-20.14	**-6.46**	123	-12.98	-19.77	**-6.79**
27	-13.28	-20.45	**-7.17**	129	-14.49	-20.18	**-5.69**
28	-12.41	-20.52	**-8.11**	132	-13.29	-19.48	**-6.19**
30	-15.21	-20.59	**-5.38**	133	-13.80	-19.82	**-6.02**
35	-12.63	-19.67	**-7.04**	134	-13.92	-19.66	**-5.74**
36	-12.63	-19.81	**-7.18**	136	-12.68	-19.52	**-6.84**
38	-13.44	-19.77	**-6.33**	137	-12.75	-20.02	**-7.27**
41	-12.68	-19.55	**-6.87**	138	-10.93	-20.36	**-9.43**
42	-12.72	-20.39	**-7.67**	140	-12.26	-20.18	**-7.92**
43	-14.20	-20.19	**-5.99**	141	-12.73	-21.06	**-8.33**
44	-13.78	-19.72	**-5.94**	142	-14.52	-19.95	**-5.43**
45	-12.63	-19.46	**-6.83**	143	-12.84	-20.3	**-7.46**
46	-13.14	-18.74	**-5.60**	144	-13.92	-20.09	**-6.17**
48	-12.70	-19.42	**-6.72**	145	-13.14	-20.23	**-7.09**
49	-0.84	-19.40	**-18.56**	146	-12.84	-19.49	**-6.65**
56	-12.82	-21.24	**-8.42**	148	-13.21	-18.75	**-5.54**
57	-12.46	-20.15	**-7.69**	149	-14.30	-19.52	**-4.41**
59	-13.61	-20.35	**-6.74**	150	-13.32	-19.87	**-6.55**
60	-13.80	-19.84	**-6.04**	151	-13.42	-19.54	**-6.12**
61	-13.24	-19.85	**-6.61**	152	-14.56	-20.00	**-5.44**
64	-13.56	-20.25	**-6.69**	153	-12.69	-20.09	**-7.40**

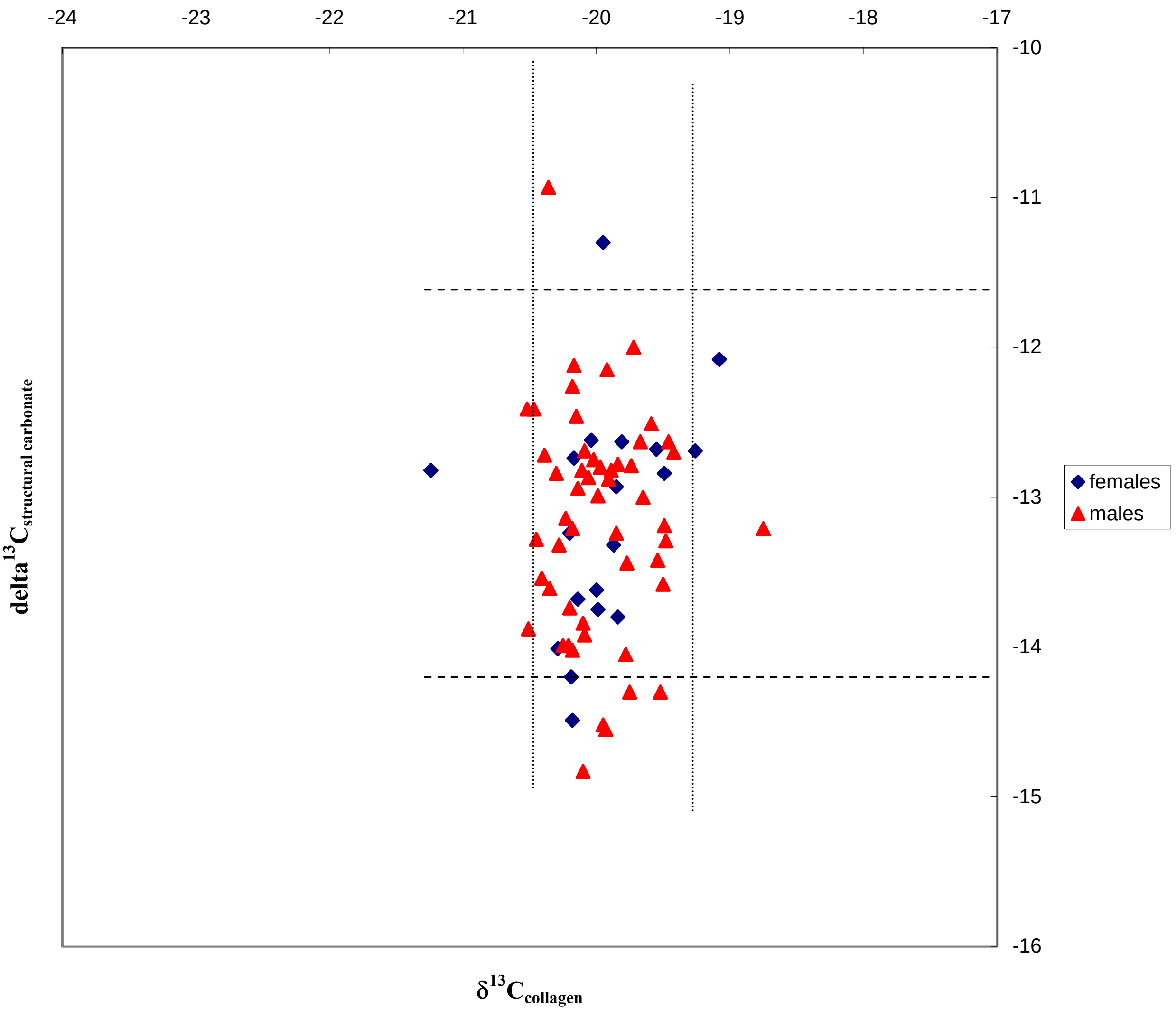

Figure 9.24. Scatter diagram illustrating the $^{13}C_{structural\ carbonate}$ to $^{13}C_{collagen}$ plot for all adult individuals at Volders. Due to poor collagen preservation, burials 113 and 114 were excluded, as were adult individuals whose sex was not identifiable. Lines indicate the clustering of ratios that are characteristic for agriculturally based populations.

Figure 9.24 plots $\delta^{13}C_{carbonate}$ against $\delta^{13}C_{collagen}$, which clearly illustrates the existence of a pattern typical for agriculturally based nutritional intake. The majority of $\delta^{13}C$ collagen ratios fall in between -19.5‰ and -20.5‰, and the $\delta^{13}C_{carb}$ range between -12‰ and -15‰. A total of 17 individuals have $\Delta(\delta^{13}C_{carb} - \delta^{13}C_{coll})$ values below 6‰, with two of these are even below 5‰, indicating that they were consuming more animal based foods, perhaps dairy products, since the lipids in this food type are heavily depleted in ^{13}C and functions to reduce the carbon spacing value significantly (Asam et al. 2004). The low level of variability for $\delta^{13}C_{coll}$ is readily apparent in Figure 9.24, in contrast to those of $\delta^{13}C_{carb}$, which shows a range of up to 3‰. The more negative ratios for $\delta^{13}C_{carb}$ may indicate the inclusion of food stuffs

like wine, imported olive oil or other consumables with more depleted levels of ^{13}C (Prowse et al. 2005).

The series of diagrams depicted by Figures 9.25a-c illustrate the carbon spacing difference for adult individuals at Volders. A total of 18 individuals (burials 2, 4, 20, 25, 30, 42, 44, 46, 82, 84, 110, 119, 129, 134, 142, 148, 149 and 152) displayed a carbonate to collagen difference in carbon below 6, which is characteristic for diets including more animal based fats. Only six values were found to exceed a difference of 8, although one of these (burial 138) reached a difference of nearly 9.5, which indicates a diet based less on animal products and more on vegetable foods.

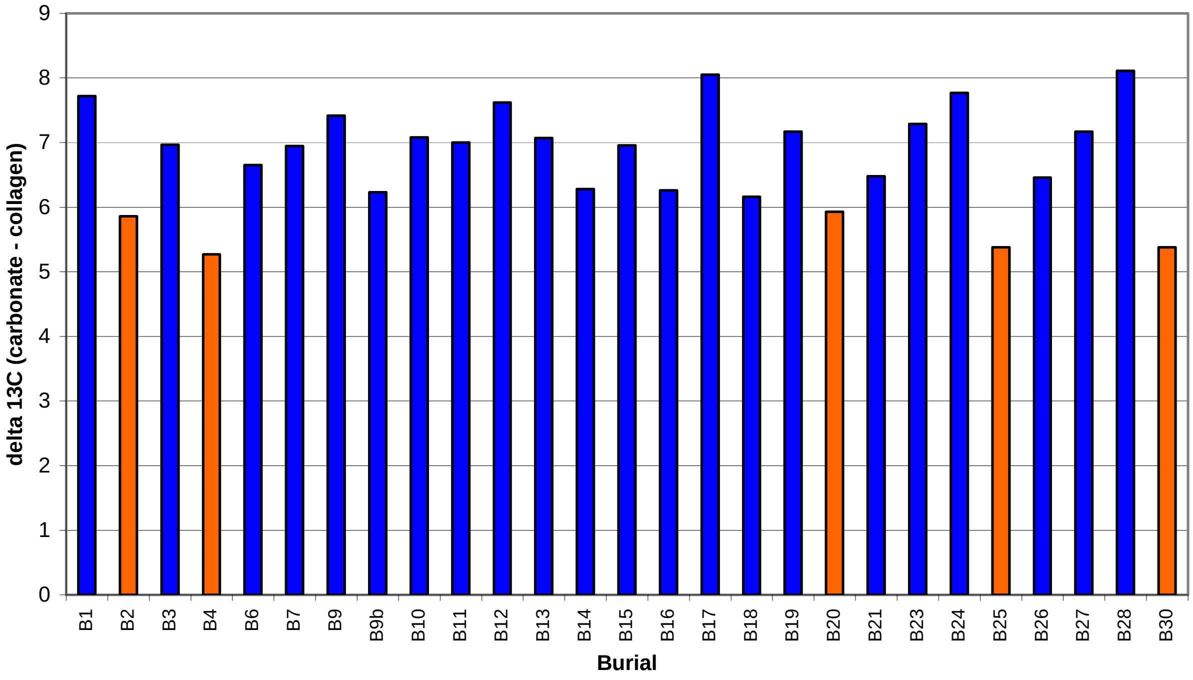

Figure 9.25a. This diagram shows the carbon spacing values for Volders burial numbers between 1 and 30. Orange lines indicate values that fall below a spacing difference of 6, implying a diet more dependant on lipid rich animal products.

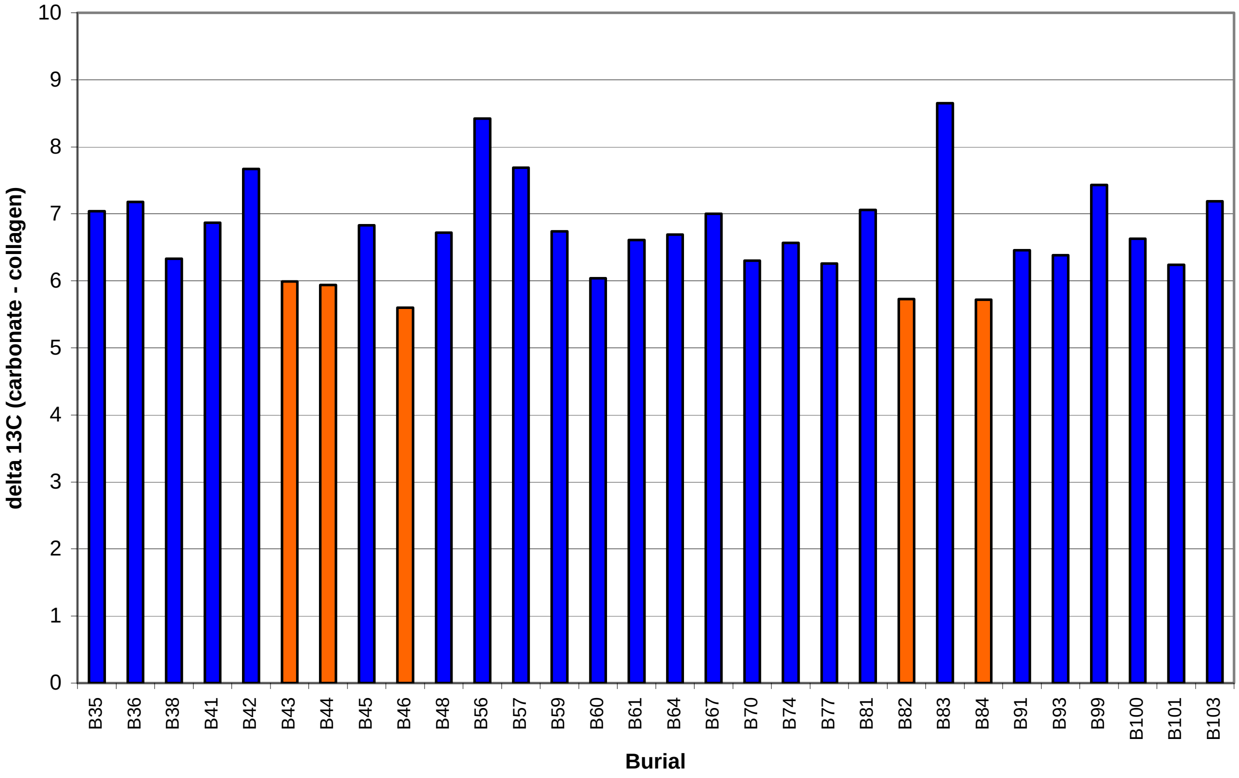

Figure 9.25b. Same diagrammatic depiction as for 9.25a showing spacing values for burial numbers between 35 and 103.

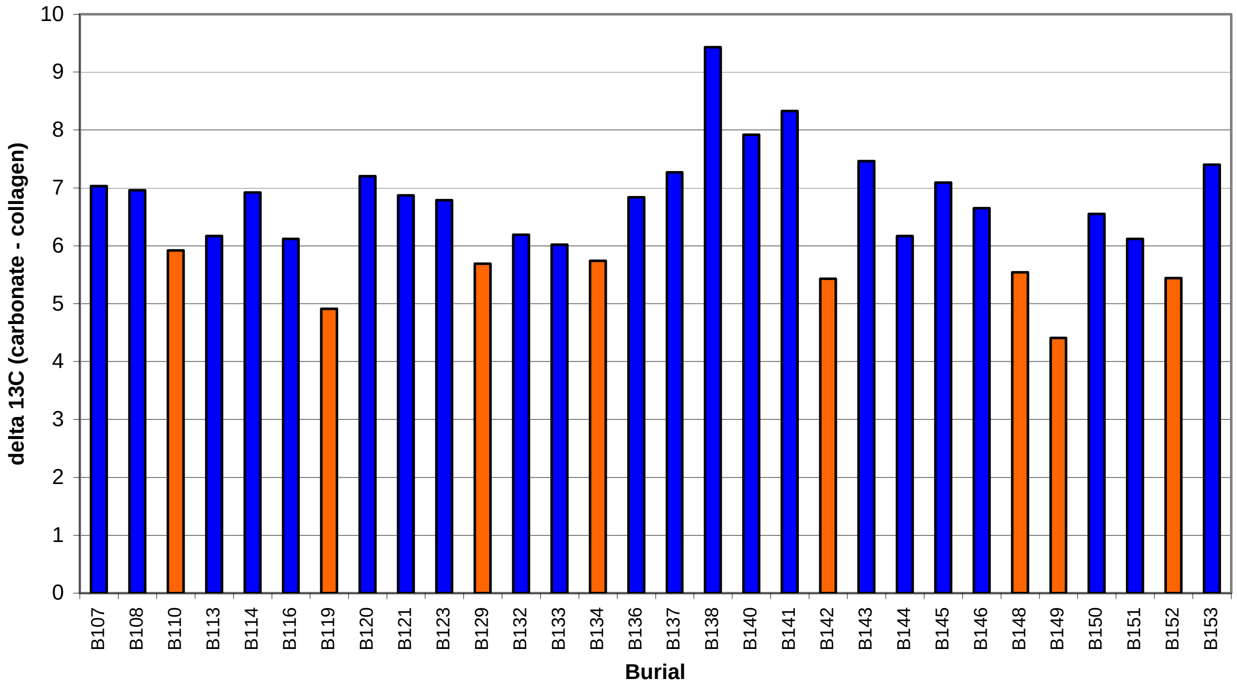

Figure 9.25c. Continuation of previous two diagrams showing burial numbers between 107 and 153. Two of the individuals depicted show spacing values even below 5 (burials 119 and 149). Burial 138 on the contrary, exhibits a wide spacing value (9.43) indicative of a more vegetarian based diet.

The negative correlation between $\delta^{15}N$ and $\Delta(\delta^{13}C_{carb} - \delta^{13}C_{coll})$, which would normally be expected was not apparent, and most of the highest nitrogen ratios in adults were found in between 6-8‰ (see Figure 9.26). The exception was seen in the values observed for subadult individuals.

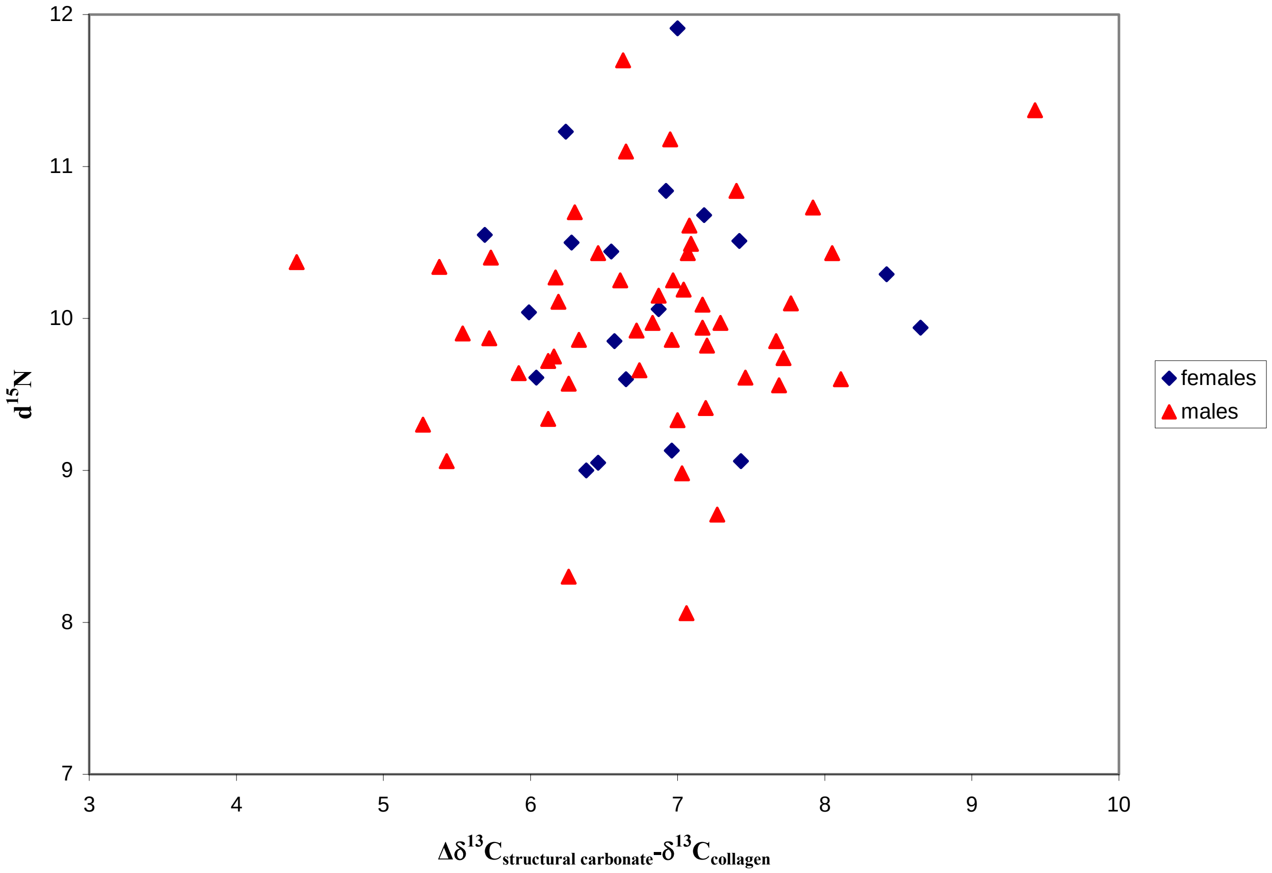

Figure 9.26. Scatter diagram plotting $\Delta(\delta^{13}C_{carb}$-$\delta^{13}C_{coll})$ against $\delta^{15}N$ for adult individuals at Volders.

The mean $\Delta(\delta^{13}C_{carb} - \delta^{13}C_{coll})$ for subadults was found to be 6.25‰, also suggesting an omnivorous diet similar to that of the adults. The scatter diagram in Figure 9.27 and bar diagram 9.28 both show a general distribution very similar to that observed for the adult individuals. This further suggests that the subadults of non-nursing age were consuming the same foods as the adults in the population since there is no significant difference in carbon spacing values between the two groups. In addition, children who are presumed to have been nursing at the time of death (Burials 12, possibly 30, 46, 119, 133 and 152) were not clustered and exhibited a large variability in $\delta^{13}C_{carb}$ ratios, i.e. there is no correlation between high $\delta^{15}N$ and more negative $\delta^{13}C_{carb}$ ratios. However, according to the carbon spacing evidence the infants and children exhibiting a difference below 6 as depicted in Figure 9.28 were all of an age where nursing is plausible. The only exception is burial 30, which is thought to be a newborn. This would suggest nursing until an age of up to approximately 3 years. The age of burial 134, which sets the upper age limit to this small group, is listed as 3-5 and based only upon femur length, rendering it fairly unreliable. The burials which did not exhibit a carbon

spacing difference of 6 or less included 12, 20, 21, 123, 133, 136 and 141. Interestingly, with the exception of burial 12, a 1.5-2 year old child, all of these individuals were of an age in which the cessation of nursing is presumed to have already occurred. Burial 133 is listed as a newborn and it is unlikely that the infant was already nursing.

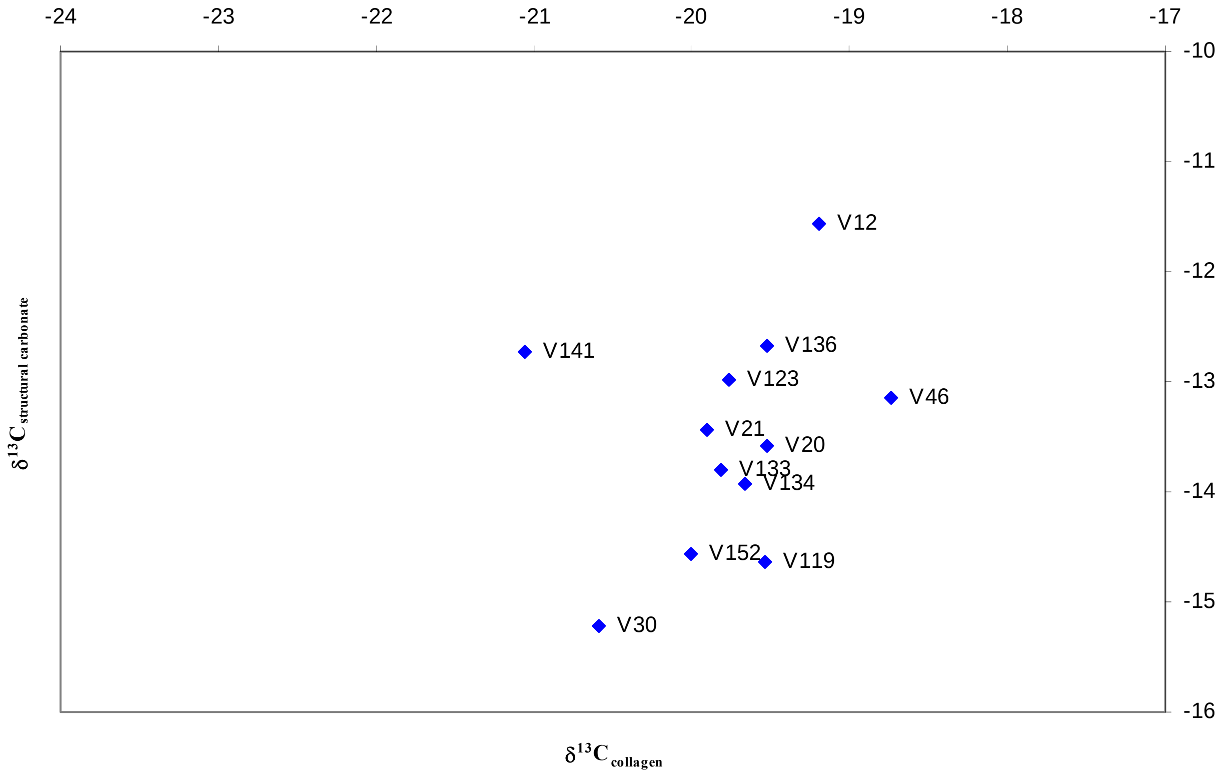

Figure 9.27. Scatter diagram of the Volders subadult $\delta^{13}C_{sc}$ vs. $\delta^{13}C_{coll}$. Individual burial numbers are given.

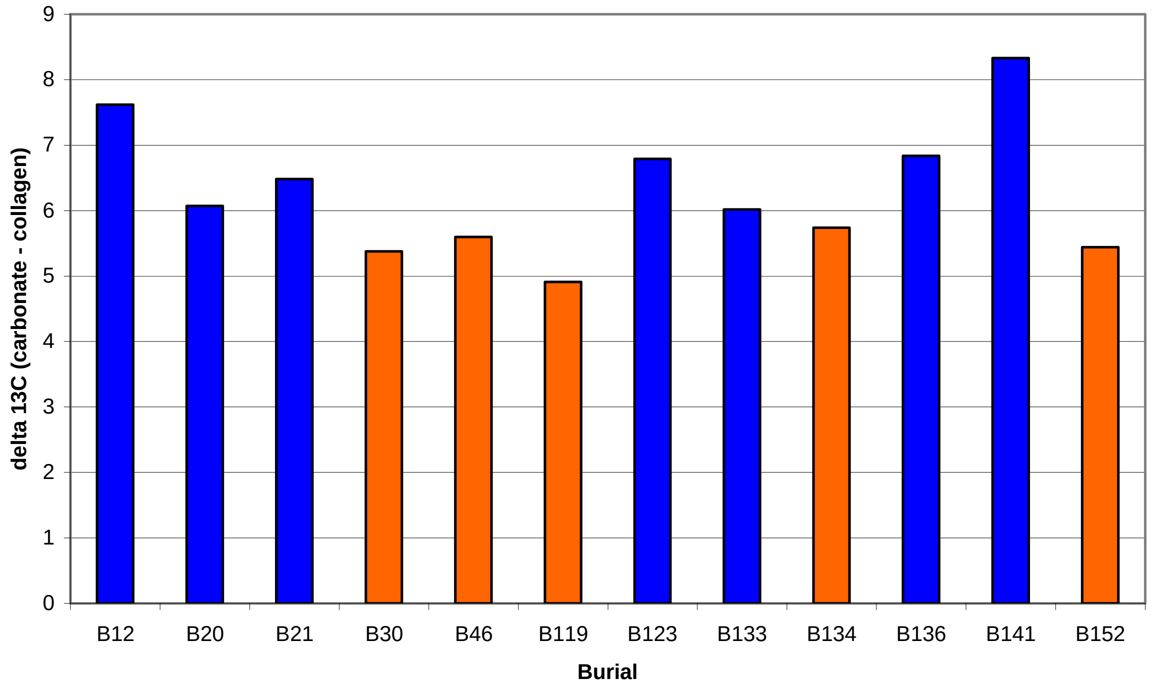

Figure 9.28. Subadult carbon spacing with children under a spacing difference of 6 shown in orange.

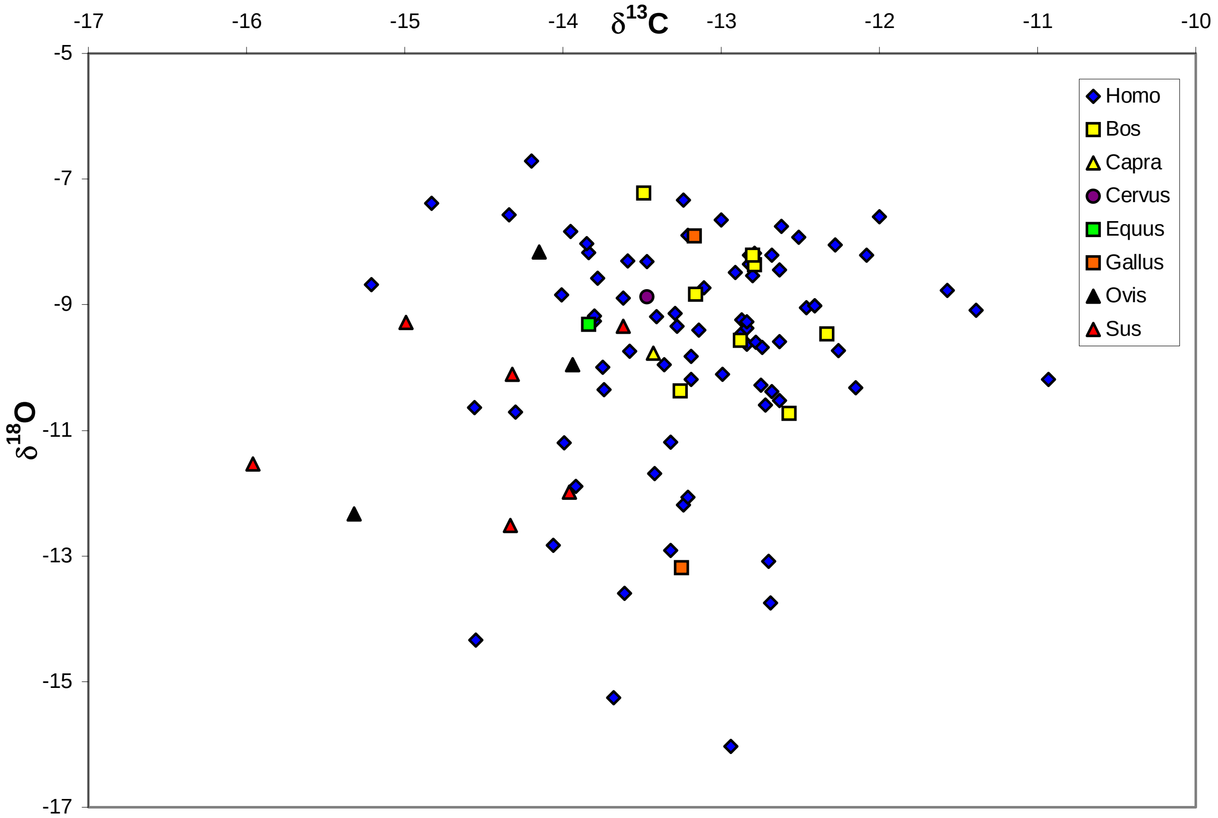

Figure 9.29. Comparison of faunal and human carbonate data.

The majority of animals used for sampling are domestics and are therefore expected to live near their owners, especially in a time were predation of herds by wolves and bear was still a very real threat. According to Sponheimer & Lee-Thorp (1999b) carnivores have skeletal $\delta^{18}O$ values similar to their dominant prey, therefore, people consuming animal products should also have oxygen signatures that parallel the animals and animal products that they consume to some extent. This presumption is supported by Figure 9.29, which shows that human and animal values intermingle and even overlap. In addition, it would appear that cattle were either not driven to higher elevations or that their stay was too brief to result in any real change in isotopic signature for oxygen. Longer stays or permanent grazing at high altitude would otherwise cause them to exhibit the depleted levels characteristic for these higher elevations.

10 Discussion

The discovery of the early medieval cemetery at the alpine village Volders and the subsequent investigation of the 145 skeletal remains afforded the unique opportunity to take a closer look at certain aspects of the palaeodemography of this past population. By employing a combination of osteological and archaeometric methods including $\delta^{13}C$, $\delta^{15}N$ and $\delta^{18}O$ stable isotope analysis, amino acid assays, and the histological examination of tooth root cementum (TCA), new insights to these past people have been gathered.

Initial, gross observations during the morphological examination involved assessing the level of skeletal preservation. Macroscopically, skeletal condition at the Volders site was heavily impacted by numerous taphonomic factors including bad weather, construction work (present and past), inexperienced excavation workers, burial surroundings as well as factors intrinsic to the bones structure itself. Together, these resulted in the partial or total destruction of numerous skeletons, leading to the irretrievable loss of information.

At the molecular level, diagenetic alteration of bone substance destined for chemical analysis also proved to be a critical hurdle for the analyses of stable isotopes. Approximately 30 samples of extracted collagen produced no result whatsoever. Of the successful collagen extractions, only three human samples and one faunal sample were deemed unusable based upon the C:N ratios. It should be noted, that ratios of up to 3.9 were used in the analysis, a level which other researchers consider too high because of the possibility of contamination, however, archaeological material does often tend to exhibit slightly higher C:N ratios and a certain amount of flexibility was granted here. The random amino acid assay tests also reaffirmed the integrity of the collagen samples, all of which produced results within the range of acceptability set for this study. A comparison of $\delta^{13}C$ with the cristalinity index for carbonate preservation was not conducted, however, potential diagenetic alteration was compensated for by careful removal of exogenous carbonates prior to analysis. The evidence gathered in this study indicates that this population displayed a high level of fitness and was well adapted to the alpine environment

10.1 Palaeodemography

In the absence of written documentation, the data necessary to establish the demography of a past population must be acquired through osteological examination of the recovered skeletal remains and of course through interpretation of the associated artifacts. This differs from the tools used by modern researchers interested in the demographics of a population differ from the osteologically based type used for a palaeodemographic study and include census,

surveys, and registrations systems and the statistical analysis of the data deriving from them (Newell 1988). Data variables accessible through this type of examination and investigated in the present study are the total number of individuals in the complex (not the population), sex, age at death, clues to health status, diet, mobility and migration.

Hassan (1981) indicated the dynamics of a population comprise the various areas of interest for demographic investigation and include population characteristics such as size, growth, cycles in births and deaths, sex composition, age, migrations, disease and epidemics. Scott and Duncan (1998) have shown that these same factors affect the overall population dynamics of a single community and, in turn, may be explicable in terms of its demography, geographical location and local economy.

One of the most common problems associated with a palaeodemographic study is to what extent the human skeletal remains being examined represent the populations from which they originate. They are cumulative aggregates usually containing multiple generations of individuals that died at different times, through different circumstances, and have different life histories, and are not true biological populations per se (Larsen 1997). Biological, cultural, and social factors can all potentially lead to biases that influence palaeodemographic interpretations. Also, inaccuracies in age estimation and sex identification can lead to highly distorted demographic profiles.

When conducting a demographic study, large samples relative to the actual population size are preferable to small ones. It is not known exactly how many people lived in the early Middle Age village of Volders, however, the population segment represented by the Volders skeletal sample is the largest yet available for that period. Nonetheless, inferences to the community's demographic makeup have been carefully considered because the actual population size of the village is unknown.

Demographic information elucidated from this study will lead to a better understanding how well these people adapted to their environment (Leslie & Gage 1989).

Sex

The sex determination of the adult individuals represented in the skeletal series at Volders produced a remarkable result with respect to the proportion of males to females, with males outnumbering females by 2 to 1. The disproportionately large number of males might have indicated a preferential burial area for men, however, this is not supported by the archaeological documentation, which shows no sex related pattern to burial practice. Another possible explanation for this observation is the existence of a concentrated work force

involved in a male dominated secular activity such as mining or possibly work related to commerce. There is no archaeological or historical evidence for a cloister, so support for the existence of a male oriented religious community is also lacking. The reason for the predominance of men at this location remains, at the moment, unsolved, although it should be noted that this skeletal series is far from complete since a large portion of the cemetery was destroyed by the earlier construction of the neighboring house and adjacent road.
Sex determination for the subadult group proved to be an insurmountable problem due to poor bone preservation, especially among infants. The bones of young children, infants and even fetuses already display some of the characteristics used for sexing that gradually become more pronounced as the person develops (Fazekas & Kósa 1978), however, this diagnosis is not uncomplicated and the accuracy is accompanied by the dangers of misinterpretation even when the skeleton is complete and in good condition.

Child deficit

The deficit of infant skeletal remains often reflects their increased susceptibility to the deleterious effects of taphonomic processes. In areas like the Inn Valley, where the domination of Roman influence was ubiquitous even long after the demise of the Empire, there was also the tendency to bury infants apart from the adults, for example, in the vicinity of a church or in or near structures like a house or pathways frequented by family members (Struck 1993). This burial custom, which was practiced earlier by Romans, must be taken into consideration for any archaeological cemetery situations dating from or around this time. The fact that several infant skeletons were recovered indicates that this burial custom may not have been practiced at Volders. It is more likely that the proportionally small number of adult females represents the decisive factor behind this observation, since the number of reproductive females in a population has a direct influence upon the number of infants.

Ageing

Information to the age at death was based upon a combination of the morphological examination and the analysis of tooth cementum (see 10.2). The TCA analysis also frequently aided in the determination of age at death in situations where the morphological examination was only able to provide a rough estimation due to poor preservation of the requisite bone features. The life table presented in section 9 indicates a slope characteristic for a living population and there does not appear to be a particular segment of the population that had a significantly higher risk rate for death. What is remarkable, however, is that many adults at

Volders reached a very old age, a circumstance not often associated with the medieval period. The reason for this frequent observation is caused primarily by the tendency to underestimate the ages not only of young adults but also the older individuals over 60 yrs of age during the morphological examination (Cipriano-Bechtel et al. 1995). The TCA analysis provides the opportunity to make more concise the broad morphological age categories normally employed in archaeoosteology.

Stature and physical characteristics

The morphological examination also revealed that physically, with several exceptions, the adults from Volders are neither remarkable for their height or physique and fall within the stature type typical for the area at this time period. The frequently observed skeletal characteristics such as well developed points of muscle attachment and increased robustness of bone structure both indicate that the Volders population was a physically active one and not unaccustomed to strenuous work. Although cranial measurements were generally not possible because of their widespread destruction, the overall morphological appearance of the preserved skulls and of those where a reconstruction was possible, showed an elliptical form typical for the early medieval row burial period of this area (Hug 1940).

Health

The health of a population exerts a powerful influence upon its wellbeing and success. Since nutrition plays a vital role in the health of not only the individual but also the population as a whole, the information acquired through the analysis of stable isotopes that reflect aspects of diet can potentially yield important indices to this demographic variable (see Palaeodietary reconstruction, this section). According to Larsen (1997), skeletal signatures of morbidity alone are insufficient to establish the level of a community's health and it is essential that other lines of evidence, including subsistence and settlement, environmental context, social and cultural contexts, and population structure be taken into account in interpreting levels of health. The examination of skeletal remains for evidence of sickness or trauma remain, however, vital to establishing the health status of a past group or population and through diligent examination and interpretation, skeletal data can provide useful indices of the human condition.

There are, however, many infectious diseases that do not leave visible traces on the skeleton. Infectious diseases were certainly an important dynamic feature of the demography of early medieval populations in the Inntal like Volders at a time when vaccinations and efficacious

medical treatment were not available. This risk of illness is even more apparent, especially when the interregional migration of people unwittingly introducing new pathogens is taken into consideration. Based upon archaeological findings, a small group of those interred at Volders (burials 32, 36 and 37) gave the impression that they were buried together or within a very short period of time from one another. This suspicion rests on the observation that the skeletons lay in close proximity to each other with overlapping extremities and also the lack of clearly definable individual grave outlines. Substantiating this will only be possible following the interpretation of the burial situation profiles, which are in progress and expected to be complete in 2007. It is well known that diseases become mobile and are transferred over long distances because of population movement. Contagions and carriers move to new areas, resulting in epidemics or worse still, pandemics (Scott & Duncan 1998). On the other hand, when the mobility involves people entering an infected area that exposes them to new contagions, a similar situation can arise. Colonization, trading voyages and military expeditions in the 19th century were all subject to the brunt of “strange climates” (Curtain 1995).

The pattern of health observed for the Volders skeletal group probably reflects the overall demographic situation of the once living population for that given time period. It is presumed that this late Roman-early medieval population was rural. The osteological examination revealed that the human remains exhibited a remarkably low rate of skeletal trauma for a working people. The low rate of bone infections, symptoms of metabolic disorder related to malnourishment, or other pathological conditions further suggests that the Volders population was a relatively healthy one. The question arises, whether a portion of these people were more involved in trade traffic and crafts than agrarian work.

Dental status can provide information not only regarding food consumption by the presence of tooth wear and caries, but is also useful for identifying stress factors related to childhood nutritional deficiencies and illness in the form of enamel hypoplasias. A very low rate of dental caries (approx. 15% of intact dentitions), yet, high rate of tooth crown attrition (over 80% of adults), implies the consumption of poorly milled grain foods. The inclusion of stone powder ground off the milling stone into the flours led to the aggressive abrasion of tooth enamel beginning at an early age. The deciduous teeth of children often exhibited significant crown wear, which also indirectly indicates that children were fed diets containing grain foods. Dental abscesses were rare and it appears that the abrasive nature of the food consumed and of course the lack of refined sugars, resulted in a reduction of caries and other periodontic diseases. Enamel hypoplasias, which are caused by a disruption of normal tooth crown

formation and can be initiated by nutritional stress during the weaning phase, were not observed in the skeletal series, implying that infant malnutrition probably did not play a decisive factor at Volders.

In the past, malnutrition and a range of infectious diseases like bubonic plague, typhus, tuberculosis, smallpox, typhoid, cholera, scarlet fever, malaria, dysentery and further forms of parasitization imposed a substantial burden on the physical and mental health of cultures throughout the world. These factors also exerted a heavy influence upon the birthrate and mortality of populations.

An effective method towards attaining a better understanding of palaeodemography is through comparison with modern demographic situations. This is a major motivation for organizing projects like the Global History of Health, into which the Volders skeletal series data will be incorporated. A glance at today's international health statistics with regard to infectious disease and malnourishment worldwide quickly help make clear just what kind of impact poor health and living conditions can have on a population.

10.2 Tooth Cementum Annulation

In a comparison with the results accumulated by the morphological age at death analysis, the TCA method, as applied to the Volders archaeological remains, proved to be a useful tool in determining age at death. The results tend to support assertions made by (Kagerer & Grupe 2001a, Wittwer-Backofen et. al. 2004). Incremental line counts provided valuable support, especially in situations where the bone preservation left much to be desired. This method was an important indicator not only for age at death analysis, but also for identifying traumatic events such as bone fractures, events that are visible in the anomalous histological morphology of the cement rings and could be detected in a comparison with known traumata diagnosed on the skeleton acquired during the osteological examination (Kagerer and Grupe 2001b). The morphological method utilized for an age at death estimation still remained necessary for several reasons. Tooth destruction through microbial activity was not uncommon and rendered the affected tooth samples useless for histological analysis. Gross observation during tooth removal yielded no identifiable indicators for the condition of tooth preservation. Only after the tooth had been embedded, sectioned and adhered to a cover slip, was its viability revealed.

Lines were counted manually from the tooth section image on the computer monitor, a print out or directly through the microscope objective. The manual counting of lines proved to be time consuming and required a great deal of precision and patience. Also, it was established

that manual counts were sometimes subject to human error and potentially unreliable, especially during the learning phase, however, experience with the procedure increased reliability dramatically. The computer automated counting procedure was much less time consuming, yet, also showed some weaknesses with respect to counting reliability. This was best illustrated by the experiment involving juvenile individuals of approximate known age which was conducted within the Volders examination to determine the feasibility of the manual counting of incremental lines as compared with the counting ability of the computer program. The superiority of the manual counting procedure quickly became evident, as the automated procedure was shown to overestimate ages in very young individuals. This trend for overestimation was also seen in the elderly. There were also instances where the computer program was unable to provide a count due to inadequate image quality. It was therefore decided that the manual counting procedure results would be used.

Today, highly developed computers in a myriad of areas have the capacity to complete tasks once done by hand and in the process eliminate human subjectivity, error and tedium and there seems to be no end in sight to what the computer is capable of. It is certainly only a matter of time before a simple automated method replaces man as the tool in this procedure.

The manual procedure did, however, end up producing the more reliable results, and were often comparable to the morphologically won age at death estimations. As a result of the various situations described throughout the sections devoted to TCA, it becomes clear, that the accuracy of this method for establishing an age at death estimation at this present time, has its foundation not in the perfecting and use of just one procedure (i.e. automated or manual), but in both TCA methods as well as the morphological examination results being utilized in conjunction with one another and so functioning as backups for the foibles each contains.

One of the most valuable assets of this method is the ability to make a relatively reliable assumption about the age at death in the event that bone preservation is poor and insufficient for a morphological estimation. It can contribute valuable information in such situations where previously only a question mark appeared in the anthropological catalogue. The possibility of establishing a chronological age at death quantitatively instead of an age based on biological indicators as seen on the skeleton is an exciting prospect. Although TCA research efforts focused on TCA are striving to develop a sound quantitative method for providing chronological age estimations, yet, the method still remains inextricably bound to biology, relying on a once living tissue for its calculation, and is therefore susceptible to the whims and woes of an organism’s physiology. Awareness of diagenetic effects, mechanical

influences, pathologies, as well as the method's present weaknesses remains at the moment, paramount when evaluating the age estimations attained. Recently, Renz and Radlanski (2006) indicated that in line counts taken from different sides, i.e. mesial, distal, buccal and lingual, of the same tooth root section, the presence of cementocytes in AEFC, poor line visibility as well as observer error can all result in line count total variability. Although it should be noted, that the images provided by them to support these assertions, which depicted irregularly formed cementum or barely distinguishable lines, would have been excluded from analysis here. Further variables in need of attention are the possible existence of physiologically driven age-related cementum changes that might bias estimates. It is therefore necessary to conduct more known-age studies in an effort to elucidate the effects of aging on cementum histomorphology. In addition, the effects of masticatory loading on cementum production need to be better elucidated (Lippitsch, in prep.). Since one basic function of cementum is to help anchor the tooth in the alveolar socket, it might be inferred that differences in the degree of mechanical stress imposed upon the dental apparatus could result in a variable buildup in incremental layers or in the amount of cementum deposited in each layer. Specifically, it must be clarified whether discernable differences exist between people chewing different kinds of food (e.g. nuts and beef jerky vs. apple sauce and white bread) or between those using their jaws as a clamp for holding objects and as a tool for tearing or stripping fibers with those who do not, which could perhaps be clarified by investigating the cementum layer of individuals displaying temporal-mandibular joint changes.

One definitive and unavoidable drawback to the TCA method is that bioarchaeological material, albeit a relatively small amount, must be sacrificed in order to perform the examination. The decision to physically tap valuable ancient finds or fossil remains should be carefully considered, and some researchers have voiced their concern over the irrevocable loss of material. The question arises if this method should be employed at all in certain instances and if we can't live with the morphologically based estimates in these special situations until the method is perfected or another better one replaces it. Other procedures such as radiocarbon dating, isotope and trace element analysis, and histomorphological examinations also require the removal of material, and here too efforts are in progress to decrease sample size and reduce the damage incurred.

Aspects of life history seen in incremental lines

Another significant prospect, in addition to providing a true quantitative method for calculating age at death, is the potential of identifying traumatic experiences such as bone fractures based upon the incremental line form itself. Researchers have indicated that

fractures, pregnancies or other physiologically taxing experiences will have a direct influence upon cementum formation. In a study conducted using dental patients whose life histories were well documented, Kagerer and Grupe (2001b) found that in cases where an individual had suffered a traumatic injury or gone through other physiologically taxing circumstances such as a pregnancy, that the cementum layer formation during this period was affected and resulted in a visible alteration of the line as seen histologically. The lighter colored complement to the dark line appears broader, owing perhaps to an extended formation interval due to a reduction in available calcium, phosphate or other minerals required for normal cementogenesis, because they were being used as building blocks to repair damaged or develop new tissues elsewhere in the body (see Fig.10.1).

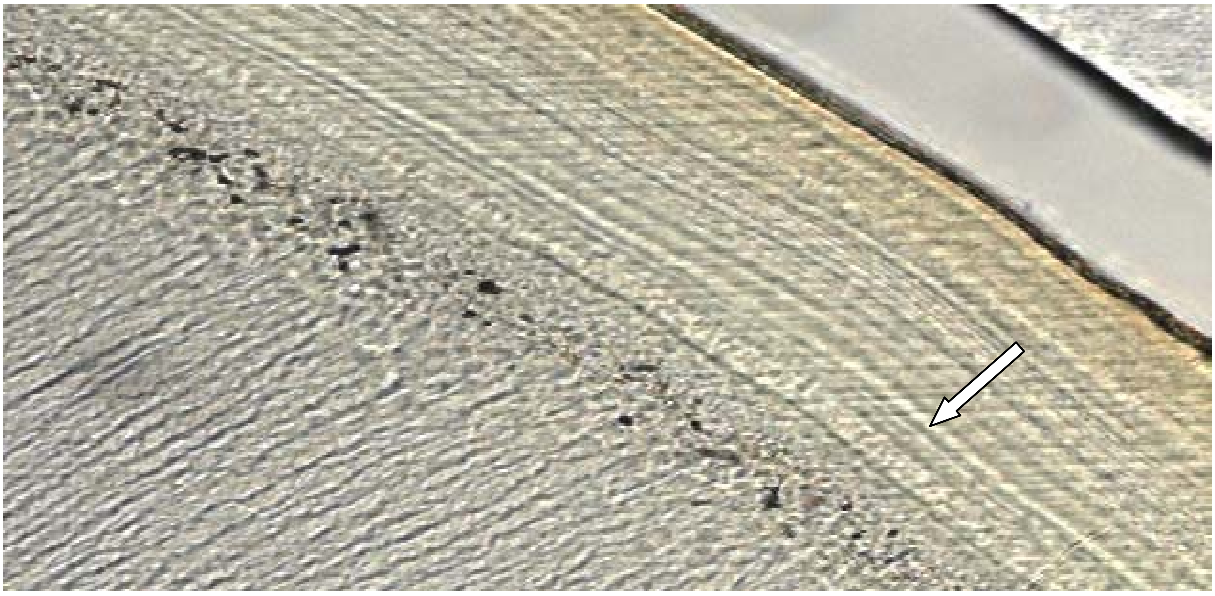

Figure 10.1. Burial 56, adult female. Several broad, light incremental lines (arrow) can be observed at various points in the section.

A mere three bone fractures were diagnosed amongst the Volders group, a relatively low rate of bone injury for a rural, working medieval population. Burials 61, an adult male who suffered a severe tibial fracture with subsequent anatomical dislocation, and 101, an adult female with a healed fracture of the ulna, both showed abnormally broad light lines at the fifth and sixth, and for burial 101, seventh line from the eruption line, perhaps indicating that the fractures occurred around the ages of 17 and 19 respectively. Images taken from other individuals, males and females alike, also showed similarly broad white lines, however, no causal agent could be identified during the skeletal analysis. Burial 24 contained an adult

male who had suffered a fractured clavicle, yet in contrast to the previous two individuals, exhibited no markedly altered incremental lines. It can be conjectured, however, that should broadened, light colored lines in fact exist, that they perhaps can be interpreted as indicators for the occurrence of traumatic or physiologically trying experiences.

The automated counting procedure software program is a good start towards developing a more reliable and efficacious counting procedure while at the same time alleviating the strain and tedium involved during manual counts. The guarantee of accuracy, however, with respect to the computers ability in deciphering true lines from image anomalies, remains somewhat unclear and certainly represents the aspect of this procedure where refinement is necessary. It is also apparent that the state of tooth root preservation and the image clarity are of no trivial importance with respect to the accuracy of age computations. The use of the maximum line count established by the automated procedure does not appear to be a feasible variable for calculating age at death estimates. The assertion made by Czermak et al. (2006), that the “mode” or line count calculated most often should be used in the estimation of ages, is certainly correct.

The desire to categorize age is quite ancient. The Greek philosopher Aristotle wrote in De Anima II that age can be categorized in three main phases, nutrition, growth and demise. It is a wonderful aphorism, and in retrospect, principally correct and descriptive of the basic physiological and anatomical metamorphosis each person goes through from birth to death, however, for the purposes of today’s bioarchaeological analyses, it is lacking in the requisite conciseness. Age estimations based upon morphological examinations are susceptible to inaccuracies resulting from observer subjectivity, differences in the amount of wear and tear features that create biologically older or younger “looking” skeletons, lack of necessary bone characteristics used in ageing (e.g. cranial sutures, pubic symphysis) and a significant amount of variability in those features. Current attempts focused on improving the accuracy of age determinations in skeletal remains are in full swing, and the quest to develop new methods or refine existing ones like TCA moves on.

10.3 Faunal analysis

Acquiring diet based isotopic indices is helpful in clarifying certain aspects of subsistence practices, however, placing these values within the framework of an environmental context is vital to understanding them. Foodweb reconstruction is based upon measuring stable isotope ratios of botanical and faunal remains as well as the human subjects that lived amongst them. It is therefore advantageous to include a diversity of animals from the various trophic levels

including domestic and feral species in an effort to generate a broader and more comprehensive picture. In the archaeological setting this is often problematic because the availability of animal bones varies. Contrary to settlement sites contemporaneous faunal remains are a lucky find in a cemetery situation like Volders. At other archaeological digs focusing on profane sites where these remains are plentiful, the bad habit of discarding animal bones with the opinion that they are useless for future study still persists. It was unexpected, yet, fortuitous that faunal remains were discovered directly at the Volders burial site and also that, following initial efforts by various inexperienced members of the dig crew to meticulously remove and dispose of them, they were carefully collected for later study. This provided the foundation from which to facilitate a reliable comparative analysis for the Volders human skeletal material, for both the collagen and carbonate analyses.

True herbivores like deer, cattle or horses are helpful in establishing the trophic level base from which other members of the ecosystem such as omnivores (e.g. man, pigs, bears etc.) or true carnivores (wolves) can be compared to. Species from aquatic environments such as fish, crustaceans, and marine plants also comprise a necessary element to the picture, however, these were not available for this study. Animals such as domestic goats and dogs living contiguous to man are usually opportunistic omnivores that potentially eat human refuse and cannot be used to establish baseline values for carnivores and herbivores respectively. Early on, researchers such as Matson and Chisholm (1991) recognized the potential of this method, and examined the faunal and floral samples of potential food items from archaeological sites, for example, the American Southwest (Cedar Mesa, Utah) in order to provide baseline $\delta^{13}C$ values for maize (-9.9 ‰), pine nuts (-23.8 ‰) and rice grass (-17.0 ‰). Grupe et al. (2003) reconstructed vertebrate foodwebs at the Iron Gates site Vlasac and the Pestenacker site in southern Bavaria to better understand Mesolithic and Neolithic subsistence strategies respectively (Fig. 10.2). This facilitates the trophic positioning of these humans within the framework of their environment and gives precise clues to what types of foods were hunted, gathered and consumed. Price et al. (2002) also advocates the incorporation of small animal samples for comparative purposes when studying isotope ratios.

The faunal remains at Volders contain a diversity of domestic animals including horses, cattle, swine, goats, sheep and chickens, and are assumed to be contemporaneous with the human remains because of their stratification. These animals are thought to have played an integral role in the diet of Volders' past inhabitants. Hunting was obviously included as a subsistence strategy since red deer bones were also discovered during the excavation, yet, the

remains of wild fauna were scant and this mode of subsistence is not believed to have been of significant import.

The faunal collagen data support the assumption that humans subsisted on a diet consisting primarily of C_3 herbivore products and C_3 plant foods. The distribution of faunal $\delta^{13}C$ and $\delta^{15}N$ correspond to species specific expectations, with the main cluster of human ratios exhibiting the trophic level step up characteristic between herbivores and omnivores (Fig. 10.2).

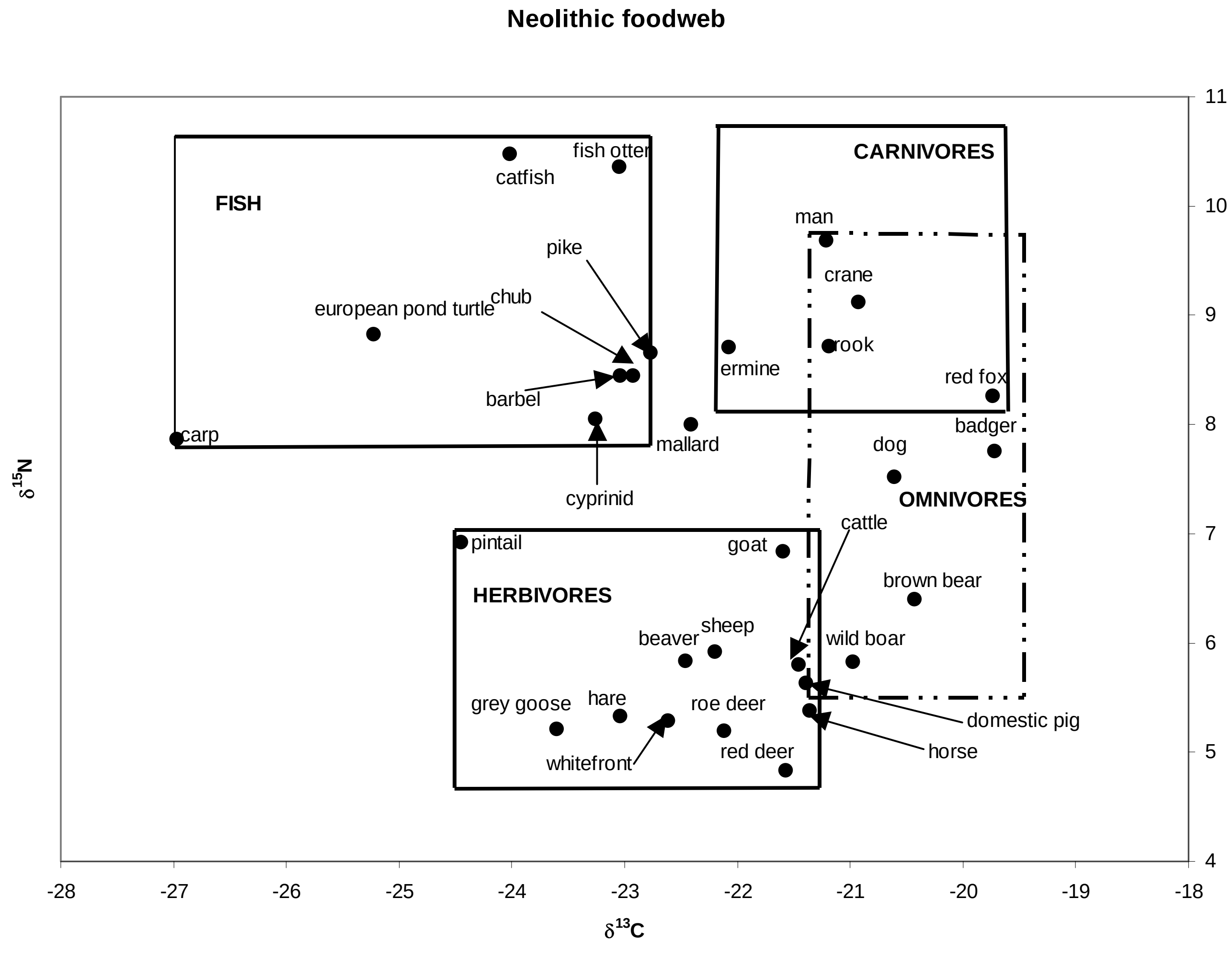

Figure 10.2. Diagram of the Neolithic foodweb as ascertained for the central European “Pestenacker” site (Bösl et al. 2006).

An examination of $\delta^{13}C$ and $\delta^{18}O$ in the bone structural carbonate showed a similar spread out pattern of depleted oxygen ratios as seen in the human samples, which afforded quantitative

support for the isotopic ratios observed in the human bone samples. Most interesting was the finding that swine showed heavily depleted oxygen ratios indicating that they were being kept at high elevation. This finding coincides with historical documentation describing the driving of pigs to high altitude alms for intensive periods of “fattening”, a practice which is being reintroduced by present day farmers (Weekend Magazin 2006). There did not appear to be any observable species specific clustering of ratios. The majority of samples, each taken from a different individual, have ratios that suggest they were living at altitudes associated with elevations between approximately 600 and 1400m. Still other animals exhibited $\delta^{18}O$ more positive than local rainfall is theoretically capable of producing including a cow (-7.22‰), chicken (-7.9‰), and a sheep (-8.16‰). This might indicate the import of these animals from lower lying, warmer areas to the south. Because nearly all of the animals tested were domestic species it is assumed that they remained in the vicinity of people. This assumption is substantiated by the scatter diagram Figure 9.27, which shows animal values not separate but in and amongst the values depicting the human population. The expectation that animals normally driven to the alms like sheep and cows would display significantly more depleted levels of $\delta^{18}O$ did not materialize. The question arises whether the 4-5 month period spent at higher elevations is sufficient to produce a noticeable change in carbonate based stable isotope ratios. Because these months are during late spring, summer and early fall, they represent the season of most rapid growth. This period of time is sufficient to facilitate the incorporation of signatures into bone mineral. However, further study must be conducted in order to assess the exact duration of time necessary for a specific signature to develop as well as the influence bone remodeling rate variability has on defining this signature. In addition, the influence of species specific physiology appears to play a significant role in the variability observed between different species, and probably represents the area where future studies should focus their efforts (White et al. 2004).

Figure 10.3 shows the distribution of carbon and oxygen ratios for the Neolithic foodweb at the Central European Pestenacker site. This diagram underlines the importance of acquiring the ratios from diverse species, aquatic and terrestrial, including mammals, fowl, fish and birds. Here herbivores are generally seen to display the more depleted oxygen values than carnivores and omnivores. Goats, cattle and sheep are all in close range of those values found for man.

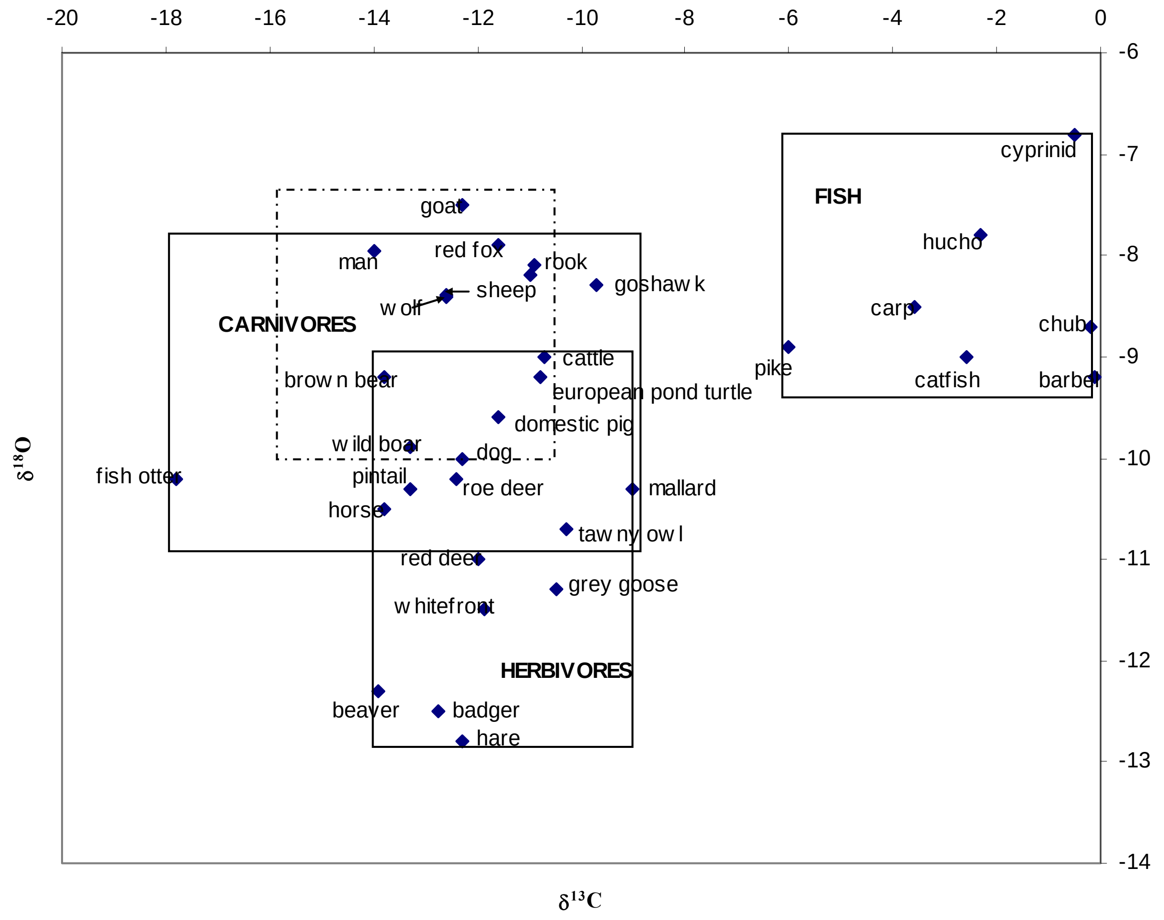

Figure 10.3. Scatter diagram of the Neolithic Pestenacker site showing carbonate oxygen and carbon values used to establish the foodweb boundaries (Bösl et al. 2006).

10.4 Palaeodietary reconstruction

Comprehensive isotopic data regarding a diversity of dietary adaptations and specializations, which provide guidelines for evaluating and interpreting palaeodiet, exist (see section 7). Emphasis on aquatic or terrestrial based diets within the context of various habitats and culturally generated situations such as status, have lead to a better understanding of isotope behavior in nature and its relationship to many organisms including man. One interest in this study is to ascertain the average diet aided by a combined examination of stable isotope ratios of carbon and nitrogen from the organic and carbon from the inorganic fraction of bone, however, special problems such as the identification of a seasonal diet in those individuals found to have been residing at higher altitudes for longer periods of time, which can be observed in mixed isotopic signatures, is also investigated.

The nutrition of an individual within a group or population can vary tremendously, and comparisons between different individuals represented in a skeletal series, especially with regards to $\delta^{15}N$ ratio differences, can yield valuable socio-economic information pertaining to

status and access to dietary proteins. For example, the relationship between status and diet was examined in precontact Highland Ecuador by Ubelaker et al. (1995) in a study which compared nutrition in high and low status people. The high-status group showed evidence for a comparatively greater consumption of maize, probably in the form of beer. Czermak and Lederrose (2004), observed marked differences in $\delta^{15}N$ ratios in skeletal samples taken from a small burial sector separated from the main cemetery, which, based upon the rich assortment of grave associations, was believed to contain the skeletons of higher status individuals. The connection between higher levels of ^{15}N and elevated status is based upon the presumption that $\delta^{15}N$ ratios fluctuate with the varied intake of animal proteins, in this case meat. Because the energy investment required for producing meat foodstuffs is costly when compared to plant foods, and because a live animal is often more desirable than a dead one simply through its continual generation of foods like dairy products or wool, meat is considered only to have been regularly eaten by social elites. There is, however, no indication of status differences at Volders and animal proteins appear to have been available to and consumed by common farmers and workers, although not all, since a considerable range was observed.

Isotopic compositions of carbon and nitrogen in organic bone collagen were determined primarily to gain information regarding the relative proportions of plant versus animal foods in the diet. According to the bone collagen ratios of $\delta^{15}N$ and $\delta^{13}C$, the majority of the Volders population fell within the expected range of 8-11‰ for $\delta^{15}N$ and 19-21‰ for $\delta^{13}C$ that is characteristic for humans subsisting primarily on indigenous C_3 plants and products acquired from C_3 herbivores. The $\delta^{13}C$ data acquired in this study are consistent with research conducted by O'Leary (1981, 1988) and DeNiro et al. (1985) who showed that C_4 plants have a higher $\delta^{13}C$ / ^{12}C ratio than C_3 plants. In addition, C_4 plants were as yet not widely cultivated in the Old World. Therefore, based on the $\delta^{13}C$, C_3 plant cultivation and animal husbandry appeared to have provided the main supply of foods. No significant differences in carbon isotope ratios could be detected between males and females in the Volders population. There was, however, one individual who exhibited a $\delta^{13}C$ ratio indicative for the periodic consumption of freshwater fish. This individual, a middle aged male and represented by burial 112, was the only outlier. Unfortunately, the sample was one of three discounted due to an imbalanced C:N ratio (5.1) indicative of contamination. The remaining data do not provide any indication for dependence on nearby rivers, lakes or streams for freshwater resource subsistence since the consumption of freshwater fish results in depleted levels of $\delta^{13}C$ not seen here (see trophic level partitioning, Figs. 7.1 and 10.2).

This present study showed that $\delta^{15}N$ ratios for both adult men and women had a mean of 10.01‰ consistent with the consumption of terrestrial foods. However, this series of isotope values is remarkable in terms of a number of $\delta^{15}N$ ratios observed which spanned over two trophic levels and indicate a significant difference in protein intake amongst certain individuals in the group. A number of individuals exhibited $\delta^{15}N$ ratios well above the group mean and some had lower than average ratios. The difference between the two groups resulted in the striking trophic level differences observed and implies substantial differences in these particular individual's respective diets. Initially, the high $\delta^{15}N$ ratios might have been interpreted as evidence that certain individuals were at least augmenting their diet with readily available freshwater food resources from the adjacent Inn River. In aquatic based diets, the inclusion of freshwater food sources results in an increase in $\delta^{15}N$ levels and more depleted $\delta^{13}C$ levels. The observation that those individuals with elevated nitrogen values were shown to have oxygen isotope compositions associated with low altitude residency tended to support this since people living in this area would have had more immediate access to the Inn River food resources than those living at higher altitudes. However, the $\delta^{13}C$ values from collagen were not highly depleted and did not coincide with those values expected for the consumption of freshwater fish species common for the area like trout, pike, carp and barbel (see Fig. 10.2). Furthermore, the $\delta^{13}C$ from carbonate did not show the heavy enrichment in $\delta^{13}C$ values characteristic for freshwater fish, and it is more likely that individuals with higher $\delta^{15}N$ values were simply consuming more meat and animal products than their counterparts. The faunal analysis, which provides a baseline isotope pattern for the Volders area, also supports this assumption. A diet augmented with cheese and milk, as would be expected from an agriculturally based group, is indicated by a mixed balance of isotopic values. Milk and dairy products are enriched isotopically relative to their producer due to physiological fractionation, which are then passed on to the consumer.

The fact that there was no significant difference in $\delta^{13}C$ or $\delta^{15}N$ ratios between men and women would tend to indicate that both were subsisting on similar diets. From this data, it can be inferred that the majority of Volders population represented a model of egalitarian agriculturalists and pastoralists. In addition, the stable isotope data, reflecting average dietary intake over the last several years of life, do not indicate the presence of chronic malnutrition.

A wide range of $\delta^{15}N$ values were observed for the subadult group. There were a number of infants with elevated nitrogen isotope values, indicating that they were still nursing at the time of their deaths, while other very young infants did not exhibit these high nitrogen levels, implying that they had already been weaned at the time of their deaths. These results are

consistent with various authors findings such as Fogel et al. (1997, 1989), Katzenberg et al. (1993) and Tuross and Fogel (1994), who found that nursing infants have enriched nitrogen values when compared to weaned infants or adults. Based upon the nitrogen levels that progressively declined with age between the second and fourth year, the trend shows that children at Volders were nursed at least until their second year, some longer. Due to their increased susceptibility to the deleterious effects of taphonomic factors, many of the subadult collagen samples were diagenetically altered and had levels of preservation that did not allow for analysis. The overall number of samples necessary for a realistic comparison between different ages and subsequently to make inferences about weaning age was therefore significantly reduced. Based upon their $\delta^{15}N$ and $\delta^{13}C$ ratios, weaned subadults also appear to have been fed diets similar to the adults and a significant difference between the two was not observable. The mean $\delta^{15}N$ for 6 subadults was 9.74‰, whereby, one of these, a 5-7 year old (Burial 141), showed a very low ratio indicating a diet based entirely upon C_3 plant foods or perhaps a metabolic defect or illness.

The relationship between body size and protein consumption as reflected by nitrogen values in adults showed a marked difference in the ratios exhibited by men and women and is depicted in Figures 10.4 and 10.5. Male individuals showed a definite increase in physical height with more elevated nitrogen levels. Females on the other hand, showed neither an increase nor reduction in height with respect to nitrogen ratios.

In 11 large males with heights at least 1 standard deviation above the mean for height, the mean nitrogen score was 10.31, well within 1 standard deviation above the mean for males.

Six small males with heights at least 1 standard deviation below the mean for height showed a mean nitrogen ratio of 9.76, well within 1 standard deviation below the mean.

In six tall females with heights 1 standard deviation above the mean, the mean nitrogen level was 10.11 well within 1 standard deviation from the mean. There were 2 small females with heights 2 standard deviations below the mean. Their mean nitrogen score was 9.75, well within 1 standard deviation below the mean.

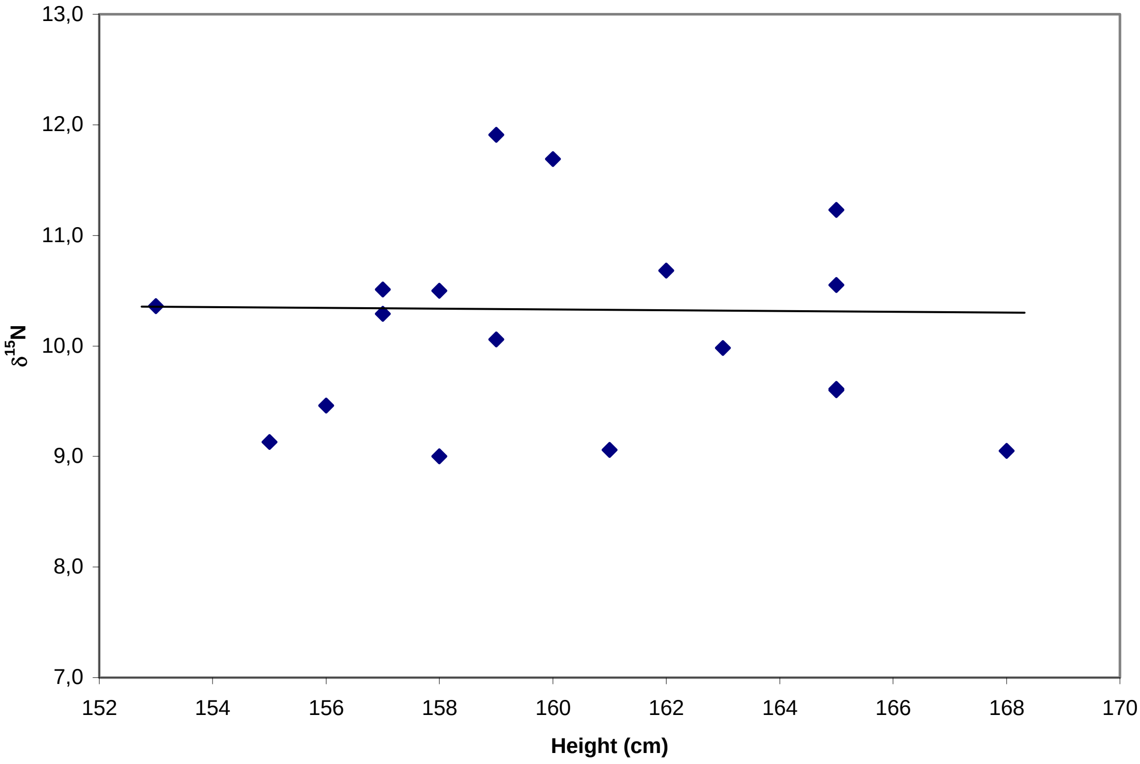

Figure 10.4. Scatter diagram comparison of adult female $\delta^{15}N$ and body height. The trend line indicates a near uniform relationship between nitrogen ratios and body height.

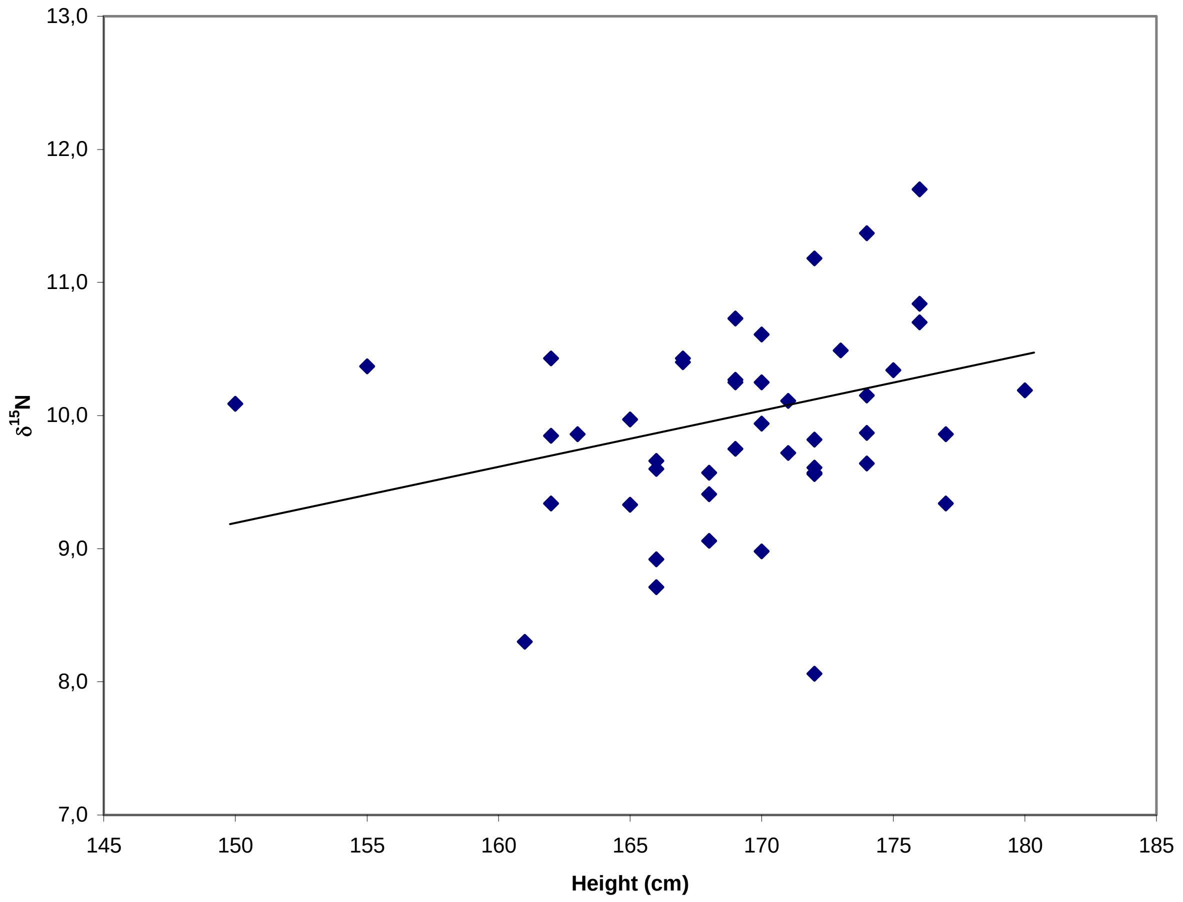

Figure 10.5. Adult male nitrogen values plotted against height. In contrast to the female group, males show a clear tendency to possess a larger body size with increasing nitrogen levels.

10.5 Identifying the utilization of high altitude sites

Many different environments have been studied using oxygen isotopes to identify area or niche specific signatures and also to gain insight to palaeoecological conditions that predominated in the past (see section 7.9). The sensitivity of ^{18}O to climatic and topographic features such as temperature and elevation make it a potential tool for investigating habitat preference, in this case, high altitude sites. The inorganic bone fraction hydroxyapatite is one origin of structural carbonate (teeth are another) and the source of carbon and oxygen isotopes analyzed in this study. The feasibility of oxygen isotopes as an accurate indicator for altitudinal changes is based primarily on the linear relationship of meteoric precipitation, which becomes isotopically lighter with increasing altitude, and in turn correlates with decreasing temperatures. Recently, Budd et al. (2004) indicated that oxygen isotopes provide an effective method for tracing population movements at a regional and international level. The intent in the Volders study is to concentrate the focus within a local area and identify

movement based on altitudinal changes, where the distances are vertical and not horizontal. Carbon and oxygen in bone carbonate undergo a continuous process of resorption and replenishment, however, they are representative of the dietary intake over at least the last several years of an individual's life (Ambrose & Norr 1993). The oxygen isotopic values collected in this study, therefore, reliably represent dietary intakes over longer periods and indicate stays at various higher levels of elevation for extended periods and not brief or periodic visits.

As illustrated in Table 19, the OIPC's calculations for the isotopic composition of precipitation are based on known variables coinciding with the geographical location of Volders (557m) and its associated mountain elevations (max. 3000m). These variables are latitude 47°.3' and longitude 11°.8', with suggested altitudes in 100m increments. A total of 128 individuals were analyzed for the oxygen isotope compositions of bone apatite structural carbonate, which produced a wide range of values (see Table 20).

Table 19. OIPC $\delta^{18}O$ and δD ratio estimates for the Volders coordinates combined with 100m elevation increments. These calculations provide the basis with which to compare the actual bone $\delta^{18}O$ ratios acquired from the Volders skeletal material. Latitude and longitude for sea level and slightly higher elevation (0m and 100m respectively) are indicated separately.

Altitude (m)	$\delta^{18}O$ (V-SMOW)	δD (V-SMOW)	Lat. / Long.	Altitude (m)	$\delta^{18}O$ (V-SMOW)	δD (VSMOW)	Lat. / Long.
0	-6.93	-44.77	45°.5';12°.5'	1700	-10.82	-74.19	47°.3';11°.8'
100	-7.04	-45.41	45°.3';11°.8'	1800	-11.01	-75.62	
200	-7.24	-46.83		1900	-11.2	-77.04	
300	-7.43	-48.25		2000	-11.4	-78.46	
400	-7.62	-49.68		2100	-11.59	-79.88	
500	-8.55	-57.43	47°.3';11°.8'	2200	-11.78	-81.31	
600	-8.69	-58.55		2300	-11.98	-82.73	
700	-8.89	-59.97		2400	-12.17	-84.15	
800	-9.08	-61.39		2500	-12.36	-85.57	
900	-9.27	-62.82		2600	-12.55	-87	
1000	-9.47	-64.24		2700	-12.75	-88.42	
1100	-9.66	-65.66		2800	-12.94	-89.84	
1200	-9.85	-67.08		2900	-13.13	-91.26	
1300	-10.05	-68.51		3000	-13.33	-92.69	
1400	-10.24	-69.93		3500	-14.29	-99.8	
1500	-10.43	-71.35		4000	-15.26	-106.91	
1600	-10.62	-72.77					

Table 20. Altitude estimations based upon Volders $\delta^{18}O$ bone carbonate ratios for all individuals using OIPC standards and calculations.

$\delta^{18}O$	est. Alt.	$\delta^{18}O$	est. Alt.	$\delta^{18}O$	est. Alt.	$\delta^{18}O$	est. Alt.
-6.19	0	-8.52	498	-9.37	950	-10.53	1553
-6.42	0	-8.54	500	-9.4	990	-10.6	1600
-6.71	0	-8.54	500	-9.48	1010	-10.64	1590
-7.33	245	-8.58	520	-9.48	1010	-10.70	1645
-7.39	250	-8.58	520	-9.49	1010	-10.70	1645
-7.57	380	-8.59	525	-9.49	1010	-10.71	1650
-7.6	398	-8.64	575	-9.56	1040	-11.19	1890
-7.65	410	-8.65	580	-9.59	1050	-11.2	1900
-7.75	430	-8.68	600	-9.6	1080	-11.32	1950
-7.83	435	-8.73	550	-9.62	1090	-11.69	2150
-7.9	440	-8.77	630	-9.63	1098	-11.81	2220
-7.93	445	-8.77	630	-9.68	1120	-11.89	2275
-8.03	449	-8.84	690	-9.73	1150	-11.94	2290
-8.05	450	-8.87	695	-9.74	1140	-12.07	2350
-8.12	455	-8.89	700	-9.82	1190	-12.19	2420
-8.17	460	-8.96	720	-9.84	1200	-12.33	2450
-8.18	460	-9.02	798	-9.85	1210	-12.40	2520
-8.21	460	-9.02	790	-9.88	1220	-12.43	2550
-8.21	460	-9.02	790	-9.91	1230	-12.83	2780
-8.21	460	-9.05	800	-9.96	1280	-12.91	2790
-8.25	465	-9.09	802	-10	1290	-13.08	2890
-8.3	470	-9.14	820	-10.01	1295	-13.53	3100
-8.31	475	-9.17	845	-10.10	1315	-13.59	3250
-8.32	475	-9.18	850	-10.11	1320	-13.75	3300
-8.33	480	-9.19	860	-10.14	1335	-14.20	3450
-8.33	480	-9.20	865	-10.19	1350	-14.34	3500
-8.33	480	-9.21	870	-10.19	1350	-14.77	3750
-8.35	480	-9.22	870	-10.21	1360	-15.16	3950
-8.35	480	-9.24	880	-10.28	1375	-15.26	4000
-8.45	485	-9.26	895	-10.32	1450	-15.31	4025
-8.49	490	-9.27	900	-10.35	1450	-15.45	4100
-8.50	495	-9.34	950	-10.38	1490	-16.03	4500

Statistically, the data indicate that no significant differences in $\delta^{18}O$ ratios exist between adult males and females, although, based upon the OIPC calculations, there were a number of $\delta^{18}O$ values that exceeded the predicted altitudes at both ends of the spectrum for those found in the Volders geographical study area. According to one of the OIPC's originators, there is a certain amount of error associated with the resulting calculations since they are interpolated estimations and not actual physical measurements (G. Bowen, personal communication). Based upon the suggested OIPC data and the actual bone $\delta^{18}O$ ratios, it quickly becomes apparent that many isotopic compositions are more negative than predicted for the area. The differences observed between heavily depleted and less depleted ratios, as listed in Table 20, may be attributed to fractionation effects associated with water transport down and through

the mountain. Water ending up at the valley floor catchments in an alpine area is actually a mixture of snowmelt, precipitation falling as rain, and sub-surface water trapped within the mountain geology that is forced out during the melt. Meteoric water, as it travels down the steep incline of the mountain, becomes heavier as a result of a variety of kinetic variables such as evaporation, temperature changes, and rapid mixing with various source waters (e.g. sub-surface-, melt-, lake- and river water), all of which are linked to the local topography. People living at lower levels were therefore drinking isotopically heavier water created by this process. Those residing at higher elevations drank water that still retained most of its original signature since it is less subjected to the alteration processes resulting in isotopically heavier drinking water. The wide dispersion of values indicates that many of the Volders individuals were acquiring their drinking water from a variety of different sources and not one central basin. This is no doubt the main factor that accounts for the remarkable differences found in the isotopic composition of $\delta^{18}O$ in the Volders bone samples and clearly implies altitudinal differences in terms of occupation. The only plausible physiological differences that could result in such a large variability might be related to the increased necessity of those living at higher altitudes, who are exposed to altitudinal effects like drier air and colder temperatures and also engaged in strenuous work, to drink greater volumes of water. A combination of this with the availability of isotopically lighter water rushing down from the highest and coldest peaks would definitely result in very low $\delta^{18}O$ ratios in bone carbonate. The work, however, involved in tending herds is quite minimal and, with the exception of ascents, shepherds actually remain sedentary for long periods thereby conserving energy.

The highly depleted ratios observed in a large portion of the individuals from Volders, may suggest that they were spending significantly longer periods of time at elevation than their herd animals, which interestingly exhibited less depleted ratios. It is also plausible that the source water was different. People residing in the mountains might have preferred fast rushing mountain streams for their water consumption, which would be carrying water significantly more depleted in ^{18}O than the ponds or catchments containing a more mixed and, therefore, more enriched in ^{18}O water, from which their animals were drinking. It is also possible that these individuals were spending the entire year at elevation, providing for their animals in the spring and summer months, then engaging in other activities such as mining, which would have taken them to even higher altitudes, in the fall and winter months.

Of these individuals with highly depleted values (-11.56 or more negative) 3 were females and 8 were males. However, it must be taken into consideration that the sample size for males composed 69% of the total of 118 adults. It is therefore probable, that both men and women

resided or worked at high altitudes. This might also suggest that not just individual workers, but families lived at these higher elevations throughout the year, a finding that gives credence to the theory that these people were probably mountain farmers and pastoralists.

Figure 9.18 shows a clustering of approximately 30 individuals whose isotope ratios indicate that they were residing between 550 and 700 meters. Approximately 20 people who had $\delta^{18}O$ values correlating with altitudes of 700 to 1400 meters most likely resided at those elevations and did not travel to and fro to the valley floor since regular accents back to these elevations from the valley floor would certainly have been too exhausting and time consuming. Those individuals with substantially depleted values $\delta^{18}O$ represent those whose subsistence strategy or occupation required them to work and live at higher altitudes where they were engaged in a variety of functions such as shepherding, grass harvesting and perhaps mining. As mentioned previously, there is also the possibility that the dehydrating effects of strenuous labor involved in specific occupations such as mining, forced these particular individuals to drink larger quantities of water. The greater the need to consume water the more negative the $\delta^{18}O$ value becomes (Bryant & Froelich 1995, Kohn 1996, Kohn et al. 1996, Kohn 1999).

The seasonal availability of water for human consumption within the confines of the mountain environment appears straightforward. If the presumption is true that early medieval peasant populations acquired their drinking water from surface sources, then the water they drank in winter derives from winter precipitation and the water they drank in the summer months was comprised of both winter melt- and summer water. This translates into winter water being depleted in ^{18}O and summer water showing a mixed, more enriched signature. Those living at higher altitude would have continual access to winter-type ^{18}O depleted water since the spring and summer months at high elevation are characterized by a copious supply of water from snowmelt.

The relatively large number of individuals exhibiting $\delta^{18}O$ ratios characteristic for lower lying, warmer areas to the south and north (according to OIPC estimates) seems to imply that the Volders population was very heterogeneous and perhaps multi-cultural. No less than 34 individuals had $\delta^{18}O$ levels lower than predicted for the lowest elevation at Volders (557m). These may be itinerate members of the group indicating a pattern of recent migration from lower elevations associated with the lowlands to the south (see Fig. 7.9). Some of the ratios are positive enough to be indicative of Adriatic or Mediterranean coastal areas, and others are more characteristic for the inland foothill region just south of the Alps or to the north in Bavaria. Other authors have used this type of observation to assess migratory patterns. White et al. (1998) found very low levels of $\delta^{18}O$ variation in the burials she analyzed from the

Teotihuacán site Tlajinga 33 in the Mexican Basin, which is typical for a stationary population consuming the same source water, in this case a ground water source that is subject to little seasonal variation. The large variation of $\delta^{18}O$ values recorded for the Volders series, which would be interpreted as signifying migration at another topographically more monotonous location, is taken here to indicate not only occupation of the mountainous habitat at different altitudes (vertical differences), but also possible population movement from other areas (horizontal differences).

The agriculturally unfortuitous wetland situation in the Inn Valley during this medieval time period acted as an incentive for many of Volders inhabitants to more intensively utilize higher elevations for subsistence and in essence their survival. This accounts for the majority of the population evidencing depleted $\delta^{18}O$ ratios indicative of elevations higher than that of Volders. The stable isotope variations of oxygen in water exhibited in the Volders area are directly related to the effects of the significant differences in altitude and temperature that characterize the region. The drastic changes in elevation are also within a relatively small radius of the village, which makes this type of comparative analysis possible. Transhumance systems are still practiced in various regions of the world, especially Europe and it can be readily observed in countries such as Portugal, Spain, Italy, Russia, Armenia, France, Poland, Czechoslovakia and Rumania.

Since oxygen isotopic compositions are the result of ingested water and food, and the consumption of large amounts of milk by pastoralists is well known, it is plausible that animals grazing and drinking at high altitudes might have milk depleted in ^{18}O. Animal milk is more enriched in ^{18}O than water, however, the exact isotope composition for sheep, goat and cow milk for this area remains to be analyzed in a future study.

The effects of elevation based upon $\delta^{18}O$ on body size was also investigated. Figures 10.6 and 10.7 both indicated that body size actually increased the higher up the mountain these individuals lived. This finding clearly negates the existence of hypoxic living conditions, which exert a negative influence upon growth and development (Little et al. 1971). It also suggests that those individuals that were living at higher elevations not only had access to sufficient nutrition but were also physically active, both factors that stimulate both growth. The most important conclusion out of the data presented on health, nutrition, life expectancy and body size is that these people were perfectly adapted to this alpine environment.

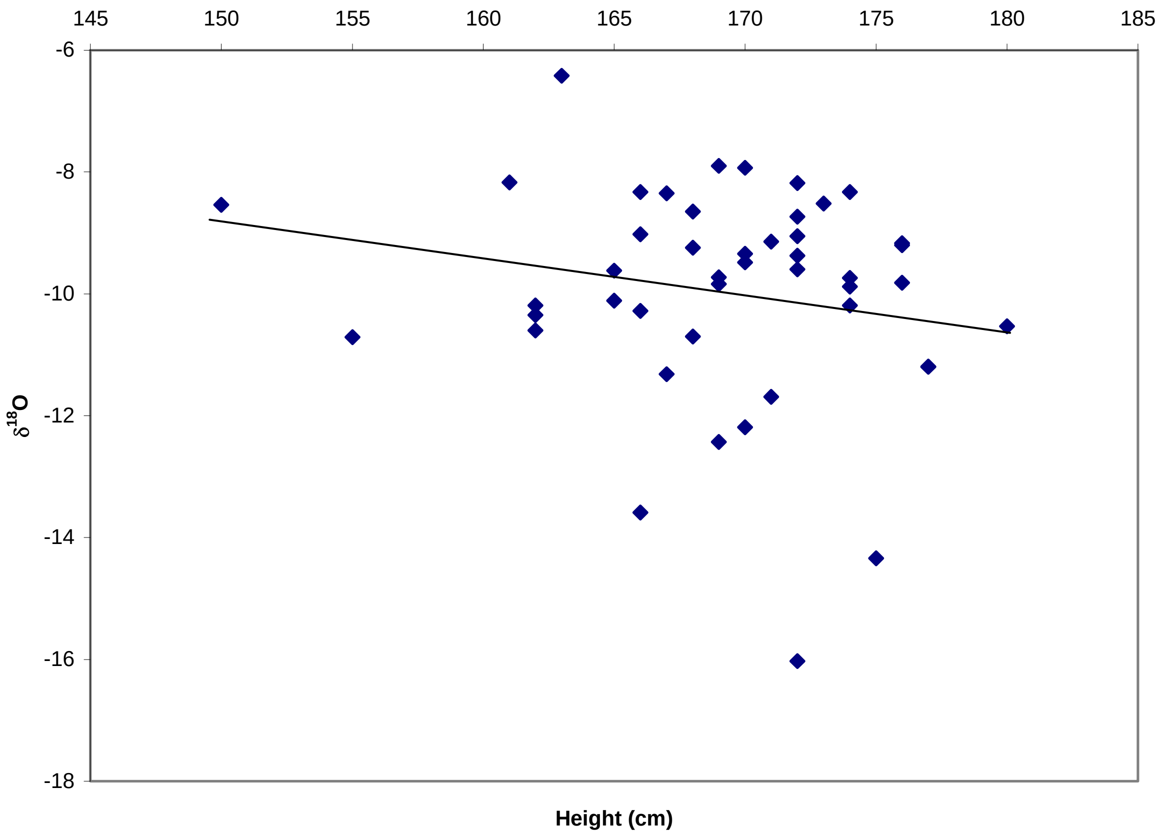

Figure 10.6. The scatter diagram of male individuals at Volders shows a clear trend for increased height with increasing elevation of habitation.

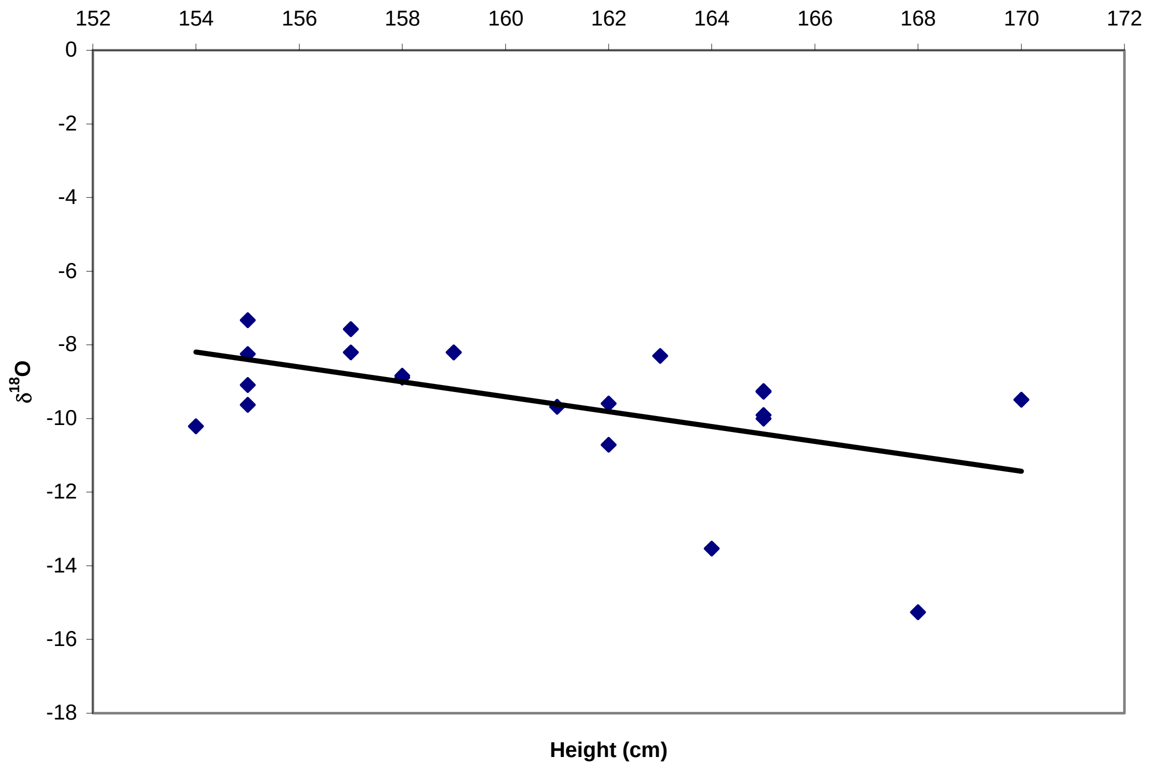

Figure 10.7. Female individuals at Volders exhibited a similar trend to the males and showed a trend towards increased body height with increasing altitude.

Summary

This present study of a 5-7th century Austrian burial site, featuring palaeodemography, dietary reconstruction, subsistence and transhumance activity, is important in furthering our understanding of phenomena involving human adaptability and cultural change in the alpine region and elsewhere in the early and modern European world. The comprehensive breakdown of the Volders alpine population will add a further cog in the information wheel by interpreting observable differences between individuals residing within a heterogeneous group and utilizing different parts of a unique, yet, similar environment.

Based upon the osteological, histological and isotopic data, the individuals represented at the Volders site were well nourished, suffered few traumatic injuries, showed little evidence of disease, and lived relatively long lives. The findings indicate a high level of fitness and show that the Volders population was superbly adapted to the alpine environment. Dietary evidence indicates a tremendous amount of variability with respect to nitrogen levels, clearly indicating substantial differences in the amounts of proteins consumed. Adult nitrogen isotope variability spans over two trophic levels and range between 7.78‰ and 11.91‰. There is, however, no specific group that seems to be separate from the rest, which would otherwise

suggest strict social stratification with a segment of the population enjoying a higher level of status than the majority. As expected, collagen carbon isotope values showed a distribution typical for the reliance upon C_3 plant consumption and showed little variability, ranging between -18.75‰ and -21.47‰, with the vast majority well within one trophic level. In addition, none of the isotopic data from bone collagen or carbonate indicate the regular consumption of fish from nearby rivers or lakes.

Infants also exhibited great variability in their nitrogen ratios, which provided information regarding nursing and weaning behavior. Higher levels in infants who are expected to be nursing substantiate assumptions upon which this determination method is based. Although the subadult group was limited in number, nitrogen isotope and carbon spacing results both suggest that infants were nursed at least until the second year. Based upon the carbon spacing differences observed, results from this calculation were shown to be an effective method for detecting nursing and weaning.

Oxygen isotope ratios displayed a very high degree of variability within the Volders group, indicating not only significant altitudinal differences with respect to residence within the confines of the mountain ecosystem, but also differences suggestive of migration from lower lying, warmer regions to the Volders area. Some of the people with extraordinarily depleted levels of $\delta^{18}O$ (less than -11.5‰) must have resided and worked for extended periods of time at very high elevations and drank water severely depleted in $\delta^{18}O$, probably from high mountain snowmelt runoff. These individuals certainly represent mountain farmers engaged in transhumance pastoral activity. There is also the possibility that some people were working ore- or quartz mines, however, at the moment there is no empirical way to determine this. Others who may have resided in the valley but traveled frequently to higher elevations would exhibit a mixed $\delta^{18}O$ isotopic composition, and would be expected to exhibit ratios of approximately -9‰. The oxygen isotope results seem to indicate a general distribution along the incline of the mountain, with a smaller group of individuals utilizing the higher elevations. This distribution can be seen today and suggests that the successful adaptive responses and strategies of Volders' ancestral Raetish mountain farmers were passed down through the millennia and continue to characterize the countenance of this alpine region even today.

The analysis of human osseous remains plays an integral role in the quest for achieving a clearer understanding of past human ecology, cultural evolution, subsistence practices and social organization. The combination of osteological and archaeometric methods allows for a broader retrieval of information locked in the bones of archaeological skeletons. In this study routine osteological and histological examinations provided basic data necessary to build the

demographic foundation for the Volders skeletal series. Amongst the ever growing arsenal of methods employed in archaeometry, isotopic analysis has proven a stalwart instrument for gathering information in archaeological settings. The stable isotopes of carbon and nitrogen in human bone collagen reflect the chemistry of the diet and therefore, provide a direct qualitative measure of foods consumed. The use of stable isotopes of oxygen, traditionally employed to unlock the doors to palaeoclimate, is emerging as a new and accurate instrument for determining location of physical habitation and determining migration patterns by tracking isotopic signatures unique to specific areas. Even these methods will soon become traditional and eventually, perhaps some time in the near future, they will be supplanted by other novel palaeogeochemical and histological laboratory techniques.

The fields of forensic anthropology and bioarchaeology share a pedestal of fundamental knowledge essential to achieving each one's individual goals, irregardless of how different these may be. The similarity is apparent is the words of forensic anthropologist William Haglund, who stated that the main objective of his work is to "…give the victim a face" (GfA conf., 2005). The "face" in this case is used in the metaphorical sense to imply the physical attributes, personal history, habits and other features or qualities of a deceased person, and first begins to take shape as various informations from the anthropological examination are collected. Each bit of information gathered is a further brush stroke that adds more color and clarity to the forgotten portrait, and although the paths of intention of these two distict, yet, closely related fields only seldom cross, for the bioarchaeologist, giving the past and the people who lived in it a face helps to fill the gaps in history and brings us a step closer to understanding our roots and perhaps even ourselves.

Appendix 1

Volders Anthropological Catalog

Burial	Condition	Sex	Age (yrs)	Height (cm)	Pathologies and Comments
1	poor	tm	adult	nd	Extended position, orientation east-west with head at the west end. Burial was to a large extent destroyed by backhoe. The sex determination is based upon the robust structure of the skeletal elements present.
2	average	tf	>60	157.3	Extended position, orientation east-west with head at the west end. Distal phalanges of toes all have exostotic growths. Man-TCA age est.: 90 yrs., Auto-TCA age est.: 99 yrs.
3	poor	m	>60	169.2	Extended position, orientation east-west with head at the west end. Partially damaged by backhoe. Right femoral head exhibits lesion on inferior surface. Proximal right radius and the left ulna near olecranon show degenerative changes similar to a necrosis. Deformation of a metacarpal due to hand injury. Exostotic growth on both distal humeri. Osteophytes on all five LV, left acetabulum show signs of advanced arthritic defect, diverse lesions, Cranium not present
4	poor	tm	adult	nd	Extended position, orientation east-west with head at the west end. Skeleton almost completely destroyed by backhoe. The sex determination is based upon the robust structure of the skeletal elements present.
5	poor	nd	adult	162.6	Extended position, orientation east-west with head at the west end. Bone displays mushy consistency. Traumatic injury to left tibia in the form of a puncture wound with subsequent infection. Bone callus on the right ulna.
6	poor	tm	51-55	nd	Extended position, orientation east-west with head at the west end. Robust bone structure, slight lipping on right acetabulum, pronounced muscle attachments on both humeri. Heavy osteophyte growth on one LV and Schmorl’s nodes. Advanced periodontal disease and very heavy tooth wear on all teeth. Morphological age estimate 50-60 yrs. Man-TCA age est.: 54 yrs., Auto-TCA age est.: 60.
7	poor	m	adult	172.1	Extended position, orientation east-west with head at the west end. Backhoe removed cranium and CV 1-3. BV 1-5 show slight osteophyte formation, Schmorl’s nodes on BV 8-12, osteophytes, medial clavicle show heavy calcification indicating that the individual is probably of middle to late adult age.
8	poor	tm	adult	170.4	Extended position, orientation east-west with head at the west end. Heavy osteophyte growth on LV 5 with resulting synostosis with sacrum. Evidence for herniated vertebral disk. Bone structure is very robust.

9	poor	f	adult	nd	Extended position, orientation east-west with head at the west end. Burial partially damaged by burial 8, which lies above it. Small osteophytes on TV 1-5. A second burial labeled as "with 9" also exists. The archaeological record does not indicate if the burial is primary, secondary or part of a double burial. What is known is that it is a young infant.
10	good	m	>60	169.6	Extended position, orientation east-west with head at the west end. Pronounced muscle attachments. Variation: inscisura patella lateralis. Slight lipping on right glenoid cavity. Median crest completely open (spina bifida occulta). LV 2-4 block vertebrae (fused), LV 2 has a significant left lateral compression that resulted in scoliosis. It is uncertain if the compression resulted from a fracture. All of the right side intervertebral articular surfaces of CV 1-7 and the corpus of 3-4 indicate degenerative joint disease. Tooth wear is moderate to heavy and premolar and molar resorption is present. Morphological age thought to be approx. 60-70. Man-TCA age est.: 72 yrs., Auto-TCA age est.: 72.
11	poor	f	46-50	159.3	Extended position, orientation east-west with head at the west end. Burial borders disturbed by burial 10 and 18. Delicate bone structure, pitting on the pubic surface visible (potential indicator for parturition. LV corpus height compromised and arthritic. Moderate tooth wear, tooth resorption and dental caries. Morphological age estimate 50-60 yrs. Man-TCA age est.: 47 yrs., Auto-TCA age est.: 27.
12	average	nd	ca. 1.5-2	nd	Extended position, orientation east-west with head at the west end. Lower extremities pressed down by stone wall. Age estimation based on bone development and long bone lengths.
13	average	tm	adult	167.3	Extended position, orientation east-west with head at the west end. Wood rests indicate body was placed on a board. Muscle attachments pronounced. Lipping on distal right radius. Pronounced osteophyte formation on LV 3-5. Heavy tooth wear on all teeth, dental abscess in lower right quadrant labial to canine, periodontal disease in same area, and dental caries with complete destruction of crown in m3.
14	poor	f	>60	157.9	Extended position, orientation north-south with head at the north end. Very weathered bone material, most of the analysis conducted in situ. Calcified cricoid cartilage. Morphological age thought to be approx. 60-65. Man-TCA age est.: 85 yrs., Auto-TCA age est.: 77.
15	average	f	31-35	155.1	Extended position, orientation north-south with head at the north end. Delicate bone structure. Variation: septal aperture of right distal humerus. Dental caries (fissure) upper right m2, resorption upper left m2, and moderate tooth wear. Both Auto- and Man-TCA age est.: 37 yrs.
16	good	m	46-50	161.4	Extended position, orientation east-west with head at the west end. Skeleton in humus layer. Robust bone structure. Spondylitis (lumbar vertebrae), osteophytes, block

					vertebrae LV 1-2, moderate to heavy tooth wear. Man-TCA age est.: 40 yrs., Auto-TCA age est.: 48.
17	poor	m	>60	nd	Extended position, orientation east-west with head at the west end. Disturbed by burial 18. Very robust bone structure, calcification of medial clavicle, and minor osteophyte formation on LV and TV 4-5. Man-TCA age est.: 79 yrs., Auto-TCA age est.: 81 yrs.
18	average	m	46-50	169.2	Extended position, orientation east-west with head at the west end. Disturbed by burials 11 and 17. Badly weathered with additional damage by backhoe, most measurements made in situ. Morphological age estimate 50-60 yrs. Man-TCA age est.: 49 yrs., Auto-TCA age est.: 58 yrs.
19	average	m	51-55	150.4	Extended position, orientation east-west with head at the west end. Bone structure is delicate for a man. Caput femoris is slightly degenerative and exhibits a bone spur (sequester). Raised area in the middle of the olecranon fossa (injury callus) reduced lower arm flexibility. TV shows osteophyte formation on 7-8 and a block vertebra between 3-4.
20	poor	nd	5-7	nd	Extended position, orientation east-west with head at the west end. Moderate damage by weathering. Age estimation according to femur length.
21	poor	nd	4-5	97	Extended position, orientation north-south with head at the north end. Heavy weathering damage.
22	average	m	20-25	160.9	Extended position, orientation east-west with head at the west end. Foot skeleton cemented into garden wall. Moderately robust bone structure. Iliac crest and medial clavicle growth plates both still visible. Arthritic degeneration of the 6-8 TV, possible compression fracture of the 8th thoracic vertebra.
23	average	m	>60	164.5	Extended position, orientation east-west with head at the west end. Skeleton in sandy gravel matrix. Weathered, gnaw marks on right humerus. Synostosis of LV1-2 with the TV 12, moderate to heavy tooth wear. Morphological age estimate 50-60 yrs. Man-TCA age est.: 67 yrs., Auto-TCA age est.: 66 yrs.
24	poor	m	>60	nd	Extended position, orientation north-south with head at the north end. Grave border ringed by stones. Backhoe damage caused loss of all elements below LV 3. Synostosis of the LV 1 with the TV 12, osteomyelitis of right humerus, porosity of costae 1-2 probably caused by a right, healed clavicular fracture. Morphological age estimate 50-60 yrs. Heavy tooth wear and dental caries. Man-TCA age est.: 86 yrs., Auto-TCA age est.: 90 yrs.
25	average	m	56-60	175	Extended position, orientation east-west with head at the west end. Skeleton buried in gravel surrounding. Artifact (knife with sheath, belt buckle), massive bone structure, bi-lateral arthritis of the foot joint, periosteal changes to right and left distal tibia. Morphological age estimate 50-60 yrs. Man-TCA age est.: 58 yrs., Auto-TCA age est.: 59 yrs.

26	average	f	36-40	167.7	Extended position, orientation east-west with head at the west end. Burial was exposed in the winter, wet conditions and freezing temperatures destroyed the structural integrity of the compact bone. Morphological age estimate 26-30 yrs. Man-TCA age est.: 42 yrs., Auto-TCA age est.: 37 yrs.
27	good	m	41-45	169.9	Extended position, orientation east-west with head at the west end. Foot skeleton embedded in concrete garden wall. Skeleton in humus layer. Stones ring grave border at head. Massive bone structure. Right humeral head slightly degenerative. LV 4-5 and TV 8, 10-12 have small osteophytes, TV 7, 9-11 show Schmorl's nodes, periodontal disease upper right m2, moderate to heavy tooth wear and resorption in mandible m1-m3 bi-lateral. Morphological age estimate 51-55 yrs. Man-TCA age est.: 42 yrs., Auto-TCA age est.: 38 yrs.
28	poor	tm	adult	166.2	Extended position, orientation east-west with head at the west end. Skeleton partially destroyed by lime pit. Elements above the proximal femur are missing.
29	good	f	36-40	154.6	Extended position, orientation east-west with head at the west end. Skeleton in humus layer. Beginning arthritis of the knee joint (condylar porosity of left distal femur), Schmorl's nodes TV 9-11. Minimal tooth wear.
30	good	tm	neonate	nd	Extended position, orientation east-west with head at the west end. Possible stone border.
31	poor	m	adult	nd	Extended position, orientation east-west with head at the west end. Periodontal disease and positional abnormality with incisors and canine.
32	good	m	15 ±36m	160.2	Extended position, orientation east-west with head at the west end. Foot skeleton cemented in garden wall. Cranium destroyed by winter exposure. Age estimation according to dentition.
33	average	f	adult	155.3	Extended position, orientation east-west with head at the west end. Skeleton in humus layer. Skeletal elements above the 6th thoracic vertebra are missing. No pathologies or age related alterations (lipping) are visible, which leads to the presumption that the individual is a probably a young adult.
34	poor	f	adult	155	Extended position, orientation east-west with head at the west end. Skeletal elements above the sacrum are missing. Destruction by grave pits 40 and 35. Reactive bone growth of the hip joint, beginning arthritis, likely of late adult age.
35	average	m	>60	180.1	Extended position, orientation east-west with head at the west end. Very robust bone structure. Osteom on left medial mid-femur diaphysis. Humerus and scapula show slight lipping, as does the right acetabulum. LV 1-5 degenerative, osteophytes on 3-4 and corpus compression of LV 4 left side. TV 11-12 pronounced osteophyte formation. Moderate tooth wear. Morphological age estimate 50-60 yrs. Man-TCA age est.: 73yrs., Auto-TCA

					age est.: 67 yrs.
36	poor	f	46-50	161.8	Extended position, orientation east-west with head at the west end. Skeleton partially damaged by winter exposure. Slight lipping of right femoral head. LV 2, 4 have osteophytes. Man-TCA age est.: 43 yrs., Auto-TCA age est.: 48 yrs.
37	average	m	>60	167.5	Extended position, orientation east-west with head at the west end. Garden wall above skeleton at about the knee. Winter exposure rendered the bone brittle, measurements made in situ. Morphological age thought to be between 60-70 yrs. Man-TCA age est.: 70yrs.
38	average	m	adult	162.5	Extended position, orientation east-west with head at the west end. Distal half of the lower extremities pinned under the garden wall. Earth is very moist. Epiphyseal line still partially visible. Age of individual is therefore probably early adult.
39	poor	m	adult	155	Extended position, orientation east-west with head at the west end. Lateral stone border. Skeleton is buried in gravel. Dental caries, periodontal disease, and heavy tooth wear.
40	good	f	15 ±36m	171.3	Extended position, orientation north-south with head at the north end. Artifact (bone comb). Age based on epiphyseal development and dentition. Man-TCA age est.: 17 yrs.
41	average	f	adult	158.8	Extended position, orientation east-west with head at the west end. Skeleton in humus layer. Reactive bone growth of the left femoral head.
42	good	m	41-45	162.1	Extended position, orientation north-south with head at the north end. Osteophytes on the dens axis, heavy tooth wear. Morphological age thought to be between 60-70 yrs. Man-TCA age est.: 45 yrs., Auto-TCA age est.: 40 yrs. Clear example for an older biological age compared to the actual chronological age estimate.
43	poor	f	31-35	nd	Extended position, orientation east-west with head at the west end. Weathered. Disturbed position of the bones gives impression that it is a secondary burial. Delicate bone structure. Heavy tooth wear. Morphological age estimate 30-40 yrs. Man-TCA age est.: 32 yrs., Auto-TCA age est.: 31 yrs.
44	poor	nd	adult	158	Extended position, orientation north-south with head at the north end. Stone ringed border. Heavy damage by weathering.
45	poor	m	>60	nd	Extended position, orientation north-south with head at the north end. Stone border near head. Most of post cranial skeleton missing, destroyed by grave 14. Morphological age estimation thought to be between 60-70 yrs. Man-TCA age est.: 72 yrs., Auto-TCA age est.: 89 yrs.

46	poor	nd	<1	53	Orientation appeared to be east-west; destruction by backhoe complicated the observation. Removed en bloc.
47	poor	nd	ca. 2	82	Oriented northeast-southwest. Skeleton disheveled, original position altered by careless digging. Only partially preserved.
48	average	m	46-50	nd	Extended position, orientation east-west with head at the west end. Underneath burial 2. Robust bone structure. Defect on left caput femoris. Degenerative disease on the inferior articulation of atlas. Mild to moderate tooth wear. M1 lower left resorbed. Morphological age estimate 50-60 yrs. Man-TCA age est.: 48 yrs., Auto-TCA age est.: 53 yrs.
49	poor	m	31-35	175	Extended position, orientation east-west with head at the west end. Skeleton only partially preserved. Elements exhibit no signs of degeneration and are robust with pronounced muscle attachments. Age estimation is not accurate; however, an age of mid-adult is also plausible 30-40 yrs. Man-TCA age est.: 35 yrs., Auto-TCA age est.: 37 yrs.
50	good	m	31-35	181.5	Extended position, orientation east-west with head at the west end. Partially disturbed by grave 41. Artifact (belt buckle). In situ measurement 193 cm. Extremely large and robust individual. M3s are lacking, m2 lower left and incisors 1-2 and canine resorbed. Man-TCA age est.: 34 yrs., Auto-TCA age est.: 39 yrs.
51	poor	m	adult	166.7	Extended position, orientation east-west with head at the west end. Partially under grave 40. Tooth resorption-upper left m3.
52	average	m	51-55	169.8	Extended position, orientation north-south with head at the north end. In gravel layer. Artifacts (leather pieces on chest area, iron knife blade, coin near head, precious metal and iron pieces underneath head). Bone structure robust. Morphological age estimate approx. 30-40 yrs. Man-TCA age est.: 52 yrs., Auto-TCA age est.: 57 yrs.
53					Grave orientation north-south. Stone border. No content, appears to have been destroyed by overlying burial.
53b	average	m	41-45	nd	Orientation not known. Archaeological documentation does not exist, the burial, however, does. The skeleton is labeled as Volders 53b. Morphological age estimate 30-40 yrs. Man-TCA age est.: 42 yrs., Auto-TCA age est.: 43 yrs.
54	poor	m	>60	nd	Extended position, orientation east-west with head at the west end. Two layered stone border. Majority of the skeleton disintegrated, Extensive tooth resorption (pm2, m1-m3 in both upper quadrants and one lower) as well as dental caries. Morphological age estimate 40-60 yrs.? Man-TCA age est.: 68 yrs., Auto-TCA age est.: 84 yrs.
55					No data
56	good	f	56-60	157.4	Extended position, orientation north-south with head at the north end. Underneath burial 42. Slight lipping on right

					caput femoris. Delicate bone structure. Moderate tooth wear, prognathic tooth position. Anomalous tooth- m1 lower right quadrant. M3 lower left is resorbed. Morphological age estimate 50-60 yrs. Man-TCA age est.: 54 yrs., Auto-TCA age est.: 58 yrs.
57	good	m	>70	171.9	Extended position, orientation north-south with head at the north end. Very robust bone structure. Periodontitis in labial area of mandible. Morphological age estimate >70 yrs. Man-TCA age est.: 72 yrs., Auto-TCA age est.: 63 yrs.
58					No data
59	average	m	36-40	165.8	Extended position, orientation east-west with head at the west end. Morphological age estimate 30-40 yrs. Man-TCA age est.: 40 yrs., Auto-TCA age est.: 43 yrs.
60	good	f	adult	164.7	Extended position, orientation north-south with head at the north end. Bi-lateral acetabular lipping, extensive tooth resorption and dental caries.
61	average	m	31-35	170.3	Extended position, orientation east-west with head at the west end. Healed right tibia fracture with anatomical dislocation and lesion on the medial/posterior condylar surface, medial condyle of right femur shows arthritic degeneration probably caused by fracture related positional change. Mild tooth wear. Morphological age estimate 30-40 yrs. Man-TCA age est.: 35 yrs., Auto-TCA age est.: 38 yrs.
62					No data
63	poor	nd	adult?	nd	Extended position, orientation east-west with head at the west end. Stone bordering at the north side. Burial contained very little skeletal material.
64	poor	nd	adult	nd	Extended position, orientation east-west with head at the west end. Skeleton is missing for the most part. Lower extremities missing. Slight lipping on the humeral head
65	average	m	36-40	159.6	Extended position, orientation east-west with head at the west end. Lime pit destroyed right lower extremity. Skeleton in humus layer. Robust bone structure. Osteophytes on LV 1-2 and TV 6-8. Exostosis on the medial condyle of the left femur as well as the pelvis near the greater sciatic notch. Tooth resorption in both upper quadrants (right m1-m3 and left m1). Man-TCA age est.: 44 yrs., Auto-TCA age est.: 36 yrs.
66	good	m	46-50	164.7	Extended position, orientation east-west with head at the west end. Skeleton only approx. 40 cm under the surface in the humus layer. Skeletal elements were frozen together, especially vertebrae. Partially damaged by backhoe (cranium and CV). Pronounced muscle attachments and robust bone structure. Osteophytes on LV 1-5 and TV 8-10. Morphological age estimate 36-40 yrs. Man-TCA age est.: 50 yrs., Auto-TCA age est.: 47 yrs.
67	poor	m	>60	165.1	Extended position, orientation east-west with head at the

					west end. Synostosis of the sacrum with the 5th lumbar vertebra, arthritis of the hip joint, degenerative caput femoris, Schmorl's nodes on LV. Morphological age approx. 60-70 yrs. Man-TCA age est.: 78 yrs., Auto-TCA age est.: 73 yrs.
68	average	m	36-40	165.8	Extended position, orientation east-west with head at the west end. Robust bone structure. Arthritis of the 4th lumbar vertebra. Morphological age estimate 40-50 yrs. Man-TCA age est.: 38yrs.
69	average	f	adult	155.6	Extended position, orientation east-west with head at the west end. Skeleton in humus layer. Right femur partially dissolved by lime. Cranium missing.
70	average	m	46-50	176.1	Extended position, orientation north-south with head at the north end. Cranium missing. Removed at once because of snowfall. Man-TCA age est.: 49 yrs., Auto-TCA age est.: 53 yrs.
71	poor	f	26-30	152.8	Extended position, orientation east-west with head at the west end. In humus layer. Large portion of the skeleton is missing. Delicate bone structure. Epiphyseal lines visible. Man-TCA age est.: 26 yrs., Auto-TCA age est.: 31 yrs.
72					No data. Although the archaeological record indicates a cranium, upper extremities and ribs were documented. Skeletal elements have apparently been misplaced and no anthropological field documentation exists.
73	poor	f	31-35	nd	Extended position, orientation east-west with head at the west end. Postcranial skeleton largely destroyed by lime pit. Morphological age estimate 30-40 yrs. Man-TCA age est.: 32 yrs., Auto-TCA age est.: 40 yrs.
74	poor	f	>60	nd	Extended position, orientation east-west with head at the west end. Cranium badly damaged by dig workers (pick-axe) and lower extremities distal from both femoral metaphysis were destroyed by the lime pit. Morphological age thought to be just over 60 yrs. Man-TCA age est.: 59 yrs., Auto-TCA age est.: 58 yrs.
75					No data
76	poor	tm	adult	167.8	Extended position, orientation east-west with head at the west end. Skeleton represented by scant remains, both tibia and foot skeleton remained in earth.
77	poor	m	adult	168.3	Extended position, orientation east-west with head at the west end. Partial stone border. Robust bone structure. Cranium and thoracic elements missing.
78	average	nd	neonate	nd	Extended position, orientation east-west with head at the west end. Soil very wet, bones soft. Unfortunately, the photographer tripped and stepped onto the skeleton thereby incurring irreparable damage to most of the skeleton.
79	good	f	41-45	159.8	Extended position, orientation east-west with head at the west end. Moderately robust bone structure. Small

					osteophytes on TV 10. Moderate tooth wear, tooth resorption lower left quadrant-m1-m3. Morphological age estimate 50-60 yrs. Man-TCA age est.: 39 yrs., Auto-TCA age est.: 42 yrs.
80	poor	f	26-30	nd	Extended position, orientation east-west with head at the west end. Lower extremities partially disturbed by grave 114. Iliac crest is not completely fused and the epiphyseal line on the caput femoris is still visible. Delicate bone structure.
81	average	m	46-50	172.2	Extended position, orientation east-west with head at the west end. Several stones in a line near the left foot. Cranium missing. Morphological age estimation of 31-35 yrs. is questionable. Lipping on the superior articular surface of the calcaneus would seem to indicate an age older than 31-35; however, tooth wear was absolutely minimal and no other lipping or signs of wear and tear are visible on the rest of the skeleton. The presence of a hypoplastic anomaly indicates physiological stress factor during infancy. Man-TCA age est.: 50 yrs., Auto-TCA age est.: 68 yrs. Combination of the morphological and TCA method for acquiring an age estimation was used.
82	poor	m	adult	166.7	Extended position, orientation north-south with head at the north end. Majority of skeleton destroyed by garage construction. Parts of lower extremities cemented into garden wall.
83	poor	f	adult	nd	Extended position, orientation north-south with head at the north end. Identical situation as for burial 82.
84	poor	m	adult	174	Extended position, orientation north-south with head at the north end. See burial 82. Bones present are very robust.
85	good	m	adult	166.5	Extended position, orientation east-west with head at the west end. Skeleton cemented into garden wall below the knee. Robust bone structure and pronounced muscle attachments on the upper extremities. Ribs are very robust. Schmorl's nodes on TV 8. Heavy tooth wear, teeth exhibit angular wear pattern.
86	good	m	26-30	174	Extended position, orientation east-west with head at the west end. Stones ring the grave around the head area. Both foot skeletons are cemented into garden wall. Bone structure is generally robust. Epiphyseal line on caput femoris visible as is the iliac crest apophysis border. Large peri-apical abscess (upper right quadrant between pm2-m3, periodontal disease and tooth resorption. Man-TCA age est.: 26 yrs., Auto-TCA age est.: 27 yrs.
87	average	m	adult	156.5	Extended position, orientation east-west with head at the west end. Skeleton under the garden wall at about mid-section. Skeleton is wet and soft. Calcified cricoid cartilage probably indicates individual is late adult. Moderate tooth wear, dental caries and tooth resorption (upper left 5-8; lower left 6, 8).
88	average	nd	1-1.5	nd	Extended position, orientation east-west with head at the

					west end. Skeleton is wet and heavily fragmented.
89	average	nd	4-5	90	Extended position, orientation east-west with head at the west end. Skeleton is severely fragmented, the height estimation is based upon an *in situ* whole body measurement. Age based upon development of deciduous dentition.
90	good	m	56-60	167	Extended position, orientation east-west with head at the west end. Stone border to the sides and at head. Strong skeletal build. Manubrium and corpus s. are fused (possibly older than 40-50) Schmorl's nodes on LV 1, 3. Heavy tooth wear, resorption lower right m2. Incisors appear to have been used as a clamping tool (cuspal grooving). Morphological age estimate 40-50 yrs. Man-TCA age est.: 58 yrs., Auto-TCA age est.: 61 yrs.
91	average	m	>60	162.4	Extended position, with right side rolled over to the left. Orientation east-west with head at the west end. Stone border on both sides of grave. Lesion on posterior surface of the right femur, possibly by injury, resulted in osteomyelitis. Caries on lower right m2. Lower left show evidence for periodontitis and tooth resorption pm2, m1 and m3. Morphological age approx. 60-70 yrs. Man-TCA age est.: 62 yrs., Auto-TCA age est.: 74 yrs.
92	poor	m	adult	nd	Extended position, orientation east-west with head at the west end. Postcranial skeleton under the garden wall. Osteophytes on the CV.
93	good	f	46-50	157.9	Extended position, orientation east-west with head at the west end. Delicate bone structure. Variation: septal aperture right humerus. Resorption of incisor 2 and canines in both lower quadrants, m3s not present. Man-TCA age est.: 46 yrs., Auto-TCA age est.: 39 yrs.
94	average	f	ca. 7-9	nd	Extended position, orientation east-west with head at the west end. Soil very moist. Age estimation based on femur length and bone development.
95	poor	tf	adult	169.7	Extended position, orientation east-west with head at the west end. Disturbed by grave 97. Elements are jumbled and need to be reconstructed to provide examination result. Moderately robust skeletal structure. LV 1-5 and TV have osteophytes and show signs of degenerative joint disease of the vertebral bodies, Schmorl's nodes on LV 1-5 and TV 8-12. Vertebral bodies are partially deformed.
96	good	f	adult	164.4	Extended position, orientation east-west with head at the west end. Extensive tooth resorption, nearly all teeth lost in both lower quadrants.
97	average	m	adult	165.9	Extended position, orientation east-west with head at the west end. Skeleton is at edge of dig tent and got wet. Bone structure is robust.
98	good	m	15 ±36m	nd	Extended position, orientation east-west with head at the west end. Stone border at the left side of the grave. Age based upon dental development and epiphyseal growth.

					Lesion on frontal bone (infection?). Man-TCA age est.: 14 yrs., Auto-TCA age est.: 27 yrs.
99	average	f	36-40	161	Extended position, orientation east-west with head at the west end. Cranium was dissolved by contents of lime pit. Muscle attachment of legs (linea aspera) extremely pronounced. Heavy tooth wear in mandibular quadrants. Teeth from maxilla are damaged by lime. Morphological age estimate between 30-40 yrs. Man-TCA age est.: 38 yrs., Auto-TCA age est.: 34 yrs.
100	good	m	46-50	175.7	Extended position, orientation east-west with head at the west end. Grave ringed by large stones. Very robust bone structure, especially the forearms. LV 1-3 and TV 5-12 Schmorl's nodes. Osteophytes on LV 4-5 and TV 4-5, 7-8, 10. Squatting facet, Hiatus canalis sacralis, minimal to moderate tooth wear, and nearly complete molar tooth resorption in both lower quadrants. Morphological age estimate 36-40 yrs. Man-TCA age est.: 50 yrs., Auto-TCA age est.: 46 yrs.
101	good	f	41-45	165.1	Extended position, orientation east-west with head at the west end. Weathering and soil pressure incurred damage. Moderate bone structure, yet pronounced muscle attachments. Healed fracture of the right distal ulna with slight dislocation. Advanced caries and abscess in upper left jaw m1-m2, resorption m3. Morphological age estimate 40-50 yrs. Man-TCA age est.: 42 yrs., Auto-TCA age est.: 40 yrs.
102	average	nd	ca. 7-8	114.3	Extended position, orientation east-west with head at the west end. Age estimation based on dental and long bone development.
103	good	m	26-30	168	Extended position, orientation east-west with head at the west end. Foot skeleton in garden wall. Robust bone structure. Iliac crest and medial clavicle still fusing. Mild to moderate tooth wear, dental calculus. Man-TCA age est.: 27 yrs., Auto-TCA age est.: 27 yrs.
104	good	f	16 ±24m	129.2	Extended position, orientation east-west with head at the west end. In gravel layer. Juvenile individual, m3s erupting in maxilla but not in mandible. Man-TCA age est.: 16 yrs., Auto-TCA age est.: 23 yrs.
105	poor	f	>60	nd	Extended position, orientation east-west with head at the west end. Lower extremities disturbed by garden wall, upper half destroyed by another grave pit. Stout build, pronounced muscle attachments. Degenerative joint disease of LV. Morphological age estimate 50-60 yrs. Man-TCA age est.: 62 yrs., Auto-TCA age est.: 65 yrs.
106	poor	m	adult	165.6	Extended position, orientation east-west with head at the west end. All skeletal elements above the proximal femur destroyed by lime pit. Underneath the left tibia is an infant burial. Massive bone structure.
107	good	m	26-30	170.2	Extended position, orientation east-west with head at the west end. Lower extremities pinned under garden wall at

					about mid-tibia. Bone defect similar to a lesion on the left distal third of the radial diaphysis. Schmorl's nodes on TV 6-7, 12. Mild to moderate tooth wear. Dental caries and abscess between pm2-m1.
108	good	m	46-50	177.3	Extended position, orientation east-west with head at the west end. Foot skeleton cemented into garden wall. Partial stone border. Powerfully built stature. Bones are massive in structure with well developed muscle attachments. Evidence for a traumatic injury to the sacrum at promontory, compression of LV 4-5, perhaps as the result of irreparable damage to the LV 5-S 1 disc. Schmorl's nodes on LV 1-2 and TV 7-12 as well as beginning osteophytosis. Heavy tooth wear. Morphological age estimate 40-50 yrs. Man-TCA age est.: 50 yrs., Auto-TCA age est.: 46 yrs.
109	poor	tf	adult	162.8	Flexed position, orientation east-west with head at the west end. Skeleton above proximal femur destroyed by lime pit. Delicate to moderate bone structure.
110	good	m	26-30	174.2	Extended position, orientation east-west with head at the west end. Bones are wet. Bone structure is robust. Median spine of the sacrum is not completely fused. Moderate tooth wear in maxilla. Man-TCA age est.: 27 yrs., Auto-TCA age est.: 36 yrs.
111	average	m	adult	171.8	Extended position, orientation east-west with head at the west end. Garden wall runs over the skeleton at about the pelvis. Partial stone border or stone ringed post hole. Bone structure robust, especially in the upper arms where the muscle attachments are very pronounced. Caries of lower right pm2 and resorption of pm1, m1, and m3. Resorption of m1 lower left. Moderate tooth wear.
112	good	m	>60	171.6	Extended position, orientation east-west with head at the west end. Several stones border the grave pit. Pronounced muscle attachment on the lower extremities. Reactive bone growth on the femoral head, arthritic destruction of the acetabulum. LV 2 and TV 7-10 have small osteophytes. Molar tooth resorption in all quadrants. Heavy tooth wear and dental calculus. Morphological age estimation 56-60 yrs. Man-TCA age est.: 88 yrs., Auto-TCA age est.: 112yrs.
113	good	m	>60	162.2	Extended position, orientation east-west with head at the west end. Garden wall directly above waist area. Degenerative joint disease, not DISH of the spinal column. Ankylosis of the TV 1-4, 5-8 and 10-12+LV1, and osteophytes on LV 2-4. Dental caries in upper right m3, tooth resorption of lower right m3. Moderate tooth wear. Morphological age thought to be between 60-70 yrs. Man-TCA age est.: 68 yrs., Auto-TCA age est.: 58 yrs.
114	good	f	>60	158.2	Extended position, orientation east-west with head at the west end. Stones at border of grave. Diminutive stature. Beginning arthritis of the left acetabulum, caries upper right pm1, extensive tooth resorption upper right incisor 1-2 and m2-m3; upper left incisor 1; lower right m1-m2; lower left m1-m3. Moderate tooth wear. Morphological

					age thought to be between 60-70 yrs. Man-TCA age est.: 66yrs., Auto-TCA age est.: 75 yrs.
115	poor	nd	ca. 6-7	ca. 102	Extended position, orientation east-west with head at the west end. Skeleton in fragments, height based on left humerus length. Infant skeleton underneath this burial.
116	good	m	>60	177.2	Extended position, orientation east-west with head at the west end. Relatively robust bone structure. Kyphosis, reactive bone growth on the patella, lesion of the right humeral diaphysis. Lipping on the left humeral head. Tooth resorption lower left pm2-m1, moderate tooth wear. Lesion on frontal bone (infection). Morphological age estimate 60 yrs. Man-TCA age est.: 54 yrs., Auto-TCA age est.: 71 yrs.
117	poor	nd	perinatal	nd	Orientation not known. Impression is that baby was buried in a sitting position. Only a few skeletal elements are present.
118	average	m	adult	168.2	Extended position, orientation east-west with head at the west end. Skeleton is trapped under the garden wall at about elbow level. Osteophyte on the dens axis. Dental calculus, caries on pm2 lower right quadrant. Mild to moderate tooth wear.
119	poor	nd	ca. 2-3	53	Extended position, orientation east-west with head at the west end. Heavy damage through burial 114. Height and age based on tibial length.
120	poor	tm	adult	171.5	Extended position, orientation east-west with head at the west end. Majority of skeleton destroyed by nearby grave. Only lower extremities, epiphyseal line still visible, therefore probably young adult.
121	poor	m	adult	174.2	Extended position, orientation east-west with head at the west end. Cranium missing, postcranial skeleton below the waist is under the garden wall, robust stature,
122					No data
123	good	f	18-19	163	Extended position, orientation east-west with head at the west end. Artifact: Green color on left radius and ulna indicate a bracelet was worn. Intricate glass pearl necklace found with skeleton, possible indication of status. Man-TCA age est.: 18 yrs., Auto-TCA age est.: 29 yrs.
124	average	nd	ca. 6-7	nd	Extended position, orientation east-west with head at the west end. Severe dental caries, possible lethal cranial fracture in parietal and frontal bone.
125	average	f	adult	154	Extended position, orientation north-south with head at the north end. Stone border. Artifact: arm bracelet and pearl necklace. Synostosis of the right caput femoris in the acetabulum.
126	average	m	adult	160	Extended position, orientation north-south with head at the north end. Artifact: belt buckle, leather remains, sheath, iron knife. Moderate bone structure. Heavy tooth wear.

127	poor	nd	adult	nd	Extended position, orientation east-west with head at the west end. Very poor preservation. Artifact: knife blade.
128					No data
129	good	f	26-30	164.5	Extended position, orientation east-west with head at the west end. Postcranial skeleton below CV under garden wall. During the recovery, the deeper position of the postcranial elements allowed their removal to the innominate bone. Man-TCA age est.: 30 yrs., Auto-TCA age est.: 37 yrs.
130					No data
131					Grave orientation east-west. No content
132	good	m	36-40	171.2	Extended position, orientation east-west with head at the west end. Very robust bone structure. Schmorl's nodes 5th LV. TV 11-12 have osteophytes and evidence of a herniated disk, the atlas is fused to the base of the cranium, the axis is fused with the 3rd cervical vertebra (block vertebrae), lesion on the occipital portion of the skull, tooth resorption m3 in all four quadrants, caries upper left m1, mild tooth wear and calculus. Man-TCA age est.: 44 yrs., Auto-TCA age est.: 40 yrs.
133	poor	nd	neonate	nd	Extended position, orientation east-west with head at the west end. Uncertainty with respect to exactness of age due to poor preservation of necessary features.
134	average	nd	ca. 3-5	97.7	Extended position, orientation east-west with head at the west end. Disturbed by the construction of grave 140. Age and height estimation based upon femur length.
135	average	m	50-60	168.7	Extended position, orientation east-west with head at the west end. Below LV 3 cemented into garden wall. Stones ring the upper border of the grave pit. Osteophytosis CV 1-7, TV 1-7, and LV 1-2. Heavy tooth wear. Variation: septal aperture on right humerus. One of the few examples where the morphological and TCA age at death estimations contrasted significantly. Man-TCA age est.: 26 yrs., Auto-TCA age est.: 30 yrs. For the life table the morphological age estimate was considered more reliable and entered as 51-55 yrs.
136	good	m	ca. 17-19	168.2	Extended position, orientation east-west with head at the west end. Skeleton destroyed by lime pit above knee. Late juvenile based on epiphyseal development.
137	poor	m	adult	166.2	Extended position, orientation east-west with head at the west end. Destruction by the lime pit. Robust bone structure. Squatting facet.
138	poor	tm	adult	173.5	Extended position, orientation east-west with head at the west end. Disturbed by another grave pit.
139	average	f	>60	162.3	Extended position, orientation east-west with head at the

					west end. Skeleton gives the appearance that body was buried in a sitting / reclining position. Very heavy tooth wear, and last stage pubic symphysis surface according to Todd indicates high age. Originally estimated at 40-50yrs based upon morphological data. Man-TCA age est.: 72 yrs., Auto-TCA age est.: 71 yrs.
140	poor	m	adult	169.1	Extended position, orientation east-west with head at the west end. Lipping of the proximal tibia, patella, and acetabulum. Lateral bowing of the fibula.
141	poor	nd	ca. 5-7	98.2	Extended position, orientation east-west with head at the west end. Disturbed by backhoe work.
142	poor	tm	adult	168.1	Extended position, orientation east-west with head at the west end. Majority of skeleton lost by construction work. Only lower extremities present.
143	poor	m	adult	172.1	Extended position, orientation east-west with head at the west end. See burial 142.
144	average	m	>60	169.1	Extended position, orientation north-south with head at the north end. Directly parallel to the garden wall. Robust bone structure. Defect on the patellar articular surface. Extensive tooth resorption-upper right incisor 1-2, pm1, m2-m3; upper left 1-8 resorbed; lower right m1-m3; lower left 5-8 resorbed. Very heavy tooth wear. Morphological age thought to be between 60-70. Man-TCA age est.: 69 yrs., Auto-TCA age est.: 71 yrs.
145	average	m	>60	172.8	Extended position, orientation east-west with head at the west end. Lower half of skeleton is in concrete retaining wall. Lipping on right humeral head, osteophytes on LV 1, heavy tooth wear. Morphological age estimate thought to be between 60-70. Man-TCA age est.: 56 yrs., Auto-TCA age est.: 78 yrs. A combination of all three estimates was used for the age estimation.
146	good	f	adult	164.5	Extended position, orientation east-west with head at the west end. Cranium and CV 1-6 removed by backhoe. Bone structure not very robust. Lesion-like bone defects near the facies auricularis might indicate parturition. Small osteophytes on LV 5.
147	good	f	adult	nd	Extended position, orientation east-west with head at the west end. Majority of skeleton is under the garden wall. Incomplete epiphyseal closure of the acromium on the scapula indicates a very young adult or even late juvenile.
148	poor	m	>60		Extended position, orientation north-south with head at the north end. Skeleton seems to be a secondary burial with mixture of other skeletal elements. Left half of mandible shows tooth resorption pm2-m3. Age originally estimated between 50-60 yrs, TCA estimation was, however, significantly higher and the age was therefore adjusted. Man-TCA age est.: 88 yrs., Auto-TCA age est.: 105 yrs.
149	poor	m	adult	155	Extended position, orientation north-south with head at the north end. Grave borders on riverbed sand and is

					embedded in gravel. Most of the skeleton has been decomposed by taphonomic processes. Reactive bone growth on right talus. Robust bone structure.
150	poor	f	12-14	nd	Extended position, orientation east-west with head at the west end. Clearly defined stone border. Postcranial skeleton is covered by garden wall. Virtually no tooth wear of permanent dentition observable, m3 not erupted.
151	poor	m	51-55	170.6	Extended position, orientation east-west with head at the west end. Same position as burial 150. Upper arm are robust with pronounced muscle attachments. Moderate tooth wear. Age originally listed as 30-40 yrs. According to morphological examination, later adjusted using TCA information. Man-TCA age est.: 55 yrs., Auto-TCA age est.: 58 yrs.
152	average	nd	<1		Orientation is not discernable.
153	average	m	51-55	175.5	Extended position, orientation east-west with head at the west end. Most of the postcranial skeleton has been severely damaged by the gravel bed burial surroundings. Heavy tooth wear and resorption, robust bone structure. Morphological age estimate 50-60 yrs. Man-TCA age est.: 57 yrs., Auto-TCA age est.: 48 yrs.
19152	poor	m	>60	nd	Museum artifact represented by a skull and several small fragments. The skull was recovered during street work in 1960 in the Augasse, directly adjacent to the present archaeological site. Man-TCA age est.: 67yrs.

REFERENCES

Acsádi G. & Nemeskéri J., 1970. History of human life span and mortality. Akademiai Kiado, Budapest.

Ambrose S.H., 1986. Stable carbon and nitrogen isotope analysis of human and animal diet in Africa. *Journal of Human Evolution* **15**: 707-731.

Ambrose S.H., 1987. Chemical and isotopic techniques of diet reconstruction in eastern North America. In: Keegan W.F. (ed.). *Emergent Horticultural Communities of the Eastern Woodlands*. Carbondale, Illinois: Center for Archaeological Investigations, Occasional Paper **7**: 87-107.

Ambrose S.H., 1990. Preparation and characterization of bone and tooth collagen for stable carbon and nitrogen isotope analysis. *Journal of Archaeological Science* **17**: 431-451.

Ambrose S.H., 1991. Effects of diet, climate and physiology on nitrogen isotope abundances in terrestrial foodwebs. *Journal of Archaeological Science* **18**: 293-317.

Ambrose S.H., 1993. Isotopic Analysis of Palaeodiets: Methodological and Interpretive Considerations. In: Sanford M.K. (ed.). *Investigations of Ancient Human Tissue: Chemical Analysis in Anthropology*. Amsterdam: Gordon & Breach Science Publications. 59-130.

Ambrose S.H., 1998a. Chronology of the Later Stone Age and food production in East Africa. *Journal of Archaeological Science* **25**: 377-392.

Ambrose S.H., 1998b. Prospects for stable isotopic analysis of Later Pleistocene hominid diets in West Asia and Europe. In: Akazawa T., Aoki K. & bar-Yosef O. (eds.). *Origin of Neanderthals and Modern Humans in West Asia*. New York: Plenum Press. 277-289.

Ambrose S.H., 2000. Controlled diet and climate experiments on nitrogen isotope ratios of rats. In: Ambrose S.H. & Katzenberg M.A. (eds.). *Biogeochemical Approaches to Paleodietary Analysis*. New York: Kluwer Academic/Plenum Publishers. 243-259.

Ambrose S.H. & DeNiro M.J., 1986. Reconstruction of African human diet using bone collagen carbon and nitrogen isotope ratios. *Nature* **319**: 321-324.

Ambrose S.H. & DeNiro M.J., 1989. Climate and habitat reconstruction using stable carbon and nitrogen isotope ratios of collagen in prehistoric herbivore teeth from Kenya. *Quaternary Research* **31**: 407-422.

Ambrose S.H. & Norr L., 1993. Isotopic composition of dietary protein and energy versus bone collagen and apatite: purified diet growth experiments. In: Lambert J. & Grupe G. (eds.). *Molecular Archaeology of Prehistoric Human Bone*. Berlin: Springer. 1-37.

Ambrose S.H., Butler B.M., Hanson D.B., Hunter-Anderson R.L. & Krueger H.W., 1997. Stable isotopic analysis of human diet in the Mariana Archipelago, western Pacific. *American Journal of Physical Anthropology* **104**: 343-361.

Arneborg J., Heinemeier J., Lynnerup N., Nielson H.L., Rud N. & Sveinbjornsdottir A.E., 1999. Change of diet of the Greenland Vikings determined from stable carbon isotope analysis and ^{14}C dating of their bones. *Radiocarbon* **41**(2): 157.

Asam T., Bösl C., Grupe G., Lösch S., Manhart H., Mekota A.M. & Peters J., 2004. Palaeoecosystem reconstruction and the Neolithic Transition in temperate climates. In: Grupe G. & Peters J. (eds.). *Documenta Archaeobiologiae: Conservation policy and current research, Vol.2*. 97-137.

Bach H., 1965. Zur Berechnung der Körperhöhe aus den langen Gliedmaßenknochen weiblicher Skelette. *Anthropologischer Anzeiger* **29**: 12-21.

Balzer A., Gleixner G., Grupe G., Schmidt H.-L., Schramm S. & Turban-Just S., 1997. *In vitro* decomposition of bone collagen by soil bacteria: The implications for stable isotope analysis in archaeometry. *Archaeometry* **39**(2): 415-429.

Bamforth F., Jackes M. & Lubell D., 2000. Mesolithic-Neolithic population relationships in Portugal: the evidence from ancient mitochondrial DNA. The 6th International Symposium on the Mesolithic in Europe (MESO 2000). Oxford, U.K.: Archaeopress.

Baraybar J.P., 1997. Reconstruction of diet with trace elements of bone at the Chalcolithic site of Pico Ramos, Basque Country, Spain. *Journal of Archaeological Science* **24**(4): 355-364.

Baraybar J.P., 1999. Diet and death in a fog oasis site in Central Coastal Peru: a trace element study of tomb 1 Malanche 22. *Journal of Archaeological Science* **26**(5): 471-482.

Beall C.M. & Steegmann Jr. A.T., 2000. Human Adaptation to Climate: Temperature, Ultraviolet Radiation, and Altitude. In: Stinson S., Bogin B., Huss-Ashmore R. & O'Rourke D. (eds.). *Human Biology, An Evolutionary and Biocultural Perspective*. New York: Wiley-Liss, Inc. 163-224.

Behrensmeyer A.K., 1978. Taphonomic and ecologic information from bone weathering. *Paleobiology* **4**: 150-162.

Bell L., Cox C. & Sealy J., 2001. Determining isotopic life history trajectories using bone density fractionation and stable isotope measurements: A new approach. *American Journal of Physical Anthropology* **116**: 66-79.

Ben-David M. & Schell D.M., 2001. Mixing models in analyses of diet using multiple stable isotopes: a response. *Oecologia* **127**: 180-184.

Bender M.M., 1968. Mass spectrometric studies of carbon 13 variations in corn and other grasses. *American Journal of Science, Radiocarbon Supplement* **10**: 468-472.

Bender M.M., Baerreis D.A. & Steventon R.L., 1981. Further light on carbon isotopes and Hopewell culture. *American Antiquity* **46**: 346-353.

Bentley R.A., 2001. Human Migration in Early Neolithic Europe, PhD. Dissertation, University of Wisconsin.

Bentley R.A., Price T.D. & Stephan E., 2004. Determining the "local" 87Sr/86Sr range for archaeological skeletons: a case study from Neolithic Europe. *Journal of Archaeological Science* **31**: 365-375.

Berkovitz B.K.B., Holland G.R. & Moxham B.L., 2002. *Oral Anatomy, Histology, Embryology 3rd ed*. Oxford: Elsevier Books. 168-179.

Bernasconi S.M., 2002. Paleoprecipitation $\delta^{18}0$ from Tree Rings and Lakes in sediments in Switzerland. *Science Highlight*. www.pages.unibe.ch.

Bocherens H., Fizet M., Mariotti A., Lange-Badre B., Van der Meersch B., Borel J.P. & Bellon G., 1991. Isotopic biogeochemistry (^{13}C, ^{15}N) of fossil vertebrate collagen: application to the study of a past food web including Neanderthal man. *Journal of Human Evolution* **10**: 481-492.

Bocherens H., Billiou D., Mariotti A., Patou-Mathias M., Otte M., Bonjean D. & Toussaint M., 1999. Palaeoenvironmental and palaeodietary implications of isotopic biogeochemistry of last interglacial Neanderthal and mammal bones in Scladina Cave (Belgium). *Journal of Archaeological Science* **26**: 99-607.

Bocherens H., Billiou D., Mariotti A., Toussaint M., Patou M., Marylene B. & Dominique O. M., 2001. New isotopic evidence for dietary habits of Neanderthals from Belgium. **40**: 497-505.

Bocquet J.P. & Masset C., 1977. Estimateurs en paléodemographie. *L'Homme* **17** : 65-90.

Bökönyi S., 1974. History of domestic mammals in Central and Eastern Europe. Budapest: Akadémiai Kiadó. 56-57.

Bonsall C., Lennon R., McSweeney K., Stewart C., Harkness D., Boroneant V., Bartosiewicz V., Payton R. & Chapman J., 1997. Mesolithic and early Neolithic in the Iron Gates: A palaeodietary perspective. *Journal of European Archaeology* **5**(1): 50-92.

Boskey A.L., 1999. Mineralization, Structure, and Function of Bone. In: Siebel M.J., Robins S.P. & Bilezikian J.P. (eds.). *Dynamics of Bone and Cartilage Metabolism*. San Diego, CA.: Academic Press. 153-164.

Bösl C., Grupe G. & Peters J., 2006. A late Neolithic vertebrate food web based on stable isotope analyses. *International Journal of Osteoarchaeology* **16**(4): 296-315.

Bowden A. & Tieszen L.L., 1999. Dietary reconstruction of human bone samples from early populations in the Central Columbian Andes, CA. 7000-4000BP, with the use of stable carbon and nitrogen isotopes. *Proceeding of the South Dakota Academy of Science* **78**: 233-234.

Bowen G. & Revenaugh J., 2003. Interpolating the isotopic composition of modern meteoric precipitation. *Water Resources Research* **39**(10): 1299, SWC 9.1-9.13.

Bowen G. & Wilkinson B., 2002. Spatial distribution of $\delta^{18}O$ in meteoric precipitation. *Geology* **30** (4): 315-318.

Boyle E.A., 1997. Cool tropical temperatures shift the global $\delta^{18}O$-T relationship: An explanation for the ice core $\delta^{18}O$-borehole thermometry conflict? *Geophysics Res. Lett.* **24**: 273-276.

Brain C.K., 1981. *The Hunters or the Hunted? An Introduction to African Cave taphonomy*. Chicago and London: The University of Chicago Press.

Breitinger E., 1938. Zur Berechnung der Körperhöhe aus den langen Gliedmaßenknochen. *Anthropologischer Anzeiger* **14**: 249-274.

Brooks S. & Suchey J.M., 1990. Skeletal age determination based on the os pubis: a comparison of the Acsádi-Nemeskéri and Suchey-Brooks methods. *Human Evolution* **5**: 227-238.

Bryant J.D. & Froelich P.N., 1995. A model of oxygen isotope fractionation in body water of large mammals. *Geochimica et Cosmochimica Acta* **59**: 4523-4537.

Bryant J.D., Koch P., Froelich P.N., Showers W. & Genna B.J., 1996. Oxygen isotope partitioning between phosphate and carbonate in mammalian apatite. *Geochimica et Cosmochimica Acta* **60**(24): 5145-5148.

Budd P., Montgomery J., Barreiro B. & Thomas R.G., 2000. Differential diagenesis of strontium in archaeological human dental tissues. *Applied Geochemistry* **15**: 687-694.

Budd P., Millard A., Chenery C., Lucy S. & Roberts C., 2004. Investigating population movement by stable isotope analysis: A Report from Britain. *Antiquity* **78**(299): 127.

Burger R.L. & van der Merwe N.J., 1990. Maize and the origin of Highland Chavin civilization: an isotopic perspective. *American Anthropologist* **92**: 85-95.

Burkitt H.G., Young B. & Heath J.W., 1993. *Wheater's functional histology. A text and colour atlas*. Edinburgh: Churchill Livingstone. 241.

Burton J.H., Price T.D., Cahue L. & Wright L.E., 2003. The Use of Barium and Strontium Abundances in Human Skeletal Tissues to Determine their Geographical Origins. *International Journal of Osteoarchaeology* **13**: 88-95.

Carey S.K. & Quinton W.L., 2004. Evaluating snowmelt runoff generation in a discontinuous permafrost catchment using stable isotope, hydrochemical and hydrometric data. *Nordic Hydrology* **35**(4-5): 309-324.

Cerling T.E., 1992. Development of grasslands and savannas in East Africa during the Neogene. *Palaeogeography Palaeoclimate Palaeoecology* **97**: 241-247.

Cerling T.E., Harris J.M., Ambrose S.H., Leakey M.G. & Solounias N., 1997. Dietary and environmental reconstruction with stable isotope analyses of herbivore tooth enamel from the Miocene locality of Fort Ternan, Kenya. *Journal of Human Evolution* **33**: 635-650.

Cerling T.E., Harris J.M. & MacFadden B.J., 1998. Carbon isotopes, diets of North American equids, and the evolution of North American C_4 grasslands. In: Griffiths H. (ed.). *Stable Isotopes and the Integration of Biological, Ecological, and Geochemical Processes*. Oxford: Bios Scientific Publishers. 363-379.

Champe P.C. & Harvey R.A., 1994. *Lippincott's Illustrated Reviews: Biochemistry*, 2nd ed.. Philadelphia: J.B. Lippincott Co. 335-337.

Chickerur N.S., Tung M.S. & Brown W.E., 1980. *Calcified Tissue International* **32**: 55-62.

Chisholm B.S., 1989. Variation in Diet Reconstructions Based on Stable Carbon Isotopic Evidence. In: Price T.D. (ed.). *The Chemistry of Human Bone*. Cambridge: Cambridge University Press. 10-37.

Chisholm B.S., Nelson D.E. & Schwarcz H.P., 1982. Stable carbon isotope ratios as a measure of marine versus terrestrial protein in ancient diets. *Science* **216**: 1131-1132.

Chisholm B.S., Nelson D.E. & Schwarcz H.P., 1983. Marine and terrestrial protein in prehistoric diets on the British Columbia Coast. *Current Anthropology* **24**: 396-398.

Cipriano-Bechtle A., Grupe G. & Schröter P., 1995. Ageing and life expectancy in the early Middle Ages. *Homo* **46**(3): 267-279.

Collins M.J., Nielsen-Marsh C.M., Hiller J., Smith C.I. & Roberts J.P., 2002. The survival of organic matter in bone: a review. *Archaeometry* **44**(3): 383-394.

Coltrain J.B., Harris J.M., Cerling TE., Ehleringer J.R., Dearing M.D., Ward J. & Allen J., 2004. Rancho La Brea stable isotope biogeochemistry and its implications for the Palaeoecology of late Pleistocene coastal southern California. *Palaeogeography, Palaeoclimatology, Palaeoecology* **205**: 199-219.

Coltrain J.B., Willliams D.G., Lott M., English N.B. & Ehleringer J.R., 2005. Oxygen isotopes in cellulose identify source water for archaeological maize in the American Southwest. *Journal of Archaeological Science* **32**(6): 931-939.

Condon K., Charles D., Cheverud J.M. & Buikstra J.E., 1986. Cementum annulation and age determination in Homo sapiens II, estimates and accuracy. *American Journal of Physical Anthropology* **71**: 321-330.

Coplen T.B., Herczeg A.L. & Barnes C., 2000. Isotope engineering: using stable isotopes of the water molecule to solve practical problems. In: Cook P.G. & Herczeg A.L. (eds.). *Environmental Tracers in Subsurface Hydrology*. Boston: Kluwer Academic Publishers.

Cox G. & Sealy J., 1997. Investigating identity and life histories: isotopic analysis and historical documentation of slave skeletons found on the Cape Town foreshore, South Africa. *International Journal of Historical Archaeology* **1**(3): 207-224.

Craig H, 1961. Isotopic variations in meteoric water. *Science* **133**: 1702-1703.

Craig H. & Gordon L., 1965. Deuterium and oxygen 18 variations in the ocean and the marine atmosphere. In: *Symposium on marine geochemistry*. Graduate School of Oceanography, University of Rhode Island, Occ. Publ. NQ 3:27.

Currey J.D., 2002. *Bones, Structure and Mechanics*. Princeton and Oxford: Princeton University Press. 5.

Curtain P.D., 1995. *Death by Migration, Europe's encounter with the tropical world in the nineteenth century*. Cambridge: Cambridge University Press.

Cyrulnik B., Matignon K.L. & Fougea F., 2003. *Tiere und Menschen. Die Geschichte einer besonderen Beziehung.* New York: Knesebeck Verlag.

Czermak A. & Ledderose A., 2004. Getrennt und Gemeinsam – Zur gessellschaftlichen Gliederung eines frühmittelalterlichen Separatfriedhofs. Erste Ergebnisse einer archäologisch-anthropologischen Synthese. In: Grupe G. & Peters J. (eds.). *Documenta Archaeobiologiae, Conservation policy and current research*. Rahden/Westf.: Maria Leidorf GmbH. 71-95.

Czermak A., Czermak A., Ernst H. & Grupe G., 2006. A new method for the automated age-at-death evaluation by tooth-cementum annulation (TCA). *Anthropologischer Anzeiger* **64**(1): 25-40.

Dalai T., Bhattacharya S.K. & Krishnaswami, 2002. Stable isotopes in the source waters of the Yamuna and its tributaries: seasonal and altitudinal variations and relation to major cations. *Hydrological Processes* **16**(17): 3345-3364.

Dansgaard W., 1964. Stable isotopes in precipitation. *Tellus* **16**: 436-468.

Dart R.A., 1957. The osteodontokeratic culture of Australopithecus prometheus. *Transvaal Museum-Memoir* **10**: 87. Pretoria: Transvaal Museum.

Darwent C.M. & Lyman R.L., 2002. In: Haglund W.D. & Sorg M.H. (eds.). *Advances in Forensic Taphonomy, Method, Theory, and Archaeological Perspective*s. Boca Raton, London, New York, Washington D.C.: CRC Press. 355-377.

Dempster D.W., 1999. New Concepts in Bone Remodeling. In: Seibel M.J., Robins S.P. & Bilezikian J.P. (eds.). *Dynamics of Bone and Cartilage Metabolism*. San Diego: Academic Press. 261-273.

DeNiro M.J., 1985. Post-mortem preservation and alteration of in vivo bone collagen isotope ratios in relation to palaeodietary reconstruction. *Nature* **317**: 806-809.

DeNiro M.J., 1987. Stable isotopy and archaeology. *American Scientist* **75**: 182-191.

DeNiro M.J. & Epstein S., 1978a. Influence of diet on the distribution of carbon isotopes in animals. *Geochimica et Cosmochimica Acta* **42**: 495-506.

DeNiro M.J. & Epstein S., 1978b. Carbon isotopic evidence for different feeding patterns in two Hyrax species occupying the same habitat. *Science* **201**: 906-907.

DeNiro M.J. & Epstein S., 1981. Influence of diet on the distribution of nitrogen isotopes in animals. *Geochimica et Cosmochimica Acta* **45**: 341-351.

DeNiro M.J., Schoeninger M.J. & Hastorf C.A., 1985. Effect of heating on the stable carbon and nitrogen isotope ratios of bone collagen. *Journal of Archaeological Science* **12**: 1-7.

Dittmann K. & Grupe G., 2000. Biochemical and palaeopathological investigations on weaning and infant mortality in the early Middle Ages. *Anthropologischer Anzeiger* **58**(4): 345-355.

Dopsch A., 1930. The early social and economic history of the peasants in the alpine districts of Austria. Abstract of chapter III: The era of the wandering nations. Translation from the German of chapters IV and V by Pennybacker R.: 1-30.

Dorozynski A. & Anderson A., 1991. Collagen; a new probe into prehistoric diet. *Science* **254**: 520-521.

Dupras T.L. & Schwarcz H.P., 2001. Strangers in a strange land: Stable isotope evidence for human migration in the Dakhleh Oasis, Egypt. *Journal of Archaeological Science* **28**(11): 1199-1208.

Dupras T, Schultz M., Wheeler S. & Williams L.J., 2006. *Forensic recovery of Human Remains, Archaeological Approaches*. Boca Raton, London, New York, Washington D.C.: CRC Press.

Efremov I.A., 1940. Taphonomy: a new branch of paleontology. *Pan-American Geologist.* **74**: 81-93.

Eichhorn B., 2006. Charcoal analysis and vegetation reconstruction in arid areas – a case study from northern Namibia. In: Grupe G. & Peters J. (eds.). *Documenta Archaeobiologiae 4. Microscopic Examinations of Bioarchaeological Remains, Keeping a close eye on ancient tissues*. Rahden/Westf.:Verlag Marie Leidorf GmbH. 189-205.

Epstein S., Buchsbaum R., Lowenstam H.A. & Urey H.C., 1953. Revised carbonate-water isotopic temperature scale. *Bulletin of the Geological Society America* **62**: 417-426.

Ericson J.E., 1989. Some problems and potentials for strontium isotope analysis for human and animal ecology. In: Rundel J.R.E.P.W. & Nagy K.A. (eds.). *Stable isotopes in ecological research*. New York (NY): Springer Verlag. 252-259.

Ericson J.E., Sullivan C.H. & Boaz N.T., 1981. Diets of Pliocene mammals from Omo, Ethiopia, deduced from carbon isotopic ratios in tooth apatite. *Palaeogeography, Palaeoclimatology, Palaeoecology* **36**: 69-73.

Ericson J.E., West M., Sullivan C.H. & Krueger H.W., 1989. The development of maize agriculture in the Viru Valley, Peru. In: Price T.D. (ed.). *The Chemistry of Prehistoric Human Bone.* Cambridge: Cambridge University Press: 68-104.

Evans J.A., Chenery C.A & Fitzpatrick A.P., 2006. Bronze Age childhood migration of individuals near Stonehenge, revealed by strontium and oxygen isotope tooth enamel analysis. *Archaeometry* **48** (2): 309-321.

Ezzo J.A., Johnson C.M. & Price T.D., 1997. Analytical perspectives on prehistoric migration: a case study from East Central Arizona. *Journal of Archaeological Science* **24**: 447-466.

Fairchild I.J., Killawee J.A., Sharp M.J., Spiro B., Hubbard B., Lorrain R.D. & Tison J.L., 1999. Solute generation and transfer from a chemically reactive alpine glacial-proglacial system. *Earth Surface Processes and Landforms* **24**(13): 1189-1211.

Faure G., 1986. *Principles of Isotope Geology*. New York: Wiley & Sons. 598.

Fazekas I.G. & Kósa F., 1978. *Forensic Fetal Osteology*. Budapest: Akadémiai Kiadó. 372-384.

Fine D. & Craig G.T., 1981. Buccal surface wear of human premolar and molar teeth: a potential indicator of dietary and social differentiation. *Journal of Human Evolution* **10**: 335-44.

Fogel M.L., Tuross N. & Owsley D., 1989. Nitrogen isotope tracers of human lactation in modern and archaeological populations. *Annual Report of Director, Geophysical Laboratory, Carnegie Institution of Washington 1988-1989*. 111-116.

Foster D., 1994. Diabetes mellitus. In: Isselbacher J., Braunwald E., Wilson J.D., Martin J.B., Fauci A.S. & Kasper D.L., (eds.). *Harrison's Principles of Internal Medicine*. 1979-2000.

Fricke H.C., O'Neil J.R. & Lynnerup N., 1995. Oxygen isotope composition of human tooth enamel from medieval Greenland; linking climate and society. *Geology* **23**(10): 869-872.

Fricke H., Clyde W. & O'Neil J., 1998. Intra-tooth variations in $\delta^{18}O$ (PO_4) of mammalian tooth enamel as a record of seasonal variation in continental climate variables. *Geochemica et Cosmochimica Acta* **62**: 1839-1850.

Gannes L.Z., O'Brien D.M. & Martinez del Rio C., 1997. Stable isotopes in animal ecology: caveats, and a call for more laboratory experiments. *Ecology* **78**(4): 1271-1276.

Garvie-Lok S., Varney T. & Katzenberg M., 2004. Preparation of bone carbonate for stable isotope analysis: the effects of treatment time and acid concentration. *Journal of Archaeological Science* **31**: 763-776.

Gleirscher P., 1991. *Die Raeter*. Raetisches Museum Chur, Engadin Press AG. Samedan. 1-20.

Global History of Health Project. (http://global.sbs.ohio-state.edu/)

Gokhan K., Keklikoglu N. & Buyukertan M., 2004. Comparison of the thickness of the cementum layer in Type 2 diabetic and non-diabetic patients. *Journal of Contemporary Dental Practice* **5**(2):124-133.

Green T.J., Cochran B., Fenton T.W., Woods J.C., Titmus G.L., Tieszen L., Davis M.A. & Miller S.J., 1998. The Buhl Burial: A Paleoindian Woman from Southern Idaho. *American Antiquity* **63**(3): 437-456.

Grue H. & Jensen B., 1979. Review of the formation of incremental lines in tooth cementum of terrestrial mammals. *Danish Review of Game Biology* **11**: 165-187.

Grupe G., 1985. Die >>Ressource Frau<<- Aussagemöglichkeiten der Biowissenschaft. In: W. Affeldt (ed.), *Frauen in Spätantike und Frühmittelalter, Lebensbedingungen - Lebensnormen - Lebensformen*. Sigmaringen: Thorbecke Verlag. 105-114.

Grupe G., 1987. Ernährungsgewohnheiten ur- und frühgeschichtlicher Bevölkerungen. Ergebnisse der Analyse von Spurenelementen und stabilen Isotopen an Knochen. *Veroffentlichung Übersee-Museum* A9. 147-163.

Grupe G., 1988a. Impact of the choice of bone samples on trace element data in excavated human skeletons. *Journal of Archaeological Science* **15**: 123-129.

Grupe G., 1988b. Aussagen der Elementanalysen von Skelettfunden für die Prähistorische Anthropologie. Vortrage auf dem Symposium: Analyse von Spurenelementen und stabilen Isotopen. *Homo* **39**: 61-65.

Grupe G., 1998. Diet and health depicted from human skeletal remains by archaeometry. *Rivista di Antropologia*. Supplement **76**: 121-131.

Grupe G., 2001. Archaeological Microbiology. In: Brothwell D.R. & Pollard A.M. (eds). *Handbook of Archaeological Sciences*: 351-58. New York: Wiley & Sons, Ltd.

Grupe G. & Piepenbrink H., 1989. Note on microbial influence on stable carbon and nitrogen isotopes in bone. *Applied Geochemistry* **4**: 299.

Grupe G., Price T.D., Schröter P., Söllner F., Johnson C. & Beard B., 1997. Mobility of Bell Beaker people revealed by stable strontium isotope ratios of teeth and bones. A study of southern Bavarian skeletal remains. *Applied Geochemistry* **12**: 517-525.

Grupe G. & Turban-Just S., 1998. Amino acid composition of degraded matrix collagen from archaeological human bone. *Anthropologischer Anzeiger* **56**(3): 213-226.

Grupe G. & Schweissing M.M., 2001. Stable isotope analysis of fossil bone. *Anthropologie* **29** (2-3): 109-116.

Grupe G., Mikić Ž., Peters J. & Manhart H., 2003. Vertebrate food webs and subsistence strategies of Mesolithic and Neolithic populations of central Europe. In: Grupe G. & Peters J. (eds.). *Documenta Archaeobiologiae, Decyphering ancient bones: The research potential of bioarchaeological collections Vol. 1*. Rahden/Westfahlen: Verlag Marie Leidorf GmbH. 193-213.

Grupe G., Christiansen K., Schröder I. & Wittwer-Backofen U., 2005. *Anthropologie. Ein einführendes Lehrbuch*. Berlin: Springer Verlag. 72-88.

Grupe G. & Mekota A.M., 2005. Stable isotope analysis of archaeological avian bones. In: Grupe G. & Peters J., (eds.). *Documenta Archaeobiologiae, Feathers, grit and symbolism. Birds and humans in the ancient Old and New Worlds Vol. 3*. Rahden/Westf.: Verlag Marie Leidorf GmbH. 62.

Grzesik W.J., Narayanan A.S., 2002. Cementum and periodontal wound healing and regeneration. *Crit. Rev. Oral Bio. Med.* **13**(6): 474-484.

Gulson B.L., Jameson C.W. & Gillings B.R., 1997. Stable lead isotopes in teeth as indicators of past domicile- a potential new tool in forensic science? *Journal of Forensic Science* **42**: 787-791.

Haglund W.D., 2003. Forensic Taphonomy. In: James S.H. & Nordby J.J. (eds.). *Forensic Science, An Introduction to Scientific and Investigative Techniques*. Boca Raton, London, New York, Washington D.C.: CRC Press. 99-112.

Haglund W.D., Conner M. & Scott D.D., 2002. The Effect of Cultivation on Buried Human Remains. In: Haglund W.D. & Sorg M.H. (eds.). *Advances in Forensic Taphonomy, Method, Theory, and Archaeological Perspective*s. Boca Raton, London, New York, Washington D.C.: CRC Press. 133-150.

Hall R.L., 1967. More about Corn, Cahokia and Carbon-14. Presentation at Cahokia Field Conference, Collinsville.

Hare P.E. & Estep M.L.F., 1983. Carbon and nitrogen isotopic composition of amino acids in modern and fossil collagens. *Carnegie, Institute Washington Yearbook* **82**: 410-414.

Hare P.E., Fogel M.L., Stafford T.W., Mitchell A.D. & T.C. Hoering, 1991. The isotopic composition of carbon and nitrogen in individual amino acids isolated from modern and fossil proteins. *Journal of Archaeological Science* **18**: 272-292.

Hassan F.A., 1981. *Demographic Archaeology*. New York: Academic Press Inc. 1-6.

Haynes C.V., 1968. Radiocarbon: Analysis of inorganic carbon of fossil bone and enamel. *Science* **161**: 687-688.

Heaton T.H.E., 1986. Isotopic studies of nitrogen in the hydrosphere and atmosphere: A review. *Chemical Geology* (Isotope Geoscience) **59**: 87-102.

Heaton T.H.E., Vogel J.C., Chevallarie G. & Collett G., 1986. Climatic influence on the isotopic composition of bone nitrogen. *Nature* **322**: 822-823.

Hedges R.E.M., 2000. Appraisal of radiocarbon dating of Kiore bones (Pacific rat *Rattus exulans*). *New Zealand Journal of the Royal Society* **30** (4): 385-398.

Hedges R.E.M., 2002. Bone diagenesis: an overview of processes. *Archaeometry* **44** (3): 319-328.

Hedges R.E.M., 2003. On bone collagen - apatite-carbonate isotopic relationships. *International Journal of Osteoarchaeology* **13**: 66-79.

Herrmann B., Grupe G., Hummel S., Piepenbrink H. & Schutkowski H., 1990. *Prähistorische Anthropologie, Leitfaden der Feld- und Labormethoden*. Berlin: Springer Verlag. 52-73.

Hildebrand M., 1995. *Analysis of Vertebrate Structure 4th ed*. New York: John Wiley & Sons, Inc. 109-118.

Hiraguchi T., Hiroko K. & Nobuyuki N., 1991. Stable carbon isotopic analysis of human skeletons from Mawaki, Akaura and Kamiyamada. *Journal of the Anthropological Society of Nippon* **99** (2): 194.

Ho S.P., Balooch M., Goodis H.E., Marshall G.W. & Marshall S.J., 2004. Ultrastructure and nanomechanical properties of cementum dentin junction. *Journal of Biomedical Materials Research* **68A**: 343-351.

Hobson K.A. & Collier S., 1984. Marine and terrestrial protein in Australian aboriginal diets. *Current Anthropology* **25**: 238-240.

Hocquet J.C., 1995. Salz. In: Angermann N. et al. (eds.). *Lexikon des Mittelalters Band VII.* München: LexMa Verlag. 1323-1327.

Hodell D., Quinn R., Brenner M. & Kamenov G., 2004. Spatial variation of strontium isotopes ($^{87}Sr/^{86}Sr$) in the Maya region: a tool for tracking ancient human migration. *Journal of Archaeological Science* **31**: 585-601.

Hoefs J., 1997. *Stable Isotope Geochemistry 4th edition*. Berlin: Springer Verlag.

Hoogewerff J., Papesch W., Kralik M., Berner M., Vroon P., Miesbauer H., Gaber O., Künzel K.H. & Kleinjans J., 2001. The Last Domicile of the Iceman from Hauslabjoch: A Geochemical Approach Using Sr, C and O Isotopes and Trace Element Signatures. *Journal of Archaeological Science* **28**(9): 983-989.

Houck M.M., Ubelaker D., Osley D., Craig E., Grant W., Fram R., Woltanski T. & Sandness K., 1996. The role of forensic anthropology in the recovery and analysis of Branch Davidian compound victims: assessing the accuracy of age estimations. *Journal of Forensic Science* **41**(5): 796-801.

Hubatschek E., 1990. *Bauernwerk in den Bergen*. Bozen: Frangart. 50.

Hug E., 1940. Die Schädel der frühmittelalterlichen Gräber aus dem solothurnischen Aarzgebiet in ihrer Stellung zur Reihengräberbevölkerung Mitteleuropas. Ein Beitrag zum Problem der Europäischen „Brachycephalie“. *Zeitschrift für Morphologie und Anthropologie, Erb und Rassenbiologie* **38**: 359-528.

Iacumin P., Bocherens H., Chaix L. & Marioth, 1998. Stable carbon and nitrogen isotopes as dietary indicators of ancient Nubian populations (Northern Sudan). *Journal of Archaeologcial Science* **25**(4): 293-301.

International Atomic Energy Agency (IAEA), 1969-1994. Environmental Isotope Data No. 1-10: World Survey of Isotope Concentration in Precipitation.

Jäger H., 1997. Transhumanz. In: Angermann N. et al. (eds.). *Lexikon des Mittelalters. Band VIII*. München: LexMa Verlag GmbH. 942-943.

Kagerer P. & Grupe G., 2001a. On the validity of age-at death diagnosis by incremental line counts in human dental cementum. Technical considerations. *Anthropologischer Anzeiger* **59**(4): 331-342.

Kagerer P. & Grupe G., 2001b. Age-at-death diagnosis and determination of life-history parameters by incremental lines in human dental cementum as an identification aid. *Forensic Science International* **118**(1): 75-82.

Kaplan F.S., Haddad J.G. & Singer F.R., 1994. Paget’s disease: Complications and controversies. *Calcified Tissue International* **55**(2): 75-78.

Kasseroler A., 1959. *Das Urnenfeld von Volders*. Innsbruck: Universitätsverlag Wagner. 232-234.

Kasseroler A., 1956? *Der vorgeschichtliche Weiler auf dem Himmelreichbühel*. (Date of publication, publisher and city of publication not listed).

Katzenberg M.A., 1989. Stable isotope analysis of archaeological faunal remains from southern Ontario. *Journal of Archaeological Science* **16**: 319-329.

Katzenberg M.A., 1992. Advances in stable isotope analysis of prehistoric bones. In: Saunders S.R. & Katzenberg M.A. (eds.). *Skeletal Biology of Past Peoples: Research Methods*. New York: Wiley-Liss. 105-119.

Katzenberg M.A. & Pfeiffer S., 1995. Nitrogen isotope evidence for weaning age in a nineteenth century Canadian skeletal sample. In: Grauer A.L. (ed.). *Bodies of Evidence, reconstructing history through skeletal analysis.* New York: Wiley- Liss. 221-235.

Keegan W.F., 1989. Stable isotope analysis of prehistoric diet. In: Iscan M.Y. & Kennedy K.A.R. (eds.), Reconstruction of Life from the Skeleton. New York: Alan R. Liss. 223-236.

Keegan W.F. & DeNiro M.J., 1988. Stable carbon and nitrogen isotope ratios of collagen used to study coral-reef and terrestrial components of prehistoric Bahamian diet. *American Antiquity* **53**: 320-336.

Kennedy B.V.E., 1988. Variation in $\delta^{13}C$ values of postmedieval Europeans. Ph.D. dissertation. Department of Archaeology, University of Calgary.

Klevezal G.A. & Kleinenberg S.E., 1967. Age determination of mammals from annual layers in teeth and bones. Translation by Israel Program Scientific Translations, Jerusalem, Israel 1969.

Knudson K., Price T., Buikstra J. & Blom D., 2004. The use of strontium isotope analysis to investigate Tiwanaku migration and mortuary ritual in Bolivia and Peru. *Archaeometry* **46**: 5.

Koch P., 1998. Isotopic reconstruction of past continental environments. Annual Review of Earth & Planetary Sciences **26**: 573-613.

Koch P., Behrensymeyer A., Tuross N. & Fogel M., l990. Isotopic fidelity during bone weathering and burial. Annual report of the director, Geophysical Laboratory, Carnegie Institution, Washington, 1989-1990: 105-119.

Koch P., Zachos J. & Gingerich P., 1992. Correlation between isotope records in marine continental carbon reservoirs near the Paleocene/Eocene boundary. *Nature* **358**: 319-322.

Koch P.L., Fogel M.L. & Tuross N., 1994. Tracing the diets of fossil animals using stable isotopes. In: Lajtha K. & Michener R.H. (eds.). *Stable Isotopes in Ecology and Environmental Science*. Oxford: Blackwell Scientific. 63-92.

Kohn M.J., 1996. Predicting animal $\delta^{18}O$: accounting for diet and physiological adaptation. *Geochimica et Cosmochimica Acta* **60**: 4811-4829.

Kohn M.J., 1999. You are what you eat. *Science* **283**: 335-336.

Kohn M.J., Schoeninger M.J. & Valley J.W., 1996. Herbivore tooth oxygen isotope compositions: effects of diet and physiology. *Geochimica et Cosmochimica Acta* **60**: 3889-3896.

Konstam A., 2002. *Atlas der Kelten*. Wien: Tosa Verlag. 14-15.

Krueger H., 1991. Exchange of carbon with biological apatite. *Journal of Archaeological Science* **18**: 355-361.

Krueger H.W. & Sullivan C.H., 1984. Models for carbon isotope fractionation between diet and bone. In: Turnlund J.E. & Johnson P.E. (eds.). *Stable Isotopes in Nutrition*. Wash. D.C.: American Chemical Society, Symposium Series **258**: 205-222.

Langenscheidt F., 1985. Methodenkritische Untersuchungen zur Paläodemographie am Beispiel zweier fränkischer Gräberfelder. *Materialien zur Bevölkerungswissenschaft* Sonderheft **2**: 80-86.

Lanting J.N., Aerts-Bijima A. & van der Pflicht H., 2001. Dating of cremated bones. *Radiocarbon* **43**(2): 1.

Larsen C.S., 1997. *Bioarchaeology. Interpreting human behavior from the human skeleton*. Cambridge: Cambridge University Press. 334-337.

Latkoczy C., Prohaska T., Stingeder G. & Teschler-Nicola M., 1998. Strontium isotope ratio measurements of prehistoric human bone samples by means of HR-ICPMS. *Journal of Analytical Atomic Spectrometry* **13**: 561.

Lee-Thorp J.A., 1989. Stable Carbon Isotopes in Deep Time. The Diets of Fossil Fauna and Hominids. Ph.D. dissertation. Archaeology Department, University of Cape Town.

Lee-Thorp J.A., 2002. Two decades of progress towards understanding fossilization processes and isotopic signals on calcified tissue minerals. *Archaeometry* **44**(3): 435-446.

Lee-Thorp J.A. & van der Merwe N.J., 1987. Carbon isotope analysis of fossil bone apatite. *South African Journal of Science* **83**: 712-715.

Lee-Thorp J.A., Sealy J.C. & van der Merwe N.J., 1989a. Stable carbon isotope ratio differences between bone collagen and bone apatite, and their relationship to diet. *Journal of Archaeological Science* **16**: 585-599.

Lee-Thorp J.A., van der Merwe N.J. & Brain C.K., 1989b. Isotopic evidence for dietary differences between two extinct baboon species from Swartkrans. *Journal of Human Evolution* **18**: 183-189.

Lee-Thorp J.A & van der Merwe N.J., 1991. Aspects of the chemistry of modern and fossil biological apatites. *Journal of Archaeological Science* **18**: 343-354.

Lee-Thorp J.A., van der Merwe N.J. & Brain C.K., 1994. Diet of Australopithecus robustus at Swartkrans from stable carbon isotopic analysis. *Journal of Human Evolution* **27**: 361-372.

Lee-Thorp. J.A., Manning L. & Sponheimer M., 1997. Problems and potential for very small samples of fossil tooth enamel. *Bulletin de la Societe Geologique de France* **168**: 767-73.

Lee-Thorp J.A., Sponheimer M. & van der Merwe N.J., 2003. What do stable isotopes tell us about Hominid Dietary and Ecological Niches in the Pliocene? *International Journal of Osteoarchaeology* **13**: 104-113.

Leitner W., 2001. Steinzeitlicher Bergkristalabbau am Riepenkar in den Tuxer Alpen. *Archaeologische Forschungen und Grabungsberichte aus Tirol, Archaeo Tirol* **3**:188.

Leslie P.W. & Gage T.B., 1989. Demography and Human Population Biology: Problems and Progress. In: Little M.A. & Haas J.D. (eds.). *Human Population Biology. A Transdisciplinary Science*. New York: Oxford University Press. 15-44.

Levinson A.A., Luz B. & Kolodny, 1987. Variations in oxygen isotopic compositions of human teeth and urinary stones. *Applied Geochemistry* **2**(4): 367-371.

Lillie M.C. & Richards M., 2000. Stable isotope analysis and dental evidence of diet at the Mesolithic - Neolithic transition in Ukraine. *Journal of Archaeological Science* **27**: 965-972.

Lipsinic F.E., Paunovich E., Houston G.D. & Robinson S.F., 1986. Correlation of age and incremental lines in the cementum of human teeth. *Journal of Forensic Science* **31**: 982-989.

Lippert A., 1989. Nordtirol in der Spätantike und im frühen Mittelalter. In: Lippert A. & Spindler K. (eds.), *Frühes leben in den Alpen. Ausgrabungen und Forschungen des Institutes für Ur- und Frühgeschichte der Universität Innsbruck: Begleitheft zur Ausstellung.* Innsbruck: Eigenverlag des Institutes für Ur- und Frühgeschichte. 69-84.

Lippitsch A., in preperation. Masters thesis at the Ludwig-Maximilians University, Munich, Germany.

Little M.A., Thomas R.B., Mazess R.B. & Baker P.T., 1971. Population differences and developmental changes in extremity temperature responses to cold among Andean Indians. Human Biology 43: 70-91.

Longinelli A., 1973. Preliminary oxygen-isotope measurements of phosphate from mammal teeth and bones. *Colloque International du CNRS* **219**: 267-271.

Longinelli A., 1984. Oxygen isotopes in mammal bone phosphate: A new tool for paleohydrological and paleoclimatological research. *Geochimica et Cosmochimica Acta* **48**(2): 385-390.

Lowenstam H.A. & Weiner S., 1989. *On biomineralization*. New York: Oxford University Press.

Lubell D, Jackes M., Knyf M. & Meiklejohn C., 1994. The Mesolithic-Neolithic transition in Portugal: Isotopic and dental evidence of diet. *Journal of Archaeological Science* **21**: 201-216.

Luz B., Kolodny Y. & Horowitz M., 1984. Fractionation of oxygen isotopes between mammalian bone-phosphate and environmental drinking water. *Geochimica et Cosmochimica Acta* **48**(8): 1689-1693.

Luz B. & Kolodny Y., 1989. Oxygen isotope variation in bone phosphate. *Applied Geochemistry* **4**(3): 317-323.

Luz B., Cormie A.B. & Schwarcz H.P., 1990. Oxygen isotope variations in phosphate of deer bones. *Geochimica et Cosmochimica Acta* **54**(6): 1723-1728.

Lyman R.L., 1994. *Vertebrate Taphonomy*. Cambridge: Cambridge University Press. 404-433.

Lyman R.L., 2002. Foreword. In: Haglund W.D. & Sorg M.H. (eds.). *Advances in Forensic Taphonomy, Method, Theory, and Archaeological Perspective*s. Boca Raton, London, New York, Washington D.C.: CRC Press. xix.

Marean C.W., 1991. Measuring the post-depositional destruction of bone in archaeological assemblages. *Journal of Archaeological Science* **18**: 677-694.

Maréchal J.C. & Etcheverry D., 2003. The use of ^{3}H and ^{18}O tracers to characterize water inflows in Alpine tunnels. *Applied Geochemistry* **18**(3): 339-351.

Marin D.L., Goodman A.H. & Armelagos G.J., 1985. Skeletal Pathologies as Indicators of Quality and Quantity of Diet. In: Gilbert R.I. & Mielke J.H. (eds.). *The Analysis of Prehistoric Diets*. Academic Press, Inc.

Marino B.D. & McElroy M.B, 1991. Isotopic composition of atmospheric CO_2 inferred from carbon in C4 plant cellulose. *Nature* **349**: 127-131.

Martin R.B., Burr D.B. & Sharkey N.A., 1998. *Skeletal Tissue Mechanics*. New York: Springer. 39-40.

Martin-Kilcher S., 1993. Römische Gräberfunde als Quelle zur Trachtgeschichte im zirkumalpin Raum. In: Struck M. (ed.). *Römerzeitliche Gräber als Quellen zu Religion, Bevölkerungsstruktur und Sozialgeschichte*. Archäologische Schriften des Instituts für Vor- und Frühgeschichte der Johannes Gutenberg-Universität Mainz. 181-203.

Masters P.M., 1987. Preferential preservation of noncollagenous protein during bone diagenesis: Implications for chronometric and stable isotopic measurements. *Geochimica et Cosmochimica Acta* **51**: 3209-3214.

Matson R.G. & Chisholm B., 1991. Basketmaker II subsistence: carbon isotopes and other dietary indicators from Cedar Mesa, Utah. *American Antiquity* **56**: 444-459.

Mays S.A., 1997. Carbon stable isotope ratios in mediaeval and later human skeletons from Northern England. *Journal of Archaeological Science* **24**(6): 561-567.

Mays S., 1998. *The Archaeology of Human Bones*. London: Routledge.

McCarthy E.F. & Frassica F.J., 1998. *Pathology of Bone and Joint Disorders with Clinical and Radiographic Correlation*. Philadelphia: W.B. Saunders Co. 35.

McGovern-Wilson R. & Quinn C., 1996. Stable isotope analysis of ten individuals from Afetna, Saipan, Northern Mariana Islands. *Journal of Archaeological Science* **23**(1): 59-65.

Meindl R.S. & Lovejoy C.O., 1985. Ectocranial suture closure: A revised method for the determination of skeletal age at death based on the lateral-anterior sutures. *American Journal of Physical Anthropology* **68**: 57-66.

Meyer W., 1998. Besiedlung und wirtschaftliche Nutzung hochalpiner Zonen in der mittelalterlichen Schweiz. In: Spindler K. (ed.). *Mensch und Natur im mittelalterlichen Europa, archäologische, historische und naturwissenschaftliche Befunde*, Band 4. Klagenfurt: Wieser Verlag. 231-260.

Millard A., Lucy S. & Roberts C., 2004. Direct evaluation of archaeological immigration, population dynamics and lead exposure by isotope biogeochemistry. An NERC project at the University of Durham, http://www.dur.ac.uk.

Minagawa M. & Wada E., 1984. Stepwise enrichment of ^{15}N along food chains: further evidence and the relationship between $\delta^{15}N$ and animal age. *Geochimica et Cosmochimica Acta* **48**: 1135-1140.

Montgomery J., Evans J. & Budd P., 2000. Sr and Pb isotopes for tracking human historical and ancient migrations. *Journal of Conference Abstracts* **5**(2): 715.

Moran E.F., 2000. *Human Adaptability, An Introduction to Ecological Anthropology*, 2nd ed. Boulder: Westview Press.

Moser H., 1984. Volders eine Wanderung durch dreijahtausende. Volders. Volders: Gemeinde Volders.

Müller A.H., 1963. *Lehrbuch der Paläozoologie, allgemeine Grundlagen*, *Band 1*. Jena: Gustav Fischer Verlag.

Müller W., 2004. *Tier- und Humanphysiology. Eine Einführung*. Auflage 2. Berlin, Heidelberg: Springer-Verlag. 240.

Müller W., 2005. *Isotopic tracing in Archaeometry*. Research School of Earth Sciences. The Australian National University, Canberra, ACT 0200, iummix.terra.unimi.it/www./scuolagnm/mueller.html.

Nawrocki S.P., 1995. Taphonomic processes in historic cemeteries. In: Grauer A.L. (ed.) *Bodies of Evidence. Reconstructing history through skeletal analysis*. New York: John Wiley & Sons, Inc. 49-66.

Naylor J.W., Miller W.G., Stokes G.N. & Stott G.G., 1985. Cemental annulation enhancement: a technique for age determination in man. *American Journal of Physical Anthropology* **68**: 197-200.

Newell C., 1988. *Methods and Models in Demography*. London: Belhaven Press. 9-10.

Newsome S., Philips D., Culleton B., Guilderson T. & Koch P., 2004. Dietary reconstruction of an early to middle Holocene human population from the central California coast: insights from advanced stable isotope mixing models. *Journal of Archaeological Science* **31**: 1101-1115.

Nicholson R.A., 2001. Taphonomic Investigations. In: Brothwell D.R. & Pollard A.M. (eds.). *Handbook of Archaeological Sciences*. Chichester: John Wiley & Sons, Ltd. 179-190.

Noebl A., 1960. *Bezirkskunde Innsbruck-Land*. Der Bezirk in seiner Landschaft, Kultur, Wirtschaft und Verwaltung. Innsbruck: Tyrolia. 33-39.

Norr L., 1981. Prehistoric Costa Rican diet as determined from stable carbon isotope ratios in bone collagen. *American Journal of Physical Anthropology* **54**: 258-259.

Norr L., 1991. Nutritional consequences of prehistoric subsistence strategies in lower Central America. Ph.D. dissertation. University of Illinois, Urbana, Illinois.

Norr L., 1995. Interpreting dietary maize from stable isotopes in the American tropics: the state of the art. In: Stahl P.W. (ed): *Archaeology in the Lowland American Tropics: Current analytical methods and applications*. Cambridge: Cambridge University Press. 198-223.

Norton D. & Panichi C., 1978. Determination of the sources and circulation paths of thermal fluids: the Abano region, northern Italy. *Geochimica et Cosmochimica Acta* **42**(8): 1283-1294.

OIPC: The Online Isotopes in Precipitation Calculator, 2004. http://es.ucsc.edu/~gbowen.

O'Leary M.H., 1981. Carbon isotopic fractionation in plants. *Phytochemistry* **20**(4): 553-567.

O'Leary M.H., 1988. Carbon isotopes in photosynthesis. *BioScience* **38**: 328-336.

Parfitt A.M., 1983. The physiologic and clinical significance of bone histomorphometric data. In: Becker R.R. (ed.), *Bone Histomorphometry: techniques and interpretation*. Boca Raton, Florida: CRC Press. 143-223.

Pate F.D., 1994. Bone chemistry and paleodiet. *Journal of Archaeological Method and Theory* **1**(2): 161-209.

Peterson B.J. & Fry B., 1987. Stable isotopes in ecosystem studies. *Annual Review of Ecology and Systematics* **18**: 292-320.

Pfeiffer S., 2006. Cortical Bone Histology in Juveniles. In: Grupe G. & Peters J. (eds.). *Documenta Archaeobiologiae Vol. 4*, *Bioarchaeological Remains Under the Microscope*. Rahden: Verlag Maria Leidorf GmbH. 15-28.

Pickering T.R., Clarke R.J. & Moggi-Cecchi J., 2004. Role of carnivores in the accumulation of the Sterkfontein Member 4 Hominid assemblage: A taphonomic reassessment of the complete hominid fossil sample (1936-1999). *American Journal of Physical Anthropology* **125**: 1-15.

Pingitore N.E., 2000. Synchrotron studies of strontium in ancient human bones. Workshop on synchrotron radiation in Art and Archaeology. Stanford Synchrotron Radiation Laboratory, Palo Alto, CA., Presentation Oct. 18.

Pittioni R, 1973. Alm- (Alp) wirtschaft, der Süden. In: Hoops J. (ed.). *Reallexicon der Germanischen Altertumskunde Band 1*. Berlin, New York: Walter De Gruyter & Co. 181-183.

Price T.D., 1989. Bones, Chemistry and the Human Past. In: Price T.D. (ed.). *The Chemistry of Prehistoric Human Bone*. Cambridge: Cambridge University Press. 1-9.

Price T.D., Schoeninger M.J. & Armelagos G.J., 1985. Bone Chemistry and Past Behavior. *Journal of Human Evolution* **14**: 419-447.

Price T.D., Johnson C.M., Ezzo J.A., Ericson J. & Burton J.H., 1994a. Residential mobility in the Prehistoric Southwest United States: A preliminary study using strontium isotope analysis. *Journal of Archaeological Science* **21**: 315-330.

Price T.D., Grupe G. & Schröter P., 1994b. Reconstruction of migration patterns in the Bell Beaker period by stable strontium isotope analysis. *Applied Geochemistry* **9**: 413-417.

Price T.D., Grupe G. & Schröter P., 1998. Migration and mobility in the Bell Beaker period in Central Europe. *Antiquity* **72**: 405-441.

Price T.D, Manzanilla L. & Middleton W.D., 2000. Immigration and the ancient city of Teotihuacan in Mexico: a study using strontium isotope ratios in bone and teeth. *Journal of Archaeological Science* **27**: 903-913.

Price T.D., Bentley R.A., Luening J., Gronenborn D. & Wahl J., 2001. Prehistoric human migration in the Linearbandkeramik of Central Europe. *Antiquity* **75**: 593-603.

Price T.D., Burton J., Bentley R.A., 2002. The characterization of biological available strontium isotope ratios for the study of Prehistoric migration. *Archaeometry* **44**: 117.

Prowse T., Schwarcz H., Saunders S., Macchiarelli R. & Bondioli L., 2003. Isotopic paleodiet studies of skeletons for the Imperial Roman-age cemetery of Isola Sacra, Rome, Italy, *Journal of Archaeological Science* **31**: 259-272.

Prowse T., Schwarcz H., Saunders S., Macchiarelli R. & Bondioli L., 2005. Isotopic evidence for age-related variation in diet from Isola Sacra, Italy. *American Journal of Physical Anthropology* **12**: 2-13.

Quade J., Cerling T.E., Barry J.C., Morgan M.E., Pilbeam D.R., Chivas A.R., Lee-Thorp J.A. & Van der Merwe N.J., 1992. Carbon isotope ratios of apatite from fossil bone cannot be used to reconstruct diets of animals. *Nature* **297**: 577-578.

Quade J., Cerling T.E., Andrews P. & Alpagut B., 1995. Palaeodietary reconstruction of Miocene faunas from Pasalar, Turkey using stable carbon and oxygen isotopes of fossil tooth enamel. *Journal of Human Evolution* **28**: 373-384.

Ramsl P., 2004. Migration phenomena in the early La Tène period. *Antiquity* **78:** 299.

Reed D.M., 1998. Ancient Maya diet at Copan, Honduras. Ph.D. dissertation. The Pennsylvania State University.

Redlich O., 1886. Traditionsbücher des Hochstifts Brixen. vom zehnten bis in das vierzehnte Jahrhundert. 29V, 30R.

Renfrew C. & Bahn P.G., 1996. *Archaeology: Theories, Methods, and Practice*, 2nd edition. London: Thames and Hudson, Ltd.

Renz H. & Radlanski R.J., 2006. Incremental lines in root cementum of human teeth-A reliable age marker? *Homo* **57**: 29-50.

Relethford J., 1999. *The Human Species: An Introduction to Biological Anthropology*. Fourth edition. Mountain View: Mayfield. 155.

Richards M.P., 1996. The Puzzle over Neolithic Diet. *British Archaeology* **12**: 6.

Richards M.P. & Mellars P., 1998. Stable isotopes and the seasonality of the Oronsay middens. *Antiquity* **72**(275): 178-184.

Richards M.P., Hedges R.E.M., Molleson T.I. & Vogel J.C., 1998. Stable isotope analysis reveals variations in human diet at the Poundbury Camp cemetery site. *Journal of Archaeological Science* **25**(12): 1247-1252.

Richards M.P. & Hedges R.E.M., 1999a. A Neolithic revolution? New evidence of diet in the British Neolithic. *Antiquity* **73**: 891-897.

Richards M.P. & Hedges R.E.M., 1999b. Stable isotope evidence for similarities in the types of marine foods used by late Mesolithic humans at sites along the Atlantic coast of Europe. *Journal of Archaeological Science* **26**(6): 717-722.

Richards M.P., Pettitt P.B., Trinkaus E., Smith F.H., Paunovic M. & Karavanic I., 2000. Neanderthal diet at Vindija and Neanderthal predation: The evidence from stable isotopes. *The Proceedings of the National Academy of Sciences, USA* **97**(13): 7663-7666.

Richards M.P., Mays S. & Fuller B.T., 2002. Stable carbon and nitrogen isotope values of bone and teeth reflect weaning age at the medieval Wharram Percy site, Yorkshire, UK. *American Journal of Physical Anthropology* **119**: 205-210.

Rossert J. & Crombrugge B., 1996. Type I collagen: Structure, synthesis, and regulation. In: Bilezikian J.P., Raisz L.G. & Rodan G.A. (eds.), *Principles of Bone Biology*. 127-142.

Rozanski K., Aragus-Aragus L. & Gonfiantini R., 1993. Isotopic patterns in modern global precipitation. In: Swart P.K. (ed.). *Climate change in continental isotopic records*, Geophysical Monograph. Washington: AGU. 1-36.

SAHRA (Sustainability of Semi-arid Hydrology and Riparian Areas), 2006. http://www.Sahra.arizona.edu.

Sauer M., Schweingruber F., Vaganov E.A., Shiyatov S.G. & Siegwolf R., 2002. Spatial and temporal oxygen isotope trends at the northern tree-line in Eurasia. *Geophysical Research Letters* **29**(9):10.1-10.4.

Schenk W. & Eichfeld I., 2006. Viehhaltung und Weidewirtschaft. In: Beck H., Geuenich D. & Steuer H. (eds.). *Reallexikon der Germanischen Altertumskunde Band 32*. Berlin: Walter de Gruyter GmbH & Co. KG. 348-355.

Schoeller D.A., 1999. Isotopic fractionation: why aren't we what we eat? *Journal of Archaeological Science* **26**: 667-674.

Schoeninger M.J., 1985. Trophic level effects on $^{15}N/^{14}N$ and $^{13}C/^{12}C$ ratios in bone collagen and strontium levels in bone mineral. *Journal of Human Evolution* **14**: 515-525.

Schoeninger M.J., 1989a. Reconstructing prehistoric human diet. In: Price T.D. (ed.). *The Chemistry of Prehistoric Human Bone*. Cambridge: Cambridge University Press. 38-67.

Schoeninger M.J., 1989b. Reconstructing prehistoric human diet. *Homo* **39**(2):78-99.

Schoeninger M.J. & DeNiro M.J., 1982. Carbon isotope ratios of apatite from fossil bone cannot be used to reconstruct diets of animals. *Nature* **297**: 577-578.

Schoeninger M.J., DeNiro M.J. & Tauber H., 1983. Stable isotope ratios of bone collagen reflect marine and terrestrial components of prehistoric diet. *Science* **220**: 1381-1383.

Schoeninger M.J. & DeNiro M.J., 1984. Nitrogen and carbon isotope composition of bone collagen from marine and terrestrial animals. *Geochimica et Cosmochimica Acta* **48**: 625-639.

Schoeninger M.J. & Moore K., 1992. Bone stable isotope studies in archaeology. *Journal of World Prehistory* **6**(2): 247-296.

Schoeninger M.J., Kohn M.J. & Valley J.W., 2000. Tooth oxygen isotope ratios as palaeoclimate monitors in arid ecosystems. In: Ambrose S. & Katzenberg M.A. (eds.). *Biogeochemical Approaches to Paleodiet Analysis*. New York: Kluwer Academic/Plenum Publishers. 117-140.

Schurr M.R., 1997. Stable nitrogen isotopes as evidence for the age of weaning at the Angel Site: a comparison of isotopic and demographic measures of weaning age. *Journal of Archaeological Science* **24**(10): 919-927.

Schutkowski H., 1987. Sex determination of fetal and neonate skeletons by means of discriminant analysis. *International Journal of Anthropology* **2**: 347-342.

Schwarcz H.P., Melbye J., Katzenberg M.A. & Knyf M., 1985. Stable isotopes in human skeletons of southern Ontario: reconstructing paleodiet. *Journal of Archaeological Science* **12**: 187-206.

Schwarcz H.P. & Schoeninger M., 1991. Stable isotope analysis in human nutritional ecology. *Yearbook of Physical Anthropology* **34**: 283-321.

Schwarcz H.P., Gibbs L. & Knyf M., 1991. Oxygen isotope analysis as an indicator of place of origin. In: Pfeiffer S. & Williamson R. (eds.). *Snake Hill: An Investigation of a Military cemetery from the War of 1812*. Toronto: Dundurn Press. 263-268.

Schweissing M. & Grupe G., 2003. Stable strontium isotopes in human teeth and bone: a key to migration events of the late Roman period in Bavaria. *Journal of Archaeological Science* **30**: 1373-1383.

Scott S. & Duncan C.J., 1998. *Human Demography and Disease*. Cambridge: Cambridge University Press.

Sealy J., van der Merwe N.J., Lee-Thorp J.A. & Lanham J.L., 1987. Nitrogen isotope ecology in southern Africa: implications for environmental and dietary tracing. *Geochimica et Cosmochimica Acta* **51**: 2707-2717.

Sealy J.C. & van der Merwe N.J., 1988. Social, spatial and chronological patterning in marine food use as determined by ^{13}C measurements of Holocene human skeletons from southwestern Cape, South Africa. *World Archaeology* **20**: 87-102.

Sealy J.C., Armstrong R. & Schrire C., 1995. Beyond lifetime averages: tracing life histories through isotopic analysis of different calcified tissues from archaeological human skeletons. *Antiquity* **69**: 290-300.

Shackelton N. & Renfrew C., 1970. Neolithic trade routes re-aligned by oxygen isotope analysis. *Nature* **228**:1062-1065.

Shipman P., 1981. *Life History of a Fossil, An Introduction to Taphonomy and Paleoecology*. Cambridge, Massachusetts and London, England: Harvard University Press.

Sillen A., 1998. Diagenesis of the inorganic phase of cortical bone. In: Price T.D. (ed.). *The chemistry of prehistoric bone*. Cambridge: Cambridge University Press. 211-229.

Sillen A. & Lee-Thorp J.A., 1994. Trace element and isotopic aspects of predator-prey relationships in terrestrial foodwebs. *Palaeogeography, Palaeoclimatology, Palaeoecology* **107**: 243-55.

Sillen A., Hall G., Richardson S. & Armstrong R., 1998. $^{87}Sr/^{86}Sr$ ratios in modern and fossil food-webs of Sterkfontein Valley: Implications for early hominid habit preference. *Geochimica et Cosmochimica Acta* **62**: 2463-2473.

Sorg M.H. & Haglund W.D., 2002. Advancing Forensic Taphonomy: Purpose, Theory, and Process. In: Haglund W.D. & Sorg M.H. (eds.). *Advances in Forensic Taphonomy, Method, Theory, and Archaeological Perspective*s. Boca Raton, London, New York, Washington D.C.: CRC Press. 3-29.

Spindler K., 2002. Living in High Mountain Regions – how humans conquered the Alps. In: Ackerl I. (ed.). *The Wonderful World of Mountains*. Vienna: Federal Press Services. 58-72.

Sponheimer M. & Lee-Thorp J.A., 1999a. Isotopic evidence for the diet of an early hominid, Australopithecus africanus. *Science*: 368-370.

Sponheimer M. & Lee-Thorp J.A., 1999b. Oxygen isotopes in enamel carbonate and their ecological significance. *Journal of Archaeological Science* **26**: 723-728.

Sponheimer M., Robinson T., Ayliffe L., Roeder B., Hammer J., Passey B., West A., Cerling T., Dearing D. & Ehleringer J., 2003. Nitrogen isotopes in mammalian herbivores: Hair $\delta^{15}N$ values from a controlled feeding study. *International Journal of Osteoarchaeology* **13**: 80-87.

Steckel R.H. & Rose J.C. (eds.), 2002. *The Backbone of History: Health and Nutrition in the Western Hemisphere.* Cambridge: Cambridge University Press.

Sternberg L., DeNiro M.J. & Johnson H.B., 1984. Isotopic ratios of cellulose from plants having different photosynthetic pathways. *Plant Physiology* **74**: 557-561.

Štih P., 1998. Alpine colonization and migrations in the Middle Ages with Slovenia as an example. *Istituto di Storia delle Alpi ISAlp-English summaries* **4**, www.isalp.unisi.ch.

Stloukal M. & Hanáková H., 1978. Die Länge der Langknochen altslavischer Bevölkerungen unter Berücksichtigung von Wachstumsfragen. *Homo* **29**: 53-69.

Stott G.G., Sis R.F. & Levy B.M., 1982. Cemental annulation as an age criterion in forensic dentistry. *Journal of Dental Research* **61:** 814-817.

Struck M., 1993. Kinderbestattungen in romano-britischen Siedlungen – der archäologische Befund. In: Struck M. (ed.). *Römerzeitliche Gräber als Quellen zu Religion, Bevölkerungstruktur und Sozialgeschichte.* Archäologische Schriften des Instituts für Vor- und Frühgeschichte der Johannes Gutenberg-Universität, Mainz. 313-318.

Suchey J.M. & Katz D., 1986. Skeletal age standards derived from an extensive multi-racial sample of modern Americans (Abstract). *American Journal of Physical Anthropology* **51**: 517-540.

Sullivan C.H. & Krueger H.W., 1981. Carbon isotope analysis in separate chemical phases in modern and fossil bone. *Nature* **292**: 333-335.

Tauber H., 1981. ^{13}C evidence for dietary habits of prehistoric man in Denmark. *Nature* **292**: 332-333.

Telkkä A., Palkama A. & Virtama P., 1962. Prediction of stature from radiographs of long bones in children. Journal of Forensic Science 7: 474-479-

Ten Cate A.R., 1972. An analysis of Tome's granular layer. *The Anatomical Record* **172(**2): 137-147.

Thackeray J.F, van der Merwe N.J, Lee-Thorp J.A, Sillen A., Lanham J.L., Smith R., Keyser A. & Montioro P.M.S., 1990. Changes in carbon isotope ratios in the late Permian recorded in Therapsid tooth apatite. *Nature* **347**: 751-753.

Thomas R.B., 1976. Energy flow at high altitude. In: Baker P.T. & Little M.A. (eds.). *Man in the Andes: A multidisciplinary study of high-altitude Quechua*. Stroudsburg, Pa: Dowden, Hutchinsin & Ross. 379-404.

Tieszen L.L. & Fagre T., 1993. Effect of diet quality and composition on the isotopic composition of respiratory CO_2, bone collagen, bioapatite, and soft tissues. In: Lambert J.B. & Grupe G., *Prehistoric Human Bone: archaeology at the molecular level*. New York: Springer-Verlag. 121-155.

Trueman C., Chenery C., Eberth D.A. & Spiro B., 2003. Diagenetic effects on the oxygen isotope composition of bones of dinosaurs and other vertebrates recovered from terrestrial and marine sediments. *Journal of the Geological Society*. www.looksmartscience.com

Tudge A.P.A., 1960. Method of analysis of oxygen isotopes in orthophosphate-its use in the measurement of paleotemperatures. *Geochimica et Cosmochimica Acta* **19**: 81-93.

Tuross N., Fogel M.L. & Hare P.E., 1988. Variability in the preservation of the isotopic composition of collagen from fossil bone. *Geochimica et Cosmochimica Acta* **52**: 929-935.

Tuross N., Fogel M.L., 1994. Stable isotope analysis and subsistence patterns at the Sully site. In: Owsley D.W. & Jantz R.L. (eds.), *Skeletal Biology in the Great Plains: Migration, Warfare, Health, and Subsistence*. Washington, DC: Smithsonian Institute Press. 283-289.

Ubelaker D.H., 1989. *Human Skeletal Remains: Excavation, Analysis, Interpretation*, 2nd ed. Washington D.C.: Taraxacum. 44-95.

Ubelaker D.H., Katzenberg M.A. & Doyon L.G., 1995. Status and diet in precontact Highland Ecuador. *American Journal of Physical Anthropology* **97**: 403-411.

Ulijaszek S.J., Johnston F.E. & Priece M.A., 1998. *The Cambridge Encyclopedia of Human Growth and Development.* Cambridge: Cambridge University Press. 356.

Urey H.C., 1947. The thermodynamic properties of isotopic substances. *Journal of the Chemical Society* May: 562-581.

Urzi C. & Krumbein W.E., 1994. Microbiological impacts on the cultural heritage. In: Krumbein W.E., Brimblecombe P., Cosgrove D.E. & Staniforth S. (eds.). *Durability and Change. The Science, Responsibility, and Cost of Sustaining Cultural Heritage*: Chichester: John Wiley. 107-135.

Van Klinken G.J., Richards M.P. & Hedges R.E.M., 2000. An overview of causes for stable isotopic variations in past European human populations: environmental, ecophysiological, and cultural effects. In: Ambrose S.H. & Katzenberg M.A. (eds.). *Biochemical Approaches to Paleodietary Analysis*. New York: Kluwer Academic/Plenum Pub.

Van der Merwe N.J., 1982. Carbon isotopes, photosynthesis, and archaeology. *American Scientist* **17**: 596-606.

Van der Merwe N.J. & Medina E., 1991. The canopy effect, carbon isotope ratios, and foodwebs in Amazonia. *Journal of Archeological Science* **18**: 249-259.

Vogel J.C., 1980. *Fractionation of the carbon isotopes during photosynthesis*. Berlin, Heidelberg, New York: Springer Verlag. 1-29.

Vogel J.C. & van der Merwe N.J., 1977. Isotopic Evidence for Early Maize Cultivation in New York State. *American Antiquity* **42**: 238-242.

Vrba E.S., 1985. Ecological and adaptive changes associated with early hominid evolution. In: Delson E. (ed.) *Ancestors: The Hard Evidence*. New York: Alan R. Liss. 63-71.

Walker P.L. & DeNiro M.J., 1986. Stable nitrogen and carbon isotope ratios in bone collagen as indices of prehistoric dietary dependence on marine and terrestrial resources in southern California. *American Journal of Physical Anthropology* **71**: 51-61.

Wang H., Ambrose S.H., Liu C.-L.J. & Follmer L.R., 1997. Paleosol stable isotope evidence for early hominid occupation of East Asian temperate environments. *Quaternary Research* **48**: 228-238.

Weekend Magazin, 2006. Spezialitäten vom Tiroler Alm- und Hofschwein. Nr. 24: 46-47.

Weigelt J., 1927. Rezente Wirbeltierleichen und ihre paläobiologische Bedeutung. Leipzig: Max Weg Verlag.

Wernicke I., 1989. *Die Kelten in Italien. Die Einwanderung und die frühen Handelsbeziehungen zu den Etruskern.* Stuttgart: Franz Steiner Verlag. 140-152.

Wheater P.R., Burkitt H.G. & Daniels V.G., 1987. *Functional Histology*. Edinburgh: Churchill Livingstone. 170-185.

White C.D., 1998. *Ancient Maya diet at Copan, Honduras*. Ph.D. dissertation, The Pennsylvania State University.

White C.D., Spence M.W., Stuart-Williams H.L.Q. & Schwarcz H.P., 1998. Oxygen isotopes and the identification of geographical origins: the Valley of Oaxaca versus the Valley of Teotihuacan. *Journal of Archaeological Science* **25**(7): 643-655.

White C.D., Longstaffe F.J., Spence M.W. & Law K.R., 2000. Teotihuacan state representation at Kaminaljuyú: Evidence from oxygen isotopes. *Journal of Anthropological Research* **56**: 535-558.

White C., Longstaffe F.J. & Law K.R., 2004. Exploring the effects of environment, physiology and diet on oxygen isotope ratios in ancient Nubian bones and teeth. *Journal of Archaeological Science* **31**(2): 233-250.

White T.D. & Folkens P.A., 1991. *Human Osteology*. San Diego: Academic Press, Inc.

Whittle A., 1996. *Europe in the Neolithic*. Cambridge: Cambridge University Press.

Wiedemann F.B., Bocherens H., Mariotti A., von den Driesch A. & Grupe G., 1999. Methodological and archaeological implications of intra-tooth isotopic variations (^{13}C, ^{18}O) in herbivores from Ain Ghazal (Jordan, Neolithic). *Journal of Archaeological Science* **26**: 697-704.

Wittwer-Backofen U., Gampe J. & Vaupel J., 2003. Tooth cementum annulation for age estimation: Results from a large known-age validation study. *American Journal of Physical Anthropology* **123**: 119-129.

Wright L.E. & Schwarcz H.P., 1998. Stable carbon and oxygen isotopes in human tooth enamel: Identifying breastfeeding and weaning in prehistory. *American Journal of Physical Anthropology* **106**: 1-18.

Wright L.E. & Schwarcz H.P., 1999. Correspondence between stable carbon, oxygen and nitrogen isotopes in human tooth enamel and dentine: infant diets at Kaminaljuyu. *Journal of Archaeological Science* **26**: 1159-1170.

Yakir D., 1992. Variations in the natural abundances of oxygen-18 and deuterium in plant carbonates. *Plant, Cell, and Environment* **15**: 1005–1020.

Zanesco A., 2005. Ausgrabungen am Beilstein, Mesolithikum bis Neuzeit. *Fundberichte aus Österreich* **43**, 2004, Wien: 10-14.

Zanesco A. & Stadler H., 2002. Volders, Frümittelalterliches Gräberfeld in der Augasse. *Kulturberichte aus Tirol* **55**, Denkmalbericht 2001, Innsbruck: 144-146.

Zanesco A. & Stadler H., 2003. Volders, Frümittelalterliches Gräberfeld in der Augasse. *Kulturberichte aus Tirol 431/432* **56**, Denkmalbericht 2002, Innsbruck: 155.

Zanesco A. & Stadler H., in preparation. Das frühmittelalterliche Gräberfeld von Volders/Augasse – der Grabungsbefund. In: Stadler H. (ed.) *Zwischen Totenruhe und Wissenschaft. 3500 Jahre Grabsitten in Volders*.

Zasso A, Lécuyer C., Sheppard S.M., Grandjean P. & Mariotti A., 2004. Diagenesis and the reconstruction of paleoenvironments: A method to restore original $\delta^{18}O$ values of carbonate and phosphate from fossil tooth enamel. *Geochimica et Cosmochimica Acta* **68**(10): 2245-2258.

Erklärung

Diese Dissertation wurde im Sinne von § 12 der Promotionsordnung von Frau Prof. Dr. Gisela Grupe betreut. Ich erkläre hiermit, dass die Dissertation nicht einer anderen Prüfungskommission vorgelegt worden ist und dass ich mich nicht anderweitig einer Doktorprüfung unterzogen habe.

Ehrenwörtliche Versicherung

Ich versichere hiermit ehrenwörtlich, dass die vorgelegte Dissertation von mir selbständig und ohne unerlaubte Hilfe angefertigt wurde.

München, den 01.02.2007

George McGlynn

Curriculum vitae

Name, Vorname: McGlynn, George Charles
Geburtsdatum: 16.09.1964
Geburtsort: San Francisco, California, USA
Staatsangehörigkeit: Österreich / USA

Schulische Ausbildung:

1974 - 1978	Herbert Hoover Junior High School
1978 - 1982	Lowell High School

Studienverlauf:

1982 - 1987	Bachelor of Arts degree, Anthropology, University of California at Santa Barbara
1987 - 1989	Master of Arts degree, Physical Anthropology, University of Oregon
1990 - 1991	Premed study, Santa Clara University und University of San Francisco
1993 - 1996	Medizinstudium an der Leopold-Franzens-Universität Innsbruck, Österreich
2002 - 2005	Promotionsstudium in Biologie, Ludwig-Maximilians-Universität München

Tätigkeiten:

1982 - 1987	Leichtathletik (Zehnkampf)
1984 - 1994	Tennis Lehrer, Santa Barbara und San Francisco
1988	Institute for Human Origins, Berkeley, California (Ferialjob)
1989 - 1990	Zimmermann; Cameron Craik & Sons, San Francisco
1990 - 1992	Zimmermann; Phillip Monjouz Co., San Francisco
1992	Student extern, R.K. Davies Medical Center (Surgical Unit), San Francisco
1992 - 1993	Hoch und Tiefbau; Amero Construction Inc.
1993 - 1999	Fassadengerüstbau; Just Leitern Firma
1994	Student extern, Kaiser Hospital (Katheter Labor), San Francisco
1999 - 2001	Stadt Archäologie, Hall in Tirol, freier Mitarbeiter
2001 - Jan. 2006	Selbstständige anthropologische und archäozoologische Arbeiten
Feb. 2006 -	Konservator bei der Staatssammlung für Anthropologie und Paläoanatomie München

Hobbies:

Sport, Antikmöbelrestaurierung, Kochen, Angeln, Gitarre spielen, Reisen, Gartenarbeit

Acknowledgments

My enduring thanks go out to my parents, Ingeborg and George Sr., who's enthusiasm for my changing interests throughout life, patience with me when I failed or was being impossible, belief in me as an individual, and love for their son irregardless of the situation never ceased and provided me with the support that helped me realize many of my personal goals and imbued me with a sense of self assurance and confidence in my abilities that were necessary to make it on my own.

Warmest thanks to my wonderful and talented fiancé Gitti, who always stood by me, not only when the sun was shining but also when storm clouds brewed and the rain turned to deluge. Her strength and perseverance often gave me the drive I needed to reach the finish line. She is a truly beautiful person whose presence in my life allows me to look forward to each new day.

My special thanks also go to Prof. Gisela Grupe, mentor and colleague, who gave me a new chance and lifted the blinds to a world of science within the discipline of anthropology that I was not aware of. Her fiercely inquisitive nature and superlative example as a scientific investigator and teacher are contagious and boosted my motivation in a way like Mary Shelley's creation being jolted into life.

A heartfelt thanks to my friend Prof. Harald Stadler for putting a foot in the door for me, getting me jobs during my days as a self-employed pauper, and organizing the Volders project and keeping it afloat. Without his unwavering commitment this work would not have been possible.

Thanks to Dr. Alexander Zanesco for much of the archaeological documentation described herein, including the use of his excavation plans as well as interpretations of stratification and the like. Most of all I thank him since he gave me my first job at an archaeological dig in Europe, the rest is history.
